# Origin of the RNA World in Cold Hadean Geothermal Fields Enriched in Zinc and Potassium: Abiogenesis as a Positive Fallout from the Moon-Forming Impact?

**DOI:** 10.3390/life15030399

**Published:** 2025-03-04

**Authors:** Armen Y. Mulkidjanian, Daria V. Dibrova, Andrey Y. Bychkov

**Affiliations:** 1 Department of Physics, Osnabrueck University, D-49069 Osnabrueck, Germany; 2 Center of Cellular Nanoanalytics, Osnabrueck University, D-49069 Osnabrueck, Germany; 3 School of Bioengineering and Bioinformatics, Lomonosov Moscow State University, 119992 Moscow, Russia; 4 Belozersky Institute of Physico-Chemical Biology, Lomonosov Moscow State University, 119992 Moscow, Russia; 5 School of Geology, Lomonosov Moscow State University, 119992 Moscow, Russia; bychkov@geol.msu.ru

**Keywords:** origin of lifey, prebiotic chemistry, Hadean, redox potential, lipid, oxygen fugacity, RNA World, warm little pond, metallic zinc, anoxic geothermal fields, UV light, photoselection of nucleotides

## Abstract

The ubiquitous, evolutionarily oldest RNAs and proteins exclusively use rather rare zinc as transition metal cofactor and potassium as alkali metal cofactor, which implies their abundance in the habitats of the first organisms. Intriguingly, lunar rocks contain a hundred times less zinc and ten times less potassium than the Earth’s crust; the Moon is also depleted in other moderately volatile elements (MVEs). Current theories of impact formation of the Moon attribute this depletion to the MVEs still being in a gaseous state when the hot post-impact disk contracted and separated from the nascent Moon. The MVEs then fell out onto juvenile Earth’s protocrust; zinc, as the most volatile metal, precipitated last, just after potassium. According to our calculations, the top layer of the protocrust must have contained up to 10^19^ kg of metallic zinc, a powerful reductant. The venting of hot geothermal fluids through this MVE-fallout layer, rich in metallic zinc and radioactive potassium, both capable of reducing carbon dioxide and dinitrogen, must have yielded a plethora of organic molecules released with the geothermal vapor. In the pools of vapor condensate, the RNA-like molecules may have emerged through a pre-Darwinian selection for low-volatile, associative, mineral-affine, radiation-resistant, nitrogen-rich, and polymerizable molecules.

## 1. Introduction and Background

It is impossible to understand life without elucidating its origin. Unraveling the origin of life is also important because it can explain many puzzling but universal properties of living systems. This aspect can be viewed also from another angle: the plausibility of origin-of-life scenarios must be judged by their ability to explain where these universal properties of life came from.

Most of these properties are inconsistent with the geochemistry of modern Earth. Consequently, cumulative analyses of the universal but enigmatic properties of living systems can help reconstruct the primordial environments conducive to the mass production of would-be biomolecules and, ultimately, the emergence of life.

Since there was no “biology” on the lifeless Earth, the origin of life is also called abiogenesis [1]; most likely, an interplay of certain geochemical and (photo)chemical reactions did spawn life. Earth’s geological record, however, is of little help in uncovering the geochemistry of abiogenesis. Due to the plate tectonics, Earth underwent continuous resurfacing. The oldest known crustal rocks (Acasta Gneiss Complex) are dated at 4.03 billion years (Ga) [2], meaning they are more than 500 million years (Ma) younger than the Earth itself that is 4.56–4.53 Ga old [3,4,5]. These first 500 Ma are called Hadean eon; from that time, no rocks, but only minute zircon grains, are left [6].

Zircon grains are small crystals of ZrSiO_4_ which, once formed in molten rock at depths of 5–10 km, are very refractory and therefore can withstand the later melting of the rock in which they are embedded, thus preserving information about the time and conditions of their formation [6,7,8,9]. Particularly interesting are the zircon grains from the Jack Hills of the Yilgarn Craton, Western Australia, that are 4.4–4.1 Ga old, see [10,11,12].

Remarkably, the zircon grains with ages of 4.25 Ga [13] and 4.1 Ga [14] contain inclusions of isotopically light ^13^C, indicating the presence of life in the Hadean. Thus, it appears that the biological chronicle is longer than the rock-hard geological record of Earth. Therefore, certain features common or relevant to all organisms can provide information about the Hadean [15,16,17,18].

In this sense, proceeding from the common features of life, we have previously endeavored to reconstruct early evolutionary events in contexts of photochemistry [19,20,21,22], biogeochemistry [23,24], comparative genomics [25,26,27,28,29,30,31], and bioenergetics [32,33,34,35,36,37,38,39]. In particular, some time ago we focused on the well-established fact that the composition of inorganic components inside the cell is very similar in all living organisms, but different from the chemical composition of the environments in which these organisms currently live [40,41]. The cytoplasm of all active cells contains ten times more potassium ions (K^+^) than sodium ions (Na^+^), as well as high concentrations of phosphate and transition metals. We found these properties to be inherent to the condensate of geothermal vapor. This finding allowed us to propose the origin of the first cells, and perhaps of life itself, in the pools of condensed vapor covering the primordial anoxic geothermal fields [23,24].

Here, we additionally draw on lunar rock geochemistry data that have recently provided insight into the formation of the Moon 4.4–4.5 Ga ago from a giant impact [42]. Thus, we rely on the common traits of life, lunar geochemistry, and the properties of Hadean zircons. Relevant background information is given in Section 1.1, Section 1.2, Section 1.3 and Section 1.4 below, followed, in the Results section, by the evolutionary reconstruction of the conditions on Hadean Earth conducive to the origin of life.

According to this reconstruction, based on thermodynamic modeling of the cooling protolunar disk and phylogenomic analysis of the oldest proteins, the top layer of the post-impact Earth’s protocrust must have incorporated up to 10^19^ kg of mostly metallic Zn (Zn^0^) and about 3.0 × 10^16^ kg of radioactive ^40^K, both capable of vigorously reducing CO_2_ and N_2_ to various organic molecules in the hot subsurface [43,44,45,46,47,48,49,50,51]. The nascent organics must then have escaped with the geothermal vapor enriched with catalytically active ions of K^+^, Zn^2+^, Mg^2+^ and Mn^2+^, as well as phosphorous compounds and molybdate. In the super-reduced pools of vapor condensate, these inorganic catalysts, aided by solar UV light and porous silica sinter templates, must have catalyzed various chemical transformations, eventually yielding the first ribonucleotide-like molecules. Under the UV light of the cold and faint Hadean Sun, such proto-ribonucleotides, owing to their photostability, must have selectively accumulated and assembled into oligomers capable of forming double helices, exceptionally resistant to radiation and hydrolysis. Ultimately, some of such RNA-like photostable consortia mastered self-copying, initiating the transition from (photo)chemical to Darwinian natural selection.

This may have happened 4.50–4.10 Ga ago, i.e., in the period when the upper layer of the protocrust still retained its high reducing capacity owing to the Zn^0^- and ^40^K-enriched post-impact fallout.

Our evolutionary reconstruction, however unfamiliar it may seem, is consistent with established views on the origin of life and is a further development of mainstream thinking in the field. We also consider the proposed scenario in relation to (i) the role of terrestrial volcanic systems in abiogenesis, (ii) the zinc-centricity of life, (iii) thermodynamic constraints, (iv) other anticipated giant impacts, (v) chances of the existence of life on Mars, (vi) the habitability criteria, and (vii) other origin-of-life models.

Addressing the Special Issue question “What is Life?”, we posit that life is a form of energy-driven self-organization of matter into discrete, self-assembling, self-recovering, and self-reproducing units that adapt to environmental changes through intrinsic heritable variability and evolve to use available resources as efficiently as possible.

Finally, we emphasize the feasibility of modeling the origin of life processes in experimental setups simulating the super-reduced, Zn- and K-rich Hadean geothermal systems.

### 1.1. Basics of Life

We want to reach as many interested readers as possible. Therefore, we provide some basic biological information for readers without biological background in Section S1 of the Supplementary File S1.

### 1.2. Paradoxes of Life

Life has many paradoxical features that are common to all organisms. Many of these traits have no conventional explanation; however, being so familiar and common, these features are taken for granted, and their oddness is usually not recognized. It is thought that resolving these paradoxes may help to unravel the circumstances of life’s origin [52,53,54,55]. Most of these origin-of-life paradoxes are known to the experts in the field. Still, for those readers who are not deeply involved, we review these paradoxes in Section S2 of the Supplementary File S1.

In most cases, we are not the first to identify these paradoxes, so we also review the solutions that have been proposed for them to date. In this way, we present the main scientifically plausible ideas about the origin of life.

In general, we encourage the reader to always keep the Supplementary File S1 open because we frequently refer to it throughout the text.

Some crucial information related to these paradoxes may be new even to experts in the field. This information is summarized in Section 1.3 below.

### 1.3. Common (Bio)Chemical Traits of Life

#### 1.3.1. Biomolecules and Their Abiotic Generation

**Figure 1:**
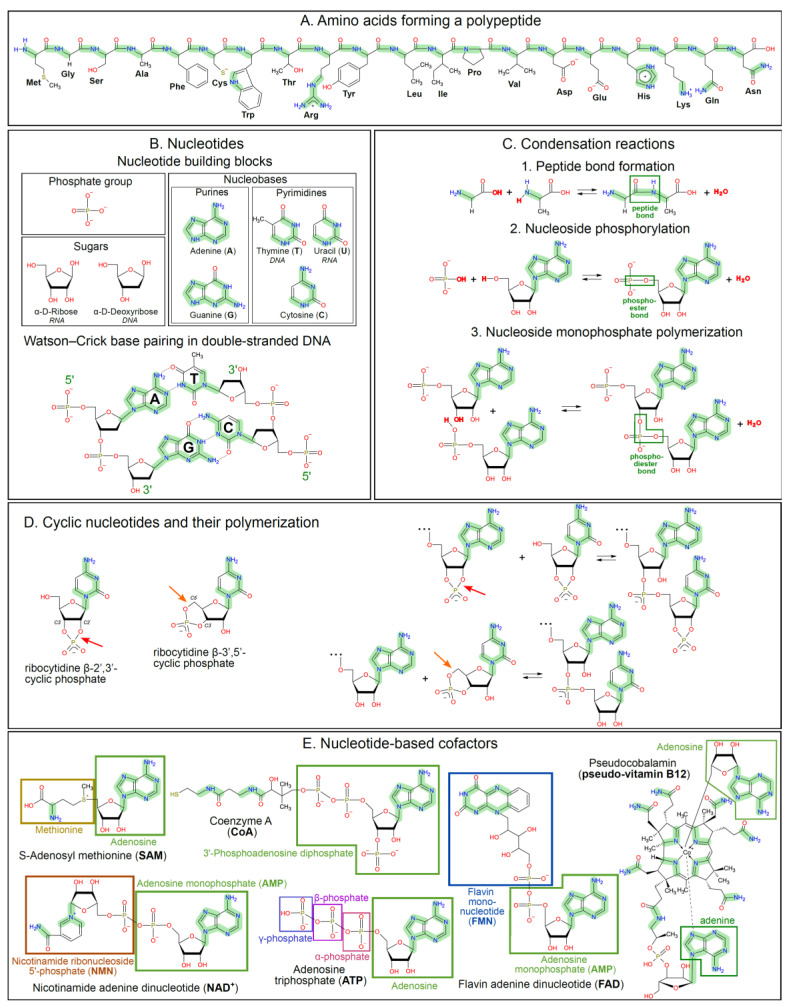
Biomolecules. The CN bonds are green shaded. (**A**) Amino acids and formation of a peptide bond between them; (**B**) nucleotides, their structure and complementary interactions; (**C**) condensation reactions; (**D**) cyclic nucleotides and mechanisms of their polymerization (modified from [56]); the red and orange arrows indicate the additional phosphoester bonds; and (**E**) ribonucleotide-containing enzyme cofactors.

Figure 1 depicts the main biomolecules in which the carbon and nitrogen atoms make bonds either with each other or with hydrogen atoms; to a lesser extent, if at all, they make bonds with oxygen atoms. This is a property biomolecules share with other organic molecules that are usually *reduced* in oxygen and, therefore, can give electrons to appropriate acceptors. The ability of a chemical compound to give/take electrons is characterized by its redox potential, as defined in Section S2.1 of the Supplementary File S1. Good electron donors/bad electron acceptors with high reducing power are characterized by lower, often negative redox potentials. Redox potential values are given relative to the so-called hydrogen electrode at which protons of water can be reduced to molecular hydrogen (H_2_); the redox potential of this reaction at 25 °C, a pressure of 1 atm is 0.0 V at pH 0.0 (E_0_), and −0.41 V at pH 7.0, see Section S2.1 for respective equations. The redox potential of the hydrogen electrode is considered as the low-potential limit of water stability; reducing agents with even lower redox potentials can decompose water into molecular hydrogen (H_2_) and OH^−^ anions.

**Figure 2:**
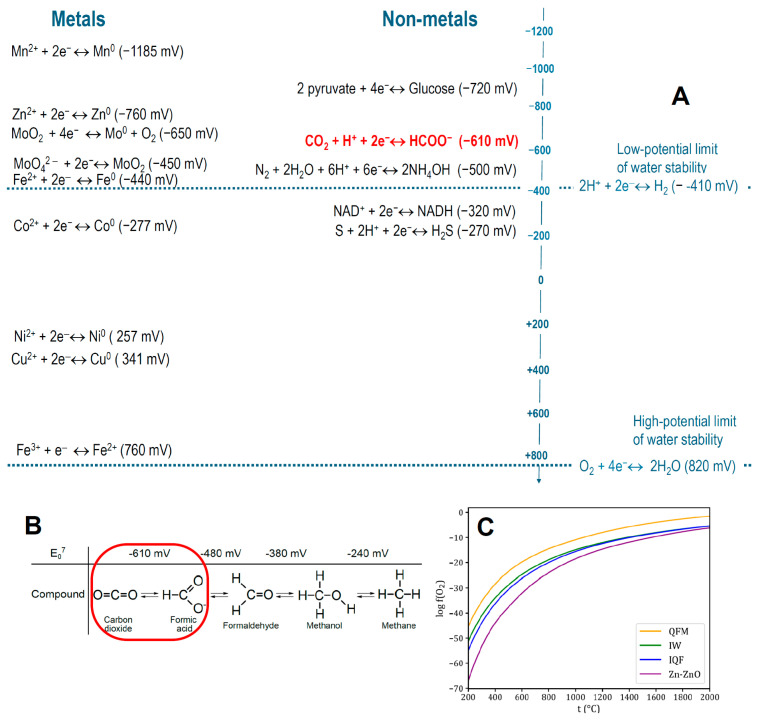
Redox potentials and oxygen fugacity *f*(O_2_). (**A**) Some biologically relevant redox half-reactions (redox pairs) with indicated values of their standard redox potential at pH 7.0 (E_0_^7^). The difference between the redox potentials of two half-reactions corresponds to the free energy of the redox reaction between them. Spontaneous electron transfer occurs when the redox potential of the electron-donating half-reaction is more negative than that of the electron-accepting half-reaction. The plot is based on data compiled from [57,58,59,60]. (**B**) Stepwise reduction of CO_2_ to methane, modified from [60]. (**C**) *f*(O_2_)–temperature diagram. Log oxygen fugacity vs. temperature at 1 bar pressure for common buffer assemblages, plotted using algorithms compiled by B. R. Frost [61]. The FMQ (fayalite–magnetite–quartz) buffer is characterized by the reaction 3Fe^2+^_2_SiO_4_ + O_2_ ↔ 2Fe^3+^_3_O_4_ + 3SiO_2_, the IW (iron–wustite) redox buffer is characterized by the reaction, [2(1-x)Fe^0^ + O_2_ ↔ 2Fe^2+^(1-x)O], and the IQF (iron–quartz–fayalite) buffer is characterized by the reaction 2Fe^0^ + SiO_2_ + O_2_ ↔ Fe^2+^_2_SiO_4_, whereas the Zn-ZnO buffer is characterized by the reaction 2Zn^0^ + O_2_ ↔ 2ZnO.

Figure 2A shows typical organic and inorganic redox half-reactions (redox pairs) with the values of their standard redox potential at pH 7.0 (E_0_^7^), which is considered as standard in biochemical literature. In the case of metals, the superscript indicates their redox state; it is not indicated below when ambiguous, or when the metal is simply listed as a chemical element.

The simplest known reaction of converting an inorganic compound into organics is the reduction of CO_2_ to formic acid, see the red-marked reaction in Figure 2A,B. This reaction requires very strong reducing agents with a redox potential of less than −0.61 V, which is outside the water stability range, as can be seen from Figure 2A. Another key reaction is the reduction of molecular nitrogen N_2_ to ammonia NH_3_ or ammonium NH_4_^+^; the redox potential of this reaction is ~−0.5 V, which is also outside the water stability range, see Figure 2A.

Consequently, the abiotic formation of organic molecules is hardly possible on the surface of the present-day water-bathed Earth under its oxygen-rich atmosphere. Therefore, most organic molecules are produced biogenically, predominantly through chlorophyll-based photosynthesis. It uses the energy of light to yield electron-donating compounds with redox potentials as low as ≤–0.7 V [62,63].

But organic molecules also seem to form abiotically in hot rocks, in a process called hydrothermal alteration. Earth’s rocks contain about 5% iron, mostly as wustite, FeO, or fayalite, 3Fe_2_SiO_4_. At high rock pressure and temperature, the redox potentials shown in Figure 2A shift so that some of the Fe^2+^ ions within the rock can be oxidized to Fe^3+^ by protons present in the geothermal fluids. This reaction produces magnetite (Fe_3_O_4_) and H_2_. Various organic molecules were shown to be produced from CO_2_ carried by geothermal fluid at t° ≤ 500 °C, albeit at low yields [64,65,66,67,68,69,70,71,72,73,74,75,76,77]. The underlying chemistry is thought to be related to the Fischer-Tropsch process of producing hydrocarbons from H_2_ and CO (or even CO_2_) at high temperature and pressure [78,79]. These abiotically produced organic molecules are transported to the surface by hot geothermal fluids.

This phenomenon of H_2_ formation in hot rocks allows the correlation of the redox potential scale for liquid systems at 25 °C and pH 7.0 (Figure 2A) with the reducing power of hot solid rocks, which is characterized not by the redox potential but by the oxygen fugacity, *f*(O_2_). The fugacity (*f*) is defined as the effective partial pressure of a gas (in this case, oxygen gas) in thermodynamic equilibrium with a given mineral assemblage, see Figure 2C and [61,80,81,82]. Typically, *f*(O_2_) is reported in log10 units relative to well-characterized mineral redox buffers, see Figure 2C and its caption. The *f*(O_2_) of today’s Earth’s crust typically corresponds to that of the fayalite-magnetite-quartz (FMQ) assemblage. Since the hot rocks reduce water protons to H_2_ at t° ≤ 500 °C, the reducing power of the FMQ assemblage at ~500 °C (log_10_*f*(O_2_) ~ −24, see Figure 2C) roughly corresponds to the reducing potential (power) of a hydrogen electrode at 25 °C, i.e., −0.41 V at pH 7.0, see Figure 2A and [61,80,81,82]. Consequently, the reducing power of other mineral assemblages shown in Figure 2C, such as iron-wustite (IW) and iron-quartz-fayalite (IQF), is much higher than that of the hydrogen electrode. The *f*(O_2_) value decreases with temperature (Figure 2C), so that at t° < 500 °C the H_2_-producing capacity of the crust even increases. However, at t° < 250 °C, redox reactions in the rock attenuate because of their high activation barriers [83].

Although *f*(O_2_) is defined in terms of oxygen partial pressure, the value of *f*(O_2_) is used as an integral parameter to characterize the reducing power of the entire rock [61,80,81,82]. The corresponding integral parameter for complex fluid mixtures, such as the Earth’s water reservoirs or cell cytoplasm, is the redox potential of the medium, E*_h_* [80,82,84,85]. It can be measured using a chemically inert platinum or gold electrode capable of exchanging electrons with all redox agents present in the solution [80,86,87].

Most modern natural reservoirs in contact with the atmosphere, including the oceans at all depths, have high and positive E*_h_* values in the range of +0.6 ÷ +0.8 V [84]. This is because the dissolved atmospheric oxygen usually acts as the dominant redox buffer. The reaction of reducing oxygen to water (E_0_^7^ = +0.82 V) is considered as the high-potential limit of the water stability, see Figure 2A.

In contrast to most water basins, the redox potential inside cells is rather low, around −0.3 V [88]. It is thought that the first cells initially lived in highly reduced habitats and then failed to adapt to the oxidation of their environment in response to the appearance of atmospheric oxygen [89]. Therefore, in most cases, cells must keep their cytoplasm much more reduced than the environment, which requires energy.

Apart from classical electrochemistry, CO_2_ and N_2_ can also be reduced by ionizing radiation, see Section S2.1 and [44,45,50,51]. This generates “solvated electrons” with high reducing power when interacting with water so that the irradiation of a CO_2_/water mixture produces organic molecules and H_2_ [90,91]. Although it is still unclear how the solvated electrons with an apparent E_0_^7^ of ~ −2.9 V are formed [92,93,94], their ability to promote the formation of carbon- and nitrogen-containing organic molecules has been demonstrated in various systems [95,96,97].

In general, it has been repeatedly shown that high-energy inputs, such as UV illumination, γ-irradiation, proton beams, or electrical discharges, can generate organic molecules from H_2_O, CO_2_, and N_2_, see [91,96,98,99,100,101,102,103,104,105,106,107,108] and references therein.

Yet, because organic molecules are hydrophobic on average, the products of such abiotic syntheses have mostly deposited on the walls of the experimental flasks as tar incapable of further chemical transformations.

This is also the case of the formose reaction where sugars (carbohydrates) and organic acids with four to seven carbon atoms (C4–C7 compounds) are obtained from C1–C3 aldehydes and/or alcohols; for details, see Section S2.2 and [109,110,111]. This reaction, discovered by Butlerov in 1859 [112], is special in producing complex organic molecules from simpler building blocks without energy input. The reaction can be additionally stimulated by UV light, in the presence of which it proceeds even with a C1 molecule of formaldehyde (COH_2_, see Figure 2B) as a sole substrate [113,114]. Still, the products of the formose reaction promptly “caramelize” to a nearly insoluble tar.

Benner and his colleagues showed that such caramelization can be prevented by adding high concentrations of borate to the solution. Polar borate anions (BO_2_^−^), by binding to the sugar molecules in a specific way, prevented their caramelization and drove the reaction toward the formation of C5 sugars, which increased the yield of ribose, a constituent of RNA; for details, see Section S2.2 and [115,116]. Most of the C5 sugars obtained in the presence of borate were branched, in contrast to ribose present in RNA. Therefore, Benner and his colleagues next showed that branched C5 sugars can be converted to ribose in the presence of molybdenum oxyanion (MoO_4_^2−^) as a catalyst; for details, see Section S2.2 and [117]. These data show that abiotically formed organic molecules can escape “asphaltization” [118] by specifically binding to and/or interacting with polar solvent components.

In contrast to sugars, nucleic acids and proteins are enriched in nitrogen, see Figure 1 and Sections S1.1 and S1.2. Therefore, proteins and nucleotides contain many bonds connecting carbon and nitrogen atoms (hereafter referred to as CN bonds and shaded green in Figure 1A–E).

Since the CN bonds are uncommon in inanimate nature, their presence in biomolecules is puzzling (see Section S2.3). Yet, Mukhin and his colleagues discovered CN bond-containing compounds in volcanic lava and attributed them to high-temperature chemical reactions in the throats of volcanoes [67]; in fact, the reaction of hydrogen cyanide (HCN) formation from methane and ammonia becomes thermodynamically favorable at T > 780 °C [119]. Also, the CN-bonds-containing molecules may have been formed in the primordial, CO_2_- and N_2_-containing atmosphere, as well as in ice under the action of UV light [120,121], solar wind [122], and lighting [99,107]; see Section S2.3. The CN bonds also form upon the dehydration of ammonium salts of organic acids, see Section S2.3 and [123,124].

As early as 1960, Oro showed that the nucleobase adenine (see Figure 1B) “assembles” from five HCN molecules on heating [125]. More recently, Sutherland and his colleagues have elucidated an entire network of chemical reactions leading from HCN to nucleotides, amino acids and some other important building blocks of life, see Section S2.3 and [126,127,128,129,130,131].

Remarkably, the addition of a water molecule to HCN yields formamide, HCONH_2_, which also forms upon the dehydration of ammonium formate, see Section S2.3. Formamide, which, thus, can be obtained by several routes, is a low-volatile compound that boils at 210 °C. Therefore, once formed, it must have persisted in a liquid state on the Hadean Earth, which property it shares with other low-molecular amides. It has been repeatedly shown that heating formamide, illuminating it with UV light or treating it with a proton beam yields nucleosides and amino acids; the reactions could be enhanced by certain minerals, see Section S2.3 and [96,97,108,132,133,134,135,136,137,138,139,140,141,142,143,144,145,146,147,148].

Thus, the abiotic formation of organic compounds, including the nitrogen-rich ones, is (electro)chemically challenging. Although there are several potentially feasible mechanisms known, the extent to which they may have contributed to the origin of life remains unclear.

#### 1.3.2. The Water Paradox

Paradoxically, water hinders the production of reduced organic molecules, since the oxidation of strong reducing agents by water protons (E_0_^7^ = −0.41 V) is thermodynamically more favorable than their oxidation by CO_2_ (E_0_^7^ ~ −0.6 V) or N_2_ (E_0_^7^ ~ −0.5 V), see Figure 2A. Consequently, researchers perform reductive syntheses of organic molecules—both in the liquid phase and on electrodes—either in anhydrous solutions or in mixtures with a small fraction of water [149,150].

Water also can hinder the assembly of complex biomolecules from building blocks, e.g., the formation of a nucleotide from a nucleobase, ribose, and a phosphate group, see Figure 1. These building blocks usually interact by a condensation mechanism where the bond formation is accompanied by the release of a water molecule, see Figure 1C and [53,151,152]. Also, the formation of biopolymers, such as RNA, DNA, proteins, and oligosaccharides, proceeds in the cell by a polycondensation mechanism. Condensation reactions are unfavorable in water which shifts the equilibria shown in Figure 1C to the left, see [53,151,152,153] for details. Therefore, in the cell, such condensations are driven by bioenergetic reactions [154].

Hence, since water (i) hinders the reductive formation of organic molecules and (ii) ultimately leads to the destruction of DNA, RNA, proteins, and polysaccharides, it could hardly have served as a suitable medium for the abiotic formation of biomolecules on the primordial Earth.

Therefore, to account for the abiotic formation of complex organic molecules and their polymers, many origin-of-life models consider fluctuating systems with wet/dry cycling, such as tidal zones, periodically drying pools, geothermal systems with fluctuating activity, deserts periodically wetted by rains, and so on [23,54,146,153,155,156,157,158,159].

Also considered are eutectic (water/ice) systems where water is temporarily removed from the reaction volume by freezing [160,161,162,163,164]; in such systems, the spontaneous polycondensation of nucleotides proceeds indeed [165,166,167].

Yet another option is to envision the formation of the first biopolymers in anhydrous solvents, specifically in formamide that can additionally serve as a building block for biomolecules, see Section S2.3 and [108,142,157].

#### 1.3.3. RNA World and the Extreme UV-Resistance of Natural Nucleosides and Nucleotides

Proteins are made with the participation of nucleic acids, while nucleic acids are synthesized by protein enzymes, which is a kind of chicken-and-egg paradox (see Sections S1.1 and S2.5). Currently, most biologists resolve this paradox by assuming that the emergence of proteins and DNA may have been preceded by an “RNA World” inhabited by small RNA-like polymers (oligoribonucleotides) and their consortia capable both of the information transfer and catalysis, see Sections S1.1 and S2.5–S2.7, and [164,165,166,167,168,169,170,171,172,173,174,175,176,177,178,179,180,181,182,183,184,185,186,187,188,189,190,191,192,193,194,195,196,197,198,199,200,201,202,203].

Notably, ribonucleotides, not only join to form RNA oligomers (Figure 1B), but are part of many enzyme cofactors (Figure 1E), which is seen as evidence for the emergence of these cofactors in the primordial RNA World [204,205]. Furthermore, ribonucleotides ATP and GTP serve as the main energy-storing compounds in the cell, see Figure 1E and Section S1.1.

Several RNA systems capable of multiplying via cooperation between short oligoribonucleotides have been obtained and studied experimentally [179,180,200,201,203]. Hereafter, we refer to this mode of assembly as “self-copying”, to distinguish it from “self-reproduction” which applies to the duplication of the whole content of a cell [206]. The self-copying may have preceded “self-replication”, i.e., the orderly sequential addition of individual nucleotides along an available polynucleotide template, as observed in modern cells, see Section S1.1.

In this context, particularly interesting are so-called cyclic ribonucleotides where the phosphate moiety is linked to ribose not by one but by two phosphoester bonds. Figure 1D shows natural β-2′,3′- and β-3′,5′-cyclic ribonucleotides, where the additional bonds are shown by red and orange arrows, respectively. Cyclic nucleotides are activated, i.e., their additional bonds “store energy” needed for polymerization. The free enthalpy of scission of these additional bonds (about −40 kJ/mol [207]) can be used for binding to another nucleotide, see Figure 1D and Section S2.5. Nonenzymatic polymerization to short oligonucleotides was demonstrated both for 2′,3′- and 3′,5′-cyclic ribonucleotides [147,148,208,209,210]. As shown in Figure 1D, the polymerization of cyclic nucleotides could proceed without water release under certain conditions.

The complexity of the primordial RNA-like molecules may have increased owing to their unique photochemical properties, see Sections S2.6 and S2.7 and [20] for details. Specifically, nucleotides (Figure 1B) strongly absorb UV light in the wavelength (λ) range of 260–280 nm. This property underlies the popular belief that solar UV quanta absorbed by nucleotides can cause hazardous DNA mutations. Actually, it is not that bad; DNA and RNA are exceptionally photostable; the energy of the UV quantum absorbed by the nitrogenous base of any of natural (canonical) nucleotides is converted into heat within femtoseconds, i.e., much faster than any destructive chemical reactions can occur, see Section S2.6 and [211,212,213,214,215].

In fact, canonical nucleotides, by intercepting and quenching the UV quanta, effectively protect RNA and DNA from the backbone breaks that can result from the interaction of the UV quanta with the linking phosphate groups, see Figure 1C,D and [216]. These phosphate groups, owing to their resonant structure, also absorb UV light in the same range of 260–280 nm, albeit very weakly [217,218]. Still, their photoexcitation generates reactive phosphate radicals that break the sugar–phosphate backbone with a very high quantum yield of about 0.5, i.e., in every second case, see Section S2.6 and [216,219] for further details.

Canonical nucleosides provide at least a 10^5^-fold protection of the sugar–phosphate backbone from the UV cleavage, see Section S2.6 and [20,216,220]. If it happens that a nucleobase gets damaged by an intercepted UV quantum, it is promptly replaced by the cellular repair systems, provided that the backbone remains intact [221].

It was Carl Sagan who first suggested that nucleotides may have initially served as UV protectors [222]. He argued that the 240–300 nm window was transparent for potentially damaging solar UV radiation before the oxygenation of the atmosphere and appearance of the ozone layer.

Earlier, in 1957, Sagan proposed that differential survival under the condition of a potentially hazardous high-energy flux, such as UV light, may have promoted the selective accumulation of more complex, but more resistant molecules [223]. Experimental evidence for such a mechanism was provided by Powner and colleagues, who demonstrated the UV selection of natural β-2′,3′-cyclic pyrimidine ribonucleotides (see Figure 1D) at the expense of other structurally similar compounds, see Sections S2.6–S2.7 and [126] for details. The authors concluded that “there must be some (photo)protective mechanism functioning with natural nucleotides but not with other pyrimidine nucleosides and nucleotides” [126]. This photoprotective mechanism not only prevented the destruction of natural 2′,3′-cyclic pyrimidine nucleotides, but also preserved the additional high-energy bond (indicated by the red arrow in Figure 1D) despite its susceptibility to spontaneous hydrolysis. The persistence of this bond after three days of UV irradiation suggests that the energy of the UV light specifically restored this additional high-energy bond in the 2′,3′-cyclic pyrimidine nucleotides after its eventual unavoidable breaks. This seminal experiment documents the UV selection of natural nucleotides in their high-energy, polymerization-prone state.

Such a UV selection may have paved the way for the RNA-like oligomers as more complex but more photostable products, in accordance with Sagan’s insight of the “differential survival of polymerized molecules over unpolymerized molecules” [223]. The RNA oligomers are even more UV resistant when they fold into double-helical loops formed by complementary nitrogen bases; for details, see Sections S1.1 and S2.7, and [211,212,213,215,224,225]. Hence, the accumulation of complex RNA-like molecules may have been driven by high-energy radiation [20,21].

#### 1.3.4. Chemistry Conservation Throughout Biological Evolution

Table 1 shows the difference between the concentration of inorganic constituents in the seawater, water of the ancient anoxic ocean, cellular cytoplasm, and extracellular media represented by the blood plasma, as compiled from [41,226,227,228,229,230,231,232,233,234]. Living cells contain much more phosphate (PO_4_^3−^), potassium, magnesium, and zinc than the media they reside in. In contrast, the intracellular concentrations of Na^+^ and Ca^+^ ions are usually much lower than in the environment. These characteristics are common to archaea, bacteria, and eukaryotes that separated billions of years ago (see Section S1.4).

As early as 1904, Archibald Macallum argued that intracellular inorganic chemistry reflects the (geo)chemistry of the habitats of the first cellular unlikely to have tight, impermeable membranes. He noted that these organisms must have been in equilibrium with the environment as it concerned inorganic ions and small molecules, see Section S2.8 and [40,235]. Consequently, modern cells are still filled with a medium similar to that inhabited by their common ancestors.

This phenomenon of chemistry conservation is very important because it helps to reconstruct the habitats of the first organisms even in the absence of any geological record [16,23,40,236].

While reconstructing the environment of the first cells, we have earlier turned to inorganic cofactors of ubiquitous proteins common to all free-living cellular organisms [23]. Their “universal” genes set, as inferred by Koonin [237,238] and listed in Table 2, must have been present in the Last Universal Cellular Ancestor (LUCA) by definition, see Section S1.4. The 87 proteins of Table 2 make up only a small fraction of genes attributable to LUCA the total number of which is estimated to be between 400 and 1000 [239,240,241,242,243]. However, the „complete” gene sets of LUCA, as reconstructed by different research groups, vary widely [244,245], so we have taken, as LUCA’s representatives, the 87 ubiquitous proteins whose presence in LUCA is not usually questioned.

The ubiquity of these genes suggests that their products are essential for cellular organisms. To test this assumption, we compared the ubiquitous set with the genome of the artificial minimal cell [246,247] by assigning both sets to Clusters of Orthologous Groups of proteins (GOGs) in the latest release of the COGs database [248], as described in Methods. We wanted to assess to what extent these ubiquitous proteins are represented in this experimentally determined minimal set. Almost all but three universal COGs were identified in the minimal genome, which indicates their indispensability. The COGs missing from the syn3 minimal bacterial genome are marked with a hash # in Table 2.

We have checked the functional dependence of ubiquitous proteins on inorganic ions and the presence of inorganic ions in their available structures, see Table 2 by assigning the sequences of the latter to the COG database [248] using the last set of profile HMMs for COGcollator [249], as described in Methods. As compared to our earlier analysis [23], Table 2 provides additional data on (i) the number of available structures for each ubiquitous protein and (ii) the number of different metal ions found in these structures, see Methods and Supplementary File S2 for details. This information is very important in identifying physiological cofactors of each ubiquitous protein because metal cofactors can be lost during protein purification or replaced by non-physiological inorganic ions from the medium.

**Table 2:** Inorganic constituents of ubiquitous proteins * (the complete Excel version of the Table with additional information is provided as Supplementary File S2).

COG	Funct. Category	COG Name	Functionally Relevant Inorganic Anions	Functional Dependence on Me^+^	Functional Dependence on Me^2+^	Me^2+^ Cations in at Least Some Structures	Number of Protein Chains in the PDB
COG0636	C	FoF1-type ATP synthase, membrane subunit c/Archaeal/vacuolar-type H+-ATPase, subunit K	-	-	-	Mn^2+^ (1)	3469
COG0112	E	Glycine/serine hydroxymethyltransferase	-	-	Mg^2+^/Ca^2+^	Mg^2+^ (2), Ca^2+^ (1)	385
COG0125	F	Thymidylate kinase	-	-	Mg^2+^	Mg^2+^ (59), Ca^2+^ (8)	230
COG0528	F	Uridylate kinase	-	-	Mg^2+^	Mg^2+^ (33), Mn^2+^ (1)	221
COG1109	G	Phosphomannomutase	-	-	Mg^2+^	Zn^2+^ (20), Mg^2+^ (24), Ca^2+^ (7)	80
COG0149	G	Triosephosphate isomerase	-	-	-	Mg^2+^ (5), Ca^2+^ (7)	622
COG0561	H	Hydroxymethylpyrimidine pyrophosphatase and other HAD family phosphatases	PPi	-	Mg^2+^	Mg^2+^ (56), Ca^2+^ (8)	108
COG0575 /COG4589	I	CDP-diglyceride/archaeol synthetase	PPi	K^+^	Mg^2+^	Mg^2+^ (3)	5
COG2890	J	Methylase of polypeptide chain release factors	-	-	Mg^2+^	Mg^2+^ (4), Ca^2+^ (14), Fe^2+/3+^ (6)	47
COG0024	J	Methionine aminopeptidase	-	-	Co^2+^/Ni^2+^/Mn^2+^/Fe^2+^/Zn^2+^	Zn^2+^ (3), Mg^2+^ (4), Mn^2+^ (64), Ca^2+^ (1), Fe^2+/3+^(5)	240
COG0242	J	Peptide deformylase	formate	-	Zn^2+^/Mn^2+^/Ni^2+^/Fe^2+^	Zn^2+^ (101), Mg^2+^ (3), Fe^2+/3+^ (13)	319
COG0533	J	tRNA A37 threonylcarbamoyltransferase TsaD	-	-	Zn^2+^/Mg^2+^/Fe^2+^	Zn^2+^ (7), Mg^2+^ (7), Ca^2+^ (1), Fe^2+/3+^ (7)	37
COG0101	J	tRNA U38,U39,U40 pseudouridine synthase TruA	-	K^+^	-	Mg^2+^ (1)	38
COG0073	J	tRNA-binding EMAP/Myf domain	n/e	n/e	n/e	-	58
COG0013	J	Alanyl-tRNA synthetase	PPi	K^+^	Mg^2+^, Zn^2+^	Zn^2+^ (14), Mg^2+^ (6)	68
COG0018	J	Arginyl-tRNA synthetase	PPi	K^+^	Mg^2+^	Mg^2+^ (1)	28
COG0124	J	Histidyl-tRNA synthetase	PPi	K^+^	Mg^2+^	Mg^2+^ (1)	85
COG0060	J	Isoleucyl-tRNA synthetase	PPi	K^+^	Mg^2+^, Zn^2+^	Zn^2+^ (19)	31
COG0495	J	Leucyl-tRNA synthetase	PPi	K^+^	Mg^2+^, Zn^2+^	Zn^2+^ (49), Mg^2+^ (36), Ca^2+^ (1)	126
COG0143	J	Methionyl-tRNA synthetase	PPi	K^+^	Mg^2+^, Zn^2+^	Zn^2+^ (31), Mg^2+^ (4)	145
COG0016	J	Phenylalanyl-tRNA synthetase alpha subunit	PPi	K^+^	Mg^2+^, Zn^2+^	Zn^2+^ (2), Mg^2+^ (12), Mn^2+^ (2)	68
COG0072	J	Phenylalanyl-tRNA synthetase beta subunit	PPi	K^+^	Mg^2+^, Zn^2+^	Mg^2+^ (66), Mn^2+^ (1)	161
COG0442	J	Prolyl-tRNA synthetase	PPi	-	Mg^2+^, Zn^2+^	Zn^2+^ (40), Mg^2+^ (32), Mn^2+^ (2), Ca^2+^ (15)	193
COG0172	J	Seryl-tRNA synthetase	PPi	K^+^	Mg^2+^, Zn^2+^	Zn^2+^ (18), Mg^2+^ (10), Ca^2+^ (6)	119
COG0441	J	Threonyl-tRNA synthetase	PPi	K^+^, Rb^+^	Mg^2+^, Zn^2+^	Zn^2+^ (53), Mg^2+^ (2), Ca^2+^ (1)	206
COG0180	J	Tryptophanyl-tRNA synthetase	PPi	K^+^	Mg^2+^, Zn^2+^	Mg^2+^ (18), Mn^2+^ (1), Ca^2+^ (5)	284
COG0162	J	Tyrosyl-tRNA synthetase	PPi	K^+^	Mg^2+^	Mg^2+^ (2)	67
COG0525	J	Valyl-tRNA synthetase	PPi	-	Mg^2+^, Zn^2+^	Zn^2+^ (1)	9
COG0081	J	Ribosomal protein L1	The ribosome, as a whole, requires high levels of Mg^2+^ and K^+^ ions, as well as sufficient levels of Zn^2+^ ions; see the table footer for the references			Mg^2+^ (5)	522
COG0244	J	Ribosomal protein L10				-	649
COG0080	J	Ribosomal protein L11				Mg^2+^ (2),	871
COG0102	J	Ribosomal protein L13				Zn^2+^ (2), Mg^2+^ (6),	1979
COG0093	J	Ribosomal protein L14				Zn^2+^ (3), Mg^2+^ (65),	1944
COG0200	J	Ribosomal protein L15				Mg^2+^ (16), Mn^2+^ (1)	1938
COG0197	J	Ribosomal protein L16/L10AE				Zn^2+^ (1), Mg^2+^ (1), Mn^2+^ (1)	1807
COG0256	J	Ribosomal protein L18				-	1866
COG0090	J	Ribosomal protein L2				Zn^2+^ (1), Mg^2+^ (106), Mn^2+^ (3)	1940
COG0091	J	Ribosomal protein L22				Mg^2+^ (1)	1980
COG0198	J	Ribosomal protein L24				Mg^2+^ (64), Mn^2+^ (2)	1955
COG0255	J	Ribosomal protein L29				-	1840
COG0087	J	Ribosomal protein L3				Mg^2+^ (78), Mn^2+^ (1)	1983
COG0088	J	Ribosomal protein L4				Zn^2+^ (3), Mg^2+^ (13), Mn^2+^ (1)	1991
COG0094	J	Ribosomal protein L5				Zn^2+^ (3), Mg^2+^ (3)	1789
COG0097	J	Ribosomal protein L6P/L9E				Zn^2+^ (3)	1827
COG0051	J	Ribosomal protein S10				Mg^2+^ (15)	1783
COG0100	J	Ribosomal protein S11				Zn^2+^ (5), Mg^2+^ (18)	1905
COG0048	J	Ribosomal protein S12				Zn^2+^ (2), Mg^2+^ (21)	1930
COG0099	J	Ribosomal protein S13				Zn^2+^ (1), Mg^2+^ (14)	1817
COG0199	J	Ribosomal protein S14				Zn^2+^ (126), Mg^2+^ (45)	1362
COG0184	J	Ribosomal protein S15P/S13E				Zn^2+^ (1), Mg^2+^ (9)	1879
COG0186	J	Ribosomal protein S17				Zn^2+^ (7), Mg^2+^ (16)	1917
COG0185	J	Ribosomal protein S19				Mg^2+^ (8)	1795
COG0052	J	Ribosomal protein S2				Zn^2+^ (17), Mg^2+^ (75)	1829
COG0092	J	Ribosomal protein S3				Mg^2+^ (23)	1764
COG0098	J	Ribosomal protein S5				Zn^2+^ (7), Mg^2+^ (98)	1885
COG0049	J	Ribosomal protein S7				Zn^2+^ (9), Mg^2+^ (12)	1899
COG0096	J	Ribosomal protein S8				Zn^2+^ (3), Mg^2+^ (29)	1842
COG0103	J	Ribosomal protein S9				Mg^2+^ (8)	1895
COG0012	J	Ribosome-binding ATPase YchF, GTP1/OBG family	Pi	K^+^	Mg^2+^	Mg^2+^ (3)	15
COG0480	J	Translation elongation factor EF-G, a GTPase	Pi	K^+^	Mg^2+^	Mg^2+^ (41)	302
COG0050	J	Translation elongation factor EF-Tu, a GTPase	Pi	K^+^	Mg^2+^	Zn^2+^ (1), Mg^2+^ (58)	175
COG0231	J	Translation elongation factor P (EF-P)/translation initiation factor 5A (eIF-5A)		K^+^		-	78
COG0361	J	Translation initiation factor IF-1	Pi	K^+^	Mg^2+^	Zn^2+^ (1), Mg^2+^ (7)	78
COG0532	J	Translation initiation factor IF-2, a GTPase	Pi	K^+^	Mg^2+^	Mg^2+^ (20)	86
COG0202	K	DNA-directed RNA polymerase, alpha subunit/40 kD subunit	PPi	K^+^	Mg^2+^, Zn^2+^	Zn^2+^ (268), Mg^2+^ (54)	1768
COG0085	K	DNA-directed RNA polymerase, beta subunit/140 kD subunit	PPi	K^+^	Mg^2+^, Zn^2+^	Zn^2+^ (363), Mg^2+^ (23)	1132
COG0086	K	DNA-directed RNA polymerase, beta’ subunit/160 kD subunit	PPi	K^+^	Mg^2+^, Zn^2+^	Zn^2+^ (835), Mg^2+^ (710), Mn^2+^ (8), Fe^2+^/^3+^ (1)	1171
COG0195	K	Transcription antitermination factor NusA, contains S1 and KH domains	-	-	-	Mg^2+^ (1)	49
COG0250	K	Transcription termination/antitermination protein NusG	-	-	-	Fe^2+^/^3+^ (1)	136
COG0258	L	5′-3′ exonuclease Xni/ExoIX (flap endonuclease)	-	K^+^	Mg^2+^, Mn^2+^	Zn^2+^ (2), Mg^2+^ (18), Mn^2+^(20), Ca^2+^ (8)	104
COG0592	L	DNA polymerase III sliding clamp (beta) subunit, PCNA homolog	-	K^+^	Mg^2+^	Mg^2+^ (3), Ca^2+^ (47)	368
COG2812	L	DNA polymerase III, gamma/tau subunits	-	K^+^	Mg^2+^, Mn^2+^	Zn^2+^ (54), Mg^2+^ (36)	92
COG0358	L	DNA primase (bacterial type)	PPi		Mg^2+^, Mn^2+^	-	29
COG0550	L	DNA topoisomerase IA	-	K^+^	Mg^2+^, Zn^2+^	Zn^2+^ (4), Mg^2+^ (5), Ca^2+^ (1)	52
COG0468 #	L	RecA/RadA recombinase	Pi	K^+^	Mg^2+^, Mn^2+^	Mg^2+^ (59), Mn^2+^ (1), Ca^2+^ (27)	514
COG0513	L	Superfamily II DNA and RNA helicase	Pi	-	Mg^2+^, Mn^2+^	Zn^2+^ (4), Mg^2+^ (65), Ca^2+^ (2)	421
COG0206	D	Cell division GTPase FtsZ	Pi	-	Mg^2+^	Mg^2+^ (13), Mn^2+^ (2), Ca^2+^ (17)	202
COG1136	M	ABC-type lipoprotein export system, ATPase component	Pi	-	Mg^2+^	Mg^2+^ (9), Mn^2+^ (1)	73
COG0084 #	N	3′->5′ ssDNA/RNA exonuclease TatD	-	-	Mg^2+^	Zn^2+^ (7), Mg^2+^ (2), Mn^2+^ (1)	20
COG1215	N	Glycosyltransferase, catalytic subunit of cellulose synthase	-	-	Mg^2+^	Mg^2+^ (22), Mn^2+^ (23)	138
COG3118	O	Chaperedoxin CnoX, contains thioredoxin-like and TPR-like domains, YbbN/TrxSC family	-	-	-	Zn^2+^ (22), Mg^2+^ (5), Ca^2+^ (4), Fe^2+^/^3+^ (2)	668
COG0459 #	O	Chaperonin GroEL (HSP60 family)	Pi	K^+^	Mg^2+^, Mn^2+^	Mg^2+^ (141), Ca^2+^ (9)	3011
COG0492	O	Thioredoxin reductase	-	-	-	Mg^2+^ (11), Ca^2+^(4), Fe^2+^/3^+^ (1)	198
COG0201	U	Preprotein translocase subunit SecY	n/e	-	-	Zn^2+^ (1)	92
COG0541	U	Signal recognition particle GTPase	Pi	-	Mg^2+^	Mg^2+^ (23), Mn^2+^ (1), Ca^2+^ (4)	123
COG0552	U	Signal recognition particle GTPase FtsY	Pi	-	Mg^2+^	Mg^2+^ (12)	87

* The table contains information about inorganic constituents and cofactors of 87 orthologous groups of proteins found in all free-living organisms [237]. The entries in the table are colored by functional categories given according to the COG database [248]: C—Energy production and conversion, E—Amino acid transport and metabolism, F—Nucleotide transport and metabolism, G—Carbohydrate transport and metabolism, H—Coenzyme transport and metabolism, I—Lipid transport and metabolism, J—Translation, ribosomal structure, and biogenesis, K—Transcription, L—Replication, recombination, and repair, D—Cell cycle control, cell division, and chromosome partitioning, M—Cell wall/membrane/envelope biogenesis, N—Cell motility, O—Posttranslational modification, protein turnover, and chaperones, and U—Intracellular trafficking, secretion, and vesicular transport. Other abbreviations: PPi—pyrophosphate and Pi—phosphate. The information on the functional dependence of the proteins on metal ions was mostly inferred from the BRENDA database [250], see Methods for details. Notably, the ribosome, as a whole, requires high levels of Mg^2+^ and K^+^ ions, as well as sufficient levels of Zn^2+^ ions [251,252,253,254]. In the *E. coli* ribosome resolved to 1.55 Å, 403 Mg^2+^ and 231 K^+^ ions were identified as bound [255]. Preparations of whole ribosomes may contain up to 10 bound Zn^2+^ ions [256].

Table 2 reiterates our earlier conclusion that many proteins and functional systems, traceable back to the LUCA and beyond, require K^+^ ions for functioning, while none of these ancestral proteins specifically requires Na^+^ [23]. This conclusion is consistent with potassium being the only alkali metal necessary for life [257]. The K^+^ ions are particularly important for protein biosynthesis on ribosomes [253,254]; they serve as catalytic cofactors (i) in the peptidyl transferase center, where amino acids are linked by a peptide bond [258], (ii) in the decoding center, where tRNA recognizes the codon of mRNA [259], and (iii) in numerous proteins that assist translation, including, most likely, ubiquitous translation factors, see Section S1.2 and [37,39]. Usually, the K^+^-binding sites of biopolymers can bind smaller Na^+^ ions even more tightly; however, these Na^+^ ions cannot functionally replace K^+^ ions in most cases [37]. To prevent such non-physiological Na^+^ binding, all active cells maintain a [K^+^] level of ~100 mM and a [K^+^]/[Na^+^] ratio of about 10.0 in the cytoplasm, see Table 1, Section S2.8, and [23,35,38,260,261].

In addition, many ubiquitous proteins deal with ATP, GTP, or pyrophosphate, which implies an abundance of phosphorous compounds in the habitats of the first cells; for details, see Section S2.11. The named enzymes usually use Mg^2+^ ions as catalytic cofactors, see Table 2 and Section S2.9. Magnesium, which is the only alkaline earth metal essential for life [257], makes about 20% of the Earth’s crust; its high level in environment is due to the solubility of its common salts, so that the recruitment of Mg^2+^ ions as cofactors by ancient enzymes is not particularly surprising, see also Section S2.9 and [262].

In contrast, the prevalence of geologically rather rare Zn^2+^ ions in the evolutionarily oldest proteins was rather surprising when first revealed [21,22]. The Zn^2+^ ions serve either as catalytic cofactors or as structural elements that stabilize the protein folds by linking several amino acid residues [263]. We have suggested that this very early recruitment of Zn^2+^ ions as mere structural elements(!) indicated the abundance of Zn^2+^ ions in the habitats of the first organisms, see Section S2.10 and [21,22,23,24,33].

Thus, in the hope of identifying the habitats of the first cells, we have searched for environments with a high content of K^+^ ions and their predominance over sodium ions ([K^+^]/[Na^+^] ~10). We have chosen the high [K^+^]/[Na^+^] ratio as a key search criterion because it cannot be distorted by an eventual evaporation of water. In addition, the environments sought had to contain high levels of phosphorus compounds and Zn^2+^.

The selected criteria were met only by the condensate of geothermal vapor discharging at geothermal fields, where the subsurface vapor-dominated zones feed numerous hot vents, see Section S2.8 and [23,24] for further details.

In general, the geothermal vapor has a quite specific chemical composition [264,265], as documented in Table 3. It accumulates those substances that have a particular affinity for the gas phase. Because of its higher affinity for vapor, K^+^ ions dominate over Na^+^ ions in the vapor condensate [23].

As it also follows from Table 3, geothermal vapor is particularly enriched in highly volatile Zn^2+^ ions. While the Zn/Fe ratio in the Earth’s crust is about 0.001 [17], it averages about 0.05 in geothermal vapor.

Based on these data, we have proposed that the first cells may have formed in anoxic pools of cool geothermal condensate, which must have been pH neutral in the absence of atmospheric oxygen. These pools may have contained relatively high levels of K^+^, Zn^2+^, and abiotically formed organic molecules, see Table 3, Sections S2.8–S2.11 and [23,24] for details.

Notably, the compounds with a specific affinity for geothermal vapor are otherwise considered to be either the building blocks for abiotic syntheses of the first biomolecules (hydrogen sulfide (H_2_S), NH_3_, and simple organics) or specific catalysts of these syntheses, such as borate, see Section 1.3.1 and Section S2.2, and [116,266]. Therefore, we hypothesized also that anoxic geothermal fields may have served as the cradles of life itself, with geothermal pools sheltering and nourishing the first, pre-cellular life forms until they evolved into the first cells [23,24]. This is the most parsimonious scenario: otherwise, one would have to envision separate mechanisms for the transfer of the first pre-cellular organisms from elsewhere to geothermal fields.

Our work [23,24] had prompted geologists to look for vestiges of anoxic geothermal fields. Van Kranendonk and his colleagues have discovered them in the 3.48 Ga old Dresser Formation of the Pilbara Craton, Western Australia [267,268,269,270], i.e., in the same location where the oldest evidence of life on Earth had been found earlier [271,272,273,274,275]. The analysis of the Dresser Formation deposits revealed the remnants of hot vents surrounded by sinter terracettes, see Section S2.8 [267,269,270]. The stromatolites, made by microbial communities dwelling in basins of these geothermal fields 3.48 Ga ago, are characterized by alternating layers of zinc and nickel [268,270]. These groundbreaking findings indicate that the anoxic geothermal fields existed as early as 3.48 Ga ago and, most likely, were inhabited.

Still, so far, we could not convincingly explain why the ubiquitous proteins do not use Fe^2+^ ions as cofactors (Table 2). In fact, only in the case of the tRNA A37 threonylcarbamoyltransferase TsaD, an Fe^2+^ ion was found to be a catalytic cofactor in the thermophilic organisms *Pyrococcus abyssi* [276]. However, further studies have shown that the orthologs of this enzyme in other organisms mostly use Zn^2+^ ions [277].

The revealed non-use of iron is striking as it makes up 5% of the Earth’s crust. Present-day geothermal vapor condensates, although heavily enriched in Zn^2+^, are always dominated by Fe^2+^ ions, see Table 3. Also, iron is present in the 3.48 Ga old deposits of the Dresser formation [267,278]. The non-use of Fe^2+^ is all the more paradoxical since Fe^2+^ ions can, in principle, replace Zn^2+^ ions as catalytic cofactors, as happens in the tRNA A37 threonylcarbamoyltransferase TsaD [277] and as has been shown for some other proteins, see [279,280] for reviews.

And yet, the avoidance of Fe^2+^ ions by the evolutionarily oldest proteins has been noticed by others as well [281,282]. Specifically, David and Alm have “mapped the evolutionary history of 3983 gene families across the three domains of life onto a geological timeline” [281]. One of their findings was that the mass appearance of Fe^2+^-containing enzymes occurred about 100–200 million years after the emergence of the very first enzymes. To these first enzymes, David and Alm assigned the ubiquitous nucleotide- and phosphate-processing enzymes, many of which are zinc-dependent, see Table 2.

A plausible solution to this Zn/Fe paradox is provided by our evolutionary reconstruction in Section 3.

### 1.4. Formation of the Moon and Its Geochemistry

The geochemistry of lunar formation is very important for our evolutionary reconstruction. As this event is usually beyond the interests of origin of life researchers, we review the available data in some detail below. Our task is facilitated by a recent comprehensive review co-authored by almost all of the contributors to the field [42].

A certain clarity about the origin of the Moon came after six American Apollo missions and four Soviet Luna stations returned to Earth bringing together approximately 400 kg of lunar rocks. Some of these rocks are up to 4.46 Ga old, thus defining the time of the Moon’s formation as 50–100 Ma after the formation of Earth [42,283]. Because of these old minerals, the early geology of the Moon is better understood than the geology of the Hadean Earth.

The study of lunar samples has revealed several peculiarities. First, the isotopic compositions of such common chemical elements as oxygen, silica (Si), and iron in the lunar samples were found to be similar to those in the Earth’s mantle [42]. Since the isotopic signatures of Earth, Mars, and meteorites are clearly different, the discovered isotopic similarity implies some kind of intimate connection between the Earth’s mantle and the Moon [42].

Second, the relative content of chemical elements in the lunar samples was found to depend essentially on their volatility. In planetology, the volatility of an element is defined through the temperature at which 50% of an element would have condensed from a gas of solar composition at a total pressure of 10^−4^ bar (50% condensation temperature, T_C_). Elements with T_C_ > 960 °C are considered as refractory elements (REs), those with 370 °C < T_C_ < 960 °C as moderately volatile elements (MVEs), and elements with T_C_ < 370 °C as volatile elements (VEs), see e.g., [284].

The refractory elements are present in the lunar rocks in exactly the same relative proportions as in the Earth’s mantle, see e.g., [285,286]. At the same time, the Moon is dramatically depleted in most MVEs, with the degree of depletion usually correlating with their T_C_ values. In terms of biologically relevant metals, the Moon is approx. ten-fold depleted in sodium (T_C_ = 762 °C [287]), copper (T_C_ = 761 °C [287]), and potassium (T_C_ = 720 °C [287]). Furthermore, the Moon is approx. hundred-fold depleted in zinc (T_C_ = 431 °C [287]) compared to the Earth [42,286]. The degree of depletion in VE is even larger; they are only present in trace amounts in lunar rocks [286,288].

These results significantly narrow the range of reasonable scenarios for the formation of the Moon. Any scenario would have to explain why lunar rocks contain iron, oxygen or silica in the same relative amounts and with the same isotopic composition as the Earth’s mantle, but a hundred times less Zn, with its isotopic signature biased towards heavier isotopes [286,289].

Currently, the isotopic similarity between lunar rocks and Earth’s mantle, as well as some other peculiarities of the Moon, are explained by a giant impact model [42,285,290,291]. It suggests that the Moon was formed by the collision of a celestial body with the already differentiated Earth. This collision resulted in the release of large amounts of energy that caused a mixing of the impactor with the Earth’s mantle, followed by the partial vaporization of the mixed melt. The silicium-dominated vapor formed a protolunar disk (PLD) that apparently extended past the Roche limit (9500 km), beyond which a celestial body can remain stable without being torn apart by Earth’s gravity [42,292]. The giant impact explains the rapid early rotation of the Earth, the traces of a magma ocean on the Moon, and the small (or missing) iron core of the Moon [42]. Within this concept, the similarity of the isotopic composition of the Earth’s mantle and the Moon is explained by equilibration within the hot PLD [293].

The depletion of the Moon in K, Na, and Zn was initially attributed to their hydrodynamic escape from the edge of PLD, see, e.g., [286]. However, the extent of hydrodynamic escape depends strongly on the atomic mass; the lightest elements, such as hydrogen (with an atomic mass of 1 Dalton (Da)) or helium (4 Da) escape much more easily. Thus, it has remained unclear how 98–99% of the “lunar” zinc, which is heavy (65 Da), could have escaped hydrodynamically, while lighter elements, such as Li (7 Da) or Si (14 Da), were retained by the Moon.

At least three models have been proposed to specifically explain the dramatic depletion of the Moon in the MVEs [292,294,295]. These models suggest that the Moon eventually lost contact with the gaseous content of the still hot PLD, so that this residual vapor condensed on Earth. In the most elaborated model, Lock and his colleagues proposed that the enormous heating by the impact (estimated at 10^30^–10^31^ erg) transformed the Earth into a transiently “expanded” state, which the authors named “synestia” [42,292,296]. In this state, the entire Earth swelled into a giant, very hot toroidal disc. In the center was the solid Earth covered by its partially melted mantle and surrounded by a dense and very hot “cloud” of silicate vapor. As the synestia cooled, the atoms beyond the Roche limit coalesced first into droplets, then into moonlets, and finally into a single moon. At the same time, those droplets that coalesced inside the Roche limit fall to Earth. Owing to cooling and condensation, the toroid gradually contracted, eventually leaving the nascent Moon outside the synestia. By the time the nascent Moon “broke free”, the REs had already coalesced and precipitated onto the Moon and Earth, respectively. In contrast, the MVEs, still in the gaseous state, stayed with the shrinking synestia, and thus lost the opportunity to fall out onto the Moon. According to Lock and his colleagues, “as the synestia cools and contracts within the lunar orbit, the remaining MVEs are destined to be incorporated into the Bulk Silicate Earth” [292].

A virtue of the synestia scenario is that it also explains the recently found similarity of isotopic signatures of tungsten (W) in the terrestrial and lunar rocks [297,298,299]. The T_C_ value of tungsten is very high (1247 °C at 10^−4^ bar [287] and 5930 °C at 1 bar, respectively), which implies that the initial post-impact thermal equilibration proceeded at much higher temperatures than previously thought. Unlike the two other hypothetical mechanisms [294,295], the synestia model implies very high initial temperatures above ~10,000 °C, which must have ensured the vaporization and equilibration of tungsten even at high initial pressure within the PLD [292]. Also, the synestia model quantitatively explains why the potassium isotope data for the Moon differ from those for the Earth and chondritic meteorites [300].

A fairly detailed description of what would have happened on Earth after the Moon-forming event was earlier provided by Zahnle and his colleagues [301]. They noted that after the Earth’s surface temperature dropped below the solidification temperature of silicate rock, a hardened (proto)crust must have formed over the molten mantle. Thus, the last PLD residuals must have precipitated onto the already solidified protocrust. The authors wrote: “The post-silicate atmosphere may also have contained moderately volatile elements such as cadmium. The most abundant of these are S, Na, Zn, Cl, and K. These may not fully condense until after the magma ocean freezes. We might therefore expect the first crust to be enriched in these elements. Mass balance would imply an early chalcophilic crust a few km thick....” [301].

## 2. Methods

### 2.1. Comparison of the Ubiquitous Protein Gene Set with the Genome of the Artificial Minimal Cell

We took 445 proteins (438 protein-coding genes in the syn3.0 version of the minimal genome [246] plus seven proteins recently identified as required for normal cell morphology and division [247]) and assigned them to Clusters of Orthologous Groups of proteins (GOGs) in the latest release of the COGs database [248] with a 1 × 10^−5^ e-value threshold and using a set of profile HMMs available on the web server http://boabio.belozersky.msu.ru/en/DomainAnalyser (accessed on 1 September 2024), yielding a total of 365 COGs, which were used for comparison.

### 2.2. Determination of Ubiquitous Proteins’ Dependence on Inorganic Ions

We took protein sequences longer than 50 amino acids from the Protein Date Bank (PDB) database ([302], https://www.rcsb.org/, checked on 13 July 2024) and attributed them to the COG database [248] using the last set of profile HMMs for COGcollator ([249], available at http://boabio.belozersky.msu.ru/tools (accessed on 1 September 2024), and the hmmscan program (http://hmmer.org/, accessed on 1 September 2024). If two profile HMMs found overlapping hits and the overlap was longer than 5% of the longest of these two hits, we filtered out the weakest hit. To avoid confusion, we further used only proteins that were attributed to a single COG with an e-value less than 1 × 10^−10^ according to this procedure. We selected only protein chains which were attributed to the set of 87 universal COGs (see Table 2). The CDP-diglyceride synthetase occurs in two COGs (COG0575 and COG4589) and therefore both are included in Table 2. A total of 71014 protein chains belonging to 7767 PDB structures were sampled. The information on the functional dependence of the proteins on metal ions was mostly derived from the BRENDA (Braunschweig Enzyme Database) [250]. If the information was missing from the BRENDA database, we manually checked the articles in which the respective structures were described.

### 2.3. Calculation of the Amount of Biologically Relevant Moderately Volatile Elements Deposited on the Earth from the PLD

In calculating the MVE fallout we have proceeded as follows. Although the mass of the PLD has been estimated as 1.3–3.0 lunar masses [42], for simplicity, we assumed that the PLD had two lunar masses, 1.4 × 10^23^ kg. We then calculated the content of each MVE of interest in 1.4 × 10^23^ kg of primitive mantle (with the mantle composition taken from [303]). Next, we calculated the amount of each MVE in the Moon, using the references indicated in Table 4 as sources. The amount of particular MVE deposited from the PDL onto the Earth’s surface was determined as the difference between the two values with the respective numbers shown in bold in Table 4.

### 2.4. Thermodynamic Modeling of the Stepwise Cooling PLD

The thermodynamic model was calculated for the composition of a dry Bulk Silicate Earth (BSE) [303]. We used the HCh energy minimization software package, version 4.6. [304,305], which utilizes the thermodynamic potential UNITHERM database of Gibbs free energy values for minerals, liquids, and gases. The calculations were performed using successive isobaric reactors with decreasing temperature in the range of 2500–100 °C (incremented by 100 °C) and at pressures of 1–100 bar (incremented by 5 bar for pressures 0–55 bar and by 10 bar for pressures 55–100 bar). The calculations were performed in a partial condensation mode, where solid and liquid phases were removed from the system at each calculation step to simulate their precipitation. The calculated values for other elements are provided in Supplementary File S3.

## 3. Results: Evolutionary Reconstruction of Abiogenesis on Hadean Earth

### 3.1. Earth Before and After the Formation of the Moon

#### 3.1.1. Differentiation of the Earth

The Earth formed 4.53–4.56 Ga ago [3,4,5] mainly from enstatite chondrites, whose chemical and isotopic composition is closest to that of the Earth, with a minor contribution from carbonaceous chondrites, which are thought to have been important in supplying the Earth with volatiles and, specifically, carbon and nitrogen [306,307,308,309,310]. Both enstatite and carbonaceous chondrites must have brought water with them, resulting in an Earth water content of 1000–3000 ppm or 5–15 volumes of modern ocean [308,309,311]. This water must have contributed to the gravitational melting of the nascent Earth, allowing the iron core to separate from the predominantly silicate mantle. As a result of this differentiation, the *f*(O_2_) of the mantle must have increased, supposedly from ~IW-3 to ~IW-1.5 [306].

#### 3.1.2. Loss of the Primary Atmosphere upon the Moon-Forming Impact

**Figure 3:**
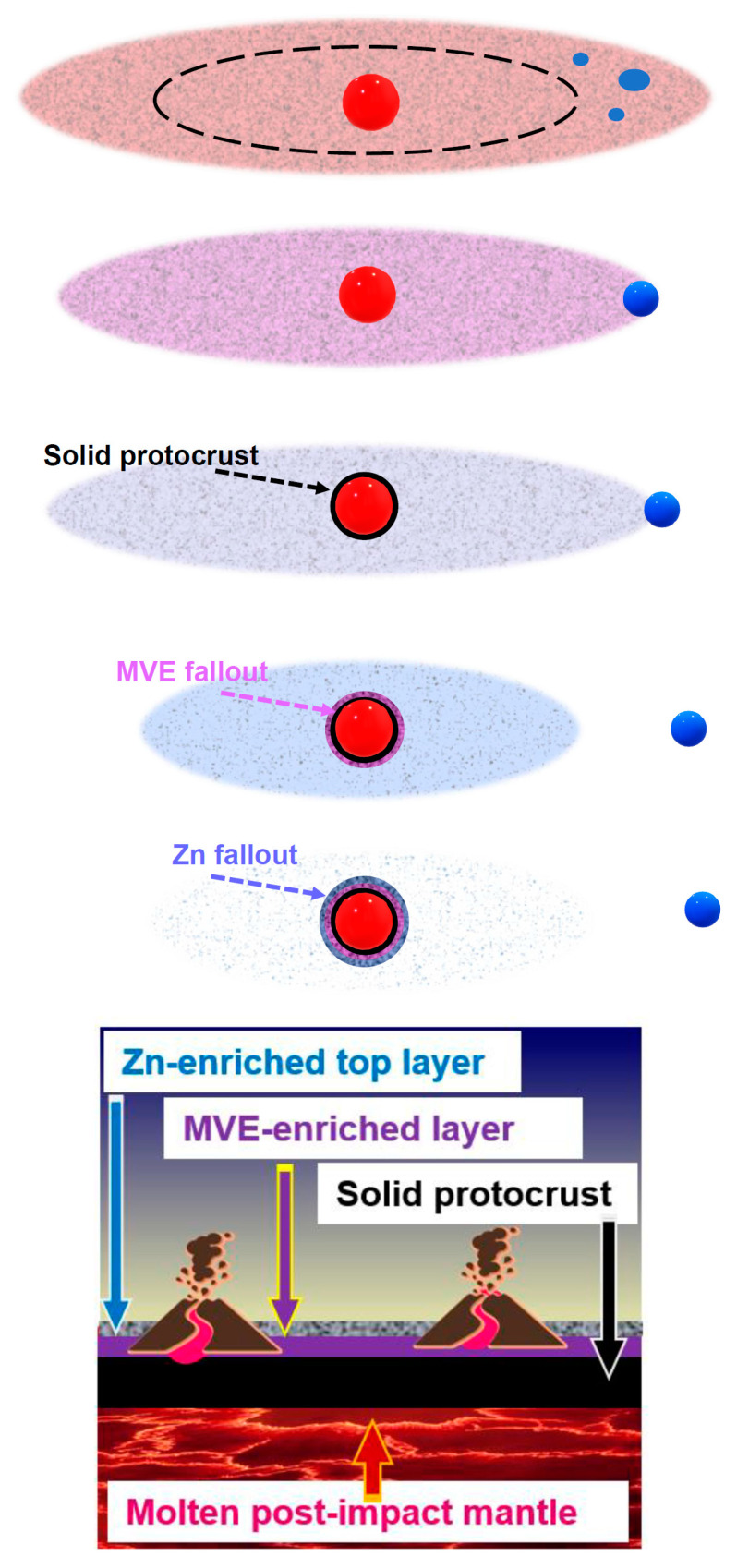
Formation of the Moon (the blue dot), gradual fallout from the PLD, and emergence of volcanoes. For details, see [42,292,301].

The celestial collision that formed the Moon happened 4.51–4.40 Gyr ago [42,283] and must have led to a partial—if not total—loss of any existing atmosphere and hydrosphere of Earth, mostly by shock escape [312,313,314]. The impact must have led to a melting and subsequent partial vaporization of the upper mantle yielding a silica-dominated PLD with an Earth-mantle composition and initial pressure of 10^4^–10^5^ bar at the vapor/liquid phase boundary, see Figure 3, Section 1.4, and [42,292]. Silicate melts can accommodate up to 10% volatiles, depending on the pressure [315,316]. Therefore, the abruptly molten upper mantle—hungry for volatiles because of the high pressure—must have drawn in (ingassed) the remaining atmospheric volatiles upon equilibration with the PLD, see also [317].

#### 3.1.3. Loss of Hydrogen from the PLD and Dry Condensation of the PLD

The initial temperature of PLD, depending on the model, has been estimated as between ~4000 °C and > ~10,000 °C (see Section 1.4 and [42,292]). Water splits into oxygen and hydrogen above ~2250 °C [318]. Consequently, hydrogen must have been lost via hydrodynamic escape from the very hot PLD, see [291].

As the PLD gradually cooled down, its atoms condensed into droplets and mineral crystals that concurrently (i) coalesced into the Moon beyond the Roche limit and (ii) precipitated onto the Earth within this limit (see Figure 3, Section 1.4 and [42,292]).

The loss of hydrogen from the PLD must have precluded the formation of water during the condensation step. The dehydrated state of the PLD is consistent with the trace amounts of water (1–10 ppm) in the bulk of the Moon [286,319,320].

#### 3.1.4. The MVEs Present in the PLD Have Mostly Fallen to Earth

It is currently thought that the PLD had 1.3–3.0 lunar masses [42]. Hence, the amount of material fallen to Earth from the PLD can be estimated at 0.3 to 2.0 lunar masses. It is easy to show that the thickness of the precipitate layer that fell to Earth from the PLD must have been in the range of ~15–80 km.

According to current views, the connection between the PLD and the nascent Moon got lost when most MVEs were still within the hot PLD; these MVEs must have ultimately fallen on Earth, see Figure 3, Section 1.4, and [42,292,301]. Table 4 shows our estimates of how much biologically relevant MVEs, such as sulfur, zinc, potassium, sodium, and copper, must have precipitated from the PLD to Earth.

Table 4 shows also that the degree of the sulfur depletion of the Moon is much lower than that for K, Na, Cu, and Zn (see also [288,321]) despite the low T_C_ of sulfur of 399 °C [287]. This discrepancy indicates the absence of H_2_S, the usually dominant volatile form of sulfur, from the hydrogen-depleted PLD, see also [288,322]. In the absence of H_2_S, the sulfur was likely to condense already at high temperatures (mostly as metal sulfides) and to precipitate onto the Moon before its connection with the PLD was lost. Thus, the marginal sulfur depletion of the Moon may be another sign of the dehydrated state of the PLD.

**Table 4:** Estimated amounts of biologically important MVEs in the post-impact fallout on Earth, see Methods for the calculation routine.

Element, T_C_ (Acc. to [287])	Fraction in Primitive Earth Mantle (According to [303])	Amount Earth, kg	Expected Amount in the PLD of 2 Lunar Masses, kg	Extent of Lunar Depletion, kg	Amount Moon, kg	Amount Fallen on the Earth from the PLD, kg	References
S, 399 °C	0.00025	1.5 × 10^21^	3.6 × 10^19^	1 ÷ 3.4	(0.6 ÷ 1.8)×10^19^	**(1.8 − 3.0) × 10^19^**	[288,321,323]
Zn, 431 °C	0.000053	3.2 × 10^20^	7.6 × 10^18^	50–200	(1.9 ÷ 7.5) × 10^16^	**~7.6 × 10^18^**	[286,288]
K, 720 °C	0.00026	1.6 × 10^21^	3.8 × 10^19^	4–7	(2.5 ÷ 4.5) × 10^18^	**(3.3** **÷ 3.5) × 10^19^**	[288]
Cu, 761 °C	0.000030	1.8 × 10^20^	4.3 × 10^18^	9	4.3 × 10^17^	**~3.9 × 10^18^**	[286,288,323]
Na, 762 °C	0.0026	1.6 × 10^22^	3.8 × 10^20^	4–8	(2.4 ÷ 4.5) × 10^19^	**(3.3** **÷ 3.6) × 10^20^**	[288]

#### 3.1.5. Fallout of Potassium from the PLD

The fallout of the PLD content on Earth must have begun when the Earth was still covered by a magma ocean, so that the precipitate, initially enriched in REs, such as iron, simply mixed with the melt. However, when the Earth’s surface temperature dropped below the solidus point of silica, the first solid protocrust must have formed [301]. Because of the PLD-imposed high pressure [292], this must have happened when the temperature at the vapor/liquid interface dropped below approx. ~1700 °C. Thereafter, the MVE-enriched precipitates fell onto the solid protocrust and could not easily mix with the molten mantle [301]. The MVEs listed in Table 4 alone have a total mass of ~4 × 10^20^ kg, which must have provided an atmospheric pressure of ≥100 bar by the time when the precipitation of MVEs began. This is the lower bound estimate, as the admixture of residually precipitating silicates must have been unavoidable [292].

As follows from Table 4, the fallout must have contained 3.3 ÷ 3.5 × 10^19^ kg of potassium, of which about 0.1%, i.e., ~3.0 × 10^16^ kg was its radioactive isotope ^40^K [50,51]. Thermodynamic calculations for the pressure range from 1 mbar to 50 bar showed that the T_C_ values of different elements increased with pressure but the relative order of the precipitation of different elements did not change [292]. According to the same calculations, K must have condensed after Na [292] and, thus, concentrated in the upper layers of the protocrust. The average content of K^+^ ions in the MVE-rich protocrust of 5–10 km thickness can be estimated, from the data in Table 4, as 1500–3000 ppm, i.e., 7–14 times more than in the primitive upper mantle [303]. If we use the chemistry of cell cytoplasm as a guideline, the ~100 mM K^+^ present in most cells corresponds to 4000 ppm, which is in good agreement with the above estimate.

#### 3.1.6. Deposition of Metallic Zn in the Topmost Layer of the Post-Impact Protocrust

Zinc, with its T_C_ of 431 °C at 10^−4^ bar [287], must have been one of the last MVEs to condense and precipitate. Since the Moon is depleted in zinc by a factor of 50–200 [286,288], essentially all the Zn originally present in the PLD, i.e., up to 10^19^ kg (see Table 4) must have fallen to Earth. The contribution of gaseous Zn alone must have produced an atmospheric pressure of ~2 bar. Along with Zn, the belated residuals of silicates, sodium, and potassium must have precipitated, see Supplementary File S3 and [292]. Since the PLD must have contained as much as ~10^22^ kg of silica, the mass of residual silicates precipitating along with Zn may have exceeded that of Zn, so that the pressure at the Earth’s surface during the Zn fallout can be estimated as ~10 bar.

To assess the state of precipitating PLD components, we have modeled the gradual cooling of the PLD assumed to have the composition of a dry Bulk Silicate Earth (BSE) [303], see Methods and Supplementary File S3 for details.

**Figure 4:**
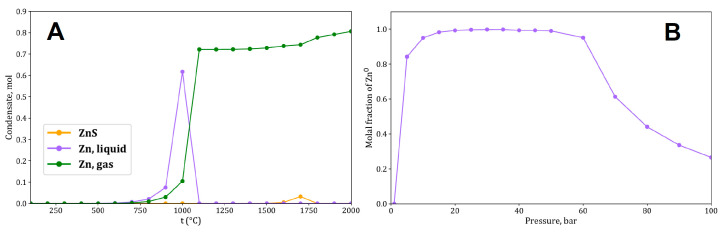
Zinc phases during cooling of 1000 kg of the BSE composition. The calculated values for other elements are provided in the Supplementary File S3. (**A**) Temperature-dependent condensation of zinc phases at 10 bar. (**B**) The molar fraction of metallic zinc in the precipitate as a function of pressure.

As shown in Figure 4A, at the pressure of ~10 bar, the condensation of Zn must have occurred first to ZnS at ~1750 °C (minor fraction) and then to liquid metallic Zn (Zn^0^) around 1000 °C (major fraction). Figure 4B shows that Zn^0^ must have dominated the pressure range of 5–60 bar.

Thus, the thermodynamic calculations indicate a significant precipitation of liquid Zn^0^ at the very end or the PLD fallout, as a kind of zinc rain. Metallic zinc must have been solidified as small crystals within silica minerals; such Zn^0^-containing minerals have been repeatedly reported from different locations [324]. Assuming a thickness of 100–1000 m for the uppermost layer of the PLD precipitate, its Zn content must have been in the range of 0.2–5.0%.

After Zn, metals with even higher volatility, such as Cd, In, and Tl, must have precipitated. We do not consider them further because of their trace amounts and biological irrelevance. This means that Zn was the last of the life-relevant metals to fall on Earth from the PLD.

We propose that the enrichment of the protocrust with zinc and potassium explains their indispensability and importance for life, see Table 2 and [21,22,23,257]. The next sections outline our current understanding of how the MVE-enriched protocrust may have contributed to abiogenesis and, specifically, to the rise of the RNA World.

### 3.2. Earth’s Volcanic and Geothermal Activity After the Moon-Forming Impact

The solid protocrust must have thermally isolated the hot mantle from the Earth’s surface and thus enabled the cooling of the latter, see Figure 3. Consequently, when, after the end of the PLD fallout, the surface temperatures became compatible with life, the Earth must have been blanketed with kilometers-thick MVE-enriched protocrust [301]. Being dominated by silica and depleted of heavier REs such as iron, the protocrust must have floated above the still-molten iron-containing upper mantle.

Because of the hydrogen escape and lack of water in the PLD, the Earth’s surface must have been essentially dry after the end of the PLD fallout. Therefore, in our further reconstruction, we rely on the model of the dry young Earth as developed by Moroz and Mukhin [325,326] and covered in detail in Section S2.12. One advantage of their modelling, which considered the atmosphere of the Earth (and other planets) in interaction with geological processes, is its predictive power. Moroz and Mukhin were the first to identify the CO_2_ feedback loop as an important climate factor [325,326]; the idea was popularized later by Walker and his colleagues [327]. This feedback loop maintains the Earth’s mild climate and is considered the key element in current global warming models. Furthermore, based on their calculations, Moroz and Mukhin predicted that the emerging life, by consuming CO_2_, could have modulated the constantly operating CO_2_ feedback loop and caused glaciation episodes [325,326]. Evidence for such glaciations was later found [328]. Also, Moroz and Mukhin predicted a clement climate throughout the Hadean, which was confirmed by the analysis of Hadean zircon grains [6,7,8,9,10,11,12].

#### 3.2.1. Formation of the Secondary Atmosphere Through Volcanic Activity

The PLD must have been depleted of both water and CO_2_. It probably never got to the point of CO_2_ formation in the cooling PLD; already at very high temperatures, carbon must have been sequestered as carbides (also found in lunar regolith [329]), which precipitated on Earth. Hence, the PLD itself was unlikely to contribute significantly to the recovery of the atmosphere. In contrast, the solidifying protocrust must have contributed by releasing the dissolved volatiles. We estimate the initial atmospheric pressure after the completion of the MVE fallout as 10^−3^ ÷ 0.5 bar. The lower bound estimate is from the model of Moroz and Mukhin [325,326], while the upper bound estimate is that derived for the Archean atmosphere from analyses of fluid occlusions in 3.5–3.0 Ga rocks [330] and gas inclusions in 2.7 Ga rocks [331,332].

Once the solid protocrust separated the atmosphere from the molten mantle, the further solidification of the latter must have continued from the bottom up with the extrusion of volatiles into the residual melt [307,322,333]. These volatiles ascended through the melt to be erupted by countless volcanic systems, see Figure 3 and [334,335]. This type of volcanic activity is currently observed on Jupiter’s satellite Io, which has a molten mantle and a thin atmosphere [336]. The volcanoes of the Io have been considered as the closest analogs to the volcanic activity on the post-impact Earth [334,335]. Since the impact-melted upper mantle must have covered the entire Earth beneath the protocrust, at least several thousand volcanoes must have been scattered over the Earth’s surface (as is now observed on Io [336]); they cooled the mantle and built up the secondary atmosphere [334,335]. Volcanoes must have ejected mostly water vapor and CO_2_ with smaller amounts of nitrogen and sulfur compounds [337,338].

After the cooling of the Earth’s surface, the climate must have been very frosty due to the faint young Sun. Its radiation output was ~70% of today’s which must have been enough to keep the average surface temperature only in the range of −50 °C ÷ −25 °C, see Section S2.12 and [325,326,339,340]. Moroz and Mukhin argued that the discharging CO_2_ must have increased the greenhouse, while the water vapor, promptly freezing as ice and snow, retarded the warming of the Earth by increasing its albedo [325,326].

In general, the geology during the Hadean must have been highly dynamic, with the eventual collapsing of exhausted volcanic systems and the emergence of new ones. These dynamics may have provided the conditions for the formation of Hadean zircons [6,7,8,9].

#### 3.2.2. Chemically Rich Vapor Got Condensed in Countless Hadean Geothermal Pools

**Figure 5:**
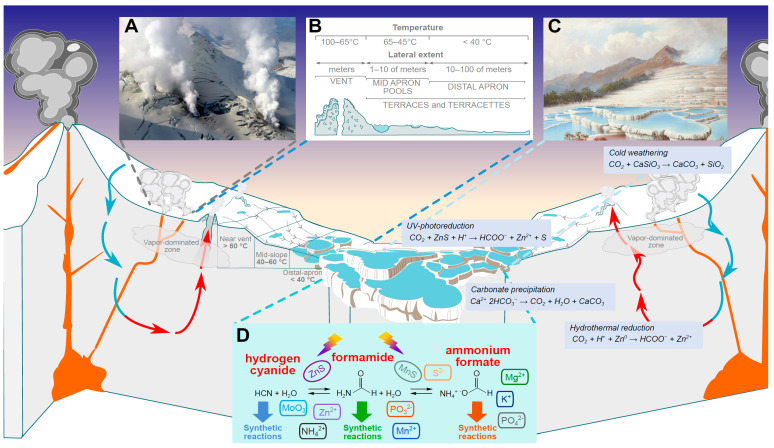
Scheme of a geothermal valley between two snow-covered Hadean volcanoes. The fumaroles, thermal springs, apron pools, and terracettes are shown based on the reconstruction of the 3.48 Ga old Hadean geothermal system [267,269,270]. On the right, various reactions of CO_2_ sequestration in a Hadean volcanic system are indicated, see Section 3.2.5 and Section 3.2.6. Inserts: (**A**) volcanic fumaroles, image from https://home.nps.gov/articles/000/fumaroles.htm, credit: USGS. (**B**) Schematic cross section of a pH-neutral hot spring, redrawn with modifications from [341,342]. (**C**) White Terraces of New Zealand as an example of volcanic terracettes (painted by Charles Bloomfield in 1884, two years before the terraces were buried under the waters of the lake Rotomahana following the eruption of Mt. Tarawera. Image credit: Museum of New Zeeland, https://collections.tepapa.govt.nz/object/42254, accessed on 23 December 2024.). (**D**) Interconversion of nitriles, amides, and ammonium salts of organic acids, represented by their simplest species, in geothermal pools in the presence of inorganic catalysts; each of the nitrogen-containing compounds shown is considered as a substrate for further synthetic reactions. Formamide and other amides, as the least volatile components, may have sustained/buffered the whole system, see also Section 1.3.1 and Section 3.3.2 and Section S2.3, and [96,97,108,123,127,128,129,130,131,132,133,134,135,137,138,139,140,141,142,143,144,145,146,147,148,343].

Figure 5 shows the structure of a typical volcanic system, see also [338]. In addition to periodic eruptions through the volcano’s throat, magma also rises through thinner conduits/cracks and interacts with groundwater, e.g., from melting snow and ice, resulting in the release of hot vapor with t° up to > 900 °C through fumaroles. The magma chamber can also be reached by descending groundwater which, once heated by the magma, becomes lighter and rises back to the surface, leaching various chemicals and bringing them to the surface. Due to the decreasing pressure of the overlying rock (decompression), the fluid begins to boil as it approaches the surface, which is accompanied by the separation of the vapor and liquid phases, which have very different chemical compositions and can discharge at different locations. The hot subsurface area, filled with hot steam and gas, forms the vapor-dominated zone with t° of 300–400 °C, see Section 1.3.4 and Section S2.8 and [23,264,265,338].

The Hadean magmas have been estimated to be hotter than today by 200–300 °C [5,344,345]. The hotter geothermal fluid and vapor must have been able to leach components with higher vaporization temperatures compared to most of today’s geothermal systems. Table 5 shows the chemical composition of the vapor condensate produced by some of the arguably hottest fumaroles studied to date, as determined by Taran and colleagues (data taken from [346]). The condensate was sampled from several fumaroles with temperatures up to 940 °C, implying a parent magma temperature >1250 °C (Kudryavy volcano, sampled in 1992). Consequently, these closest analogues to Hadean fumaroles ejected, on average, orders of magnitude more B, P, Zn, Mn, Cu, and Mo, cf. Table 3 and Table 5 and see [346,347]. Specifically, they emitted about 1000 times more phosphorus compounds than the less hot thermal springs (cf. Table 3). The phosphorus species in geothermal gases are known to be in a semi-reduced (PO_2_)_n_ form [348,349,350]. Upon interaction with water, the (PO_2_)_n_ species disproportionate to phosphate and highly soluble, reduced species like phosphite and hypophosphite, all of which may have participated in pre-biological syntheses, see Section 1.3 and Section S2.11, and [192,351,352,353,354,355].

Some (albeit not all) hot fumaroles also emit particularly high amounts of NH_3_, up to 10 g/L [356,357]. All these characteristics of exceptionally hot volcanoes of today must have been inherent and typical of Hadean volcanic systems.

While the temperature of exhalations decreases from the throat of a volcano to remote hot springs (Figure 5), the reducing power of exhalations, in contrast, increases [358]; the reason is that the *f*(O_2_) value drops with temperature, see Figure 2C. Consequently, volcanic ejecta carry H_2_O, CO_2_, N_2_, and SO_2_, which are considered to be “redox-neutral”. The vapor of hot vents is the most reduced; it carries H_2_, H_2_S, and NH_3_, as well as organic molecules formed by hydrothermal alteration [72,358,359]. The reducing power of fumarolic vapor is intermediate and depends on the chemical composition of the rock and temperature. Some fumaroles emit relatively large amounts of NH_3_ and H_2_S [357,358,359], which indicates the reduced state of the subsurface vapor-dominated zones.

As noted in Section 1.3.4 and Section S2.8, the geothermal vapor must have had a very specific chemical composition, see Table 3, Table 5 and [23,264,265,346,360,361]. It must have accumulated those substances that can exist as gases, such as H_2_S, CO_2_, and NH_3_. Being less polar than liquid water, the vapor must also have attracted organic molecules formed in hot rocks, see Section 1.3.1 and Section S2.1, as well as borate and phosphorus compounds, whose content must have been very high in the case of very hot fumaroles, see Table 3 and Table 5, Sections S2.8 and S2.11, and [23,346,350,361].

The upper layers of the protocrust must have been enriched in potassium, see Section 3.1.5. In addition, the affinity of K^+^ ions to the geothermal vapor is higher than that of Na^+^ ions, see Table 3 and Table 5. Therefore, in the Hadean vapor condensate, K^+^ must have dominated over Na^+^ which is otherwise ten times more abundant in nature, see Table 1 and Table 4. The [K^+^]/[Na^+^] ratio increases with the size of the geothermal field, reaching 75 in the vapor condensates of the world’s largest geothermal field in California, USA [362]. The Hadean geothermal fields spreading around very hot volcanoes must have been large, so that K^+^ must have dramatically predominated over Na^+^ in the vapor. Such K^+^ predominance may explain the preferential recruitment of K^+^ ions as cofactors by the evolutionarily oldest proteins of Table 2.

#### 3.2.3. A Global Network of Geothermal Pools and Basins

By analogy with the 3.48 Ga anoxic geothermal field of the Dresser Formation discovered by Djokic and her colleagues, the hot vents must have been surrounded by warm proximal ponds, with cooler pools and terracettes located in the more distant lower-lying areas as shown in Figure 5, see also Section 1.3.4 and Section S2.8, and [267,269,270]. Despite the frosty climate (see Section 3.2.1 and Section S2.12 and [325,326,339,340]), the geothermal fields were heated from beneath so that even the most remote pools and puddles may have stayed liquid, as happens in modern geothermal fields in winter. These pools must have been filled not only with condensate from the nearby hot vents, but also with the discharges of the fumaroles located up the slope, as well as with the melt of the snow and ice covering the volcanoes, see Figure 5.

In the absence of the present-day prompt oxidation of H_2_S to sulfuric acid by atmospheric oxygen, the pH of the vapor condensate must have been close to neutral [23]. H_2_S, CO_2_, and H_2_ ascending with vapor are weak acids, and their acidity must have been balanced by the interaction with basic rocks and concurrently ascending NH_3_. The Dresser Formation 3.48 Ga deposits are formed by geyserite, kaolinite/illite and borate-bearing tourmaline, i.e., minerals that form at neutral pH values, see Section S2.8 and [267,269,270]. We believe that this pH-neutrality of anoxic geothermal pools echoes in the cytoplasmic pH in almost all organisms, which is neutral or slightly alkaline.

The distal cold pools and terracettes may have been connected to their counterparts fed by other sources or other volcanoes, forming a network as shown in Figure 5. There can be up to a thousand thermal springs in a single geothermal field [270]. Thus, the Hadean Earth may have been covered by millions of hot vents and fumaroles, feeding yet more pools and ponds. One can even envision an interconnected system of anoxic geothermal fields covering the entire Earth.

#### 3.2.4. Metal Cations in Hadean Geothermal Pools: Plenty of Zn^2+^, Mn^2+^, Mg^2+^, and K^+^, but Little or No Dissolved Fe^2+^ Ions

The present scenario suggests two mechanisms that must have led to the initial depletion of the surface environments of Fe^2+^ ions.

First, iron, as an RE with a T_C_ of ~1065 °C [287], must have mostly condensed from the PLD early [292], thus precipitating and melt-mixing with the upper mantle before the silica protocrust solidified. Accordingly, the uppermost MVE-rich layer covering the solid protocrust must have been depleted of iron. Since the same MVE-rich layer must have been enriched in Zn (see Section 3.1.6), the Zn/Fe ratio in the protocrust must have been higher than in the present-day Earth’s crust. Today, the Zn/Fe ratio in the crust is ~0.001; it increases to ~0.05 in the vapor of thermal springs (see Table 3) and to ~0.3 in the vapor of very hot fumaroles (see Table 5). An arbitrary assumption of a Zn/Fe ratio of 1.0 for the Hadean protocrust yields a value of 50 for the Zn/Fe ratio in the vapor of hot vents and 300 for the vapor of hot Hadean fumaroles.

Second, the highly reduced state of the upmost layer of the MVE-rich protocrust must have prevented the release of Fe^2+^ ions. Since the protocrust must have come from the same PLD as the Moon (see Section 1.4), its average *f*(O_2_) must have been similar to that of lunar rocks still containing metallic iron (Fe^0^) as a primary phase [363,364,365,366]. This means that when the lunar crust solidified from the PLD, its average *f*(O_2_) was either below the IW level or even below the IQF level, depending on the dominant iron-bearing mineral assemblage, see Figure 1C, its caption, and [61,80,81,82]. Hence, the PLD deposits could hardly have contained Fe^2+^ under such super-reduced conditions.

However, it was concluded from the analysis of Hadean zircon grains that the *f*(O_2_) of their bathing geothermal fluids was close to that of the FMQ buffer [9]. Hence, the ascending magmatic fluid must have carried Fe^2+^ ions. On their way to the surface, the fluids, however, must have passed through the top layer of the protocrust, enriched in metallic Zn^0^ and depleted in iron. The *f*(O_2_) of this uppermost layer, essentially buffered by the Zn^0^-ZnO redox system, must have been sufficiently low (see Figure 2C) to electrochemically scavenge the passing Fe^2+^ ions.

The E_0_ value of the Zn^0^/Zn^2+^ couple (−0.76 V) is much lower than that of the Fe^0^/Fe^2+^ couple (−0.44 V), see Figure 2A. When iron and zinc can exchange electrons, they function as an electrolytic pair where the atoms of Zn^0^ get oxidized to Zn^2+^ ions while the Fe^2+^ ions get reduced to Fe^0^. Therefore, coating with Zn^0^ (zinc plating, galvanizing) is widely used to prevent the oxidation of metallic iron objects by oxygen, specifically in the car industry [367]. In the absence of atmospheric oxygen, up to 10^19^ kg of Zn^0^ in the uppermost layer of the protocrust (see Table 4), acting as a global redox buffer, must have electrochemically reduced the Fe^2+^ ions of geothermal fluids to Fe^0^ over millions of years. Notably, while the Fe^0^ atoms remained in the rock, the released Zn^2+^ ions must have been carried to the surface by the geothermal fluid and vapor; these constantly supplied Zn^2+^ ions may have been then recruited as transition metal cofactors by the first biopolymers, see Table 2.

Hence, we explain the absence of Fe^2+^ ions as cofactors in the evolutionarily oldest proteins listed in Table 2 by their scarcity in the cold, super-reduced Hadean geothermal pools.

In general, only ions of metals with E_0_ values below ~ −0.7 V (as maintained by the Zn^0^-ZnO redox buffer of the protocrust) could have been present in Hadean pools. Besides Zn^2+^, to such ions belong Mn^2+^ (the E_0_ of the Mn^0^/Mn^2+^ couple is −1.18 V) as well as Mg^2+^ and K^+^ (both with E_0_ < −2.0 V). And these are exactly the ions that are used as metal cofactors by the evolutionarily oldest proteins listed in Table 2. In contrast, the E_0_ values of the Fe^2+^/Fe^0^, Ni^2+^/Ni^0^, Co^2+^/Co^0^, and Cu^2+^/Cu^0^ redox pairs are > −0.7 V (Figure 2A), so that these ions, even if present in the geothermal fluid, must have been reduced on their way through the Zn^0^-enriched protocrust to respective metal atoms staying with the rock. Consequently, these ions are not among the physiological cofactors of the evolutionarily oldest proteins of Table 2.

Although Cu^2+^ ions were unlikely to be present in the Hadean pools (E_0_ = +0.34 V, see Figure 2A) and are absent among the cofactors of the evolutionarily oldest proteins of Table 2, the protocrust must have been enriched in metallic copper as an MVE (see Table 4). Copper may have played an important role as a powerful catalyst in the formation of organic molecules within the hot subsurface vapor-dominated zones [128,368].

#### 3.2.5. Scavenging of CO_2_ Through Chemical Weathering

The low atmospheric pressure of 0.5–1.0 bar in the Archean, 3.5–2.7 Ga ago [330,331,332], indicates a slow atmospheric buildup throughout the Hadean. One reason must have been the immediate freezing of volcanic vapor at low pressure and temperature followed by its removal from the atmosphere via the formation of polar ice caps [325,326].

The other reason must have been the scavenging of volcanic CO_2_. Moroz and Mukhin suggested that CO_2_ may have accumulated in the atmosphere until the greenhouse effect caused ice to melt, allowing CO_2_ to be trapped as insoluble carbonates (mostly CaCO_3_) in the course of chemical weathering, see Section S2.12 and [325,326]. However, such weathering may have started even earlier; liquid water must have been present in the vicinity of each volcano, so that CO_2_ may have been immobilized as water-insoluble carbonates in millions of geothermal pools and terracettes, as depicted in the right part of Figure 5. The lithium data on 4.4–4.1 Ga old zircon grains indicate that they formed within rocks that underwent weathering [369]. Intense chemical weathering is also consistent with Hadean and Archean CO_2_ levels estimated to be orders of magnitude higher than today, see Section S2.9 and [370,371].

Furthermore, while mimicking the situation on the dry surface of Mars, Garenne and his colleagues demonstrated an efficient gas–solid carbonation of Ca-bearing minerals at temperatures around freezing and at low CO_2_ pressure [372]. Thus, dry mineral surfaces of the cold Hadean Earth may have also scavenged CO_2_, see Figure 5.

#### 3.2.6. Vigorous Synthesis of Organic Molecules in Super-Reduced Geothermal Systems

The atmospheric buildup must also have been slowed by the reduction of CO_2_ to organic molecules as the geothermal fluid ascended through the highly reduced protocrust with its *f*(O_2_) below the IW/IQF/Zn-ZnO levels, depending on the depth (see Figure 2C, Section 1.3.1, Section 3.1.6 and Section 3.2.4 and Section S2.1). These *f*(O_2_) values are many orders of magnitude lower than the QFM level typical to the Earth’s crust today, which implies a vigorous reduction of CO_2_ to organic molecules via several routes, as depicted in the right part of Figure 5.

Specifically, Zn^0^, owing to its E_0_ of −0.76 V (see Figure 2A), must have acted as a powerful reductant for CO_2_. Studies on the reduction of CO_2_ to organic molecules at temperatures around 300 °C and high pressures, imitating hydrothermal processes, showed that Zn^0^ was the only metal capable of reducing CO_2_ to formate with a yield of 70–80% in the absence of other catalysts [43,373,374].

Within the hot subsurface vapor-dominated zones, Zn^0^ may have also reduced N_2_ to NH_3_, see Figure 2A. This reaction is rather complex and requires six electrons, at least. Yet, there are several reports on the reduction of N_2_ to NH_3_ by Zn^0^ [46,47,48,49]. Within the hot vents and in the presence of Zn^2+^ ions as catalysts, NH_3_/NH_4_^+^ may have further interacted with organic molecules to produce amines and amides.

Contrary to today’s conditions, CO_2_ may have been also reduced to organic molecules by minerals and solutes on the surface. In this case, Zn^0^, abundant in the rock beds of the pools, may have served as an electron donor; the respective reactions must have been congruent to the electrochemical reduction of CO_2_ to organics on zinc electrodes, see [375] for a review. In the liquid phase, CO_2_ may have been reduced by vapor-delivered water-soluble reducing agents such as dithionite S_2_O_4_^2−^ (E_0_^7^ = −0.66 V), as well as phosphite or hypophosphite anions with E_0_^7^ ~ −0.6 V, see Section 3.2.2 and Section S2.11.

Furthermore, the dissolved Zn^2+^ ions must have been partially precipitated by geothermal H_2_S and CO_2_ as ZnS and ZnCO_3_, as happens up to now [376]. The small crystals of ZnS show properties of quantum dots and photo-reduce CO_2_ to various organic molecules under the UV light with quantum efficiency reaching 80% in the case of formate, see Section S2.10 and [377,378,379,380,381,382,383,384,385,386,387,388,389,390,391,392,393,394]. Although pure ZnCO_3_ itself appears to be inactive in the photoreduction of CO_2_, it was shown to dramatically increase the efficiency of CO_2_ fixation when deposited on a photoactive surface [395]. Remarkably, the vestiges of the 3.48 Ga anoxic geothermal fields of the Dresser Formation actually contain layers of deposited zinc salts [270].

In addition, organic molecules may have been generated by ionizing radiation from about 10^16^ kg of ^40^K, see Section 1.3.1 and [50,51]. Ionizing radiation must have generated solvated electrons capable of reducing CO_2_ to organic molecules and N_2_ to NH_4_^+^, see Section 1.3.1 and [44,45,95], both in the geothermal fluids venting through the protocrust and in the surface pools. The absorbed dose rate resulting from the decay of ^40^K^+^ in a Hadean pool can be calculated as ≥1.3 × 10^−9^ Gy s^−1^ from the data in [396].

In the pools, solvated electrons may have been additionally generated by solar wind, see Section 1.3.1 and Section S2.1, and [95,96,97,122,397,398,399], as well as by the interaction of the UV quanta with inevitably present sulfur compounds in an “advanced reduction process” [93,400,401].

Furthermore, even the ice on the slopes surrounding the geothermal pools may have catalyzed the formation of various organic molecules from CO_2_ and atmospheric nitrogenous compounds under the action of solar UV light [121,402,403]; these molecules inevitably ended up in the pools brought in by thawing water.

In summary, the reduction of CO_2_ in multiple dark and light reactions, as shown in the right part of Figure 5, must have produced various organic compounds in quantities many orders of magnitude greater than those produced by geothermal systems today.

### 3.3. Multi-Step Selection of Would-Be Biomolecules in Zn^2+^- and K^+^-Rich Geothermal Fields

As envisioned in the previous Section 3.2, the super-reduced Hadean geothermal systems must have synthesized organic molecules. In addition to the substances carried by the vapor proper, the geothermal pools may have harnessed other compounds, either ejected by the volcanoes or formed in the atmosphere and on ice under the influence of UV light and the then strong solar wind, see [66,67,121,122,402,403].

The temperature and chemical composition of these pools must have varied over a very wide range, depending on their distance from the vent, the composition and temperature of the bedrock, and the chemistry of the inflow. Thus, each geothermal field was a collection of many chemical “incubators” providing different conditions [23].

Most of the ingredients identified so far as important for abiotic syntheses (see the entire Section 1.3 and Section S2, and references therein), must have been present in the Hadean geothermal fields, promoting the formation of a plethora of diverse organic molecules.

How, then, could the would-be biomolecules have been selected from such a chemical jumble? Apparently, some residents of the Hadean geothermal pools “were more equal than others” [404]. Below we consider the pre-Darwinian selection in Zn^2+^- and K^+^-rich geothermal pools.

#### 3.3.1. Selection for Low Volatility

The thin Hadean atmosphere must have been depleted of water vapor due to the frosty climate. The exhaled water vapor must have promptly evaporated and frozen under the weak young Sun, see also Section 3.2 and Section S2.12, and [325,326]. The evaporation concentrated the remaining compounds, facilitating their interactions.

Only low-volatile compounds with very high boiling points (T_B_) could have resisted evaporation at the low humidity and atmospheric pressure of the Hadean. These are simple amides (T_B_ ~200 °C; hereafter the T_B_ values are given for 1 atm), amino acids (with T_B_ ranging from 233 °C for glycine to 542 °C for tryptophan), di- and tricarboxylic acids (T_B_ ~200 °C), fatty acids (T_B_ = 360 °C for the oleic acid), branched hydrocarbon derivates such as terpenoids (T_B_ ~200 °C), glycerol (T_B_ ~300 °C), and various glycols (T_B_ ~200 °C), as well as some other compounds. Also, polar compounds such as sugars, which break down on heating before reaching their very high boiling points, must have been retained in the liquid phase. Therefore, although volcanic and fumarolic emissions must have been dominated by water, the geothermal pools must have been saturated by low-volatile organic compounds.

At the same time, the evaporating volatile organic molecules would have formed a kind of organic haze in the atmosphere, undergoing (photo)chemical transformations under the action of UV quanta and solar wind [405]. The eventually produced complex organic molecules must have fallen out and ultimately ended up in geothermal pools.

A common feature of thermal vents and fumaroles is that the intensity of their discharges oscillates [159,406]. Therefore, the proportion of water in the Hadean geothermal pools must have fluctuated, with the “anhydrous” periods particularly conducive for various condensation reactions, see also Section 1.3.2. Specifically, phosphorylation, the most widespread biochemical reaction, proceeds readily at low water activity or in anhydrous solvents, see [136,355].

Fluctuations of water content must have led to fluctuations in effective values of pH and E*_h_* since both correlate with the amount of water protons, see Section S2.1. In a water/dimethylformamide (DMF) mixture, which can serve as a mimic of a Hadean pool, the pH value, as measured by pH-sensitive dyes, increased by approx. five units as the DMF content was elevated from zero to ~50% [407]. This change corresponds to a decrease in effective E*_h_* by ~0.3 V, see also Section 1.3.1 and Section S2.1. In other words, owing to the water evaporation, the effective E*_h_* of the remaining solution in the pool may have decreased to −0.6 ÷ −0.7 V.

Notably, due to the presence of low-volatile organic compounds, the pools would not dry up even if all the water evaporates, thus constantly allowing interaction between its constituents.

Thus, the Hadean geothermal pools not only harnessed and processed various chemical ingredients supplied by the geothermal vapor, volcanic gases, and atmospheric reactions, but also selected for low-volatile substances.

#### 3.3.2. Selection for Associative Behavior

The low-volatile compounds boil at higher T_B_ because their molecules strongly interact with each other, showing so-called associative behavior [408]. Apart from water, associated liquids include organic acids, certain aldehydes, alcohols, amino acids, and polar amides/amines, as well as some phosphorus and sulfur compounds.

Notably, mixtures of associative compounds may remain liquid at temperatures far below the freezing points (T_F_) of their constituents. This property is exploited in antifreeze mixtures used in cars. For example, certain mixtures of water (T_F_ = 0 °C), formamide (T_F_ = 2 °C), and ethylene glycol (T_F_ = −16 °C) have T_F_ as low as −82 °C [409]. Hence, the Hadean pools may have remained liquid well below the freezing point of water.

Thus, molecules with associative behavior had a good chance to stay in the liquid phase of the pool. Other organic molecules were not so lucky; they either evaporated, as discussed above, or clamped together into tar particles (see Section 1.3.1 and Section S2.2).

Associative molecules not only had a better chance of retaining their “individuality”, but they may even have “rescued” other molecules. As considered in Section 1.3.1 and Section S2.2, the sugars produced in Butlerov’s reaction are specifically stabilized by borate anions that prevent their caramelization [115,117,118,410]. Formamide is effective in the industrial extraction of nitrogen-containing heterocyclic compounds from coal tar [411]; thus, formamide, together with other amides, may have prevented the “tarization” of nitrogen-containing compounds by forming strong H bonds with them.

Hence, to survive in the liquid phase, the abiotically formed organic molecules had to team up with matching tenants of the pool.

#### 3.3.3. Selection for Affinity to Silica Minerals

The Hadean geothermal vapor must have carried silica, SO_2_. Earlier we argued that it must have precipitated as porous silicates, such as clays or sinter, because the condensate of geothermal vapor must have had a neutral or slightly alkaline pH under the anoxic conditions [23]. In consistence with this prediction, the proximal pools surrounding the 3.48 Ga old hot vents of the Dresser Formation were layered by sinter, while the distal pools contained a mixture of sinter, kaolinite, and illite [267,269,270]. Thus, the anoxic Hadean vents must have resembled present-day neutral hot springs, see Figure 5B, Section S2.8, and [341,342,412,413,414,415,416].

Consequently, the Hadean geothermal pools must have contained a mix of water, low-volatile compounds, particles of silica, clumps of tar, and crystals of metal sulfides, as well as amphiphilic molecules. It has been shown, in the framework of nanotechnology research, that such mixtures could produce honeycomb-like meso- and nanoporous structures with the amphiphilic molecules lining up the pores, see [417,418,419,420,421] for reviews.

**Figure 6:**
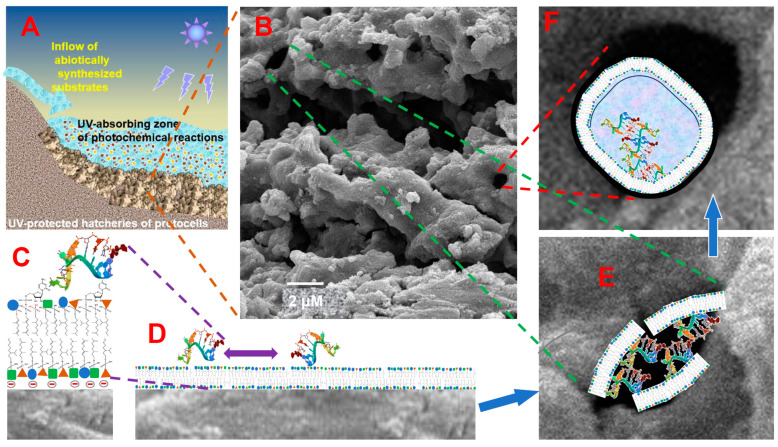
Silicate minerals and the first RNA organisms. (**A**) Digitate sinter deposits at the bottom of a geothermal pool containing dissolved UV-adsorbing particles, see also [416]; (**B**) scanning electron microscopy image of silica sinter from the Uzon Caldera (Kamchatka peninsula, Russia, see [414] for further details); (**C**,**D**) model of a sinter-lining, nucleolipid-containing bilayer interacting with RNA oligomers through Watson–Crick pairing; (**E**) a patchy protolipid “coat” of an RNA consortium within a sinter cell, and (**F**) a sealed protolipid envelope around an RNA consortium in a sinter cell picturing the first protocell. RNA molecules and protolipids are shown larger than they are in reality.

Notably, the honeycomb structures are generally common for silicate minerals [422]. Specifically, sinter forms porous structures in present-day pH-neutral thermal springs, see Figure 6A,B, Section S2.8, and [341,342,413,416].

Hence, binding to a polar surface of a mineral was another way for organic molecules or their associations to avoid the tar. Silica surfaces are negatively charged at neutral pH (Figure 6C); therefore, only polar molecules may have bound, either directly, if they carried positive charges or protonated groups, or, if negatively charged, via divalent metal cations (e.g., Mg^2+^) as linkers [423].

Thus, the individual survival of nascent organic molecules in the Hadean geothermal pools must have depended on associative behavior in a very broad sense, including not only the ability to enter favorable interactions with other dissolved molecules but also the ability to interact with negatively charged surfaces.

#### 3.3.4. Selection for UV and Radiation Resistance

The Hadean atmosphere must have absorbed the harsh UV radiation through volcanic sulfur compounds—by analogy with Io—and, perhaps, gaseous organic molecules [336,405,424]. Yet, in the absence of oxygen and the ozone layer, a part of the UV radiation with λ > 240 nm must have reached the Earth’s surface [5,222,339,424].

The geothermal pools must have provided further protection not only by the water layer itself [425], but also by porous silica precipitates and water-dissolved UV-absorbing components, i.e., nascent organic molecules, sulfur compounds, and mineral particles [23,426,427,428,429]. Sagan has estimated that the UV component must have been attenuated by a factor of up to 10^9^ to avoid irreparable damage to the first organisms [222]. The molar absorption coefficient of ZnS particles is about 2000 M^−1^ cm^−1^ at 260 nm [430]. It is easy to estimate that a ~0.5 mM solution of ZnS in the form of suspended particles attenuates the UV radiation 10^9^-fold already at a depth of 10 cm [23]. This is a lower bound estimate, because other mineral and organic constituents of a Hadean pool must have also absorbed UV radiation.

Still, the survival of molecules must have also depended on their own UV resistance. At least three different strategies may have ensured survival. First, the UV damage must have been low for those molecules that did not absorb light at >240 nm. These are simple organic molecules, such as sugars, amino acids and organic acids. Second, UV damage must be minimal for molecules promptly converting the energy of an absorbed UV quanta into heat. As discussed in Section 1.3.3 and Section S2.6, natural nucleosides and cyclic nucleotides are the champions in this regard; they dissipate the UV energy into heat in femtoseconds [212,213,214,215]. Third, the molecules with particularly large conjugated systems of alternating single and double bonds can also efficiently dissipate UV quanta [19]. These molecules are usually colored; their lowest excited energy level corresponds to the energy of visible light quanta. Such molecules do absorb UV light but then relax to their lowest excited state at femtoseconds, converting about half of the absorbed energy to heat. The remaining absorbed energy dissipates more slowly but is usually not sufficient to cause photodamage. Most notably, such colored compounds constitute the active parts of ribonucleotide-containing cofactors shown in Figure 1E, which presumably come from the RNA World, see Section 1.3.3 and Section S2.5, and [204,205]. Thus, the UV selection must have led to the accumulation of radiation-resistant molecules, even if they were chemically rather complex.

Saladino and his colleagues discovered that the irradiation of formamide by a proton beam, as a mimic of solar wind, produced a rich array of organic molecules, including sugars, organic acids, and nucleosides [96]. While the intensity of ionizing radiation must have been more or less uniform through the ^40^K-enriched protocrust, the Hadean geothermal pools must have been stratified with respect to the UV light and solar wind particles, with the upper, more vulnerable layers “harvesting” the light and solar wind to produce organic compounds, and the deeper, better UV-protected layers facilitating the interactions of organic molecules within porous silica precipitates, leading to even more complex compounds, see Figure 6A. Both the light gradient and the interlayer metabolite exchange are typical of modern stratified phototrophic microbial communities, see [431].

#### 3.3.5. Selection of Nitrogenous Compounds

As Figure 1 shows, polynucleotides and proteins are particularly rich in nitrogen. In contrast, the ratio of N to C in unanimated environments, such as volcanic vapor or carbonaceous chondrites, is lower, on the order of 0.1 [310,346]. The content of nitrogen in abiotically produced organic molecules is barely detectable [432]. Remarkably, the Zn- and K^+^-rich protocrust may have promoted the nitrogen enrichment in several ways.

The aforementioned ammonia in fumarolic vapor [357,358,359] can be attributed to NH_4_^+^ cations occupying some of the K^+^ sites in silicate rocks owing to their same diameter as K^+^ cations [433,434]. Consequently, the enrichment of the protocrust with precipitated potassium must have led to the formation of K^+^-containing minerals acting as reservoirs for NH_4_^+^ cations. In addition, within hot subsurface vapor-dominated zones, (i) Zn^0^ may have catalyzed the reduction of N_2_ to NH_3_ [46,47,48,49], (ii) the Zn^2+^ ions may have catalyzed the amination of organic molecules, and (iii) nitrogenous compounds may have formed through the dehydration of ammonium salts of organic acids, see Figure 5D, Section 1.3.1 and Section S2.3, and [123]. Out in the geothermal pools, the ZnS particles may have efficiently photocatalyzed further transfers of amino groups, see Section 3.2.6 and Section S2.10, and [390,391,392,435,436].

In the geothermal pools, the nitrogenous compounds must have persisted owing to their low volatility and high associative capacity, see Section 3.3. Organic molecules usually associate via hydrogen bonding (H-bonding), whereby protonated nitrogen atoms often act as potent hydrogen donors. Specifically, the strong H-bonding of low-volatile amides is due to the resonance structure of the N-C-O amide module [437], see also Section 1.3 and Section S2.3. Therefore, nitrogenous compounds must have preferentially accumulated in the pools as water evaporated.

Furthermore, molecules having protonated nitrogen atoms were more likely to adsorb on negatively charged silicate surfaces, see Section 3.3.3.

Also, the extreme UV resistance of natural nucleotides is attributed to the femtosecond dumping of the excess excitation energy via torsional rotation around CN bonds and the prompt deprotonation of the amino groups, with proton shifts within Watson–Crick H bonds being particularly fast [212,213,214,215]. Consequently, nitrogenous compounds had to additionally accumulate as the most UV resistant.

Thus, although the abiotic formation of organic molecules may have proceeded via multiple routes, the Zn- and K-rich Hadean geothermal systems must have specifically selected for low volatile, associative, mineral-affine, radiation-resistant, and nitrogen-rich compounds.

### 3.4. Emergence of the RNA World in Zn^2+^- and K^+^-Rich Anoxic Geothermal Fields

The concept of a primordial RNA World, central to current ideas about the origin of life, implies that the earliest organisms were consortia of small RNA-like molecules capable of self-copying, see Section 1.3.3 and Section S2.5, and [164,165,166,167,168,169,170,171,172,173,174,175,176,177,178,179,180,181,182,183,184,185,186,187,188,189,190,191,192,193,194,195,196,197,198,199,200,201,202,203]. Given the vastness of the topic, we focus here only on how the Zn^2+^- and K^+^-enriched Hadean geothermal basins may have promoted the emergence of RNA-based life.

#### 3.4.1. Zinc-Promoted Synthesis of Nucleotides

As discussed in Section 1.3.3 and Section S2.5, many pathways of abiotic synthesis have been shown for ribonucleotides [55,126,127,438,439,440]. However, only one of them, as reported by Becker and his colleagues, has so far provided a “unified prebiotically plausible synthesis of pyrimidine and purine RNA ribonucleotides” see Section S2.5 and [440]. This synthesis mimicked events in a shallow pond undergoing wet/dry cycles. Upon successive steps of this synthetic pathway, 3-aminoisoxasole with T_B_ as high as 225 °C was used first as a low-volatile solvent and later as a key common intermediate which was converted to N-isoxazolyl-urea using Zn^2+^ ions as catalysts. In another catalytic step, Zn^0^ was used to reduce nitroso-pyrimidines to formamidopyrimidines.

Just recently, Chen and her colleagues have found that the combination of Zn^2+^ and NH_4_^+^ ions provided “an unprecedently selective ribosylation condition to access furanosyl nucleosides from unprotected ribose and nucleobase” [441], solving thus the problem that had troubled chemists for decades.

The exceptional resistance of nucleosides and nucleotides to the UV light and high-energy radiation in general (see Section 1.3.3, Section 3.3.5 and Sections S2.6 and S2.7, and [96,126,212,213,215]) suggests the selection of these compounds for just this property under the solar UV light, see [20,126,211,212,215].

All of this adds plausibility to our scenario of abiogenesis in sunlit Zn^2+^- and K^+^-enriched geothermal fields.

Most likely, a competition between several radiation-resistant and/or UV-activatable compounds may have taken place, see [442]. Eventually, abiotically synthesized proto-nucleotides, likely to emerge in Zn-facilitated reactions [440,441], survived and accumulated in the Hadean pools as compounds that were simultaneously associative and resistant to radiation.

#### 3.4.2. Zn-Promoted Formation of RNA-like Oligomers in Hadean Geothermal Pools

The exact circumstances under which the proto-ribonucleotides may have joined into first RNA-like oligomers are likely to remain elusive forever. Most likely, “more than one way to RomeNA” [438] may have existed. Yet, the oligomerization may have been Zn^2+^-catalyzed. More than 40 years ago, Orgel and his colleagues discovered the exceptional ability of Zn^2+^ ions to catalyze the non-enzymatic formation of naturally occurring 3′-5′ linkages between ribonucleotides [443,444].

The non-enzymatic oligomerization has been demonstrated for nucleoside triphosphates, such as ATP, see [445,446,447,448,449,450], 2′,3′-cyclic ribonucleotides [208,209,210,451,452,453], and 3′,5′-cyclic ribonucleotides [147,148].

In many cases, the formation of RNA-like oligomers proceeded on surfaces. Specifically, the abiotic polymerization of activated nucleotides up to 50-mers was shown on clays [147,450,454,455,456,457]. Alternatively, Deamer and his colleagues have shown the abiotic polymerization of nucleotides up to 50–100-mers between the layers of amphiphilic molecules (lipids), such as fatty acids, especially under wet/dry cycling conditions [445,446,447,448,449]. In these cases, the polymerization proceeded in two-component systems that contained, in addition to ribonucleotides, either silica minerals or lipids. However, the Hadean pools may have been filled with multicomponent porous silica-based precipitates whose cells may have been lined with amphiphilic molecules, see Figure 6B, Section 3.3.3 and Section S2.8, and [267,268,269,270,341,413,414,415,416]. In addition to protecting from the UV hazards, such porous structures may have facilitated oligomerization by providing pores/cells of different sizes with a variety of surfaces.

As noted in [458,459,460], binding to the surfaces must have facilitated interactions between ribonucleotides by replacing their 3D diffusion in the liquid phase with interactions in a 2D space, see also Section S2.8.

In a cool Hadean pool, the stacking interactions between nitrogen bases, because of their higher stabilizing contribution at low temperatures (see Section S1.3), may also have promoted oligomerization, specifically on the surfaces. Helical stacks of initially unlinked proto-nucleobase-like molecules were considered as precursors of RNA-like oligomers [461,462,463,464].

Even the surface-catalyzed abiotic formation of phosphate-linked chains of sugar molecules may have been thermodynamically favorable at low water activity and low temperatures of Hadean pools, see Section 1.3.2 and Sections S2.5–S2.7, and [355]. However, the UV light could have broken such chains by acting on phosphate linkers. In this case, the condition for the survival of sugar–phosphate chains could be their acquisition of UV-protectors, the most effective of which were nitrogen bases, see Section 1.3.3 and Sections S2.6 and S2.7, and [20].

We believe that all these mechanisms may have contributed to a greater or lesser extent. Ultimately, the polymers of photo-resistant natural nucleotides, able to wind into double helical segments, may have prevailed, since the formation of double helices must have dramatically increased resistance to both UV light and hydrolysis [20,224,225,465].

#### 3.4.3. Emergence of Self-Assembling and Self-Recovering RNA Consortia

Our “frosty” geo-evolutionary scenario incorporates elements of the Cold RNA World concept [164,165,166,167,178,183,188,197,466], which is related to the earlier ideas of the cold origin of life, see Section 1.3.2 and Section S2.4 and [160,161,162,163,325,326,467,468]. Since RNA molecules are quite durable at 0 °C and below, the Cold RNA World concept provides a solution to the problem of the RNA thermolability. Indeed, without using enzymes, Holliger and his colleagues obtained >200-mers of RNA at −7 °C by applying freeze/thaw cycles for the periodic elimination of water [165,166,167,183,197].

In contrast to typical Cold RNA World models [164,165,166,167,178,183,188,191,197,466], our reconstruction provides a very broad spectrum of conditions for primordial synthetic reactions—from very hot to very cold. For example, the abrupt cooling of the geothermal vapor condensate must have prevented the decomposition of organic molecules synthesized within subsurface vapor-dominated zones and hot vents. These molecules were then delivered to cold apron pools and terracettes (see Figure 5), to become involved in the UV-driven formation of even more complex, but radiation-resistant compounds.

Since heating unwinds double-helical RNAs, the temperatures ≤ 0 °C must have stabilized them, see Section S1.3 and [178,201,466]. Therefore, at low temperatures, even short oligonucleotides may have joined into stable consortia [178,466]. They may have been stabilized via H-bonds between their daggling ends (canonical interactions), as well as by noncanonical and stacking interactions between nucleotides of different oligomers. The ions of K^+^ and Zn^2+^ may have additionally stabilized such consortia; Zn^2+^ is the most abundant transition metal cofactor found in the RNA structures [22,23,29,469] while ions of K^+^ specifically promoted the RNA folding in many cases [259,261,470,471].

The association of short oligonucleotides into consortia must have increased their survival probability. Indeed, as noted in Section 1.3.3 and Section S2.6, nucleotides quench UV quanta by converting their energy (4.8 eV or 463 kJ/mol at 260 nm) into heat in ~10^−13^ s [212,213,214,215]. However, the conversion is accompanied by a rapid heating of the UV-absorbing molecule and its surroundings, a phenomenon on which photoacoustic spectroscopy is based [472,473]. Using the temperature jump technique [473,474], it was shown that heating unwinds the double-helical segments of RNA in milliseconds [473,475], increasing the probability of their hydrolytic cleavage. The consortia must have prevented the degradation of their members by (i) spreading the energy of the UV quanta over a larger number of atoms and (ii) preventing the unwinding of helical segments through their dense packing within consortia.

Survival must also have depended on the ability to recover the original compact structure after a perturbation. The complementary interactions between bases must have ensured a rapid recovery. Small oligonucleotides, after a temperature jump, have been shown to return to their original thermodynamically stable state, with maximum H bonds between bases, in milliseconds [473,475,476].

Ultimately, the Hadean pools may have accumulated RNA-like molecules because of their ability to (i) quench high-energy radiation, (ii) form double-helical segments, (iii) assemble into tight consortia, and (iv) promptly restore their native, lowest energy structure after large thermal fluctuations and other perturbations caused by the high-energy radiation or interactions with other residents of the pool.

#### 3.4.4. Emergence of Self-Copying Organisms Through Consistent UV Photoselection

The UV-absorbing molecules in a geothermal pool, acting as sunscreens, must have protected all the molecules below them from photodamage [222,427]. Owing to this phenomenon, a chain of UV light-driven positive feedback loops may have led to the emergence of self-copying RNA consortia:

1. Photodamage to nascent organic molecules must have been less in those pools where conditions favored the formation of many UV-absorbing compounds, see also [427].
2. More sheltering by UV-absorbing compounds ensured the survival of more complex molecules, such as oligonucleotides, e.g., within porous silica precipitates (Figure 6);
3. Photodamage must have been further attenuated when the consortia of oligonucleotides acquired the ability to copy themselves, thereby increasing the density of effective UV sunscreens.

Step (3) is quite plausible. Several self-copying systems based on cooperation between oligonucleotides have been obtained and studied [179,180,201,203]. It does not seem unrealistic that over millions of years at least some of the millions of geothermal basins may have promoted the assembly of self-copying consortia—like those de facto obtained in a handful of laboratories working on the subject for only a few years.

This emergence of self-copying RNA consortia would have started the transition from the above-described multi-step abiotic natural selection to Darwinian evolution. Self-copying must have provided a clear evolutionary advantage under high-energy radiation: the appearance of each new UV-absorbing, radiation-resistant RNA consortium helped to protect the “neighbors below” from photodamage, thus serving the entire population. It is tempting to speculate that populations may have emerged as evolutionary units in the context of mutual protection against radiation damage.

Obviously, the first self-copying consortia had to self-assemble, which must have largely prevented the “error catastrophes”, see Section S2.5 and [184,477]. Oligonucleotides copied with errors simply could not fit into the forming consortia, so that the assembled consortia must have contained only acceptably “correct” oligonucleotides.

The self-copying RNA consortia may have been followed in evolution by consortia capable of template-guided self-replication using single nucleotides as building blocks [193]. Prototypes of such RNA systems were obtained and studied as well, in particular in eutectic systems at subzero temperatures [165,478,479,480,481,482].

Thus, the emergence of the RNA-based life may have been a result of a multi-step natural selection for self-assembling RNA consortia capable of self-recovery and self-copying.

#### 3.4.5. Catalysis in the Cold RNA World: Did Ribozymes Turn over Faster in Hadean Geothermal Pools?

The heating caused by the absorption of UV radiation could have been mitigated by channeling the absorbed energy into work, such as forming chemical bonds. This must have favored the survival of RNA-like molecules with catalytic properties [20,21].

In general, RNA molecules possess an intrinsic ability to cleave and form phosphodiester bonds [197,483,484,485,486,487]. This intrinsic ability is specifically exploited by catalytic RNA molecules called ribozymes, which are also capable of several other chemical reactions, namely aminoacylation, phosphorylation, alkylation, nucleotide synthesis, and RNA-dependent polymerase activity [188,202,466,478,486,488,489,490,491,492,493,494,495,496,497,498,499]. Also, ribozymes can attach nucleotide moieties to various compounds through (phospho)ester bonds, which is justified both by the discovery of ribozymes with such ability [500,501] and by the presence of nucleotide moieties in many key enzyme cofactors shown in Figure 1E. In the RNA world, Watson–Crick interactions must have enabled RNA molecules to grab such cofactors by using their nucleotide moieties as ’handles’ [204,205].

The discovery of RNA molecules selectively recognizing certain metabolites (riboswitches) [502,503,504,505,506] implies the ability of oligonucleotides to specifically bind small molecules using non-covalent interactions. Such a binding must have been stronger and more specific at low temperatures.

A certain disadvantage of ribozymes is their slowness compared to protein enzymes [507,508]. Since ribozymes usually contain double-helical RNA segments, they are thought to be kinetically limited by the need to unwind these segments by breaking the H-bonds between complementary nucleotides [509]; ribozymes must wait long for a thermal fluctuations large enough to overcome the activation barrier of unwinding.

Hadean K^+^- and Zn^2+^-rich geothermal pools may have facilitated ribozyme catalysis in several ways. First, the unwinding must have been facilitated by heating the ribozymes by the UV quanta, see Section 3.4.3. Second, Zn^2+^ and Mn^2+^ ions, that must have been constantly delivered with vapor, have been shown to accelerate the catalysis and folding of ribozymes by up to several orders of magnitude as compared to the commonly present Mg^2+^, see [510] for a review. Third, the low-volatile associative organic molecules (see Section 3.3) must have facilitated the unwinding of double-helical segments by providing alternative strong H bonds to the nitrogen bases. For example, each 1% increase in formamide concentration decreases the temperature of RNA unwinding (melting) by 0.6 °C [511], a property widely used in laboratory practice. Not surprisingly, formamide has been shown to accelerate the activity of ribozymes [512,513,514,515,516], with maximal activity observed at about 40% formamide [513,515]. Furthermore, other low-volatile reagents such as dimethylformamide, glycol, or glycerol act in the same way and even synergistically with formamide [517,518]. Therefore, ribozymes may have worked faster in the associated liquid of geothermal pools.

Thus, the cold K^+^- and Zn^2+^-enriched Hadean pools may have been populated by literally vibrant UV-absorbing RNA molecules prone to chemical reactions even in the cold, in contrast to other molecules such as sugars or organic acids that were UV inert at λ > 240 nm and could have served as substrates for these chemical reactions at best.

#### 3.4.6. Origin of Membrane-Encased Protocells in the Cold RNA World

The amphiphilic molecules, as generated in hydrothermal reactions and delivered by vapor to geothermal fields [43,519,520,521,522], may have interacted with silica minerals lining up the pores/cells in sinter deposits, as they do in artificial meso- and nanoporous structures, see Figure 6 and [417,418,419,523].

The abiotically formed amphiphilic molecules must have varied in length and structure and been both linear and branched, see [34,43,119,520,524,525,526,527,528,529,530,531,532,533]. Surfaces may have facilitated their assembly by ensuring electrostatic interactions between their polar heads and minerals, as shown in Figure 6C. At neutral pH, the surface negative charge density of silicates (mica) is similar to that of layers of fatty acids or biological lipids [534], which may not be coincidental.

The low temperatures must have additionally stabilized such primitive self-assembled bilayers, see Section S1.3. Therefore, at subzero temperatures, bilayers made of quite different amphiphiles may have been acceptably stable, see also [193,521,530,535,536,537,538,539,540]. Thus, the diversity of amphiphiles must have been less critical in cold Hadean pools.

Furthermore, the vestiges of the RNA World in modern organisms help to reconstruct how the RNA consortia may have interacted with layers of amphiphiles.

Although the lipids of archaea are fundamentally different from those of bacteria and eukaryotes (see Section S1.4 and [541,542]), the attachment of certain polar heads, common to bacteria and archaea, is in both cases mediated by the CDP-diglyceride/archaeol synthase, a K^+^- and Mg^2+^-dependent ubiquitous membrane enzyme going back to the LUCA (see it in Table 2). This enzyme attaches a CMP ribonucleotide to the protruding phosphate group of the lipid precursor yielding a *nucleolipid*, see Figure 6C and [543,544,545,546]. Next, specific enzymes replace the CMP ribonucleotides with appropriate polar heads [547].

Since the polar heads could be attached directly to the protruding phosphate groups of lipid precursors, the interim attachment of a CMP moiety makes little sense today—unless the attachment of “grabbable” nucleotides to protolipids by ribozymes may have provided a selective advantage in the RNA World. And indeed, as shown in Figure 6D, after ’saddling’ the mobile nucleolipids via Watson-Crick interactions, oligonucleotides and their consortia may have ’ridden’ over a 2D surface and interacted with each other, see also [459,460,484,548].

In this context, nucleolipids, still present in all cells as biosynthetic intermediates, can be considered as relics from the RNA world, similar to the ribonucleotide-containing cofactors shown in Figure 1E. A CDP moiety was a good choice for a tag because the Watson–Crick interaction is stronger between C and G than between A and U, see Section S1.1.

Owing to the strong hydrophobic interaction between the tails of protolipids (see Section S1.1), an RNA consortium must have been able to propriate an entire protolipid patch through just a few Watson–Crick interactions with nucleolipids, which may have eventually led to the acquisition of patchy protecting “coats” by RNA consortia as shown in Figure 6E. Ultimately, this development may have led to the first completely encased protocells, see Figure 6F.

The first protocells are thought to have membranes of single-tailed lipids with diverse polar heads, see Figure 6C and [34,524,530,531,549]. Such membranes are permeable to small molecules, including single nucleotides [40,537]. Therefore, they must not have blocked the delivery of building blocks for the new oligonucleotide consortia. However, the larger nascent oligonucleotides, unable to escape, must have accumulated within protocells causing congestion, membrane destabilization, and the spontaneous division of a protocell into two protocells. Such a primitive division has been mimicked by Szostak and his colleagues using model lipid vesicles containing RNA molecules [550,551,552] and has been observed in bacterial cells with deliberately disrupted cell division machinery [31,553]. The primitive protocell division, driven by purely physicochemical reasons, completed thus the transition from RNA consortia to reproducing protocells.

Thus, nucleolipids may have played a crucial role in evolution by coupling the two self-assembling systems, namely (i) RNA consortia and (ii) amphiphilic bilayers.

### 3.5. Timing of Abiogenesis

The Moon-forming impact is thought to have occurred 4.51–4.40 Ga ago [42,283,298]. Estimates of the cooling of the Earth’s surface to temperatures compatible with life vary widely, from 0.01 Ma to 200 Ma, see [5,554] for reviews. This cooling time must have depended strongly on the atmospheric pressure of CO_2_ and water vapor [333]. Our scenario assumes a rather thin and dry atmosphere after the complete fallout of the PDL content, see Section 3.2.1. In this case, the cooling could have been completed in <10 Ma [325,326,333,555].

It has been noted that the origin of life could have been delayed by strong elementary particle winds from the young Sun [122]. However, the irradiation of formamide with a proton beam mimicking such a solar wind has produced a variety of different organic molecules, including nucleosides [96,97,397,398,399]. Thus, the solar wind, especially attenuated by geothermal basins, may even have been useful both as a source of reducing energy in the form of solvated electrons (see Section 1.3.1) and as a selective factor that destroyed radiation-susceptible molecules (see Section 1.3.3 and Section S2.7). Thus, the solar wind was not likely to have prevented the early origin of life, although the demolishing effects of a particularly strong solar wind early on cannot be completely ruled out.

The origin of life by the mechanism described here must obviously have become impossible after the onset of plate tectonics, supposedly, between 4.0 and 3.0 Ga [556,557,558,559,560]. Due to the plate subduction, the entire MVE-enriched protocrust must have been dragged into the mantle and mix-melted. However, even before that, the ability of the Zn^0^-enriched post-impact protocrust to reduce CO_2_ to precursors of biomolecules must have diminished over time on the timescale of 50–300 Ma owing to the continuous oxidation of protocrust by the magmatic fluid, see Section 3.2.4 and [9,199,561,562,563]. Thus, it would be in the interest of life to emerge as early as possible, just as the Earth’s surface cooled after the lunar impact.

Consequently, depending on the timing of the lunar impact, the cooling duration of the Earth’s protocrust, the strength of the solar wind, and the lifespan of the super-reduced, Zn^0^-rich protocrust, life may have originated no earlier than 4.5 Ga and no later than 4.1 Ga. This time period is consistent with current estimates of when life began, which range from 4.5 to 3.7 Ga, see [5,199,554] for recent reviews.

### 3.6. Adaptation of Life to the Earth’s Recovery from the Moon-Forming Impact

The multi-step natural selection of ribonucleotides and their self-assembling oligomers must have required an ample supply of simpler building blocks such as sugars, nitrogenous compounds, amphiphilic molecules, and so on. Thus, RNA organisms must have been dependent on the nutritious “primordial soup” provided by inanimate nature [564]. As Bernal put it, “…protoorganisms need not have been autonomous at the beginning. They existed and could exist only in a general chemically active medium” [153].

The diversity of abiotically produced building blocks must have been additionally ensured by their interconvertibility. It was shown that the incubation of just one intermediate of either the tricarboxylic acid cycle, glycolysis, or the pentose phosphate pathway in warm water and in the presence of transition metal ions as catalysts led to the generation of the remaining intermediates of the respective pathway in non-enzymatic reactions [565,566,567,568,569]. These data suggest that once an organic molecule is produced, the activation barriers for its further interconversions are not so high. The geothermal systems must have been particularly conducive to interconversions of simple organic molecules. They could provide a wide range of temperatures; within the hot vapor-dominated subsurface zones, the ions of Zn^2+^, Mn^2+^, Mg^2+^, and K^+^ may have served as catalysts.

So far, the available data have helped to reconstruct to some extent how RNA organisms, in their endeavor to become more self-reliant, consecutively mastered the non-coded protein synthesis [570,571,572,573,574,575,576], developed the genetic code [171,577,578,579], and evolved the first coded peptides, which assembled into the first proteins [580,581,582]. Since the evolutionarily oldest proteins almost exclusively use metal cofactors with E_0_ < −0.7 V (see Table 2 and Section 3.2.4), all the above evolutionary steps must have taken place while the geothermal systems were still super-reduced.

But the “primordial soup” must have thinned out as the Zn^0^-rich protocrust got oxidized, see the previous Section 3.5. Yet, with the oxidation of the protocrust, cations of Fe^2+^, Ni^2+^, and Co^2+^ became available. Consequently, the first organisms learned to produce the compounds they needed by recruiting these “new” metal ions as redox cofactors, which led to sophisticated multi-subunit enzyme complexes capable of (i) reducing CO_2_ to organic compounds [583], (ii) converting N_2_ to NH_4_^+^ [584], and (iii) performing chlorophyll-based, CO_2_-reducing photosynthesis [19,63,585].

Apparently, all of these innovations were urged by the Earth’s recovery from the lunar impact. The gradual evolution of life in geothermal fields, the roles of Zn^2+^ and K^+^ ions in this evolution, and the circumstances under which life split into Archaea and Bacteria and invaded new terrains will be discussed in more detail in forthcoming publications.

### 3.7. Solutions for the Paradoxes of Life

The proposed scenario implicitly offers new solutions to a dozen life paradoxes discussed in Sections S2.1–S2.12 of Supplementary File S1. Table 6 lists these paradoxes and their novel solutions, most of which are complementary to the solutions previously proposed and discussed in Section S2. Table 6 thus demonstrates the explanatory power of our reconstruction.

## 4. Discussion

Over the past years, we have captured the three facets of early life, namely (i) the involvement of UV light as a selective factor in the emergence of RNA-like oligomers [20] and photosynthesis [19], (ii) the origin of the evolutionarily oldest proteins in the primordial Zinc World [21,22,29,33], and (iii) the emergence of the first cells in the K^+^-rich pools of geothermal vapor condensate in anoxic geothermal fields [23,24,35]. The recently clarified geochemistry of the Moon formation [42,292,294,296] allowed the merging of these three lines of evidence into a single, consistent evolutionary reconstruction presented here. It pictures the Earth being galvanized, in both senses of the word, by the Moon-forming impact.

Initially, our quest was about understanding why the evolutionarily oldest proteins and RNAs depend exceptionally on Zn^2+^ and K^+^ ions and do not use more abundant ions of Fe^2+^/Fe^3+^ and Na^+^, respectively, see Table 2 and [21,22,23,29,33,35,37]. Here, we have finally managed to solve this quest by closing in on the higher volatility of Zn^2+^ and K^+^ ions. The higher volatility must have led (i) to the inevitable enrichment of the uppermost layer of the post-impact protocrust in zinc and potassium and (ii) their elevated levels in the geothermal vapor condensate presumably harboring the first organisms. Notably, Macallum insightfully wrote as early as 1926 that “the potassium was present in excess of the sodium” when “the first condensation of water took place on the rock surface” [40].

Yet, a post-impact protocrust with initial *f*(O_2_) < IW/IQF/Zn-ZnO, containing up to 10^19^ kg of Zn^0^ and 3 × 10^16^ kg of ^40^K^+^, must have possessed a tremendous reducing power, the source of which has remained the main mystery of abiogenesis for more than a hundred years.

The pioneers of life origins research recognized early on the need for a high reducing power to obtain the first biomolecules. In 1913, Moore and Webster published a paper with the eloquent title “Synthesis by Sunlight in Relation to the Origin of Life. Synthesis of formaldehyde from carbon dioxide and water by inorganic colloids acting as transformers of light energy” [98]. Oparin has attributed the reducing power to solar light, the supposedly reduced primordial atmosphere, and metal carbides [586,587]. Haldane relied on UV light as a source of reducing power [564]. In fact, the origin of life scenarios can be categorized according to the proposed source(s) of reducing power, see [45].

The hallmark of our scenario is the super-reduced Zn^0^- and ^40^K^+^-rich post-impact protocrust as the main source of reducing power for abiogenesis. The scenario also relies on our earlier notions (i) that the hot vapor-dominated subsurface zones of geothermal systems produce and accumulate exactly the chemicals needed by the first organisms, and (ii) that the cool K^+^- and Zn^2+^ rich condensate of geothermal vapor, resembling the cell cytoplasm chemistry and fundamentally different from salty geyser discharges and seawater, may have been the medium hosting the first organisms [23,24].

In addition to Zn^0^ as the main source of reducing power, our scenario invokes several other novel factors conducive to the origin of life, namely (i) very hot Hadean fumaroles as rich sources of borates, molybdates, semi-reduced phosphorus compounds, and NH_3_, see Table 5 and Section 3.2.2, (ii) the prompt evaporation of water at low temperature and atmospheric pressure, which must have decreased the water activity and effective E*_h_* in Hadean basins without heating or drying them completely, see Section 3.3.1, (iii) multicomponent, silica-based honeycomb structures serving as hatcheries for the first organisms, see Figure 6 and Section 3.3.3 and Section S2.8, (iv) the productive interplay of extremely diverse environments ranging from the fumaroles with t° > 900 °C to the cold icy slopes of Hadean volcanoes, see Figure 5, Table 5, Section 3.2.6, and (v) the multi-step natural selection for low-volatile, associative, radiation-resistant, minerals-affine, nitrogen-rich, and polymerizable organic molecules capable of self-assembling into self-copying and self-recovering RNA-based consortia, see Section 3.3 and Section 3.4.

The plausibility of our reconstruction is supported by the following lines of evidence:

(1) We build upon the current consensus model of the Moon formation, in the very last stage of which up to 10^19^ kg of Zn^0^ and ~3 × 10^19^ kg of K^+^ must have inevitably fallen on the already solidified Earth’s protocrust, see Table 4, Figure 4, Section 1.4 and Section 3.1, and [42,288,301];
(2) Our reconstruction is consistent with the properties of the Hadean zircons, the only direct samples of the Hadean Earth [6]. They are known to have formed in hydrated silicate-rich melts and in interaction with K^+^-rich minerals, such as muscovite [6,7,8]. More recently, Trail and McCollom, based on “zircon chemistry, experiments, and modeling to infer the character of lithospheric fluids” [9], excluded the oceanic contribution to these fluids and argued that they “were interacting with nearsurface aqueous systems, possibly subaerial hydrothermal pools, amplifying redox gradients in a location attractive for prebiotic molecular synthesis” [9], which is in agreement with here resurrected geochemistry of the Hadean Earth;
(3) In our reconstruction, the abiotic reduction of CO_2_ and N_2_ to organic molecules is ensured by several complementary natural processes, namely (i) the oxidation of up to 10^19^ kg of Zn^0^, as well as other super-reduced components of the juvenile protocrust, such as carbides, (ii) photogeneration of excited electrons by sunlit crystals of ZnS, MnS and TiO_2_ [377,378,379,380,381,382,383,384,385,386,387,388,389,390,391,392,393,394], and (iii) generation of solvated electrons by 3 × 10^16^ kg of ^40^K, solar wind [122], and the UV quanta interacting with sulfur compounds [88,400,401]. All these processes must have proceeded over millions of years thus increasing the likelihood of abiogenesis.
(4) Our reconstruction is also consistent with the geochemistry of the 3.48 Ga Dresser Formation hosting the oldest microbial biosignatures. The Dresser Formation contains vestiges of hot vents surrounded by sinter terracettes; the samples also show layers of precipitated zinc, which may indicate the residual Zn-enrichment of the Archaean crust, see [267,268,269,270];
(5) Life itself, by retaining memories of its origin under conditions where K^+^ and Zn^2+^ ions were in abundance (see Table 2 and Section 1.3.4), stands as a witness in favor of the proposed evolutionary reconstruction.

The evolutionary scenario considered here describes a multi-step process of natural selection in which Darwinian selection only comes into play at the very end. Yet, with each step, this process meets the definition of natural selection by Darlington Jr. as “a selective elimination of less-fit individuals,… while (if evolution is to continue) more fit individuals survive”, where fitness is defined as “the probability that individuals with given characteristics in given environments will escape elimination” [588].

In the following, we consider the performed reconstruction in relation to (i) the role of terrestrial volcanic systems in abiogenesis, (ii) the mostly overlooked zinc-centricity of life, (iii) thermodynamic constraints, (iv) other anticipated giant impacts, (v) the chances of life on Mars and other rocky planets, and (vi) other origin-of-life models.

### 4.1. Continental Volcanic Systems as Cradles of Life

One more hallmark of our reconstruction is the “division of labor” within the Hadean volcanic systems where (i) volcanoes drove the buildup of the atmosphere and produced CN-rich compounds, (ii) the hot subsurface vapor-dominated zones served as extremely powerful chemical reactors, (iii) very hot fumaroles delivered large amounts of phosphorus compounds, ammonia, and borate, (iv) thermal vents produced Zn^2+^- and K^+^-enriched super-reduced vapor laden with organics, while (v) cold distal pools and terracettes harbored radiation-resistant organic molecules prone to further low-temperature (photo)chemical transformations, which eventually led to photostable but thermolabile RNA-like molecules and their self-copying consortia, see Figure 5 and Figure 6.

The idea of continental volcanic/geothermal systems as the cradle of life was apparently first put on paper by a Russian polymath Vladimir Komarov. He wrote in 1933, drawing on his own surveys of the volcanic systems of the Kamchatka Peninsula in 1909–1910: ”Water, heated to a temperature of about 1000 °C, is the strongest solvent and, penetrating into the cracks of the rocks through whose strata it passes… is the cause of numerous chemical transformations”….”In short, volcanism leads to the formation of extremely powerful chemical laboratories.”…“At the time of the origin of life on Earth, hot springs, as formed in the still fragile lithosphere, could easily have become the first haven for life. These springs contain a variety of mineral compounds that are dissolved by superheated steam and hot water as they travel through the cracks in the lithosphere. The interaction of these solutions and their ability to form complex compounds has not yet been studied. Particularly unclear is the role of primary carbonaceous compounds of inorganic origin” [64].

Even more specifically, the role of geothermal systems in the origin of life was considered by Florovskaya [589,590]. The continental geothermal/volcanic environments have been invoked in many origin of life papers, including those of Mukhin and his colleagues [66,67,325,326], Markhinin and Podkletnov [68,69]; Washington [591], Deamer, Kompaninchenko, and their colleagues [158,159]; Ricardo and Szostak [592]; Maruyama and his colleagues [17,44,45,593]; and van Kranendonk and his colleagues [267,269,270,594,595].

Compared to these insights, our scenario is specific in its focus on (i) the super-reduced Zn^0^- and K^+^-enriched post-impact fallout as a source of reducing power for abiogenesis, (ii) the hot subsurface vapor dominated zones as chemical reactors and accumulators of substances essential to life, (iii) the cold surface basins, filled with super-reduced vapor condensate with effective E*_h_* falling to −0.6 ÷ −0.7 V upon (periodic) evaporation of water, and (iv) multiple roles of Zn and K, which we believe, together with Mg, are the metallic keys to life and its origin.

In the context of our reconstruction, the puzzling selectivity of ubiquitous proteins for their metal cofactors, evident from Table 2, finds its natural explanation. Indeed, the reduction of CO_2_ and N_2_ to organics demands an effective E*_h_* ≤ −0.7 V. Under such conditions, only metals with E_0_ < −0.7 V exist as water-soluble ions such as K^+^, Na^+^, Mg^2+^, Zn^2+^, and Mn^2+^; other metals, such as Fe, Ni, Co, and Cu, are in their insoluble metallic state. Furthermore, their non-use by the evolutionarily oldest proteins (Table 2) can be seen as evidence that the super-reduced environment still persisted on the Hadean Earth when these proteins emerged.

Remarkably, the geothermal fields must have served as an ample source of assimilable molybdenum (Mo). As a catalyst, molybdenum may have been important for the abiotic synthesis of sugars, especially ribose, see Section 1.3.1 and Section S2.2, and [192,596]. Also, molybdenum compounds may have been involved as multielectron redox catalysts in the abiotic reduction of N_2_ to NH_3_/NH_4_^+^ by Zn^0^ [46]. Many important, although not ubiquitous enzymes also use molybdenum in their redox cofactors [41,227,584,597]. Currently, molybdenum occurs in nature as its moderately soluble Mo(IV)-Mo(VI) oxoanions (molybdates). However, under primordial anoxic conditions, molybdenum has been thought to occur as insoluble salts of Mo(III) and Mo(IV), such as MoS_2_, so the source of molybdenum accessible to the first organisms has remained obscure [41,227,584]. Yet, a closer inspection reveals that the Mo-cofactors of enzymes use not just single ions of Mo^3+^ or Mo^4+^, but derivates of its oxoanions, such as molybdate MoO_4_^2−^, or molybdenum oxysulfide MoOS_2_ [41,192,227,596,597]. Relevantly, the very hot steam (t° > 300 °C) oxidizes metallic molybdenum (Mo^0^) to MoO_2_/MoO_3_/MoO_4_^2−^, as well as MoS_2_ to MoOS_2_, respectively [598,599], in reactions that require no oxygen. Even the geothermal vapor of today carries a lot of molybdate; its content rises linearly with the temperature of the vapor, cf Table 3 and Table 5 and see [346,600]. The precipitates of mixed Mo(IV)-Mo(VI) oxides even stain the fumarole environments blue [601]. The molybdate formation—not requiring oxygen—must have been possible in the Hadean; the subsurface vapor-dominated zones feeding the hot fumaroles must have produced sufficient molybdate to catalyze both the abiotic formation of carbohydrates, including ribose [596], and, eventually, the reduction of N_2_ to ammonia by Zn^0^ [46]. Fumarolic molybdates may also have been recruited by enzymes at later stages of evolution.

Our reconstruction resolves also the long-standing conundrum between the reducing conditions required for abiotic organic synthesis (see Section 1.3.1 and [89]) and the “redox-neutral” state of primordial atmosphere, formed by volcanic gases such as CO_2_, N_2_, and SO_2_, see [106,199]. Because the reducing power of hot rocks increases as their temperature drops (see Figure 2C and Section 1.3.1), the vapor-dominated zones beneath geothermal fields (t°~ 300–400 °C) are more reduced than gases passing through the throats of volcanoes (t° > 1000 °C). Despite the crustal oxidation during the last 4.5 Ga and the presence of oxygen in the atmosphere for more than 2.5 Ga, today’s condensate-fed geothermal pools still exhibit a low E_h_ of ~ −0.3 V [87]. Furthermore, the reduced phosphate species and NH_3_ in the vapor [357,358,359,602] indicate that the subsurface vapor-dominated zones are even more reduced. In the early Hadean, the vapor-dominated geothermal systems must have been much more reduced than today.

### 4.2. On Zinc-Centric Life and Iron-Centric Life Science

By trying to convince the reader of the crucial role of zinc for early life (see Table 2 and [21,22,23,29]), we are essentially breaking down an open door. In fact, Zn is the *only* transition metal essential for life [257,603]. Not a single organism can survive without zinc. In particular, the attempts to grow microorganisms without zinc were sometimes successful, only to find out later that the resulting biomass contained much more zinc than its trace amounts available in the growth medium [604]. In this way, it was discovered that microorganisms can extract significant amounts of zinc from the lab glassware [604]. Although the extraction mechanisms await elucidation, these findings reveal clearly how vital zinc is for life.

Nevertheless, this vital role of zinc is still not fully appreciated by life scientists and for a very simple reason: the zinc atoms are spectroscopically silent [605]. The Zn^2+^ ions are colorless and diamagnetic, so they cannot be studied by either spectrophotometry or electron paramagnetic resonance (EPR) methods. The very fact that Zn^2+^ ions are the most abundant transition metal cofactors—the only one to be present in enzymes of all classes—and that about 6–10% of proteins in each organism contain bound Zn^2+^ ions was discovered rather unexpectedly while searching for Zn^2+^-binding amino acid motifs using bioinformatic tools [257,469,606,607,608,609,610,611,612].

In contrast, iron is *not essential* for life. Certain fermentative microorganisms grew even when their cultures contained <10 iron atoms per cell [257,603,613,614,615,616,617,618]. These data indicate that iron is not specifically needed for the core cellular processes.

However, iron far surpasses zinc in terms of visibility. The proteins with iron-containing cofactors, such as hemes, which have been studied since the 1820s [619], are often colored. Iron is paramagnetic, which allows the use of EPR. In addition, the function of iron-containing proteins is usually associated with changes in the redox state (and color) of iron compounds, which can be monitored in a variety of ways, ranging from visual inspection to Mössbauer spectroscopy. Consequently, iron-containing enzymes have been always eagerly investigated.

In such a historic context, the use of iron by the earliest organisms was taken for granted and not in need of evidence. Consequently, the first molecular origin-of-life scenarios were iron-centric [620,621,622,623]. Some of their ideas were later incorporated into the autotrophic hypothesis that Hartman put forward as a counterpoint to the classical heterotrophic origin-of-life scenarios of Oparin, Haldane, Bernal, and Miller. Hartman postulated the emergence of the light- and iron-catalyzed metabolic cycles before polynucleotides and replication [624].

Around the same time, Corliss and his colleagues discovered vibrant life around deep-sea hydrothermal vents that ejected very hot water rich in H_2_S and transition metals, mostly iron [625]. These vents were suggested as potential cradles of life [626,627]. Later, this idea was popularized by Russell and his colleagues [628,629,630,631]. They related it to the ability of some deep-sea hydrothermal vents to produce organic molecules through hydrothermal alteration considered in Section 1.3.1 and conventionally called serpentinization in relation to oceanic basalts [632].

Following this deep-sea trend, Wächtershäuser put forward an elaborate hypothesis on the origin of life on the surface of hydrothermally precipitated iron sulfide (FeS) [458,633,634,635]. This hypothesis emphasized the usefulness of surfaces for the interaction of would-be biomolecules, as well as the need to reduce them directly on the surfaces they reside on. Specifically, Wächtershäuser suggested that the oxidation of the seafloor FeS to pyrite (FeS_2_) may have been coupled with CO_2_ reduction [458,633,634,636]. However, attempts to use FeS or other iron-containing compounds for CO_2_ reduction have not been particularly successful. Simple organic molecules, when formed, were produced in minute amounts and usually at temperatures too high to be compatible with life [636,637,638,639]. Theoretical analysis by Schoonen and his colleagues showed that the CO_2_ reduction by the FeS-H_2_S/FeS_2_ system “is hindered by a high activation energy, even though the overall reaction is thermodynamically favorable” [83]. As Smith and Morowitz have concluded in their comprehensive book on iron-centered abiogenesis, “despite the consensus that Fischer-Tropsch-type synthesis occurs at high temperatures and pressures in the subsurface, it is a separate problem to understand whether the intermediates along a C1 reduction sequence are obtainable under mild near-surface conditions” [640].

Given this iron-centered mindset, it came as something of a surprise that ubiquitous, evolutionarily oldest proteins and RNAs do not use iron as a transition metal cofactor but frequently and almost exclusively use zinc in this capacity, see Table 2 and [21,22,23,29,33,282].

These findings, however, have asked for a closer look at the properties of zinc. In contrast to iron, Zn^0^ (commercially available as “zinc dust”) is an extremely powerful reducing agent routinely used in synthetic organic chemistry; due to its low E_0_ of −0.76 V, Zn^0^ is efficient even at room temperature and normal pressure, see Figure 2A and [641,642,643].

As described in Section 3.4.1 and Section S2.5, all the four canonical ribonucleotides have been recently obtained using Zn^0^ as a reductant [440]. Yet, the triumphant march of Zn^0^ as a reducing agent for bioorganic syntheses began much earlier. In 1884, Emil Fischer, the founder of synthetic biochemistry, by using *Zinkstaub* (zinc dust) as a reductant, synthesized a new complex compound and named it *purine* [644]. Later, Fischer used *Zinkstaub* in the synthesis of sugars, including deoxy sugars [645,646]. Chemists routinely reduce diverse substances by passing them through long narrow tubes filled with zinc dust. This setup resembles, in fact, the here envisioned passage of hydrothermal fluids through the Zn-enriched Hadean protocrust. We simply argue that nature may have used *Zinkstaub* to produce nucleotides and sugars some 4.5–4.1. Ga before Emil Fischer.

It is our belief that the full recognition of the zinc-centricity of life by the scientific community will have implications far beyond the question of the origin of life.

### 4.3. Thermodynamic Considerations

Life could have emerged only at the expense of a free energy flow [423,647,648,649]. However, this thermodynamic constraint defines neither the nature of this flow nor the general properties of its interaction with the emerging living matter.

Pascal and his colleagues brought some specificity to the field by building on the assumption that any complexification of would-be biomolecules must have been accompanied by the formation of new covalent bonds [649,650]. These authors argued that whatever the mechanism of the bond formation, the product molecule must have remained stable to participate in further transformations, which implies relatively high activation barriers for the formation of biomolecules. To overcome such a high barrier, a chemical system must either wait a long time for a large thermal fluctuation or be energized by a large quantum of energy. From this, Pascal and his colleagues concluded that the abiotic syntheses involving the formation of new, stable bonds must have been activated by high-energy radiation and/or high temperature.

Both of these sources of large energy quanta are involved in our reconstruction. It envisions the formation of simple organic molecules, including the CN bond-containing compounds, within throats of volcanoes, hot subsurface vapor-dominated zones, hot vents, and warm proximal ponds (see Figure 5 and Section 3.2.6), as well as the involvement of UV light and ionizing radiation as energy sources in cool geothermal pools (see Section 1.3.4, Section 3.2, Section 3.3 and Section 3.4 and Section S2.7). After Sagan and de Duve [223,651], we suggest that the UV light and ionizing radiation may have contributed not only by driving chemical transformations and locally heating RNA consortia (see Section 3.4), but also by destroying the UV-labile molecules and providing thus new building blocks for the would-be biopolymers, see Section 3.4 and Section S2.7, and [20,21,22].

The problem of the thermodynamic stability of biomolecules has recently been addressed by Wołos and her colleagues, who have used a forward synthesis algorithm to generate a complete network of prebiotic chemical reactions accessible from just six simplest molecules, namely methane, NH_3_, H_2_O, HCN, N_2_, and H_2_S [652]. The algorithm has generated thousands of molecules, both biotically relevant and abiotic. The biotic compounds, as compared to abiotic ones, were found to be thermodynamically more stable, more hydrophilic, and more balanced in terms of hydrogen bond donors and acceptors—“biotic molecules contain on average comparable numbers of donors and acceptors, which may facilitate the formation of supramolecular aggregates” [652]. While the higher thermodynamic stability of biotic molecules is consistent with the above ideas of Pascal and his colleagues [649,650], the increased ability to form supramolecular aggregates is consistent with the here emphasized associative nature of would-be biomolecules and the importance of protonated nitrogen atoms for their survival, see Section 3.3.

On a more general level, there has been an ongoing search for thermodynamic factors that might determine the formation of organized structures driven by energy flow. Ervin Bauer has suggested that the living systems maintain the state of “sustainable disequilibrium” [647]. Ilya Prigogine showed that near-equilibrium systems driven by an energy flow show minimal entropy production in their steady state [653]. Pross and colleagues described living systems by the principle of dynamic kinetic stability according to which “autocatalysis selection in replicator space leads from kinetically less stable systems to kinetically more stable systems” [649,654].

A specific description of highly non-equilibrium systems, both living and non-living, is being developed by England and his collaborators [655,656]. The current state of the art is that the external driving forces “are predicted to bias the system towards dwelling in states where diffusive motion (rattling) is weak, either due to the onset of dynamical order or due to the overall reduction in drive energy converted into motion within the system. To put the intuition in more primitive terms: complex driven systems gravitate toward states from which it is hard for the external drive to eject them” [656]. When applied to primordial biomolecules, this rationale predicts their assembly into stable (low rattling) consortia capable of using external energy (the flow of UV quanta and/or thermal gradients) to do work, e.g., drive chemical reactions or undergo large-scale mechanical motions.

### 4.4. Resurgence of the Zn-Rich Protocrust After the Last Sterilizing Impact

Assuming that Earth has survived several giant, all-life-killing impacts, some authors have argued that life may have forever established only after the last sterilizing impact (LSI), see [657,658]. Up to now, we have only considered the Moon-forming impact because its geochemical consequences are inferable from the analysis of lunar rocks. In the absence of any evidence for other giant impacts, the parsimonious approach is to assume that the Moon-forming impact was the LSI; this appealing approach has been chosen by Kitadai and Maruyama [593]. Nevertheless, the possibility of several SI cannot be completely ignored and deserves consideration.

It has been estimated that an impactor with a diameter over 1000 km would be required to sterilize the Earth [657,659,660]. If such an impactor could have struck Earth after the Moon-forming impact but before the onset of plate tectonics, it would have collided with a planet already blanketed by an MVE-enriched protocrust (Figure 3). A new impactor would have caused melting and the partial vaporization of this protocrust, but without the spreading of the post-impact disc beyond the Roche limit—otherwise the Earth would have more than one moon. The subsequent cooling of the post-impact disk and gradual deposition of its contents on Earth must have resulted in the recovery of an MVE-rich, highly reduced protocrust, whose top layer would have been re-fertilized with Zn^0^ and underlain by a K^+^-rich layer, see Section 3.1.6 and [292]. This sequence of resetting events could have been repeated several times. Thus, after each giant SI, the Earth must have been covered with a fresh MVE-rich and Zn^0^-blanketed protocrust. Thus, our scenario must have been realized even after sterilizing impacts ensuing the Moon formation.

### 4.5. Compulsory Zn Plating of Juvenile Rocky Planets: The Zn-Rich Clays of Mars

The Moon-forming impact is sometimes considered as the last, delayed step of the Earth’s accretion [661]. It is noteworthy that any planetary accretion is thought to be accompanied by the release of large amounts of energy and the partial vaporization of the interacting planetesimals and/or planetary embryos [662]. Then, in the very last accretion stage, any rocky planet must be blanketed by MVE-enriched silicates with Zn^0^ and K in the topmost layer.

Hence, any rocky planet must be “zinc plated” at the end of its accretion. Obviously, such a blanketing by MVEs and, in particular, Zn^0^ and K^+^, might be conducive to the emergence of an Earth-type life. Yet, the amount of Zn^0^ and K^+^ must depend on the circumstances of this final accretion step, so Earth may be said to be fortunate in this respect.

Because of the inability of the MVE-enriched protocrust to survive global resurfacing (as appears to have occurred on Earth after the onset of plate tectonics, see Section 3.5), the predicted initial Zn^0^-plating of rocky planets can be checked by studying a planet that has not experienced global resurfacing.

Mars is the closest of such planets. It is thought to have undergone only local resurfacing events, as caused by impacts and local volcanic activity, and to have retained part of its initial crust [663,664].

We have inspected the literature on the presence of Zn in Mars’ soils and found out that the “Curiosity” rover using an Alpha-Particle X-ray Spectrometer (APXS) detected high levels of zinc, up to 8.1%, while traveling through the Kimberley formation within the Gale impact crater, supposedly formed 3.5–3.8 Ga ago [665,666]. Otherwise, the environment was rich in potassium and manganese. Surprisingly, no compatible amounts of sulfur were detected. Thus, Martian zinc exists not as ZnS (sphalerite), in contrast to Earth’s sphalerite-rich zinc ore deposits. It has been speculated that zinc is incorporated into Martian clays [665,666].

Hence, the studies of properties of Martian zinc- and potassium-rich silicates might be useful for modeling the origin of life on Earth and, perhaps, on other rocky planets.

### 4.6. On the Habitability Criteria

The considerations presented here also require some revision of habitability criteria. These criteria usually include a range of surface temperatures compatible with the presence of liquid water [667]. Obviously, if there is volcanic activity on a rocky planet, liquid water would be constantly present around volcanoes and on geothermal fields, even if the overall climate is extremely frosty. Furthermore, large amounts of salty water, as in seas or oceans, must have been prohibitive for the emergence of Earth-type life, see Section 1.3.2 and Sections S2.4 and S2.12, and [54,668]. This pushes the limits of habitability further away from stars. Young, frosty, rocky planets with intense volcanic activity appear to be the most promising targets in the search for the Earth-like life. There must be an untold number of such planets out there in the universe.

### 4.7. Origin of Life in Zn^0^- and K^+^-Enriched, Postimpact Geothermal Systems in Relation to Other Models of Abiogenesis

The presented reconstruction implies a heterotrophic origin of life, i.e., that the first organisms were dependent on abiotically formed organic molecules and acquired the ability to synthesize them from CO_2_ much later. On this point, we agree with Oparin [586,587], Haldane [564], Horowitz [669], Bernal [153,620], Miller and his disciples [670,671,672], Calvin [119], Orgel [673], Deamer [522,549,594], Szostak [193,532,592], Maruyama [17,45], and many other great scholars not mentioned here.

The scenario proposed here is synthetic; although built on a novel idea of the abiotic generation of organic molecules in a never-before-considered natural process of Zn^0^-based hydrothermal alteration, it accommodates such elements of the earlier origin-of-life models as (i) impacts as triggers for the origin of life, see Section 3.1 and [127,128,129,145,199], (ii) volcanic systems on land as producers of organic molecules and essential elements, see Figure 5, Section 3.2, and [17,23,45,64,66,67,68,69,158,589,590,674], (iii) ionizing radiation and UV light as sources of usable energy, see Section 3.2.6 and Section 3.3.3, and [44,50,51,98,102,113,114,128,129,153,223,427,564,586,587,649,650,675,676,677], (iv) abiotic synthetic pathways starting from cyanide/formamide/ammonia formate or their homologs, see Figure 5D, Section 1.3.1 and Sections S2.3–S2.7, and [96,97,108,123,124,125,126,127,128,129,130,131,132,133,134,135,136,137,138,139,140,141,142,143,144,145,146,147,148], (v) the direct reduction of would-be biomolecules on the surfaces where they reside, see Section 3.2.6 and [458,633,634,635], (vi) the Zn-based abiotic (photo)chemistry, see Section 3.2.6, Section 3.4 and Sections S2.5 and S2.10, and [21,22,386,387,388,389,440,441,678,679,680], (vii) the (photo)selection of thermolabile but radiation-resistant ribonucleotides and their polymers, see Section 3.4 and [20,102,103,126,211,212,213,214,215,222,223], (viii) silica minerals as templates for the first oligonucleotides, see Figure 6, Section 3.3.3 and Section 3.4.2, and [147,148,153,454,455,456,457,620,681,682,683,684], (ix) the (cold) RNA World as the first manifestation of life, see Section 1.3.3 and Section 3.4, and [164,165,166,167,168,169,170,171,172,173,174,175,176,177,178,179,180,181,182,183,184,185,186,187,188,189,190,191,192,193,194,195,196,197,198,199,200,201,202,203], as well as the (x) early involvement of abiotic (proto)lipid membranes interacting with RNA molecules, see Figure 6, Section 3.4.6, and [206,446,524,530,533,536,539,550,551,552,685,686].

Curiously, the Hadean vapor condensates, rich in low-volatile associative organic compounds (see Section 3.3.1), may indeed have reached, owing to rapid water evaporation, the consistency of Haldane’s “hot, dilute soup” [564]—after being poured onto the ”plates” represented by the pools and terracettes (Figure 5).

Last but not least, there is a remarkable link with the famous sentence from Darwin’s letter to Hooker: “…But if (& oh, what a big if) we could conceive in some warm little pond with all sorts of ammonia & phosphoric salts, light, heat, electricity &c present, that a protein compound was chemically formed, ready to undergo still more complex changes” [687]. To put this quotation in a modern context, it is worth noting that “protein” (from the Greek adjective “proteios” meaning primary [688]) was used by Darwin to denote the then obscure primary molecule of life; it took a couple of decades after Darwin to distinguish between nucleic acids and actual proteins (chemically identified and named “peptides” by the aforementioned Emil Fischer [689]). It must also be noted that there were no electrical networks in Darwin’s time, so that the “electricity” known to him came from batteries. Their anodes were then made exclusively of metallic zinc, the oxidation of which provided the electron flow [690].

Amazingly, Darwin’s time “electricity” had Zn^0^ as its source. Hence, our reconstruction is consistent with Darwin’s vision in every way. However, in addition to “warm little ponds”, our scenario also includes cold distal pools and terracettes where the primary molecules of life—consortia of RNA-like molecules, as we believe,—persisted and evolved.

### 4.8. What Is Life?

This text has been written in response to an invitation to contribute to the special issue “What is Life?”. Here, and in the accompanying Supplementary File S1, we have considered the features common to all living organisms and, based on their analysis, have elaborated on the mechanism and circumstances of the origin of life. Thus, on the one hand, this text is an extended answer to the question posed. On the other hand, there is also a need for a short definition of life; however, the search for it is a thankless task, because a short definition cannot cover all aspects of life.

Nevertheless, from our analysis we can tentatively infer that life is a form of energy-driven self-organization of matter into discrete, self-assembling, self-recovering, and reproducing units that adapt to environmental changes through intrinsic heritable variability and evolve to use the available resources as efficiently as possible.

## 5. Conclusions and Outlook

The Hadean geotherms envisioned here can be seen as a chemist’s paradise on Earth. Various organic molecules must have formed in high yield as hot geothermal fluids passed through the Zn^0^- and ^40^K-enriched protocrust, see Section 3.2.6. These molecules ended up in super-reduced pools of vapor condensate with fluctuating water activity, enriched in H_2_S, NH_3_, transition metals, and oxides of molybdenum and boron, as well as semi-reduced phosphorous compounds, see Section 3.2. The ions of Zn^2+^, in addition to being potent acid-base catalysts, may have served as powerful photoreducing agents after precipitation as ZnS, see Section 1.3.4, Section 3.2.6 and Section S2.10. Potassium must have contributed both as a non-specific universal catalyst for various organic syntheses (Christophe Copéret, personal communication) and, via its ^40^K isotope, as a source of solvated electrons capable of reducing CO_2_ to organics and N_2_ to NH_3_ (see Section 1.3.1).

Not surprisingly, the most successful abiotic syntheses have been carried out under conditions compatible with those in the here envisioned post-impact geothermal systems. These promising experiments include the hydrothermal reduction of CO_2_ to formate by *Zinkstaub* with a yield of 70–80% [43,373,374], the UV photosynthesis of various organic molecules from CO_2_ by ZnS particles with a quantum yield reaching 80% for formate [379,381,382,385,387,389,691], UV-driven sugar synthesis just from formaldehyde in the formose reaction [113,114], increasing the ribose yield in the formose reaction by using borate and molybdate [115,117,596], abiotic syntheses of diverse biomolecules from cyanide [125,128,129,130,131], formamide [96,97,108,132,134,135,138,139,143,144,145,146,147], and ammonia formate [123,124], the photosynthesis/photoselection of natural nucleobases and nucleotides under UV light [102,126,143,692,693], the selective synthesis of furanosyl nucleosides from unprotected ribose and nucleobase in the presence of Zn^2+^ and NH_4_^+^ ions [441], Zn^0^/Zn^2+^-supported syntheses of all four natural nucleotides under conditions mimicking geothermal ponds [440], the exceptional non-enzymatic formation of naturally occurring 3′-5′ linkages between nucleotides in the presence of Zn^2+^ ions [443,444], and the non-enzymatic formation of long RNA oligomers (i) on clays [147,450,454,455,456,457,694], (ii) within lipid vesicles and layers [446,447,449,523,539,695], and (iii) in water-ice eutectic systems [164,165,166,167].

In light of these observations, we are confident that an experimental setup mimicking a Zn^0^- and K^+^-rich anoxic geothermal system, with the possibility of varying in a broad range the intensity and spectrum of high-energy radiation, temperature, pH, E_*h*_, and pressure, as well as the composition of the chemicals and minerals involved, would yield large amounts of organic compounds. The real problem would be to find conditions specifically conducive to the accumulation/selection of biomolecules and their productive interactions.

## Figures and Tables

**Table 1:** Molar concentration of life-relevant inorganic substances in different media. Data are compiled from refs. [41,226,227,228,229,230,231,232,233,234]. The K/Na and Zn/Fe pairs counterposed in this paper are highlighted by contrasting colors in this table and in most of the tables that follow.

Substance	Cell Cytoplasm	Blood Plasma	Today’s Sea Water	Anoxic Ocean
Na^+^	0.014	0.142	0.4	>0.4
K^+^	0.1	0.005	0.01	~0.01
Mg^2+^	0.1–0.01 (mostly bound)	0.0015	0.05	~0.01
Ca^2+^	10^−7^ ÷ 10^−6^ (free) 10^−3^ (bound)	0.002	0.01	~0.001
Fe^2+^/Fe^3+^	10^−3^ to 10^−4^	0.0015 (transferrin-bound)	10^−8^ (mostly Fe^3+^)	10^−5^ (Fe^2+^)
Zn^2+^	10^−3^ to 10^−4^	1.0–1.5 × 10^−5^	10^−8^	10^−15^ ÷ 10^−12^
Mn^2+^	10^−3^ to 10^−4^	10^−8^	10^−10^	10^−8^
Cu^+^/Cu^2+^	10^−5^ to 10^−4^	10^−5^	10^−9^ (Cu^2+^)	<10^−20^ (Cu^+^)
Mo (IV) − Mo (VI)	1.6 × 10^−7^	10^−8^	10^−7^ mostly MoO_4_^2−^ (VI)	10^−11^ to 10^−9^ mostly Mo (IV)
Cl^−^	0.15	0.1	0.5	~0.5
PO_4_^3−^/HPO_4_^2−^	~0.01 (mostly bound)	0.001	10^−6^ to 10^−9^	<10^−5^
CO_2_/HCO_3_^−^/H_2_CO_3_	0.025	0.027	0.002	0.1–0.02
S^0^/S^2−^	~10^−1^ (mostly as methionine and cysteine)	0.0005 (SO_4_^2−^)	0.026 (SO_4_^2−^)	~10^−2^ (mostly S^2−^)

**Table 3:** Concentration of some essential chemical elements in the vapor condensate of thermal springs, Mutnovsky volcano, Kamchatka peninsula (data from [23], expanded).

	Vent6-14k	Vent6-15k	Vent6-16k	Vent6-17k	Vent6-18k	Vent6-19k
T (°C) of the hot spring	94.00	93.00	89.00	93.00	96.00	96.00
pH	2.29	2.19	2.54	2.03	1.05	2.03
Cl (ppm)	9.81	10.77	5.23	4.38	10.42	1.15
Si (ppm)	n.d.	n.d.	n.d.	n.d.	n.d.	n.d.
Na (ppb hereafter)	5427	128	798	14.9	50.7	3082
K	15,787	45.5	2317	22.6	37.6	8399
B	2635	84.4	1092	185	215	4296
P	18.0	5.2	11.8	2.0	6.6	4.3
Ca	567	219	424	30.0	90.0	289
Mg	141.0	48.7	139	2.483	15.5	24.5
Fe	760	216	799	10.7	155	99.4
Zn	19.0	3.4	12.8	6.0	6.9	10.8
Mn	9.0	2.3	7.0	0.1	1.9	2.3
Cu	3.08	0.59	1.97	0.15	0.82	0.39
Ni	16.2	0.4	9.2	0.2	1.3	0.7
Mo	0.046	0.014	0.044	0.002	0.013	0.028
W	0.006	0.009	0.003	0.006	0.067	0.002

**Table 5:** Chemical composition of fumarolic condensates of volcano Kudryavy. Data are taken from [346].

Sampled Fumarole #	F1292	F292	F1392	F592
T °C	940	825	705	535
pH	1.01	0.67	1.38	0.78
Cl (ppm)	8580	12,850	2070	13,580
Si (ppm)	164	510	105	460
Na (ppb hereafter)	6100	5000	2560	2440
K	5620	9530	20,500	15,500
B	33,000	4200	17,540	35,300
P	30,800	2000	4000	22,700
Ca	2000	1730	14,300	2300
Mg	500	880	4300	120
Fe	7300	3620	17,650	2280
Zn	3100	1000	13,500	250
Mn	90	55	180	17
Cu	270	90	910	76
Ni	410	320	50	50
Mo	200	110	160	2
W	30	20	60	3

**Table 6:** Life paradoxes and their tentative solutions (see Sections S2.1–S2.12 of the Supplementary File S1 for a detailed consideration of each paradox).

Nr.	Paradox	Novel/Complementary Solutions Provided by Here Proposed Scenario
1.	Super-reduced state of organic molecules	Reduction of CO_2_ to organic molecules by (i) up to 10^19^ kg of metallic Zn within the post-impact protocrust, (ii) other reducing agents present in the super-reduced protocrust, such as carbides, and (iii) powerful electron donors present in super-reduced geothermal pools, see Section 3.2.6.
2.	The tar paradox	Demonstrated ability of formamide to extract nitrogen-containing compounds from tar, see Section 3.3.2.
3.	Paradox of the CN bonds	Delivery of ammonia by very hot fumaroles and Zn^2+^ and ZnS (photo)catalyzed amination of organic molecules, see Section 3.2.2 and Section 3.3.2.
4.	Water paradox	Rapid evaporation of water in frost and at low atmospheric pressure leaving non-freezing pools filled by low-volatile associative organic compounds, see Section 3.3.1.
5.	Chicken and egg paradox of the first biopolymer?	Particular suitability of cold Hadean geothermal pools for the Zn- and K-mediated emergence of RNA World, see Section 3.4.
6.	Paradox of exclusive photostability of canonical nucleotides	This exclusive photostability may have enabled (i) boosting the functional activity of oligoribonucleotides and their consortia by their UV heating at low temperatures, and (ii) accumulation of photostable compounds by selective elimination of photolabile organic molecules, see Section 3.3.4 and Section 3.4.4.
7.	The complexity paradox	Complexity may have been achieved owing to the photoselection of oligoribonucleotides capable of Zn- and K-mediated folding into tightly packed consortia rich in particularly photostable double-helical segments, which may have eventually led to self-copying RNA consortia, see Section 3.4.
8.	A ten-fold predominance of K^+^ over Na^+^ in the cell interior	The generally high K^+^/Na^+^ ratio in the geothermal vapor was specifically amplified by the enrichment of the top layer of the post-impact fallout by ~3.0 × 10^19^ kg of K, see Section 3.1.5.
9.	High intracellular Mg^2+^/Ca^2+^ ratio	Hadean reservoirs must have had low concentrations of Ca^2+^ ions due to their removal as carbonates at CO_2_ levels 10–100 times higher than today, see Section S2.9.
10.	Almost exclusive use of Zn^2+^ ions as transition metal cofactors by evolutionarily oldest proteins and RNAs	The uppermost layer of the post-impact fallout must have contained up to 10^19^ kg of mostly metallic Zn, prone to reduce CO_2_ and N_2_ to organic molecules under hydrothermal conditions. The released Zn^2+^ ions must have been continuously delivered by venting fluids to “habitable” geothermal pools to be recruited by RNAs and proteins as cofactors, see Section 3.2 and Section 3.3.
11.	Paradox of very low levels of poorly soluble phosphate in today’s environments	The amount of semi-reduced phosphorus compounds in the vapor of very hot fumaroles, fed by magmatic plumes with t° ≥ 1300 °C, is a thousand times greater than their amount in the vapor of cooler thermal springs. Such very hot fumaroles are rare today but must have been widespread in the Hadean providing ample amounts of soluble phosphorus species, see Section 3.2.2.
12.	Paradox of the faint young Sun and the frosty climate at the beginning of the Hadean.	We see no paradox here. It must have been cold at the beginning of the Hadean, but this could not have precluded the origin of life in anoxic geothermal fields around volcanoes, see Section 3.2 and Section 3.3.

## Data Availability

All the relevant data are provided as Supplementary Files S2 and S3.

## Supplementary Materials

The following supporting information can be downloaded at: https://www.mdpi.com/article/10.3390/life15030399/s1, Supplementary File S1: Life and its Paradoxes; Supplementary File S2: Inorganic Constituents of Ubiquitous Proteins; Supplementary File S3: Thermodynamic Calculation of the Condensation of a Gas with the Composition of the Bulk Silicate Earth.
