# Supplementary material for: Origin of the RNA World in Cold Hadean Geothermal Fields Enriched in Zinc and Potassium: Abiogenesis as a Positive Fallout from the Moon-Forming Impact?
Source: Life (Basel). 2025 Mar 4;15(3):399. doi: 10.3390/life15030399 (PMC11943819; doi:10.3390/life15030399)
Supplement: Supplementary file 1 [file life-15-00399-s001.zip › Manuscript ID life-3429573_Supplementary File 1.pdf]

Origin of the RNA World in cold Hadean geothermal fields enriched in zinc and potassium: Abiogenesis as a positive fallout from the Moon-forming impact?

by A.Y. Mulkidjanian, D.V.Dibrova and A.Y. Bychkov.

Supplementary File 1

Life and its Paradoxes

S1. Basic features of life.

S1.1. Biomolecules: RNA, DNA, proteins, sugars, and lipids.

Although living organisms contain diverse biomolecules, the key players of life - as we know it - are polymers of three types: ribonucleic acids (RNAs), deoxyribonucleic acids (DNAs) and proteins (Figure S1.1). Long DNA molecules store genetic information as strings of nucleotides, RNA molecules help translate information into protein amino acid sequences, and proteins do most of the work in the cell [1].

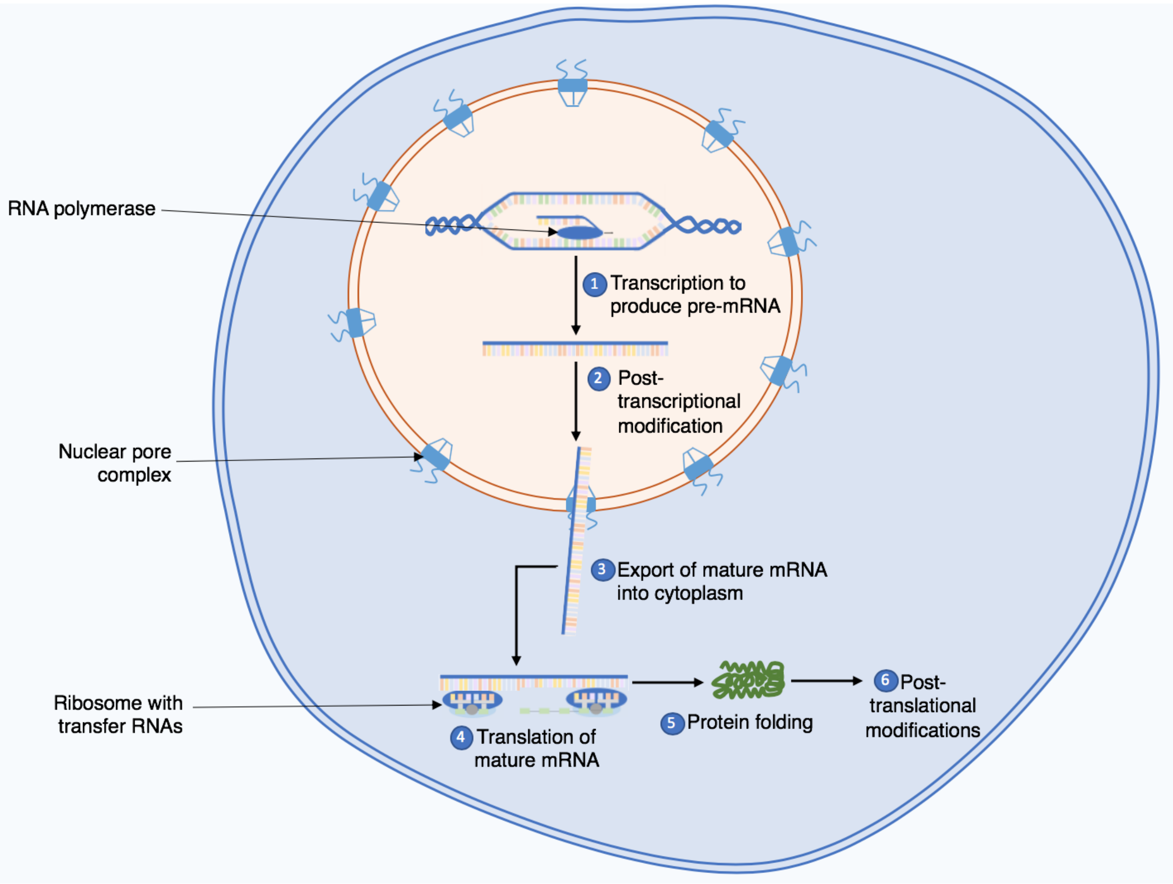

Figure S1.1. Relation between DNA, RNA, and proteins in a cell. Image credit: Kep17, Wikipedia.

RNA molecules are made up of ribonucleotides - rather complex units consisting of a nucleobase, a ribose sugar and one or more phosphate groups, see Figure S1.2. Nucleobases are one- or two-ring moieties of alternating carbon and nitrogen atoms; because of the high nitrogen content, nucleobases are sometimes called nitrogenous bases or nitrogen bases.

There are four major nucleobases in RNA, the purines adenine (A) and guanine (G), as well as the pyrimidines cytosine (C) and uracil (U), all shown in Figure S1.2. Nucleobases are attached to ribose units, which have a ring-like structure. When nucleotides join together to form an RNA molecule, phosphate groups link the ribose units, so that phosphate groups alternate with ribose units to which nucleobases are attached (Figure S1.2A).

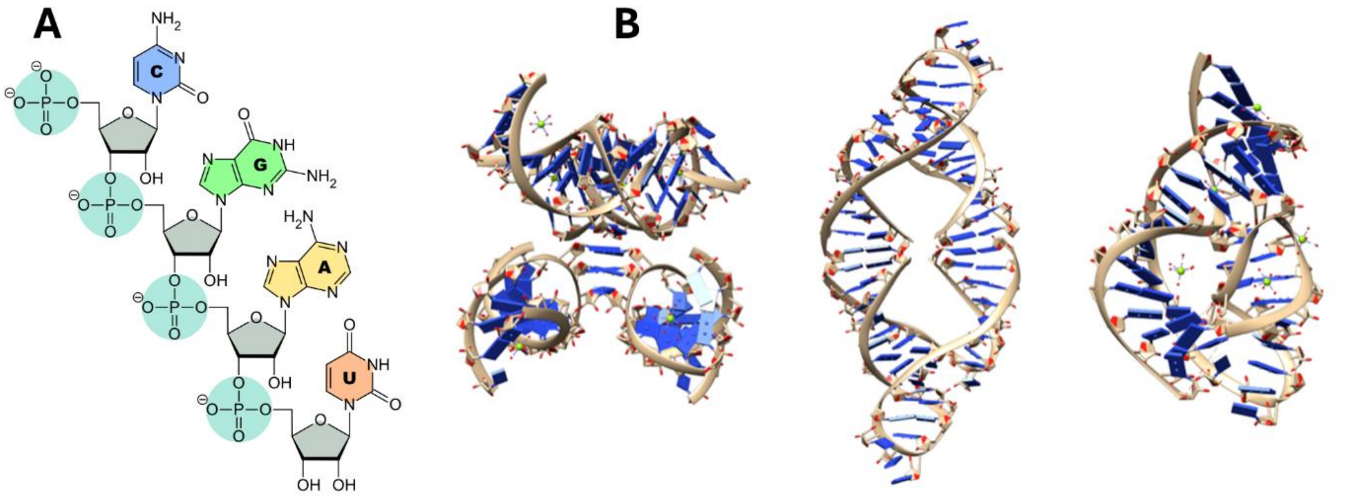

Figure S1.2. RNA. A, Structure of an RNA segment, image credit: Sponk, Wikipedia; B, RNA molecules with catalytic activity (ribozymes), from left to right: leadzyme, hammerhead ribozyme, twister ribozyme; image credit: Lucasharr, Wikipedia

The formation of polynucleotides in a so-called condensation reaction is accompanied by the release of water molecules (Figure S1.3).

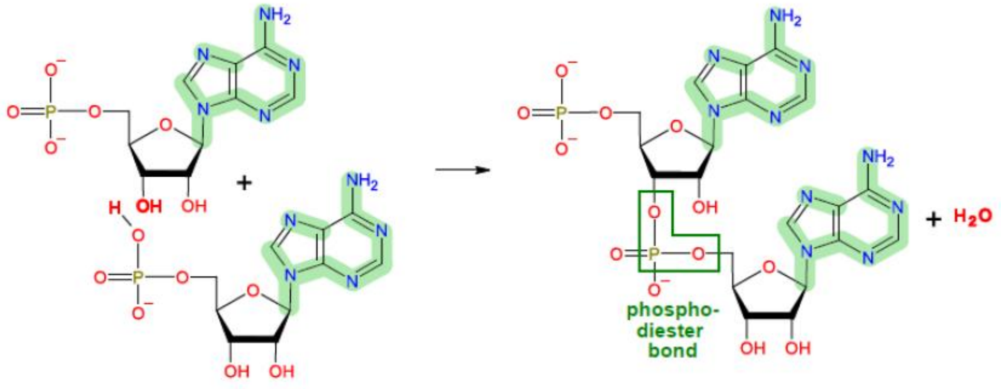

Figure S1.3. Polymerization (condensation) of ribonucleotides

In addition to being the building blocks of RNA, ribonucleotides in their triphosphate forms store energy in the cell, with adenosine triphosphate (ATP) being the most abundant energy-storing moiety, see Figure S1.4. The cleavage of their phosphate groups is accompanied by the release of free energy that can be used by specific enzymes, in particular to perform mechanical work [2,3].

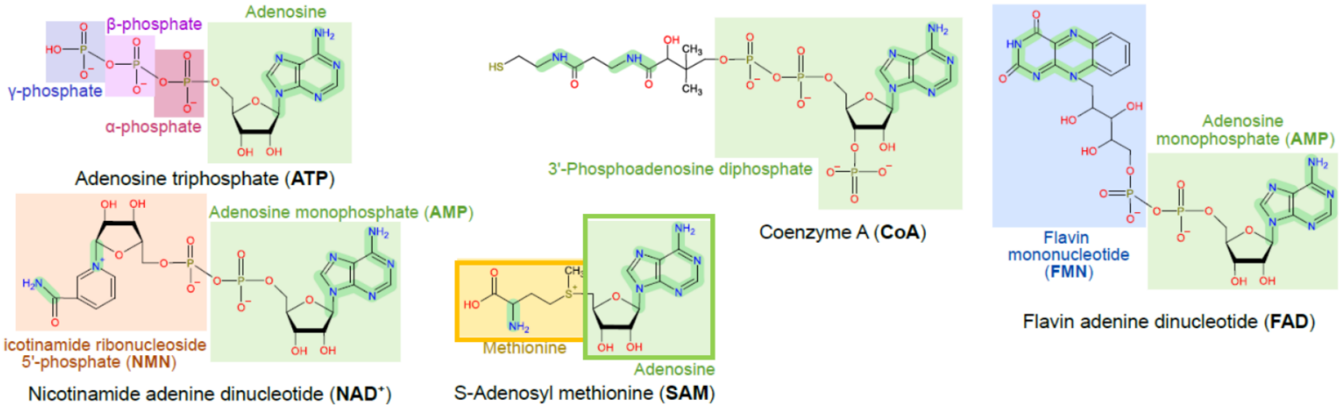

Figure S1.4. Nucleotide-containing cofactors

DNA differs from RNA in that it contains deoxyribose as the sugar moiety; deoxyribose has one oxygen atom less than ribose, which makes the DNA polymers less flexible than RNA molecules but 200 times more stable against occasional backbone breaks caused by water (hydrolysis, see [4,5]). In addition, DNA contains thymine (T) instead of the chemically similar uracil (U), see Figure S1.5.

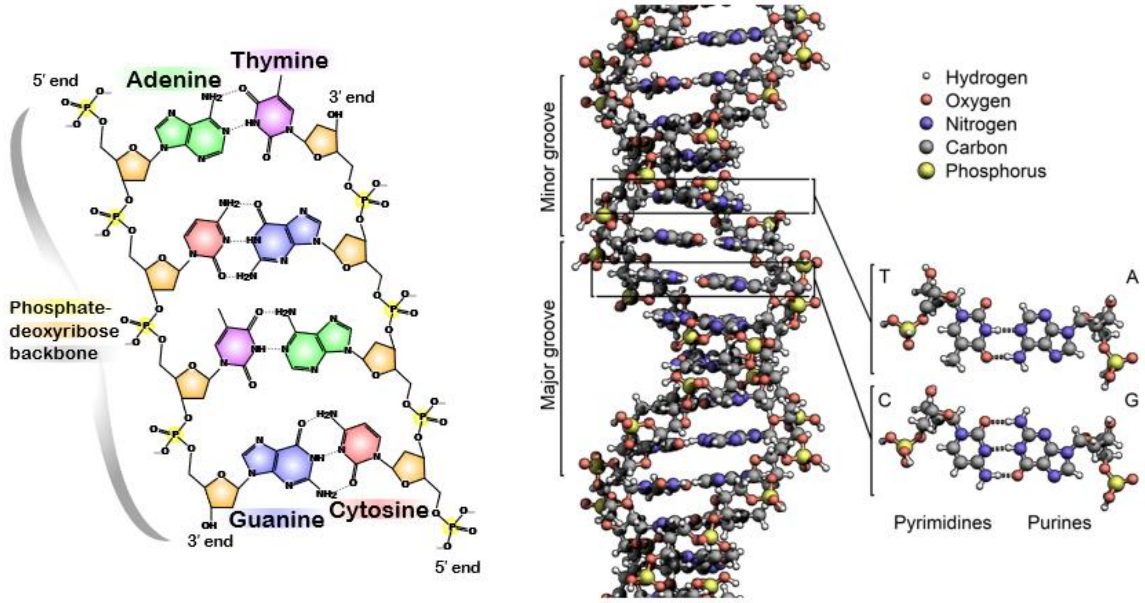

Figure S1.5. Structure of a double-strand segment of DNA. Left: chemical structure; right, ball-and-stick presentation. Image credits: Madprime and Zephyris, respectively, Wikipedia

Nucleobases can form so-called Watson-Crick pairs stabilized by hydrogen bonds (H-bonds) in which protons link oxygen or nitrogen atoms of two apposed nucleotides, see Figure S1.2, S1.5. and [6]. Adenine pairs with uracil (A-U, in RNA) or thymine (A-T, in DNA), while guanine “recognizes” cytosine (G-C, in both RNA and DNA); these pairs are called complementary bases. Multiple Watson-Crick interactions between chains of complementary nucleotides usually yield double helices. They are stabilized by H-bonds between the bases of opposite chains and stacking interactions between the bases of the same chain (Figure S1.2, S1.5.).

Rather inflexible DNA molecules usually exist as long double helices (Figure S1.5.). In contrast, RNA molecules are more versatile. They also form long double helices while storing genomic information, as in RNA viruses. RNA molecules, however, can also fold into compact structures formed by many short, intertwined double helices. Some of these structures are catalytically active, they are called ribozymes, see Figure S1.2B.

As the cell reproduces, the double helix of a DNA molecule (or RNA in some viruses that have no DNA) opens and a chain of complementary nucleotides lines up along each original chain,

resulting in the formation of two similar double helices of DNA; this process is called replication, see Figure S1.6.

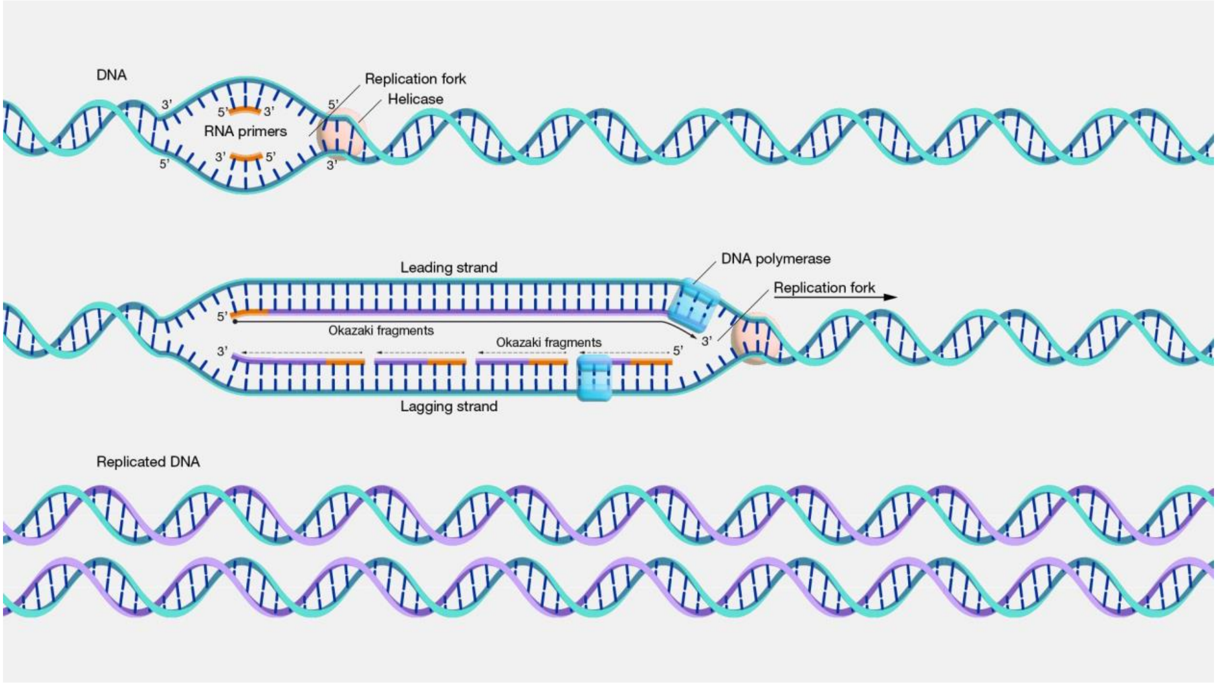

Figure S1.6. Replication scheme. Image credit: NIH (https://www.genome.gov/genetics-glossary/DNA-Replication)

Currently, replication is accomplished by proteins. In general, proteins do almost all the hard work in the cell, from building cellular structures to catalyzing (speeding up) chemical reactions. As shown in Figure S1.7, proteins are made up of amino acids, of which there are 20 ubiquitous ones. Amino acids are small molecules with an amino group at one end and an acid group at the other. Each time one amino acid binds to another, the amino group of one amino acid interacts with the acid group of another amino acid to form a new carbon-nitrogen (CN) bond (peptide bond); this reaction is accompanied by the release of a water molecule (Figure S1.7). A chain of peptide bonds forms the backbone from which the side chains of the amino acids extend. The side chains vary in size, polarity, electrical charge, presence of aromatic groups, etc. This chemical and structural diversity of amino acid side chains, as well as variations in protein sequences, accounts for the diversity of proteins and their functions.

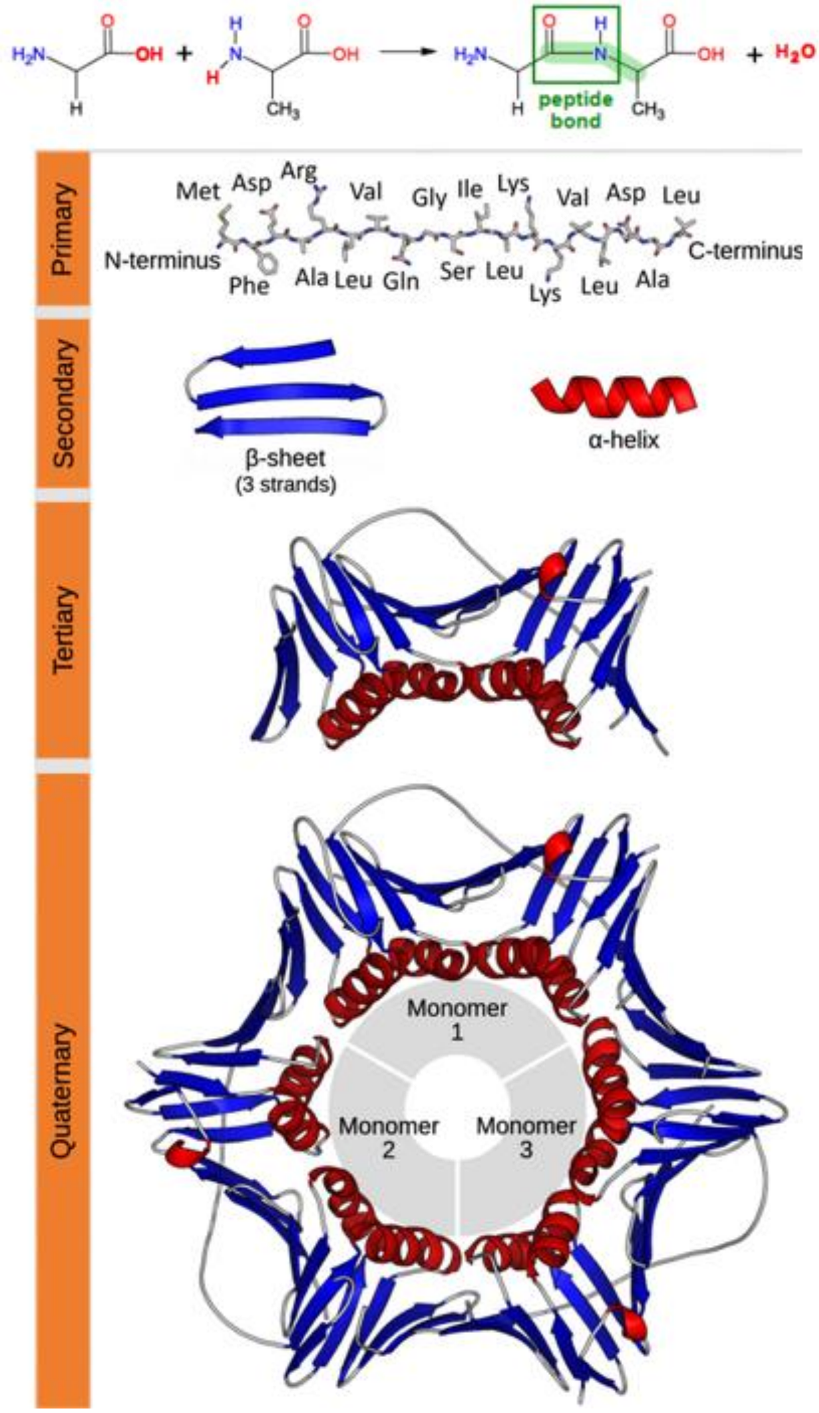

Figure S1.7. Proteins. Top, formation of a peptide bond; bottom, the four levels of protein structure. Image credit: Thomas Shafee, Wikipedia

In many cases, enzymes are assisted by so-called cofactors. These are either ions of metals such as zinc, iron, cobalt, manganese, magnesium, potassium, or complex organic molecules, or combinations of both. Cofactors help to accelerate chemical reactions that cannot be accelerated by the amino acid side chains alone. Remarkably, many ubiquitous organic

cofactors, in addition to their “catalytic” parts, also contain nucleotide moieties, the function of which remains obscure, see some examples in Figure S1.4.

Although nucleic acids and proteins have quite different structures, they share a common construction principle. In both cases, the homopolymer backbone is built of identical units (sugar-phosphate units and peptide groups, respectively) whereas the variability is owing to the dissimilarity of the groups that are attached to the backbone (nucleobases and amino acid residues, respectively), cf Figures S1.2, S1.5, and S1.7.

Sugar molecules, in addition to being constituents of nucleotides (Figures S1.2 – S.1.5.), can join into polysaccharides, which form a protective coating on the surface of cells (Figure S1.8). Polysaccharides are also used by cells to as food preservatives.

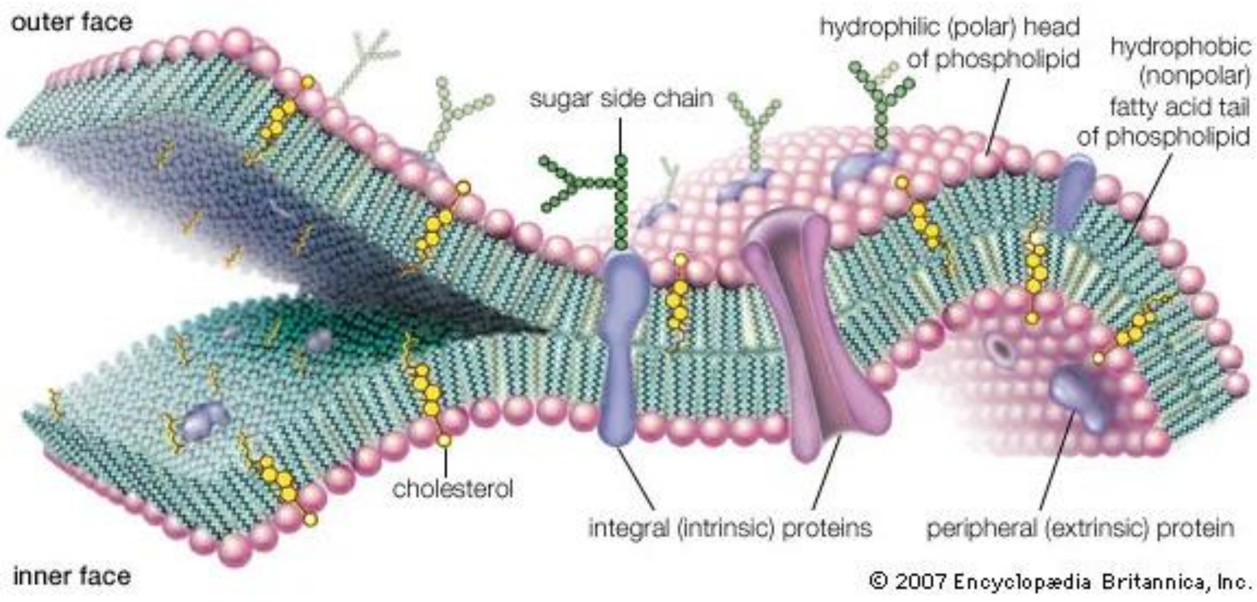

Figure S1.8. Biological membrane. Shown are two layers of amphiphilic lipid molecules stabilized by hydrophobic interactions between the “fatty” tails, integral membrane proteins, and sugar chains attached to some of membrane proteins. Image credit: Encyclopedia Britannica.

Amphiphilic lipid molecules are another important component of living systems. Because of their polar heads and hydrophobic tails, they can form bilayer membranes that can serve as barriers for polar molecules and ions. Such lipid bilayers form the core of the cell membranes

(Figures S1.8. and S1.9.).

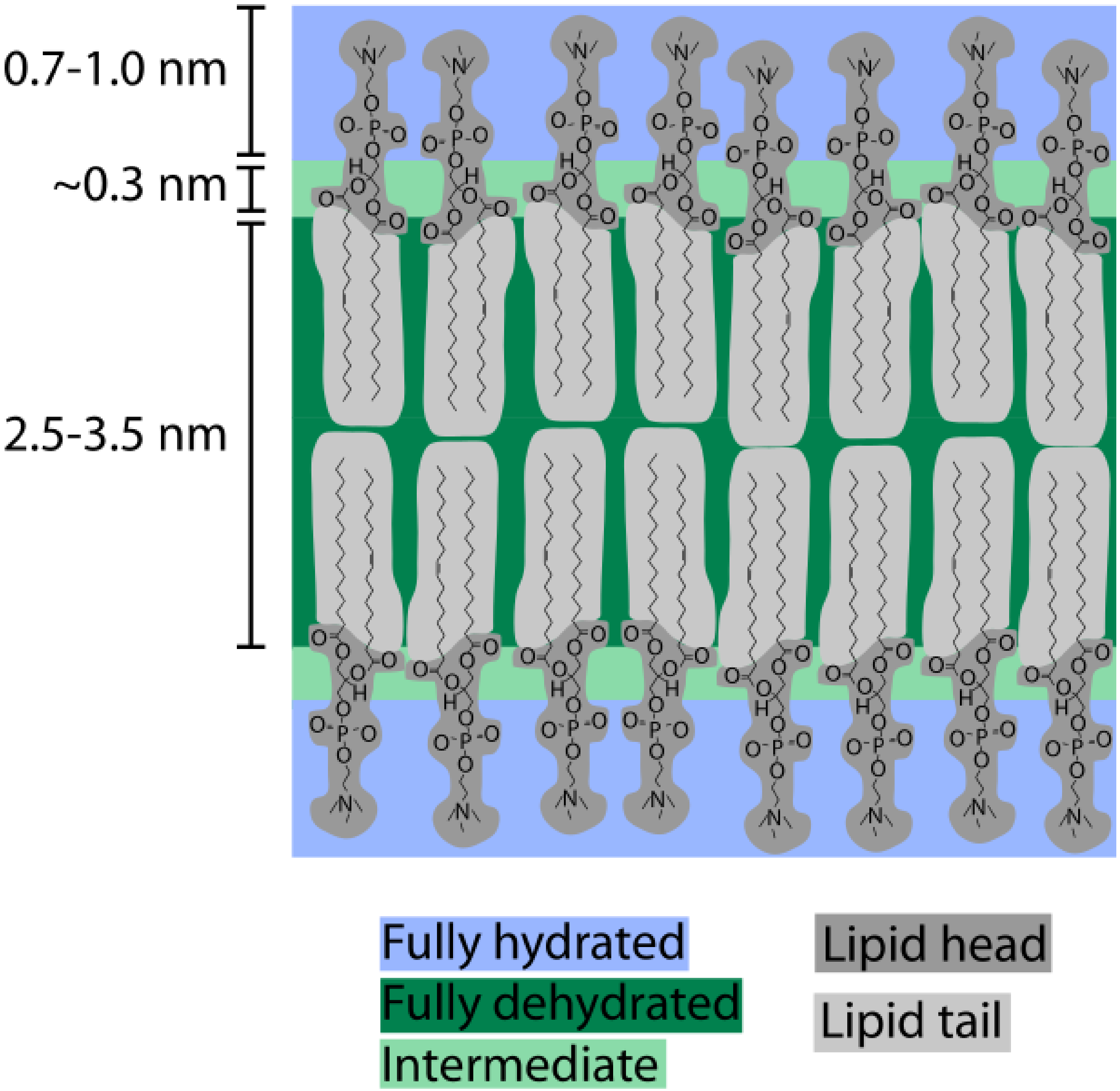

Figure S1.9. Schematic presentation of a lipid bilayer (Image credit: MDougM and Chiswick Chap, Wikipedia).

S1.2. Protein synthesis - translation

DNA, RNA and proteins are coupled via the genetic code, which assignes triplets of nucleotides to amino acids; the information stored in a four-letter nucleotide code is “translated” into the 20-letter amino acid alphabet (Figure S1.10).

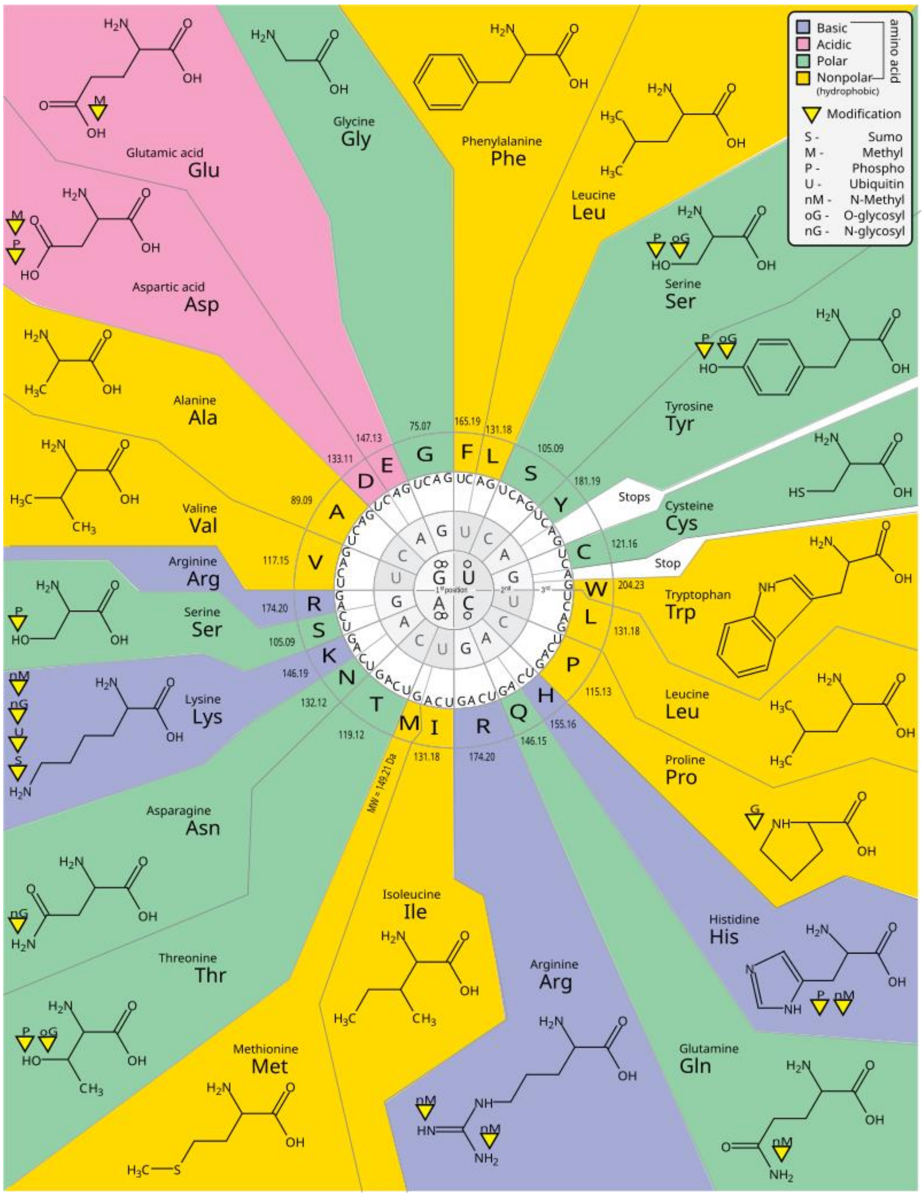

Figure S1.10. The genetic code. Image credit: Kosigrim, Wikipedia

As shown in Figure S1.1, to enable protein synthesis, the double helix of DNA opens and a special enzyme complex - called RNA polymerase – gains access to the gene-encoding strand of DNA, aligns complementary ribonucleotides along it, and links them into an RNA polymer called messenger RNA (mRNA). This process is called transcription.

After detaching from the DNA strand, the mRNA molecule can attract the large and small ribosomal subunits (LRS and SRS, respectively). Both ribosomal subunits are formed by long but tightly folded ribosomal RNA (rRNA) molecules intertwined with protein molecules [7,8].

The LRS and SRS eventually "clamp" over the mRNA with the formation of a full-fledged ribosome, the protein synthesis machine (Figure S1.11.). Ribosomes slide along the mRNA and synthesize proteins from amino acids.

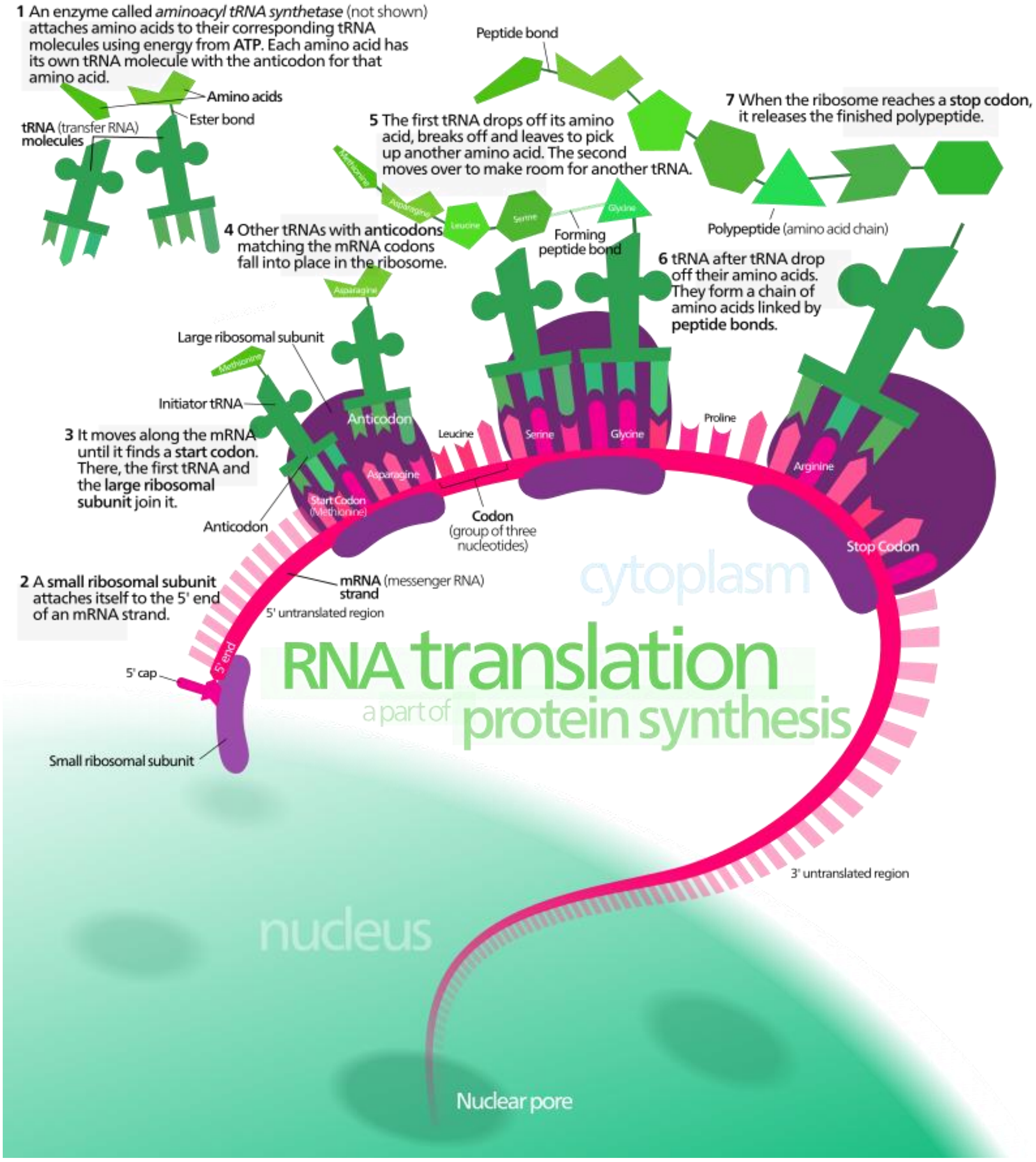

Figure S1.11., Overview of translation. Image credit: Kelvinsong, Wikipedia

Each amino acid is delivered by its specific small transfer RNA (tRNA), which carries a nucleotide triplet complementary to the one encoding this amino acid on the mRNA. Only when this triplet properly docks to the complementary triplet on the mRNA is the amino acid attached to the nascent protein chain (Figure S1.10). In sum, the SRS is responsible for the interaction with mRNA and decoding, while the LRS catalyzes the formation of peptide bonds; tRNAs interact with both subunits and functionally link them. Since the four-letter language of DNA and RNA is converted into the twenty-letter language of proteins, this process is called translation.

S1.3. Ability of biomolecules to self-assemble and self-recover

The biomolecules described in the previous section share the ability to self-assemble and self-recover. For example, heating a folded RNA molecule would result in its denaturation and loss of a particular (native) structure. However, upon gradual, slow cooling, the RNA molecule will recover its original structure, see Fig. S1.12 and [9]. This native structure is characterized by the maximum number of thermodynamically favorable interactions between the nucleotides; thus, upon recovery, biopolymers tend to reach the lowest energy state.

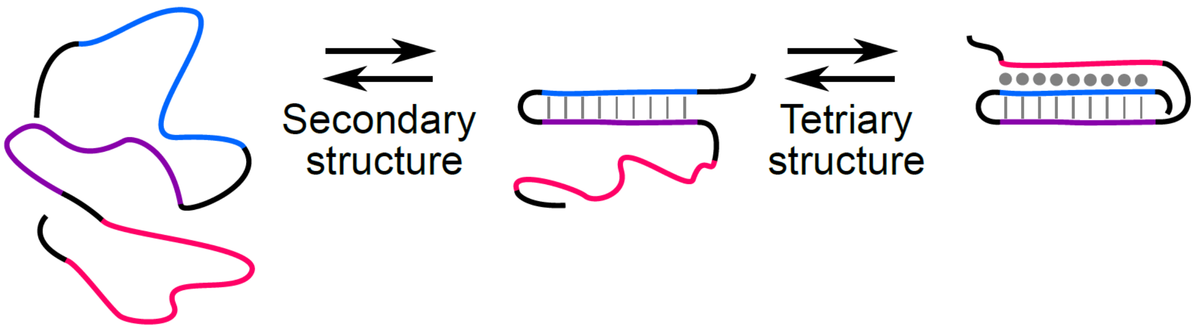

Figure S1.12. Schematic presentation of a stepwise self-assembly (folding) of an RNA molecule (redrawn with modifications from [10])

Short polymers of sugars (oligosaccharides) are also capable of self-assembly [11]; however, this area is not well explored.

Much better understood is the self-assembly of lipids. They tend to assemble into structures in which the hydrophobic tails interact with each other, while the polar heads face the water phase. In particular, when the lipids involved have an approximately cylindrical shape, they attain a bilayer structure (see Figure S1.9) and [12].

Most - but by no means all - proteins also tend to fold into their thermodynamically most stable 'native' structures. However, the stabilization of a protein globule (see Figure S1.7) involves more diverse interactions than in the case of self-assembling DNA, RNA, or lipids. Therefore, special protein complexes called chaperones often help other proteins to fold.

The ability to self-assemble is inherent not only in individual biopolymers but also in their complexes. For example, ribosomes, shown in Figure S1.11, self-assemble from a few large RNA molecules and numerous proteins.

The peculiarity of all the self-assembling systems considered is that they become disordered as the temperature rises from physiological levels. The double-helical regions of DNA and RNA unwind, proteins unfold, and membrane bilayers become less ordered and eventually disintegrate. This is because the contribution of the entropy factor $-T\Delta S$ to the Gibbs equation ($\Delta G = \Delta H - T\Delta S$, see [2]) increases with temperature, favoring disorder. The flip side of this peculiarity is that decreasing the temperature from physiological levels additionally stabilizes such self-assembling systems.

S1.4. Cellular and acellular life forms and the nature of the Last Universal Cellular Ancestor (LUCA).

Living organisms can be either cellular or acellular (Fig. S1.13). Cellular organisms include eukaryotes (e.g. plants and animals) and prokaryotes (e.g. bacteria). Eukaryotes have larger cells with a separate nucleus that preserves DNA. The smaller prokaryotes have no nuclei, their DNA is usually spread in the cell.

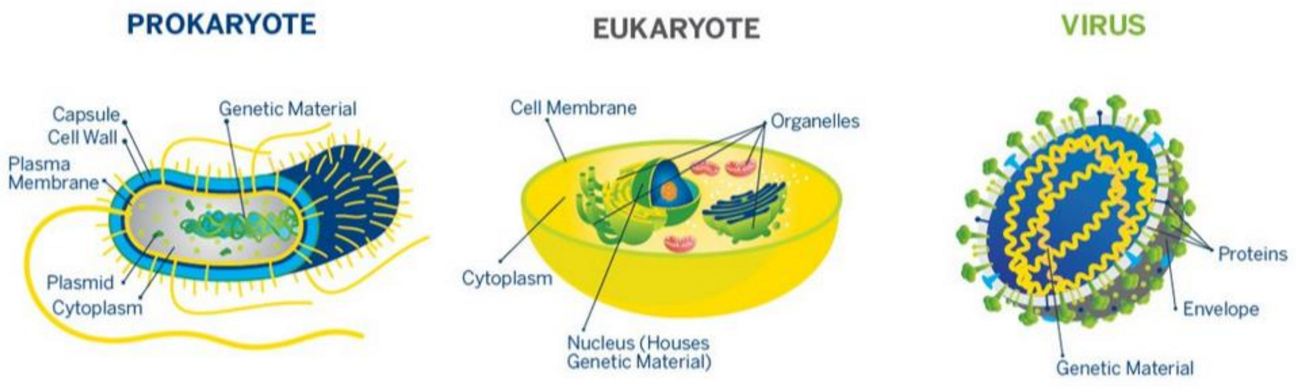

Figure S1.13. Diverse Life forms. Image credit: Webpage of bioMérieux, https://www.biomerieux.com/nl/en/blog/infectious-diseases/what-are-viruses-and-how-do-they-infect-us--.html.

Acellular life forms are represented by viruses and viroids. While cellular organisms synthesize nucleic acids and proteins themselves, viruses and viroids hijack cells and force them to perform these syntheses for them.

Most viruses are containers of tightly packed nucleic acids and proteins. Such a "package" is encased by either a protein capsid or a lipid membrane, usually "borrowed" from the previous viral host. Upon viral attack, the "package" is injected into the cell and reprograms its biosynthetic machinery to produce new viruses.

Viroids appear to be the simplest forms of life. They are circular, single-stranded RNA molecules without a shell of any kind. Once inside the cell, they (somehow) force the cell to provide for their reproduction.

The common origin of cellular life forms was clarified by Carl Woese and his colleagues, who compared the nucleotide sequences of the small ribosomal subunits (SRS) of very different organisms from all "kingdoms of life" and found that they were similar, although to different extents [13,14]. According to the tree of life based on the comparison of SRSs (Figure S1. 14), all cellular organisms belong to either the Bacteria, or to the Archaea, or to the Eukarya. Archaea, like Bacteria, are prokaryotes, but their machinery for reproduction and protein

synthesis is similar to that of Eukarya. It is thought that the first bacteria diverged from the

ancestors of the Archaea/Eukarya, followed by the divergence of the Eukarya from the Archaea lineage. Woese defined the universal ancestor at the root of the tree of life as the "progenote" [15].

More recently, it has been found that the ancestors of eukaryotes are in fact closely related to the recently characterized so-called Asgard Archaea. Apparently, the archaeal ancestors of eukaryotes underwent multiple symbioses/fusions with different bacteria before becoming fully complex eukaryotic cells, see Figure S1.14 and [16,17].

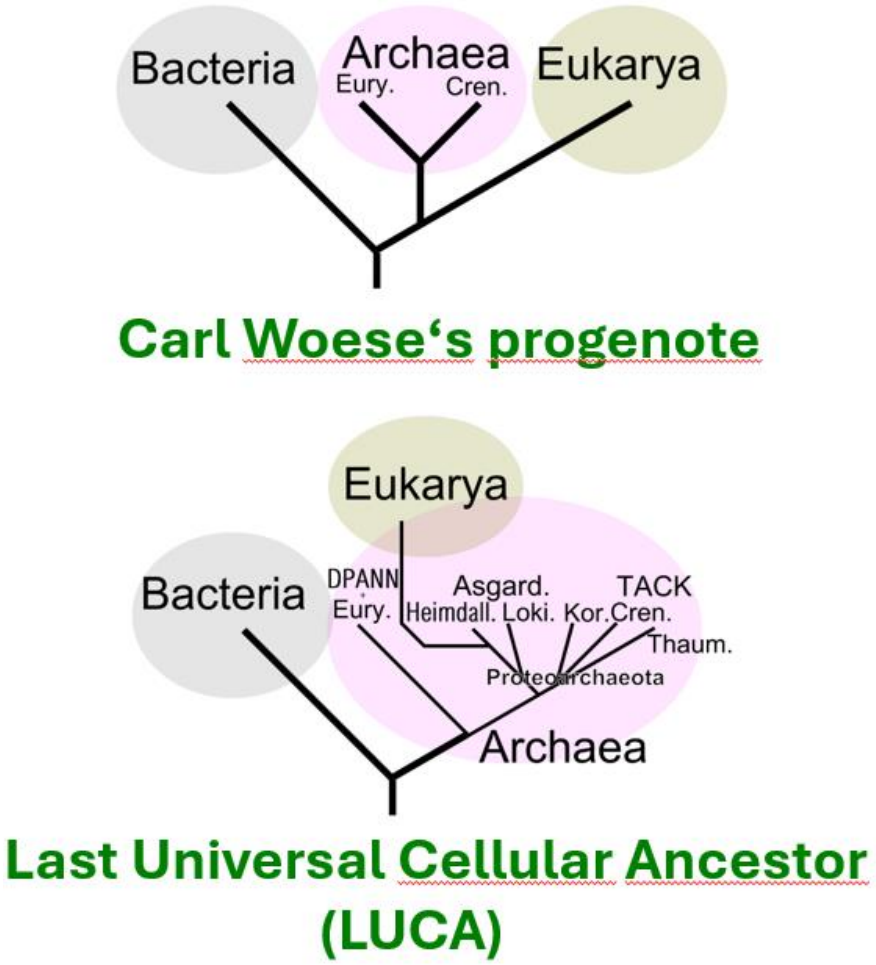

S1.14. The three-domain tree of life according to Woese [18,19] and the currently accepted two-domain tree [16]. Image credit: Crion, Wikipedia (with modifications).

Archaea and Bacteria fundamentally differ in many respects [20-23]. In particular, they have different membrane lipids [24]. Bacterial lipids are built of fatty acids connected to a glycerol-3-phosphate moiety via ester bonds (Figures S1.12 and S1.15., top), whereas archaeal membrane

lipids are composed of branched isoprenoid tails ether-linked to a glycerol-1-phosphate moiety, an optical isomer of glycerol-3-phosphate (Figure S1.15., bottom).

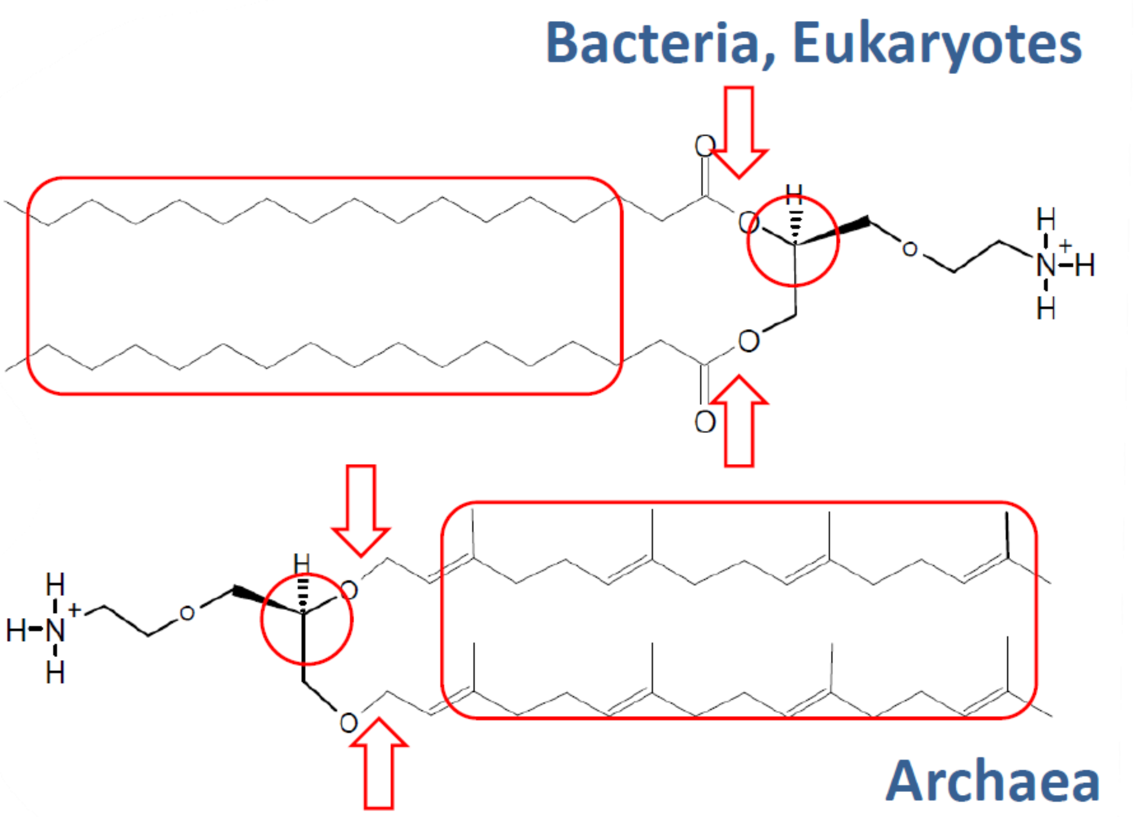

Figure S1.15. Organization of two-tail phospholipids in Bacteria/Eukarya (top, phosphatidylcholine) and Archaea (right, diphytanylglycerol ether analog of phosphatidylcholine), see also [25]. Differences in chemical organization are indicated by arrows.

The nature of the "progenote", later called the Last Universal Cellular (or Common) Ancestor (LUCA) [26,27], was specified after the decoding of the first complete genomes and the identification of those genes that are present in all free-living cellular organisms and are therefore present in the LUCA. This set is small; it contains about 100 protein- and RNA-coding genes [20-22]. Three quarters of these genes encode components of the protein synthesis machinery. It turned out that the LUCA can be defined by its ability to synthesize proteins [23].

Remarkably, DNA is not directly involved in protein synthesis; all the work is done by RNAs and proteins. It should also be noted that the apparently simple reaction of converting a ribonucleotide into a deoxyribonucleotide by removing an oxygen atom from a sugar residue (see Figures S1.2 and S1.5) is actually a complex transformation carried out by enzymes that are different in bacteria and archaea. The DNA processing enzymes involved in transcription and replication (see Figures S1.1 and S1.6) are also different in bacteria and archaea [20-23]. Therefore, it is widely believed that DNA, as a more stable information storage medium than RNA, emerged after the appearance of RNAs and proteins. Consequently, it has been suggested that LUCA may have been an RNA/protein organism, and that bacteria and archaea/eucaryotes mastered the use of DNA separately [23]. Alternatively, LUCA could have already had a primitive DNA genome and could have processed DNA with adapted versions of RNA-processing enzymes [28]; these "provisional" enzymes could have been independently replaced by more specialized DNA-tailored enzymes in bacteria and archaea, respectively, after their separation.

The fundamental difference between the membrane lipids of bacteria and archaea makes the nature of the membrane lipids of LUCA unclear. It has been suggested that LUCA may have had primitive single-tail lipids [29-31], but the chemical nature of the tails or heads of LUCA lipids remains unclear.

It is possible to imagine the LUCA as a community of interdependent organisms. Each organism was able to synthesize some proteins and metabolites but could not synthesize everything it needed. Therefore, it shared some of the synthesized proteins and metabolites by expelling them from the cell and taking the necessary ones synthesized by other organisms. Such interdependence among organisms is also typical of modern life.

S2. Paradoxes of Life

Life has many paradoxical features. It is believed that resolving these paradoxes may help to unravel the circumstances of the origin of life [32-35]. Most of these paradoxes are known to experts in the field. However, for those readers who are not deeply involved in origins of life research, we will review some of the paradoxes of life here. We move from paradoxes involving the simplest organic molecules to paradoxes involving more complex structures and cells.

In most cases, we are not the first to identify these paradoxes of nature, so we also review the solutions that have been proposed for them (if they have been proposed at all). In this way, we simultaneously present the main scientifically plausible ideas about the origin of life.

S2.1. Paradox of the super-reduced state of organic molecules.

Organic molecules are so called because they are found in organisms and are rare in inorganic nature. In organic molecules, carbon atoms are bonded to hydrogen atoms and to a lesser extent, if at all, to oxygen atoms. Therefore, these molecules are considered to be reduced in oxygen. The reducing power of organic molecules usually decreases when their hydrogen atoms are replaced by oxygen atoms; this process is called oxidation.

In chemistry, reducing power is characterized by the redox potential, which is defined as [2]:

$$\boldsymbol{E = E_0 - 2.3\,\frac{RT}{nF} log_{10}\frac{[red]}{[ox]} = E_0 - \frac{59}{n} log_{10}\frac{[red]}{[ox]}} \qquad \text{(S1)}$$

where *R* is the universal gas constant, *T* is the absolute temperature, *F* is the Faraday constant, *n* is the number of electrons transferred, and E_0 is the “standard” redox potential determined at equal concentrations of the reduced and oxidized forms when the last term becomes zero. According to Equation (S1), the redox potential depends on the concentration of the reactants and decreases with increasing concentration of the reduced form.

Redox potentials are defined relative to the potential of the so-called hydrogen electrode, a platinum plate at which protons of water can be reduced to molecular hydrogen (H_2) in a reaction:

$2H^+$ (in water) + $2e^-$ ↔ H_2 (gas) (S2)

where e^- denotes an electron. The E_0 value of this reaction at pH 0.0, 25ºC, and 1 atm pressure of H_2 is taken as 0 mV, provided that water is used as a solvent.

This equation can also be written as

$2H_2O$ (liquid) + $2e^-$ ↔ H_2 (gas) + $2OH^-$ (in water) (S3)

Redox potentials of reactions involving protons are pH dependent. For redox reactions in which the number of electrons transferred is equal to the number of protons transferred, a simplified relation can be used:

$$\boldsymbol{E = E_0 - 59 \times pH} \qquad \text{(S4)}$$

Hence, at pH 7.0, the E_0^7 value for the hydrogen electrode is –59 mV × 7 = – 413 mV. The redox potential of the hydrogen electrode is considered as the low-potential limit of water stability; reducing agents with even lower redox potentials can decompose water into H_2 and OH^- anions according to Equation (S3).

Another important redox reaction is the decomposition of water at very high potentials:

O_2 (gas) + $4H^+$ (in water) + $4e^-$ = $2H_2O$ (in water) **(S5)**

The E_0^7 value of this reaction is +820 mV.

Figure S2.1. shows typical organic and inorganic redox half-reactions (redox pairs) with the values of their standard redox potentials at pH value of 7.0 (E_0^7) which is considered as standard in the biochemical literature.

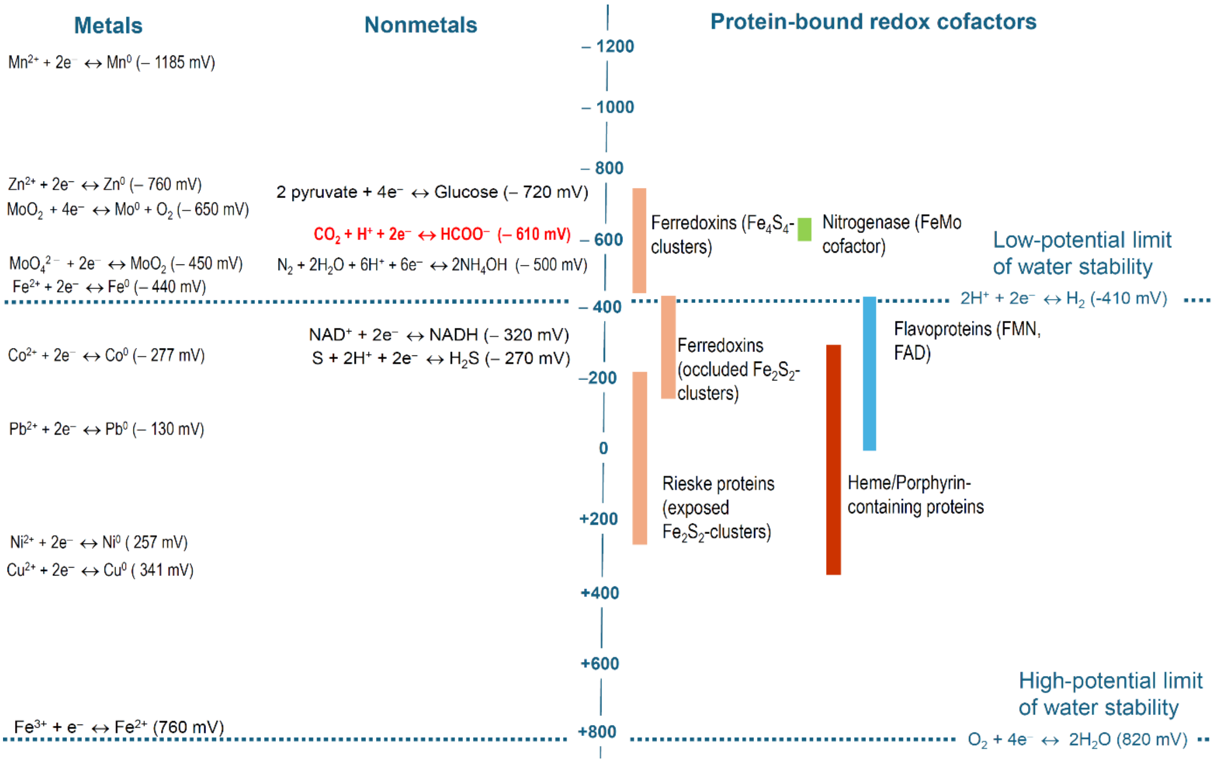

Figure S2.1. Redox potentials and oxygen fugacity *f*(O_2). **A**, Redox potentials of biologically relevant redox reactions. Left, biologically relevant half-reactions; right, operative redox ranges of main redox cofactors. The difference between the redox potentials of two half-reactions corresponds to the free energy of the redox reaction between them. Spontaneous electron transfer occurs when the redox potential of the electron-donating half-reaction is more negative than that of the electron-receiving half-reaction. The plot is based on data compiled from [36-41]. **B**, Stepwise reduction of CO_2 to methane, modified from [39]. **C,** *f*(O_2)-temperature diagram. Log oxygen fugacity vs temperature at 1 bar pressure for common buffer assemblages, plotted using algorithms compiled by B. R. Frost [42]. The FMQ (fayalite-magnetite-quartz) buffer is characterized by the reaction $3Fe^{2+}{}_2SiO_4 + O_2 \leftrightarrow 2Fe^{3+}{}_3O_4 + 3SiO_2$, the IW (iron-wustite)redox buffer by the reaction, $[2(1\text{-}x)Fe^0 + O_2 \leftrightarrow 2Fe^{2+}(1\text{-}x)O]$, the IQF (iron-quartz-fayalite) by the reaction $2Fe^0 + SiO_2 + O_2 \leftrightarrow Fe^{2+}{}_2SiO_4$ whereas the Zn-ZnO buffer is characterized by the reaction $2Zn^0 + O_2 \leftrightarrow 2Zn^{2+}O$.

The simplest known reaction of converting an inorganic compound into organic is the reduction of CO_2 to a formic acid.

As shown in Figure S2.1B, this reaction requires very strong reducing agents with a redox potential of ≤ –600 mV. In general, the very low redox potentials of most organic molecules are outside the water stability range, as can be seen from Figure S2.1A. Paradoxically, water also hinders the reduction of CO_2, since the oxidation of strong reducing agents by ubiquitous water protons (E_0^7 –410 mV) is thermodynamically and kinetically more favorable than their oxidation by CO_2 (see Figure S2.1).

Since the abiotic formation of organic molecules is hardly possible on the surface of the present-day water-bathed Earth under an oxygen-rich atmosphere, the dominant source of reducing power in the biosphere is the chlorophyll-based photosynthesis, which uses energy of light to produce stable electron-donating compounds with redox potentials as low as –700 mV [43,44]. Photosynthesis is the most productive of several biogenic pathways for the so-called "autotrophic" reduction of CO_2 to organic compounds. Because of the need to avoid losing electrons to water, all autotrophic processes are very complex and involve many interacting enzymes [45].

It is chemically much easier to produce new organic molecules by transforming pre-existing organic molecules; a network of such "heterotrophic" transformations is an essential part of cellular metabolism. Recently, it has been shown that incubation of one of the sugar phosphates involved in glycolysis or the pentose phosphate pathway in warm water and in the presence of transition metal catalysts leads to the non-enzymatic formation of other intermediates of these pathways, which implies that the chemistry of the respective reactions is not particularly demanding [46-50].

Small amounts of organic molecules are also formed abiotically, during the so-called hydrothermal alteration of hot rocks at sites of geothermal activity. Earth's rocks contain about 5% iron, mostly as iron oxide, FeO. In the presence of geothermal fluids, at high pressure of the rock, and at temperatures <500ºC, some of the Fe^{2+} ions within the rock can be oxidized to Fe^{3+} by protons present in the geothermal fluids. This reaction produces magnetite (Fe_3O_4) and H_2. Various organic molecules (mostly hydrocarbons) have been shown to be produced under such conditions, albeit at low yields, presumably from the interaction of CO_2 either with H_2 or directly with iron oxides of hot rocks. These abiotically produced organic molecules are transported to the surface by geothermal fluids [51-64].

This phenomenon of H_2 formation in hot rocks allows the correlation of the redox potential scale for liquid systems at 25°C and pH 7.0 (Figure S2.1A) with the reducing power of hot solid

rocks, which is characterized not by redox potential but by the oxygen fugacity, $f(O_2)$. Fugacity (f) is defined as the effective partial pressure of a gas (in this case, oxygen gas) in thermodynamic equilibrium with a given mineral assemblage, see Figure S2.1C and [42,65-67]. Typically, $f(O_2)$ is reported in log10 units relative to well-characterized mineral redox buffers, see Fig. 2C and its caption. The $f(O_2)$ of the present-day Earth's crust typically corresponds to that of the fayalite-magnetite-quartz (FMQ) assemblage. As the hot rocks reduce water protons to H_2 at t° ≤ 500ºC, the reducing power of the FMQ assemblage at ~ 500°C ($\log_{10}f(O_2)$ ~ –24, see Fig. S2.1C) roughly corresponds to the reducing potential (power) of a hydrogen electrode at 25°C, i.e. –0.41 V at pH 7.0, see Figure S2.1A and [42,65-67]. Consequently, the reducing power of other mineral assemblages shown in Figure S2.1C, such as iron-wustite (IW) and iron-quartz-fayalite (IQF), is much higher than that of the hydrogen electrode. The $f(O_2)$ value decreases with temperature (Figure 2C), so that at t ° < 500°C the H_2-producing capacity even increases. However, at t° < 250°C, redox reactions in the rock attenuate because of their high activation barriers [68].

When liquid water interacts with a rock, it is possible to establish a relationship between the redox potential and the oxygen fugacity, since both parameters are significant under such conditions. For example, for the reaction described by Equation (S5), this relationship will be like follows [67]:

$$E = 1.228 – 0.5991pH + 0.0148\log f(O_2)$$

Although $f(O_2)$ is defined in terms of oxygen partial pressure, the value of $f(O_2)$ is used as an integral parameter to characterize the reducing power of the entire rock [65-67]. The corresponding integral parameter for complex fluid mixtures, such as the Earth's water reservoirs or cell cytoplasm, is the redox potential of the medium, E_h [65,67,69,70]. It can be measured using a chemically inert platinum or gold electrode capable of exchanging electrons with all redox agents present in the solution [65,71,72].

Most modern natural reservoirs in contact with the atmosphere, including the oceans at all depths, have high and positive E_h values in the range of +600 ÷ +750 mV [69]. This is because oxygen, with its E_0^7 of +820 mV (Equation (S5) and Figure S2.1A), usually acts as the dominant redox buffer.

In contrast, the redox potential inside cells is rather low, around –300 mV [73]. It is thought that the first cells initially lived in highly reduced habitats and then failed to adapt to the oxidation of their environment in response to the appearance of atmospheric oxygen [74]. Therefore, in most cases, cells must keep their cytoplasm much more reduced than the environment, which requires energy.

In addition to hydrothermal alteration, CO_2 can be abiotically photoreduced to various organic molecules at the surface of several UV-absorbing naturally occurring minerals with broadband semiconductor properties, in particular TiO_2 (anatase/rutile), MnS (alabandite), and ZnS (wurtzite, sphalerite), which are found at sites of geothermal or volcanic activity [39,75-80].

Apart from classical electrochemistry, CO_2 can also be reduced by ionizing radiation. This was first shown in the context of origin of life research when Calvin and his colleagues imitated terrestrial radioactivity by irradiating a mixture of hydrogen, water, and CO_2 with a helium ion beam. They obtained formic acid and formaldehyde [81]. Gethoff and his colleagues showed in 1960 that hydrogen is not needed as an electron donor to obtain organic molecules in such a system. The ionizing radiation itself generated "solvated electrons" with high reducing power by interacting with water molecules, so that irradiation of a CO_2/water mixture produced organic molecules and H_2 [63,82]. Although it is still unclear how the solvated electrons with an apparent E_0^7 of ~ –2.9 V are formed [83,84], their ability to promote the formation of carbon- and nitrogen-containing organic molecules has been demonstrated in various systems [85-87].

Not surprisingly, several origin-of-life scenarios invoke natural ionizing radiation as a source of reducing energy for primordial syntheses. Some of these scenarios consider radioactive isotopes of actinides, such as isotopes of uranium (U) and thorium (Th) [88,89], while others

rely on the radioactivity of the natural potassium isotope ^{40}K [90,91]. The radioactivity of the actinides is stronger, while ^{40}K is more abundant in the crust [90,91], so there seems to be a some sort of tie.

In general, it has been repeatedly shown that high energy inputs, such as UV illumination, γ-irradiation, proton beams, or electrical discharges, can produce organic molecules from simpler inorganic building blocks see [82,86,92-103] and references therein.

S2.2. Abiotic Syntheses of Organic Molecules and the Tar Paradox.

The immediate products of stepwise CO_2 reduction are simple organic molecules with one carbon atom (C1 compounds) such as formate, formaldehyde, methanol, and methane, see Figure S2.1B and [92,98]. Oparin speculated that the interaction of such simple organic compounds with hydrogen and nitrogen in the atmosphere may have resulted in increasingly complex organic molecules [104,105]. In 1952, Miller and Urey tested this hypothesis experimentally by sending electrical discharges through a flask containing water vapor, hydrogen, methane, and ammonia. They obtained amino acids and some other organic molecules [93,101].

In all such experiments, however, the reaction products are mostly deposited on the walls of the experimental flasks as tar incapable of further chemical transformation. The reason is that randomly formed organic molecules are usually nonpolar and tend to stick together, which is what happens in the experiments.

Benner and his colleagues approached this problem experimentally. In addition to their formulation of the "tar paradox" ("Organic systems, given energy and left to themselves, devolve into uselessly complex mixtures" [33]), they showed how tar deposition can be prevented in the case of the so-called formose reaction, discovered by Butlerov as early as 1859 [106]. This reaction is thought to be of key importance for prebiotic syntheses because it produces a viscous mixture of sugars and organic acids with 4 to 7 carbon atoms (C4-C7

compounds) from C1-C3 aldehydes and/or alcohols [107,108]. The Butlerov reaction appears to be unique in producing complex molecules from simpler building blocks without energy input and in water. The reaction proceeds spontaneously at moderately elevated temperatures and mildly alkaline conditions and is enhanced by metal cations (Ca^{2+}, Mg^{2+}, Na^{+}, K^{+}) as catalysts. The reaction is stimulated by UV light, in the presence of which it proceeds even with a C1-molecule of formaldehyde (COH_2, see Fig. S2.2.) as the sole substrate [109]. The biologically most relevant ribose, a component of RNA, makes up about 1% of the product mixture.

Benner and his colleagues showed that the "caramelization" of the product sugars can be prevented by adding high concentrations of borate to the solution. Borate anions (BO_2^-) by binding to the sugar molecules in a unique way (see Fig. S2.2.), drove the reaction toward the specific formation of C5 sugars, which increased the yield of ribose [110,111].

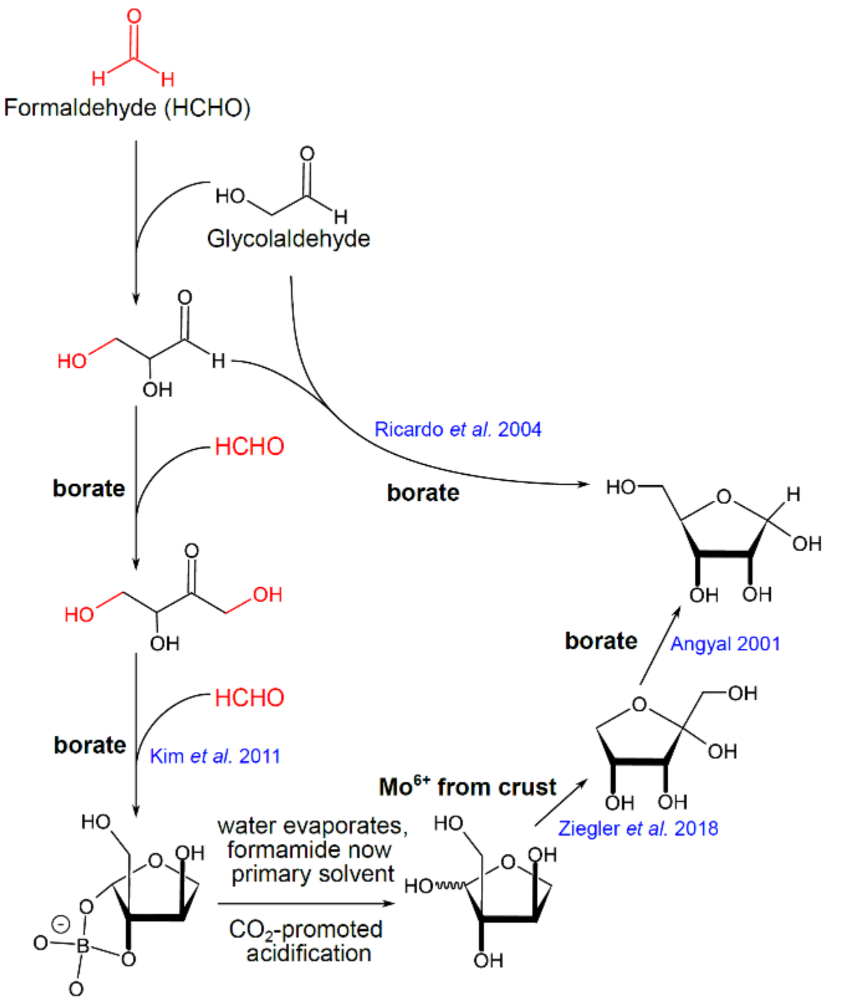

Figure S2.2. Formation of ribose under borate and molybdate control (image is based on [112]).

However, most of the C5-sugars obtained in the presence of borate were branched, in contrast to the linear sugars present in RNA and DNA. Therefore, Benner and his colleagues next showed that branched C5-sugars can be linearized in the presence of molybdenum oxide (MoO_4^{2-}) as a catalyst, see Figure S2.2. and [112].

The pioneering research of Benner and his colleagues provides a tentative solution to the "tar" paradox. Abiotically formed organic molecules can avoid getting into tar by specifically binding to solvent components. This gives them a chance to participate in further catalytic transformations, the direction of which can be controlled by particular molecules present in solution. So, the solvent matters.

S2.3. Key biomolecules contain many CN bonds that are rare in inorganic nature (paradox of the CN bonds)

Proteins and nucleic acids are more nitrogen-rich than other organic molecules. As shown in Fig. S2.3, they contain many CN bonds. Paradoxically, CN bonds are not common in inanimate nature. Therefore, the origin of the CN bond in biomolecules has been widely debated.

As early as 1976, Mukhin and his colleagues discovered large amounts of HCN in the volcanic lava and attributed them to high-temperature chemical reactions in the throat of volcanoes [53]. As Calvin pointed out, the reaction of cyanide formation from methane and ammonia becomes thermodynamically favorable at $T > 1050$ K [116].

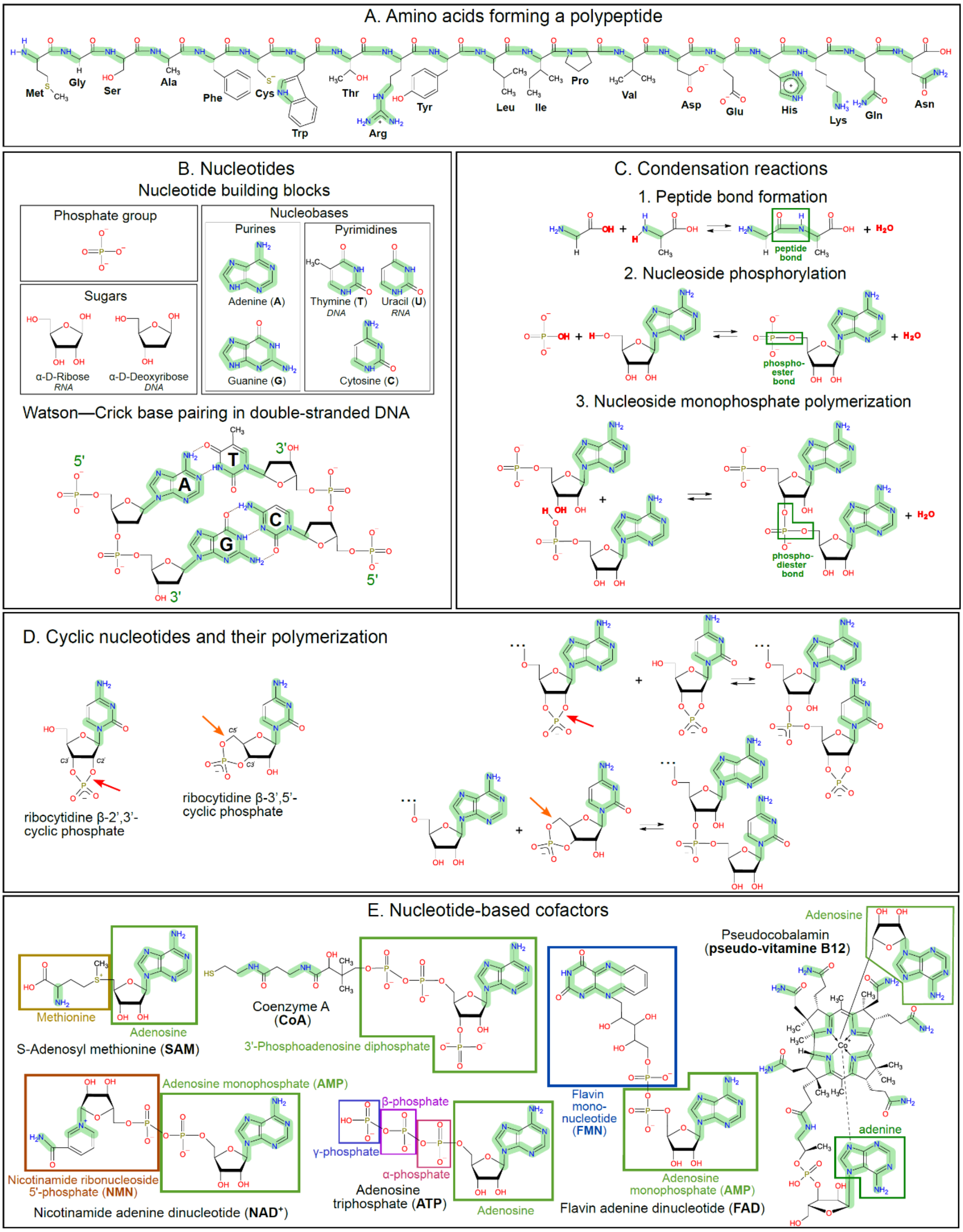

Figure S2.3. Biomolecules, the CN bonds are shaded green. A, amino acids and formation of a peptide bond between them; B, Nucleotides, their structure and complementary interactions; C, condensation reactions; D, cyclic nucleotides and mechanisms of their polymerization (modified from [113]); E, ribonucleotide-containing enzyme cofactors, see [114,115].

A promising simple starting compound for making CN bonds is ammonium formate [117]. It does not contain a covalent CN bond but can be converted to the CN bond-containing formamide ($HCONH_2$) either via a single dehydration step (Figure S2.4.A) or in the course of interaction with urea as studied by Hud and his colleagues [117]. Notably, both ammonium and formate can be produced in geochemical processes. Ammonium is the form in which nitrogen is present in the mantle and is delivered by geothermal vapor [118], see also Section S2.8. below. Formate is produced in the very first step of any abiotic CO_2 reduction (see Figure S2.1B above).

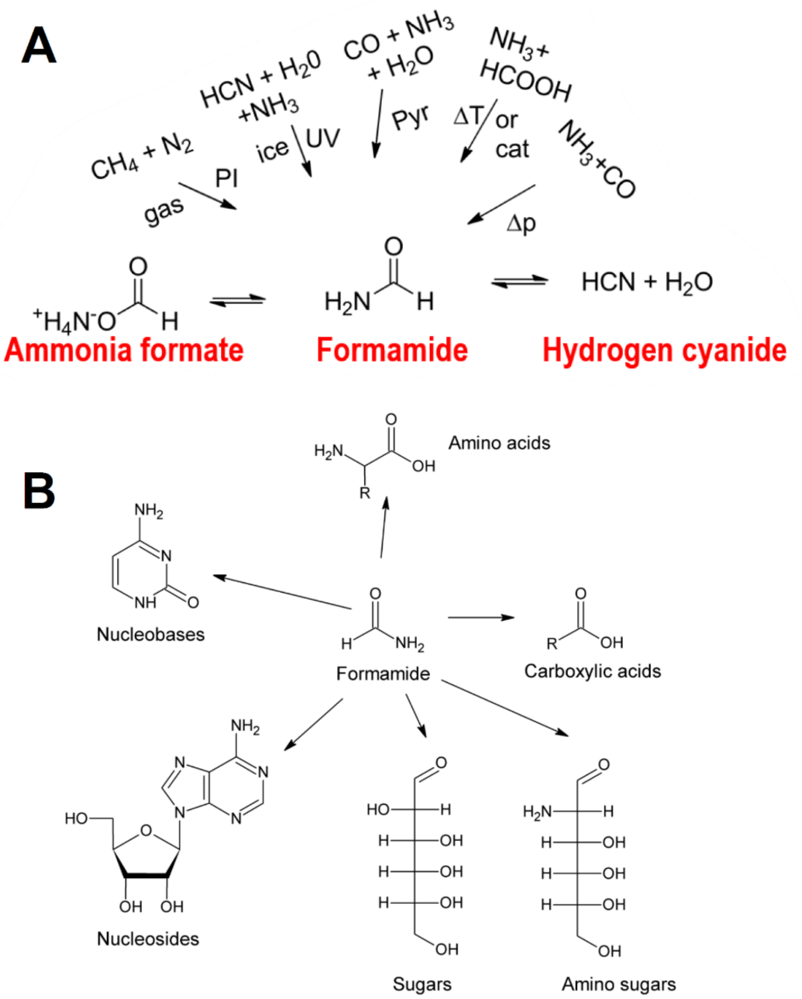

Figure S2.4. Bio(chemistry) of formamide. A, Pathways of formamide formation, and relation between formamide, ammonium formate, and hydrogen cyanide; abbreviations: PI = Photon Irradiation, Pyr = Pyrolysis, DT and DP = high temperature and/or high pressure; B, Formamide-derived biomolecules. Image credit, Image credit: Judit E. Šponer, Wikipedia, modified; see also [119].

Not surprisingly, Saladino, di Mauro and many others have favored abiotic syntheses of biomolecules from formamide ($HCONH_2$), sometimes called a “free-living peptide bond” because of the structural similarity, cf Figures S2.3A and S2.4. It has been repeatedly shown that heating of formamide, illuminating it with UV light, or treating it with a proton beam yields nitrogen bases and amino acids; the reactions can be boosted by certain minerals, see Figures S2.4B and [86,87,102,119-135]. These exciting findings led to the concept of the origin of life in a “Formamide World” based on the ability of formamide (if present) to accumulate upon the evaporation of water owing to its high boiling temperature of 210°C [102,129].

Sutherland and his colleagues put more emphasis on nitriles, compounds with a highly reactive terminal CN group, with hydrogen cyanide (HCN) as their simplest representative (Figure S2.4A). These authors have elucidated a network of chemical reactions leading to nucleotides, amino acids and even glycerol, which is the building block for both sugars (via the formose reaction, see Section S2.2 above) and lipids [136-141].

The molecules containing CN bonds, such as cyanide or formamide, may also have been formed in the primordial atmosphere and Hadean ice by UV light [142,143], solar wind [144], and electrical discharges that were simulated already by Miller [93,101].

S2.4. The water paradox.

As noted in Section S2.1, water hinders the reduction of CO_2, becuase the oxidation of strong reducing agents by ubiquitous water protons (E_0^7 = –410 mV) is thermodynamically and kinetically more favorable than their oxidation by CO_2 (E_0^7 = –610 mV, see Figs. S2.1). Therefore, researchers perform reductive syntheses of organic molecules - both in the liquid phase and on electrodes - either in anhydrous solutions or in mixtures with a small fraction of water [145,146].

In addition, a common feature of biopolymers, such as RNA, DNA, proteins, and oligosaccharides, is that they are formed in the cell by a polycondensation mechanism, where

the addition of each monomer is accompanied by the release of a water molecule, see Figure S2.3C and [33,147]. Furthermore, even the formation of single nucleotides involves condensation of a nucleobase with a sugar to form a nucleoside and further condensation(s) with phosphate group(s), see Fig. S2.3C and [148]. Such condensation reactions cannot occur spontaneously in water; on the contrary, water usually shifts the reaction equilibrium toward the breakdown of such polymers. In modern organisms, these polycondensation reactions proceed only when driven by excess free energy provided by bioenergetic reactions.

Hence, since water prevents the formation of organic molecules from CO_2 and ultimately leads to the destruction of DNA, RNA, proteins, and polysaccharides, it could hardly have served as a suitable medium for their spontaneous abiotic emergence at the beginning of time, which could be considered one of the greatest paradoxes of life.

To enable condensation reactions at the origin of life, researchers have usually considered fluctuating systems with wet/dry cycling, such as tidal zones, periodically drying pools, geothermal systems with fluctuating activity, deserts periodically wetted by rain, and so on [34,133,149-155].

Another possibility is represented by eutectic (water/ice) systems in which water is temporarily removed from the reaction volume by freezing [156-160]; in such systems spontaneous polycondensation of nucleotides actually occurs [161-163].

Yet another option is the formation of the first biopolymers in anhydrous solvents, specifically in formamide, which can additionally serve as a building block for biomolecules, see Section S2.3. above and [102,129,152].

While envisioning the formation of complex primordial biomolecules, several researchers have considered so-called impact crater scenarios [132,137,139,164]. In these scenarios, the early evolution of life was proposed to follow the evolution of a meteoritic crater: as long as the crater was hot, water boiled out and biomolecules could form from formamide/cyanide. As the crater cooled down, the condensation reactions and the formation of the first biopolymers became

possible, and eventually water from rain and snow could flow into the crater, forming cool ponds that could serve as hatcheries for the first cell-like organisms.

S2.5. The chicken-and-egg paradox of the first biopolymer: The concept of RNA World

Proteins are made with the participation of DNA and RNA while DNA and RNA are synthesized by protein enzymes, which is a kind of chicken-and-egg paradox. Currently, most biologists resolve this paradox by assuming that the RNA-like polymers came first. The very first organisms are envisioned as aggregates/consortia of small RNA-like molecules that reproduced themselves and catalyzed some biosynthetic reactions [160,162,163,165-199].

This idea is remarkably old. As early as 1957, after discovering the non-coding, structural rRNA (see Section S1.1-S1.3. and [200]), Belozersky reported at the 1st Congress on the Origin of Life: “it seems rather that ribonucleotides and then RNA originated first” [165]. He argued that RNA molecules could both store genetic information (as in RNA viruses) and perform some protein functions (as in ribosomes). Similar concepts were put forward by several other researchers [166-169]. After RNA molecules were found to be able of catalyzing chemical reactions (such RNA molecules are called ribozymes, see [171,172]), Gilbert came up with the vision of the “RNA World … containing only RNA molecules that serve to catalyze the synthesis of themselves” [173].

Chetverin and his colleagues showed that “RNAs themselves can rearrange their sequences under physiological conditions, without the need for group activation or assistance from proteins or ribozymes” [201], an ability which may have been of great evolutionarily importance.

The primacy of RNA is also supported by the observation that ribonucleotides are part of many organic cofactors (Figure S2.3E), which is seen as evidence for the emergence of these cofactors in the primordial RNA World [114,202].

As described in Section S1, the ribosomal protein synthesis apparatus forms the ancient core of the cell. When the first structure of the ribosome was solved, it was shown that the linking of the two amino acids is accomplished by RNA loops; no proteins are involved [7,8]. It turned out that proteins are made by those rRNA molecules discovered by Belozersky and Spirin in 1957.

More recently, the evolution of the ribosome has been reconstructed by using several different methods, allowing a deeper look into the primordial RNA world. In each case, an ancient catalytic peptidyl transferase center was identified in the core of the large ribosomal subunit (LRS); this center consists of two pseudosymmetric regions of only about 60 nucleotides each, see [203-211]. Furthermore, the fragments of this catalytic center have been shown to synthesize dipeptides [212,213]. These reconstructions provide conclusive evidence that the RNA World did exist and was inhabited by consortia of RNA molecules of some 50-60 nucleotides.

The evolutionary primacy of RNA is also independently supported by the fact that RNA is the *only* biopolymer present in all known life forms, namely cells, viruses, and viroids (see Section S1.3.).

Manfred Eigen was the first to recognize the danger of an “error catastrophe” for the RNA World [214,215]. Eigen has calculated that primitive RNA-like replicating entities could not properly transmit information through generations unless they possessed a sophisticated error-free replication machinery. The maximum length of a correctly replicated chain in a primordial, enzyme-free system was estimated to be about 100 bases, which is obviously insufficient to encode elaborated replication machinery. As a solution to the problem, Eigen and Schuster proposed “hypercycles” in which short RNA and protein oligomers helped each other to maintain information without loss [216-218]. In the following decades, the versatility of RNA molecules as catalysts was fully appreciated, so that some current versions of hypercycles no longer invoke proteins, but consider aggregates of short interacting RNA oligomers, see e.g.

[179,219]. Recently Joyce and his colleagues seemed to succeed in overcoming the error threshold in an experimental, purely RNA-based setup [196].

The current view is that the first RNA organisms may have initially propagated not by self-replication, which implies sequential addition of individual nucleotides along the available template, but by a less streamlined process, in which new RNA oligomers are assembled from shorter oligomers in interaction with templates. We will refer to this mode of assembly as self-copying to distinguish it from "self-reproduction", which refers to the "duplication" of the entire contents of a protocell [220]. Several systems capable of self-copying by cooperation between short RNA oligomers have been obtained and experimentally studied [175,176,194,196].

Further support for the RNA World concept came from studies of RNA molecules replicating inside lipid vesicles that mimic protocells [187,221-229]. The authors of these studies usually used either enzymes or artificially activated nucleotides to speed up the reactions studied. Nevertheless, these studies are very important as proof of principle. At the same time, Chetverin and his colleagues, using a protein replicase, obtained RNA colonies growing on a solid substrate and studied their properties [230,231].

The key problem with the RNA world concept is the complexity of the ribonucleotides. Each consists of a nitrogenous base, a ribose moiety, and one or more phosphate groups (Figure S2.3). The closed electronic system of the nitrogen base makes it (photo)stable, as discussed in the next section, but hinders the formation of the so-called glycosidic bond with the ribose moiety, see [35,232] and references therein. Since it is difficult to assemble a nucleotide from its components even under laboratory conditions, it has remained unclear how this could have occurred under primordial conditions in the absence of enzymes.

Among many new pathways of abiotic nucleotide synthesis [35,136,137,233-235], only one, as reported by Carell and colleagues, has so far provided a "unified prebiotically plausible synthesis of pyrimidine and purine RNA ribonucleotides" see Figure S2.5. and [235]. This synthesis mimics events in a shallow primordial pond that could have undergone wet/dry

cycles. Upon successive steps of this synthetic pathway, 3-aminoisoxasole with T_B as high as 225°C is used first as a low-volatile solvent and later as a key common intermediate which is converted to N-isoxazolyl-urea using Zn^{2+} ions as catalysts. In another catalytic step metallic Zn is used to reduce nitroso-pyrimidines to formamidopyrimidines.

Earlier, Sanchez and Orgel showed the possibility of stepwise assembly of a nitrogen base on a sugar template [236]. Using a similar approach, Powner and his colleagues developed a novel one-pot synthesis protocol in which the base precursor closed its ring *after* attachment to the sugar [136]. In general, this approach has proven to be a very convenient technique that is now widely used in nucleotide synthesis, see [233] for a review.

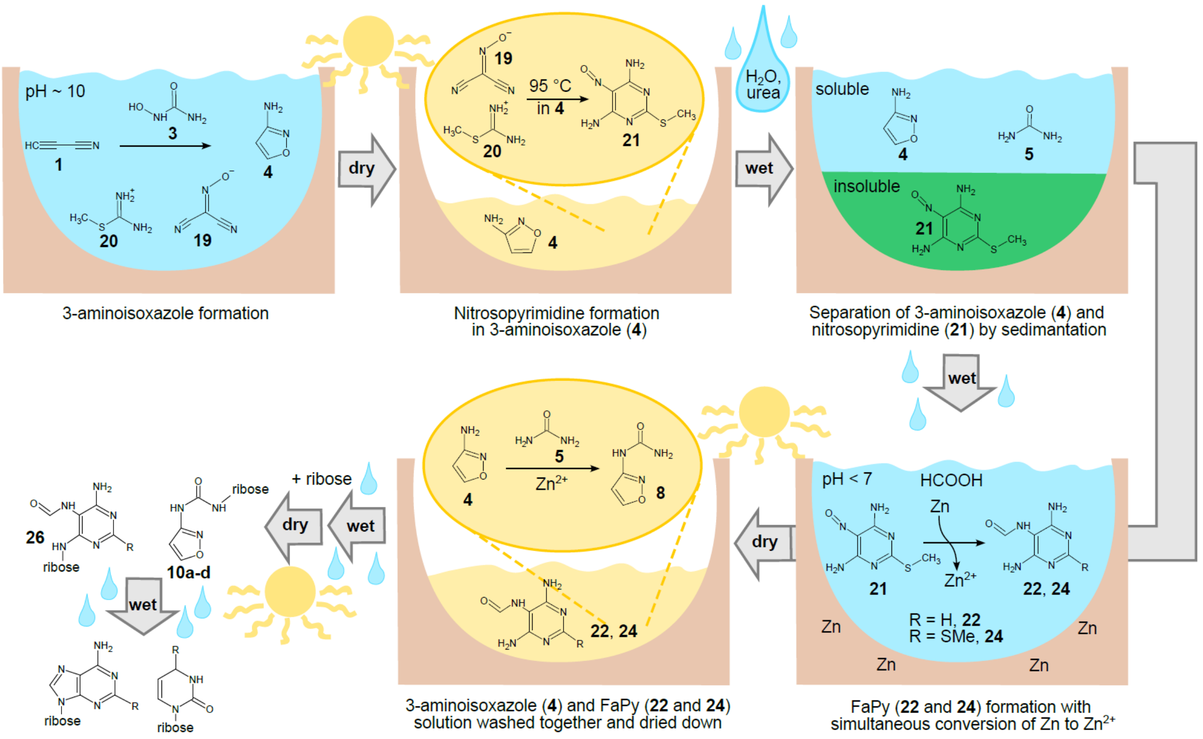

Figure S2.5. Proposed geochemical scenario for the simultaneous synthesis of purine and pyrimidine nucleosides, driven by wet-dry cycles (redrawn from [235] with modifications). In yellow, the solvent is 3-aminoisoxazole (**4**), which can be enriched from an aqueous solution due to its high boiling point (228 °C).

Remarkably, the pioneering synthesis by Powner and his colleagues [136], yielded a ribocytidine β-2',3'-cyclic phosphate, i.e. a cytidine nucleotide with a phosphate group making

not one but two bonds with the sugar moiety, see Fig. S2.3D. We will refer to such 2',3'-cyclic nucleotides as 2',3'-n>P, where ***n*** is the nucleoside moiety.

Another intensively studied type of cyclic nucleotides are the 3’,5’-cyclic nucleotides (3’,5’-n>Ps, see Fig. S2.3D and [134,135].

The cyclic nucleotides are “pre-loaded” with the free energy needed for their polymerization. The enthalpy of scission of the additional phosphate-oxygen bond in cyclic nucleotides is about –40 kJ/mol [237]; this energy can be used for binding to another nucleotide, so the cyclic nucleotides are considered “activated”. Notably, their binding to another nucleotide or to an RNA oligomer can even proceed without the release of a water molecule (see Figure S2.3D), thus overcoming the water paradox (see Section S2.4.).

The 2',3'-n>Ps are the immediate products of the RNA hydrolysis by several small “housekeeping” ribonucleases (RNAses) [238,239]. In the 1960’s, biotechnologists have tried to force such RNAses to work in the reverse mode and to synthesize oligonucleotides. RNAses synthesized short oligonucleotides from 2',3'-n>Ps in various solvents including formamide; the yield was particularly high at 0°C, see [240] for a review. An RNAse itself cannot affect the thermodynamics of the reaction, so the polymerization of 2',3'-n>Ps must be thermodynamically favorable at ≤ 0°C even in the absence of RNAses.

Recently, Braun and his colleagues described the non-enzymatic oligomerization of 2’,3’-n>Ps in thermal gradients [241], as well as at heated air-water interfaces at pH range of 7–12; in the latter case, the reaction was slightly enhanced by K^+ ions [242]. The 3’,5’-n>Ps have also been shown to spontaneously polymerize under certain conditions, especially on the surface of silicate minerals [134,135]. These observations prove the ability of cyclic nucleotides to polymerize without enzymes, which may have also occurred on the Hadean Earth.

S2.6. Natural nucleotides are extremely radiation-resistant (photostability paradox). Canonical nucleosides typically convert the energy of a UV quantum into heat in about 10^{-13} s (Figure S2.6), much faster than any destructive chemical reactions can occur. It has been argued

by several authors that the unique ability to dissipate excitation energy in femtoseconds is due to very fast deformations of the nucleobase rings and in particular to torsional motions around the CN bonds [244-246]. In addition to the five canonical bases, the ability to rapidly discard UV quanta is also shared by hypoxanthine, a non-canonical but natural base of the minor nucleoside inosine, see Figure S2.6 and [245]. Inosine often plays a key role in ribosomal protein synthesis and is thought to have preceded guanosine in evolution [247]. It is also paradoxical that this exceptional photostability of natural bases is apparently unrelated to mechanisms of information transfer.

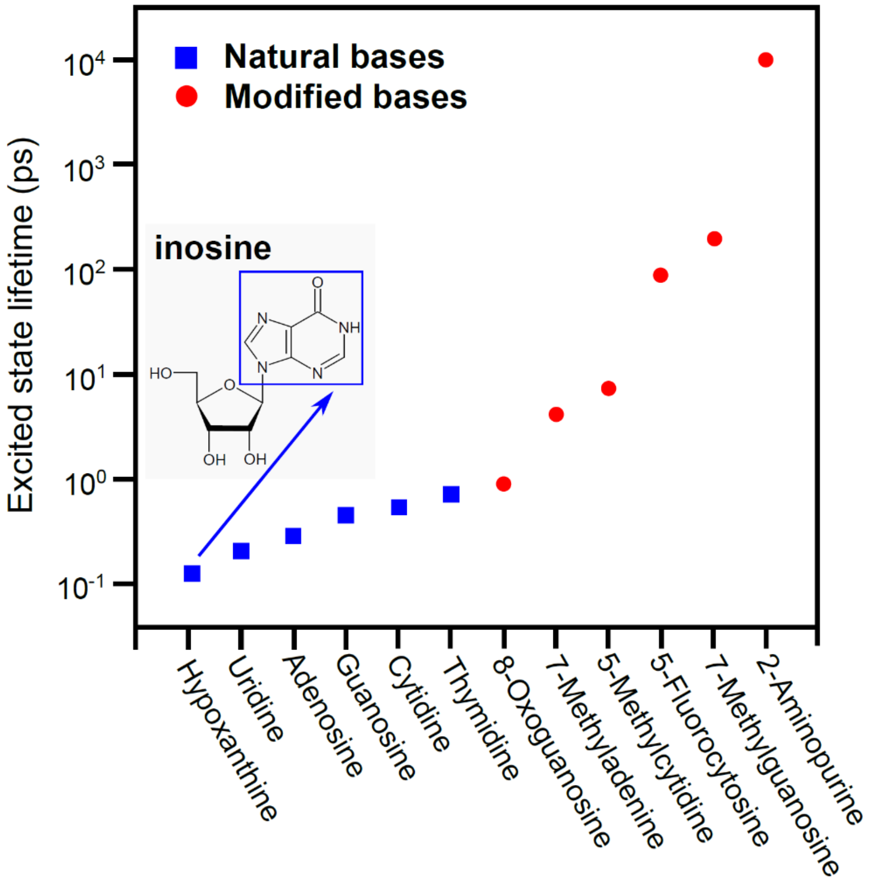

Figure S2.6. Excited state lifetimes of natural bases (blue squares) and modified bases (red circles) in aqueous solution measured in femtosecond transient absorption experiments. While the modified bases have lifetimes that span several orders of magnitude (note the logarithmic scale) all of the natural bases have lifetimes of < 1 ps. Redrawn from [245] with additions and modifications.

Nucleosides (nucleobase+sugar, see Section S1.1 and Figure S2.3B) strongly absorb UV light in

the range of 260-280 nm. This property underlies the popular belief that solar UV quanta

absorbed by DNA can cause the hazardous DNA mutations that sunscreens are designed to

prevent. This ability of nucleosides to trap potentially damaging UV quanta is paradoxical because it would seem to compromise the reliability of genetic information storage. Actually, it is not that bad; 99.9% of UV quanta are trapped by nucleoside moieties, which are exclusively photostable to UV radiation compared to structurally similar compounds such as aromatic amino acids or non-canonic nucleobase analogs [136,243-245].

One might ask: if nitrogen bases are so efficient at deactivating UV quanta, why is UV light considered harmful? In fact, the main damage comes from truly dangerous backbone breaks caused by theabsorption of UV light not by the nucleotides themselves, but by the phosphate groups that link the nucleotides together, see Figure S2.3 and [248]. Due to their resonant structures, the phosphate groups also absorb UV light in the same range of 260-280 nm, albeit very weakly [249,250]. However, their photoexcitation produces a reactive phosphate radical that is able to break the sugar-phosphate backbone with a very high quantum yield of about 0.5, i.e. in every second case [248,251].

Due to their ability to intercept and dissipate UV quanta, nitrogen bases efficiently protect RNA and DNA from these breaks. The extent of this protection can be estimated from the data of Goossen and Kloosterboer, who simultaneously irradiated glycerol-2-phosphate and AMP with UV light at 254 nm and monitored their photodestruction by measuring the release of phosphate [252]. Phosphate was photo-released ten times faster from glycerol-2-phosphate than from AMP [252], even though the adenine moiety of AMP absorbed 10^4 times more UV quanta per unit time than the phosphate moiety of glycerol-2-phosphate [248]. Thus, in RNA, nitrogen bases provide at least 10^5-fold protection of the sugar-phosphate backbone from UV cleavage. This feature explains why deleterious UV damage to the backbones of RNA and DNA molecules is 10^3–10^4 times less frequent than photodestruction of the nitrogen bases proper (Cadet and Vigny 1990). Eventually, the bases are sacrificed to prevent the breaks in the backbones of RNA and DNA; however, provided that the backbone is not damaged, the cellular repair systems rapidly replace the damaged bases [253].

Hud, di Mauro and their colleagues reported the formation of notable amounts of adenine, guanine, and hypoxanthine upon irradiation of a 10% formamide solution in water with UV light; these nitrogen bases were the main products of the irradiation [130]. Furthermore, irradiation of liquid formamide with a high-energy proton beam in the presence of powdered meteorites yielded a broad spectrum of organic compounds, the most complex of which were the canonical nitrogen bases [86]. In another set of experiments, the proton beam catalyzed the transglycosylation of pyrimidine nucleobases to yield canonical N^1-pyrimidine nucleosides [87]. These data indicate that canonical nitrogen bases and nucleosides, once formed in an energy flux, are more resistant to high-energy radiation than other compounds of comparable complexity.

The photostability of nitrogen bases may have played a key role in the origin of life. As early as 1973 Carl Sagan suggested that they may have initially served as UV-protectors [254]. He argued that the 240-300 nm window was transparent for potentially damaging solar UV radiation before oxygen accumulated in the atmosphere and could be converted to ozone by solar UV quanta, so protection from this radiation may have been a prerequisite for the emergence of the first replicating entities.

In summary, nitrogen bases are not only letters of the genetic alphabet, but also UV-protectors, which may have been their original function, as has been suggested by Sagan [254] and is elaborated in Section S2.7. below.

S2.7. Emergence of the first complex molecules (the complexity paradox)

Although the second law of thermodynamics tells us that the disorder of the universe increases with time, living beings are rather well-ordered. This paradox is usually explained by complexification of organisms at the expense of external energy – brought in by light, contained in the food and so on. Organisms are quite adept at using this energy to drive thermodynamically unfavorable reactions, namely synthesizing highly reduced compounds,

driving polycondensation reactions in the water phase, or maintaining chemical disequilibria across cell membranes. However, the very first organisms had neither energy-harvesting nor energy-transforming systems, yet they had to reach a certain level of complexity to survive. How could external energy have been used to produce increasingly complex molecules without sophisticated energy conversion machinery?

As far as we know, this paradox was first formulated by Carl Sagan who also proposed its solution. As early as 1957, he wrote that "differential survival of polymerized molecules over unpolymerized molecules" under condition of a high-energy flux, such as UV light, could promote the selective accumulation of more complex molecules [255].

Elsewhere, building on the experimental observations of [248,252] considered in Section S2.6, we used Monte-Carlo modeling to investigate how the ability of attached nitrogen bases to protect sugar-phosphate units in RNA-like polymers from UV damage might have affected the complexity of such units, see Figure S2.7 and [256]. Upon modeling, the binding of nitrogen bases to sugar-phosphate moieties was set to be thermodynamically unfavorable (see the caption to Fig. S2.7 for the reaction constants used). When the nitrogen bases were assumed to provide no UV protection, the polymers were short and the extent of nucleobase incorporation into the polymers was close to zero (Figure S2.7, circles in panels *a* and *b*). In another simulation run, the UV protection was "turned on" so that the binding of a nucleobase to a sugar-phosphate moiety reduced the probability of its UV breakage by a factor of 30, which is a rather modest value compared to the experimentally determined factor of 10^5, see Section S2.6. and [248,252]. In this case, the sugar-phosphate units began to acquire UV-protecting nitrogen bases, and the length of the polymers increased dramatically (Figure S2.7, triangles in panels *a* and *b*).

When we simulated the funneling of UV energy into the condensation reactions with an efficiency as low as about 10^{-7}, the length of the polymer chains formed increased dramatically

and these chains comprised predominantly from nitrogen base-possessing nucleotides (Figure S2.7, squares in panels *a* and *b*). This result was expected and trivial, though.

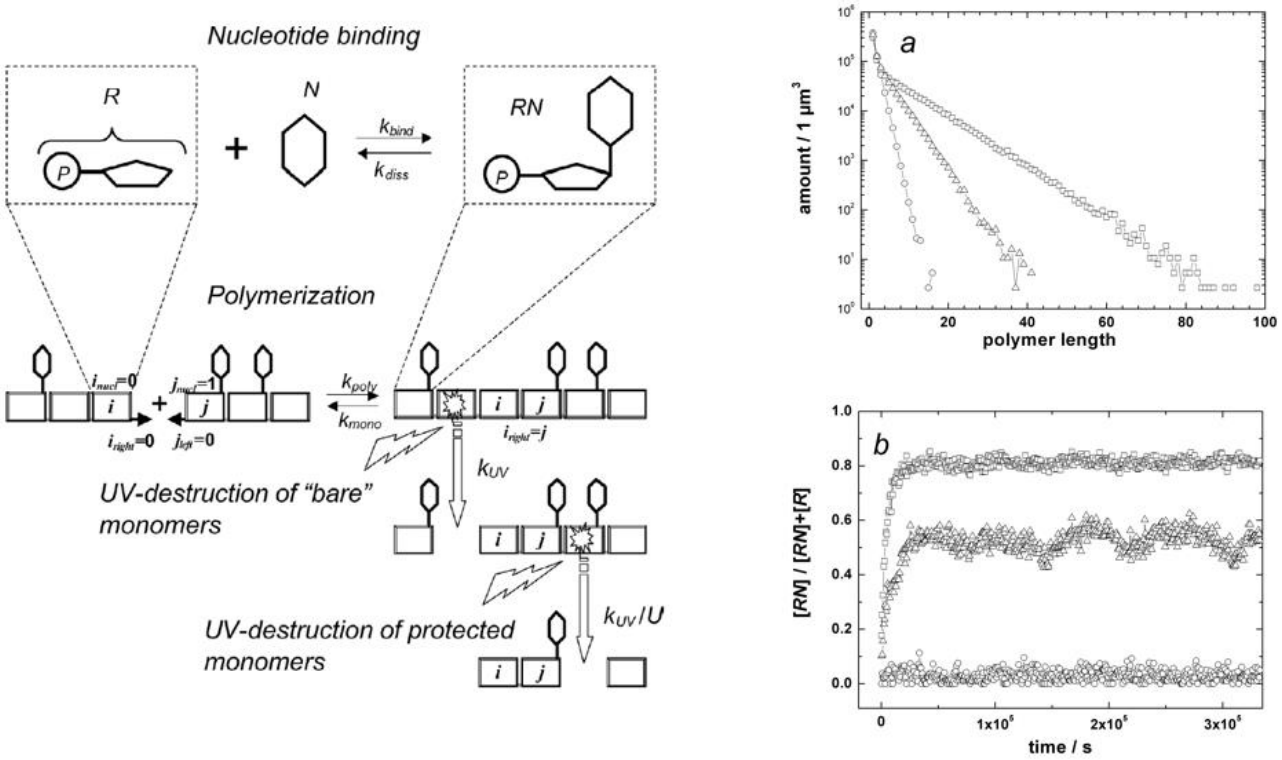

Figure S2.7. Monte Carlo simulation of a sugar-phosphate polymerization reaction in the presence of nitrogenous bases and under UV-illumination. The image is taken from [256]. The sugar-phosphate polymerization reaction in the presence of nitrogenous bases and under UV-illumination was simulated using the following set of parameters: The concentration of monomers in the reaction volume was kept constant at 10^{-3} M (comparable to their concentration in the cell). The second-order rate constant of polymerization k_{poly} was 3 M^{-1} s^{-1} and the first-order rate constant of re-dissociation k_{mono} was 10^{-4} s^{-1}, which corresponds to an equilibrium constant of 30. The rate constants of nucleotide binding (k_{bind}) and dissociation (k_{diss}) were 3×10^{-8} s^{-1} and 10^{-6} s^{-1}, which corresponded to an equilibrium constant of 3×10^{-2}. Under the UV illumination, the monomers decomposed with the rate constant of $3 \cdot 10^{-3}$ s^{-1}, regardless of their position in the chain. For simplicity, the UV protection factor *U* of 30 was used for both monomers and oligomers. The partial funneling of UV energy was assumed to increase the k_{bind} value from 3×10^{-8} s^{-1} up to 1.2×10^{-7} s^{-1}. *a*, Polymer length distribution at equilibrium. *b*, Fraction of monomers protected by nitrogenous bases as a function of time.

The increase in the relative proportion of longer, nucleobase-carrying polymers in response solely to the "turning on" of the UV-quenching ability of nitrogen bases (Figure S2.7, triangles in panels *a* and *b*) was not trivial at all, since no radiation energy was allowed to be funneled into any reaction of bond formation in these simulation runs. The increase in the proportion of more complex molecules was solely due to the UV-destruction of less photostable molecules down

to building blocks that could then re-enter the polymerization reactions. Since the number of molecules in the Monte Carlo simulation was limited, the relative proportion of more photostable and thus more complex polymers increased under UV light, in accordance with Carl Sagan's prediction [255]. Therefore, and this is the key point, the enrichment in more complex RNA-like polymers proceeded without direct chemical coupling between energy flow and bond formation.

Furthermore, the folding of RNA molecules into double helices has been shown to increase their UV stability a hundredfold [257], suggesting that complementary nucleobase pairs may have been specifically photoselected under UV irradiation. Based on all these observations, we hypothesized that, in the absence of the ozone layer, the UV-rich sunlight may have promoted the selective accumulation of RNA-like polymers capable of forming double helices [256].

Remarkably, the above described one-pot synthesis of pyridine nucleotides by Powner and his colleagues (see Section S2.5. and [136]) yielded initially a mixture of 2’,3’-cytidine>Ps with several by-products. To get rid of the latter, the authors exposed the mixture to 254 nm UV light for three days. The prolonged UV irradiation resulted in the destruction of the by-products and the partial conversion of the β-ribo*cytidine*-2',3'-cyclic phosphate into β-ribo*uridine*-2',3'-cyclic phosphate (2’,3’-uridine>P), another activated natural ribonucleotide. The authors concluded that “there must be some (photo)protective mechanism functioning with natural nucleotides but not with other pyrimidine nucleosides and nucleotides” [136]. This photoprotective mechanism not just prevented the destruction of natural 2',3'-n>Ps, but also preserved the additional high-energy bond (see the red arrow in Figure S2.3D) despite its susceptibility to spontaneous hydrolysis. The fact that this bond persisted after three days of UV irradiation may indicate that the energy of the UV light specifically restored this additional bond in the 2',3'-pyrimidine n>Ps after its eventual breaks. This groundbreaking experiment documents the UV selection of natural nucleotides - in their high-energy, polymerization-prone state – from a mixture of different structurally related compounds.

The emergence of complex, potentially information-bearing molecules by UV selection seems to be related to the so-called Landauer principle [258]. Landauer analyzed the thermal behavior of a physically realistic computer with a limited number of memory units and showed that information cannot be stored without expending energy. He found that energy can be expended in two ways: either to store information in the empty memory units, or to "clean up" the previously filled but no longer needed memory units. Thus, energy can support the emergence of new information - aka an increase in complexity - by dismantling the unneeded, less complex entities. In this respect, Landauer's formalism is consistent with our Monte-Carlo simulation, which used a limited number of building blocks [256], as well as with the experiments of [136], where the amount of chemicals in the flasks was obviously limited.

Therefore, the experimentally demonstrated UV-selection of activated natural 2',3'-n>Ps [136], as well as the results of our earlier modeling of UV effects on RNA-like polymers [256], indicate that the emergence of activated nucleotides and their polymers on the primordial Earth may have been driven by energy-consuming, selective destruction of less "perfect" variants, in agreement with the insightful suggestion of Carl Sagan [255]. The Landauer formalism implies that the preferential accumulation of radiation-resistant – and therefore complex - activated 2',3'-n>Ps [136] was driven by the energy of UV light in accordance with the laws of thermodynamics.

Thus, complex molecules, even in their high-energy reactive states, may have emerged and accumulated at the expense of solar UV radiation even before the development of complex biological mechanisms for harnessing energy and directing it to synthetic reactions.

S2.8. Phenomenon of chemistry conservation and predominance of K^+ over Na^+ inside the cells (paradox of high intracellular potassium levels)

Table S2.1 shows the difference between the concentration of inorganic constituents of seawater, the water of the ancient anoxic ocean, cellular cytoplasm, and extracellular media

represented by the blood plasma, as compiled from [259-267]. Living cells contain much more phosphate (PO_4^{3-}), potassium, magnesium, and zinc than the media they reside in. In contrast, the intracellular concentrations of Na^+ and Ca^+ ions are usually much lower than in the environment. These characteristics are common to archaea, bacteria, and eukaryotes which diverged about 4 Ga ago (see Section S1.3).

Table S2.1. Molar concentration of life-relevant inorganic substances in different media. Data are compiled from refs. [259-266,268].

Substance	Cell cytoplasm	Blood plasma	Today's sea water	Anoxic, sulfidic ocean
Na+	0.014	0.142	0.4	> 0.4
K+	0.1	0.005	0.01	~0.01
Mg^{2+}	0.1-0.01 (mostly bound)	0.0015	0.05	~0.01
Ca^{2+}	10^{-7}÷10^{-6} (free) 10^{-3} (bound)	0.002	0.01	~0.001
Zn^{2+}	10^{-3} to 10^{-4}	1.0-1.5 × 10^{-5}	10^{-8}	10^{-15} ÷ 10^{-12}
Fe	10^{-3} to 10^{-4}	0.0015 (transferrin-bound)	10^{-8} (mostly Fe^{3+})	10^{-5} (Fe^{2+})
Mn^{2+}	10^{-3} to 10^{-4}	10^{-8}	10^{-10}	10^{-8}
Cu (I) – Cu (II)	10^{-5} to 10^{-4}	10^{-5}	10^{-9} (Cu^{2+})	< 10^{-20} (Cu^+)
Mo (IV) – Mo (VI)	1.6×10^{-7}	10^{-8}	10^{-7} mostly Mo (VI)	10^{-11} to 10 $^{-9}$ mostly Mo (IV)
Cl^-	0.15	0.1	0.5	~ 0.5
PO_4^{3-}/HPO_4^{2-}	~0.01 (mostly bound)	0.001	10^{-6} to 10^{-9}	<10^{-5}
CO_2/HCO_3^- /H_2CO_3	0.025	0.027	0.002	0.1-0.02
S (free)	~10^{-1} (mostly as methionine and cysteine)	0.0005 (SO_4^{2-})	0.026 (SO_4^{2-})	~10^{-2} (mostly S^{2-})

Unlike biopolymers such as RNA, DNA, and proteins, which are enclosed by the cell, small molecules and ions constantly leak across cell membranes, driven by their concentration gradients. Therefore, cells use membrane-embedded ion pumps to counteract the leakage and maintain gradients of different ions across cell membranes, which requires energy.

The membranes of the first cells are thought to prevent the loss of polymers, but to be not particularly impermeable to small molecules and ions. It has been repeatedly noted that semipermeable primordial membranes must have been vital for the very first cells, which, in

the absence of various membrane transporters and pumps, had to rely on the diffusion of small molecules and ions across primordial, leaky membranes [30,31,223-226,269-274].

Therefore, intracellular inorganic chemistry is thought to reflect the (geo)chemistry of the environments in which the first cellular organisms formed [154,275-278], just as the low E_h of the cytoplasm reflects the reduced state of the primordial environments, see section S2.1. and [74]. In response to environmental changes, cells were not able to modify all of those enzymes that originally depended on certain substances as cofactors (see also the following Sections S2.9-S2.11). Consequently, although modern cells have colonized a wide variety of environments, they are filled with a medium similar to that in which their common ancestors lived.

This phenomenon of chemistry conservation is very important because it helps to reconstruct the habitats of the first organisms even in the absence of any geological record [154,275-277]. For example, it is safe to say that the formation of the first cells occurred under pH-neutral conditions, since the cytoplasmic pH in almost all organisms is neutral or slightly alkaline.

In particular, Archibald Macallum recognized more than a hundred years ago that potassium is more abundant than sodium in cellular tissues, in contrast to both seawater and body fluids, such as blood and lymph, see Table S2.1 and [275,279]. It was already accepted at that time that the high salt content of blood and lymph was related to the origin of multicellular organisms in seawater, see [275,279] and references therein. To explain the chemical difference between cytoplasm and seawater, Macallum proposed that "the cell...has endowments transmitted from a past almost as remote as the origin of life on earth" [275][1]. Accordingly, Macallum has suggested that the habitats of the first unicellular organisms had more potassium than sodium.

[1] Macallum wrote insightfully in this regard: "...the very earliest organisms must have been of the micellar or ultramicroscopic kind... These had as yet no nuclei, and an enclosing membrane could only have been of the most elementary character". "...as the cell is older than its media as now constituted, the relative proportions of the inorganic elements found in it are of more ancient origin than the relative proportions of the same elements found in the media, blood plasmas, and lymph..." [275].

Elsewhere, we have turned to the ubiquitous proteins common to all free-living cellular organisms [154]. Their “universal” genes set, as inferred by Koonin [20,21] and listed in Table 2, must by definition have been present in the Last Universal Cellular Ancestor (LUCA), see Section S1.4. The 87 proteins of Table S2 make up only a small fraction of the genes attributable to LUCA, the total number of which is estimated to be between 400 and 1000 [23,280-283]. However, the „complete” gene sets of LUCA, as reconstructed by different research groups, vary widely [284,285], so we have taken, as LUCA’s representatives, the 87 ubiquitous proteins whose presence in LUCA is not usually questioned.

The ubiquity of these genes suggests that their products are essential for cellular organisms. To test this assumption, we compared the ubiquitous set with the genome of the artificial minimal cell as obtained by Venter and his colleagues [286]; we wanted to assess to what extent these ubiquitous proteins are represented in this experimentally determined minimal set. We took 445 proteins (438 protein-coding genes in the syn3.0 version of the minimal genome [286] plus 7 proteins recently identified as required for a normal cell morphology and division [287]) and assigned them to Clusters of Orthologous Groups of proteins (GOGs) in the latest release of the COG database [288] with $1e^{-5}$ e-value threshold and using a set of profile HMMs available on the web-server http://boabio.belozersky.msu.ru/en/DomainAnalyser), yielding totally 365 COGs, which were used for comparison. Almost all but three universal COGs were identified in the minimal genome, indicating the indispensability of these genes. The three COGs missing from the syn3 minimal bacterial genome are marked with a hash # in Table 2.

We have checked the functional dependence of ubiquitous proteins on inorganic ions (mostly using the data from the BRENDA database [289]) and the presence of inorganic ions in the available structures, the updated version of such an analysis is presented as Table S2.2. Compared to our previous analysis [154], Table 2 provides additional data on (i) the number of available structures for each ubiquitous protein and (ii) the number of different metal ions found in these structures. This information is very important for the identification of

physiological cofactors of each ubiquitous protein since metal cofactors may be lost during protein purification or replaced by non-physiological inorganic ions from the medium.

Table S2.2. Inorganic constituents of ubiquitous proteins. (the full Excel version of the Table with additional information is provided as Supplementary File 2).
The table provides information on the inorganic constituents and cofactors of 87 orthologous groups of proteins found in all free-living organisms which, by definition, must have been present in the Last Universal Cellular Ancestor (LUCA) [20]. We took protein sequences longer than 50 aa from the PDB database (checked 13.07.2024) and assigned them to the COG database [288] using the latest set of profile HMMs for COGcollator ([290], available at http://boabio.belozersky.msu.ru/tools) and the hmmscan program (http://hmmer.org/). If two profile HMMs found overlapping hits and the overlap was longer than 5% of the longest of these two hits, we filtered out the weakest hit. To avoid any confusion, we also used only proteins that were assigned to a single COG with an e-value less than 1e-10 according to this procedure. We selected only protein chains which were assigned to the set of 87 aforementioned universal COGs. Total 71014 protein chains belonging to 7767 PDB structures were sampled.

Functional categories are given according to the COG database [288]:C — Energy production and conversion, E — Amino acid transport and metabolism, F — Nucleotide transport and metabolism, G — Carbohydrate transport and metabolism, H — Coenzyme transport and metabolism, I — Lipid transport and metabolism, J — Translation, ribosomal structure and biogenesis, K — Transcription, L — Replication, recombination and repair, D — Cell cycle control, cell division, chromosome partitioning, M — Cell wall/membrane/envelope biogenesis, N — Cell motility, O — Posttranslational modification, protein turnover, chaperones, U — Intracellular trafficking, secretion, and vesicular transport.
Other abbreviations: PPi — pyrophosphate, Pi — phosphate ; n/e — absence of an EC number due to the protein being **n**ot an **e**nzyme.
COGs which were missing from the syn3 minimal bacterial genome are marked with the hash # sign. Notably, The ribosome, as a whole requires high levels of Mg^{2+} and K^{+} ions, as well as sufficient levels of Zn^{2+} ions [291-294], see the main text for further references.

**COG**	**Funct.. Cat.**	**COG name**	**EC number (if available)**	**Functionally relevant inorganic anions**	**Functional dependence on monovalent cations**	**Functional dependence on divalent cations**	**Divalent cations in at least some structures**	**Number of structres in the PDB**
COG0636	C	FoF1-type ATP synthase, membrane subunit c/Archaeal/vacuolar-type H+-ATPase, subunit K	7.1.2.2	-	-	-	Mn^{2+}(1)	3469
COG0112	E	Glycine/serine hydroxymethyltransferase	2.1.2.1	-	-	Mg^{2+} / Ca^{2+}	Mg^{2+}(2), Ca^{2+}(1)	385
COG0125	F	Thymidylate kinase	2.7.4.9	-	-	Mg^{2+}	Mg^{2+}(59), Ca^{2+}(8)	230

COG0528	F	Uridylate kinase	2.7.4.22	-	-	Mg^{2+}	Mg^{2+}(33), Mn^{2+}(1)	221
COG1109	G	Phosphomannomutase	5.4.2.8	-	-	Mg^{2+}	Zn^{2+}(20), Mg^{2+}(24), Ca^{2+}(7)	80
COG0149	G	Triosephosphate isomerase	5.3.1.1	-	-	-	Mg^{2+}(5), Ca^{2+}(7)	622
COG0561	H	Hydroxymethylpyrimidine pyrophosphatase and other HAD family phosphatases	2.5.1.3	PPi	-	Mg^{2+}	Mg^{2+}(56), Ca^{2+}(8)	108
COG0575 /COG4589	I	CDP-diglyceride synthetase	2.7.7.41	PPi	K^{+}	Mg^{2+}	Mg^{2+}(3),	5
COG2890	J	Methylase of polypeptide chain release factors	2.1.1.297	-	-	Mg^{2+}	Mg^{2+}(4), Ca^{2+}(14), $Fe^{2+/3+}$(6)	47
COG0024	J	Methionine aminopeptidase	3.4.11.18	-	-	Co^{2+}/Ni^{2+}/ Mn^{2+}/Fe^{2+}/Zn^{2+}	Zn^{2+}(3), Mg^{2+}(4), Mn^{2+}(64), Ca^{2+}(1), $Fe^{2+/3+}$(5)	240
COG0242	J	Peptide deformylase	3.5.1.88	form ate	-	Zn^{2+}/Mn^{2+}/ Ni^{2+}/Fe^{2+}	Zn^{2+}(101), Mg^{2+}(3), $Fe^{2+/3+}$(13)	319
COG0533	J	tRNA A37 threonylcarbamoyltransferase TsaD	2.3.1.234	-	-	Zn^{2+}/Mg^{2+}/ Fe^{2+}	Zn^{2+}(7), Mg^{2+}(7), Ca^{2+}(1), $Fe^{2+/3+}$(7)	37
COG0101	J	tRNA U38,U39,U40 pseudouridine synthase TruA	5.4.99.12	-	K^{+}, NH^{4+}	-	Mg^{2+}(1),	38
COG0073	J	tRNA-binding EMAP/Myf domain	n/e	n/e	n/e	n/e		58
COG0013	J	Alanyl-tRNA synthetase	6.1.1.7	PPi	K^{+}	Mg^{2+}, Zn^{2+}	Zn^{2+}(14), Mg^{2+}(6),	68
COG0018	J	Arginyl-tRNA synthetase	6.1.1.19	PPi	K^{+}	Mg^{2+}	Mg^{2+}(1),	28
COG0124	J	Histidyl-tRNA synthetase	6.1.1.21	PPi	K^{+}	Mg^{2+}	Mg^{2+}(1),	85
COG0060	J	Isoleucyl-tRNA synthetase	6.1.1.5	PPi	K^{+}, NH^{4+}	Mg^{2+}, Zn^{2+}	Zn^{2+}(19),	31
COG0495	J	Leucyl-tRNA synthetase	6.1.1.4	PPi	K^{+}, NH^{4+}	Mg^{2+}, Zn^{2+}	Zn^{2+}(49), Mg^{2+}(36), Ca^{2+}(1)	126
COG0143	J	Methionyl-tRNA synthetase	6.1.1.10	PPi	K^{+}, NH^{4+}	Mg^{2+}, Zn^{2+}	Zn^{2+}(31), Mg^{2+}(4),	145
COG0016	J	Phenylalanyl-tRNA synthetase alpha subunit	6.1.1.20	PPi	K^{+}, NH^{4+}	Mg^{2+}, Zn^{2+}	Zn^{2+}(2), Mg^{2+}(12), Mn^{2+}(2)	68

COG0072	J	Phenylalanyl-tRNA synthetase beta subunit	6.1.1.20	PPi	K^+, NH^{4+}	Mg^{2+}, Zn^{2+}	Mg^{2+}(66), Mn^{2+}(1)	161
COG0442	J	Prolyl-tRNA synthetase	6.1.1.15	PPi	-	Mg^{2+}, Zn^{2+}	Zn^{2+}(40), Mg^{2+}(32), Mn^{2+}(2), Ca^{2+}(15)	193
COG0172	J	Seryl-tRNA synthetase	6.1.1.11	PPi	K^+	Mg^{2+}, Zn^{2+}	Zn^{2+}(18), Mg^{2+}(10), Ca^{2+}(6)	119
COG0441	J	Threonyl-tRNA synthetase	6.1.1.3	PPi	K^+, NH^{4+},Rb^+	Mg^{2+}, Zn^{2+}	Zn^{2+}(53), Mg^{2+}(2), Ca^{2+}(1)	206
COG0180	J	Tryptophanyl-tRNA synthetase	6.1.1.2	PPi	K^+	Mg^{2+}, Zn^{2+}	Mg^{2+}(18), Mn^{2+}(1), Ca^{2+}(5)	284
COG0162	J	Tyrosyl-tRNA synthetase	6.1.1.1	PPi	K^+	Mg^{2+}	Mg^{2+}(2),	67
COG0525	J	Valyl-tRNA synthetase	6.1.1.9	PPi	-	Mg^{2+}, Zn^{2+}	Zn^{2+}(1),	9
COG0081	J	Ribosomal protein L1	n/e				Mg^{2+}(5),	522
COG0244	J	Ribosomal protein L10	n/e				-	649
COG0080	J	Ribosomal protein L11	n/e				Mg^{2+}(2),	871
COG0102	J	Ribosomal protein L13	n/e				Zn^{2+}(2), Mg^{2+}(6),	1979
COG0093	J	Ribosomal protein L14	n/e				Zn^{2+}(3), Mg^{2+}(65),	1944
COG0200	J	Ribosomal protein L15	n/e				Mg^{2+}(16), Mn^{2+}(1)	1938
COG0197	J	Ribosomal protein L16/L10AE	n/e	The ribosome, as a whole requires high levels of Mg^{2+} and K^+ ions, as well as sufficient levels of Zn^{2+} ions, see the text for further references			Zn^{2+}(1), Mg^{2+}(1), Mn^{2+}(1)	1807
COG0256	J	Ribosomal protein L18	n/e				-	1866
COG0090	J	Ribosomal protein L2	n/e				Zn^{2+}(1), Mg^{2+}(106), Mn^{2+}(3)	1940
COG0091	J	Ribosomal protein L22	n/e				Mg^{2+}(1),	1980
COG0198	J	Ribosomal protein L24	n/e				Mg^{2+}(64), Mn^{2+}(2)	1955
COG0255	J	Ribosomal protein L29	n/e				-	1840
COG0087	J	Ribosomal protein L3	n/e				Mg^{2+}(78), Mn^{2+}(1)	1983
COG0088	J	Ribosomal protein L4	n/e				Zn^{2+}(3), Mg^{2+}(13), Mn^{2+}(1)	1991
COG0094	J	Ribosomal protein L5	n/e				Zn^{2+}(3), Mg^{2+}(3),	1789

COG0097	J	Ribosomal protein L6P/L9E	n/e				Zn^{2+}(3),	1827
COG0051	J	Ribosomal protein S10	n/e				Mg^{2+}(15),	1783
COG0100	J	Ribosomal protein S11	n/e				Zn^{2+}(5), Mg^{2+}(18),	1905
COG0048	J	Ribosomal protein S12	n/e				Zn^{2+}(2), Mg^{2+}(21),	1930
COG0099	J	Ribosomal protein S13	n/e				Zn^{2+}(1), Mg^{2+}(14),	1817
COG0199	J	Ribosomal protein S14	n/e				Zn^{2+}(126), Mg^{2+}(45),	1362
COG0184	J	Ribosomal protein S15P/S13E	n/e				Zn^{2+}(1), Mg^{2+}(9)	1879
COG0186	J	Ribosomal protein S17	n/e				Zn^{2+}(7), Mg^{2+}(16)	1917
COG0185	J	Ribosomal protein S19	n/e				Mg^{2+}(8)	1795
COG0052	J	Ribosomal protein S2	n/e				Zn^{2+}(17), Mg^{2+}(75)	1829
COG0092	J	Ribosomal protein S3	4.2.99.18				Mg^{2+}(23)	1764
COG0098	J	Ribosomal protein S5	n/e				Zn^{2+}(7), Mg^{2+}(98)	1885
COG0049	J	Ribosomal protein S7	n/e				Zn^{2+}(9), Mg^{2+}(12)	1899
COG0096	J	Ribosomal protein S8	n/e				Zn^{2+}(3), Mg^{2+}(29)	1842
COG0103	J	Ribosomal protein S9	n/e				Mg^{2+}(8)	1895
COG0012	J	Ribosome-binding ATPase YchF, GTP1/OBG family	3.6.5.3	Pi	K^+	Mg^{2+}*	Mg^{2+}(3)	15
COG0480	J	Translation elongation factor EF-G, a GTPase	3.6.5.3	Pi	K^+	Mg^{2+}	Mg^{2+}(41)	302
COG0050	J	Translation elongation factor EF-Tu, a GTPase	3.6.5.3	Pi	K^+	Mg^{2+}	Zn^{2+}(1), Mg^{2+}(58)	175
COG0231	J	Translation elongation factor P (EF-P)/translation initiation factor 5A (eIF-5A)	n/e		K^+		-	78
COG0361	J	Translation initiation factor IF-1	3.6.5.3	Pi	K^+	Mg^{2+}	Zn^{2+}(1), Mg^{2+}(7)	78
COG0532	J	Translation initiation factor IF-2, a GTPase	3.6.5.3	Pi	K^+	Mg^{2+}	Mg^{2+}(20)	86
COG0202	K	DNA-directed RNA polymerase, alpha subunit/40 kD subunit	2.7.7.6	PPi	K^+	Mg^{2+}, Zn^{2+}	Zn^{2+}(268), Mg^{2+}(54)	1768
COG0085	K	DNA-directed RNA polymerase, beta subunit/140 kD subunit	2.7.7.6	PPi	K^+	Mg^{2+}, Zn^{2+}	Zn^{2+}(363), Mg^{2+}(23)	1132

COG0086	K	DNA-directed RNA polymerase, beta' subunit/160 kD subunit	2.7.7.6	PPi	K^+	Mg^{2+}, Zn^{2+}	Zn^{2+}(835), Mg^{2+}(710), Mn^{2+}(8), $Fe^{2+/3+}$(1)	1171
COG0195	K	Transcription antitermination factor NusA, contains S1 and KH domains	n/e				Mg^{2+}(1)	49
COG0250	K	Transcription termination/antitermination protein NusG	n/e				$Fe^{2+/3+}$(1)	136
COG0258	L	5'-3' exonuclease Xni/ExoIX (flap endonuclease)	3.1.11.-		K^+	Mg^{2+},Mn^{2+}	Zn^{2+}(2), Mg^{2+}(18), Mn^{2+}(20), Ca^{2+}(8)	104
COG0592	L	DNA polymerase III sliding clamp (beta) subunit, PCNA homolog	2.7.7.7	-	K^+	Mg^{2+}	Mg^{2+}(3), Ca^{2+}(47)	368
COG2812	L	DNA polymerase III, gamma/tau subunits	2.7.7.7	-	K^+	Mg^{2+},Mn^{2+}	Zn^{2+}(54), Mg^{2+}(36)	92
COG0358	L	DNA primase (bacterial type)	2.7.7.101	PPi		Mg^{2+},Mn^{2+}	-	29
COG0550	L	DNA topoisomerase IA	5.6.2.1	-	K^+	Mg^{2+}, Zn^{2+}	Zn^{2+}(4), Mg^{2+}(5), Ca^{2+}(1)	52
COG0468#	L	RecA/RadA recombinase	3.5.4.B7	Pi	K^+	Mg^{2+},Mn^{2+}	Mg^{2+}(59), Mn^{2+}(1), Ca^{2+}(27)	514
COG0513	L	Superfamily II DNA and RNA helicase	3.6.4.13	Pi	-	Mg^{2+},Mn^{2+}	Zn^{2+}(4), Mg^{2+}(65), Ca^{2+}(2)	421
COG0206	D	Cell division GTPase FtsZ	3.6.5.6	Pi	-	Mg^{2+}	Mg^{2+}(13), Mn^{2+}(2), Ca^{2+}(17)	202
COG1136	M	ABC-type lipoprotein export system, ATPase component	3.6.3.-	Pi	-	Mg^{2+}	Mg^{2+}(9), Mn^{2+}(1)	73
COG0084#	N	3'->5' ssDNA/RNA exonuclease TatD	3.1.16.-	-	-	Mg^{2+}	Zn^{2+}(7), Mg^{2+}(2), Mn^{2+}(1)	20
COG1215	N	Glycosyltransferase, catalytic subunit of cellulose synthase and poly-beta-1,6-N-acetylglucosamine synthase	2.4.-.-	-	-	Mg^{2+}	Mg^{2+}(22), Mn^{2+}(23)	138

COG3118	O	Chaperedoxin CnoX, contains thioredoxin-like and TPR-like domains, YbbN/TrxSC family	1.8.1.6	-	-		Zn^{2+}(22), Mg^{2+}(5), Ca^{2+}(4), $Fe^{2+/3+}$(2)	668
COG0459#	O	Chaperonin GroEL (HSP60 family)	5.6.1.7.	Pi	K^+	Mg^{2+}, Mn^{2+}	Mg^{2+}(141), Ca^{2+}(9)	3011
COG0492	O	Thioredoxin reductase	1.8.1.9	-	-	-	Mg^{2+}(11), Ca^{2+}(4), Fe^{2+}/3+(1)	198
COG0201	U	Preprotein translocase subunit SecY	n/e	n/e			Zn^{2+}(1)	92
COG0541	U	Signal recognition particle GTPase	3.6.5.4	Pi	-	Mg^{2+}	Mg^{2+}(23), Mn^{2+}(1), Ca^{2+}(4)	123
COG0552	U	Signal recognition particle GTPase FtsY	3.6.5.4	Pi	-	Mg^{2+}	Mg^{2+}(12)	87

As follows from Table S2.2, most of the ubiquitous proteins that can be confidently traced back to LUCA are involved in ribosomal protein synthesis, see also Section S1.3. This synthesis is K^+-dependent in all organisms [293,295,296] because the proper functioning of the ribosomal apparatus requires more than 100 mM K^+ ions and their predominance over sodium ions ([K^+] > [Na^+]) [293,294]. Potassium ions serve as cofactors (i) in the peptidyl transferase center, where amino acids are linked by a peptide bond [297], (ii) in the decoding center, where tRNA recognizes the codon of mRNA [298], and (iii) in numerous proteins that assist translation [3,299]. In addition, [K^+] deficiency leads to an unspecific overall disintegration of ribosomes [298,300]. That is why all active cells contain more potassium than sodium. Usually, the concentrations of K^+ and Na^+ ions in active cells are of the order of 100 mM and 10 mM, respectively, giving a K^+/Na^+ ratio of about 10 [301]. Maintaining such a high K^+/Na^+ ratio is a costly enterprise, especially in marine environments, so cells use up to half of the available energy to maintain a tenfold excess of K^+ ions over Na^+ ions in the cytoplasm [302].

Thus, in the hope of identifying the habitats of the first cells, we have searched for environments with a high content of K^+ ions and their predominance over sodium ions

($[K^+]/[Na^+]$ ~ 10.0). We chose the high $[K^+]/[Na^+]$ ratio as a key search criterion because, unlike absolute ion concentrations, it cannot be distorted by possible evaporation of water.

In seawater, $[K^+] \ll [Na^+]$ (Table S1), so that our search criterion bluntly excludes all saline marine environments as potential hatcheries of the first cells. Rivers and lakes also usually contain more sodium than potassium [303]. The Earth’s mantle also contains ten times more sodium than potassium [304]. Furthermore, even in meteorites and asteroids, sodium predominates over potassium [305,306]. Thus, the chosen criterion $[K^+]/[Na^+]$ ~ 10.0 was a rather strong one.

The selected criteria were met only by the condensate of geothermal vapor (Table S2.3.). It stems from meteoric water (water from rain and snow) that descends through the rock until it reaches a very hot magma chamber, which heats the water up to 300-400°C and saturates it with CO_2. The heated fluid becomes lighter and rises to the surface, leaching various compounds from the rock along the way. As the rock pressure near the surface decreases, the fluid begins to boil, causing the vapor and liquid phases to separate (Figure S2.8). The vapor accumulates beneath the surface in so-called vapor-dominated zones, where it fills open fractures. From there, the vapor vents strictly upward and escapes through the hot vents, which together form a geothermal field above the vapor-dominated zone. In contrast, the liquid phase reaches the surface by penetrating between rock layers (Figure S2.8) and can erupt as geysers even outside the geothermal field.

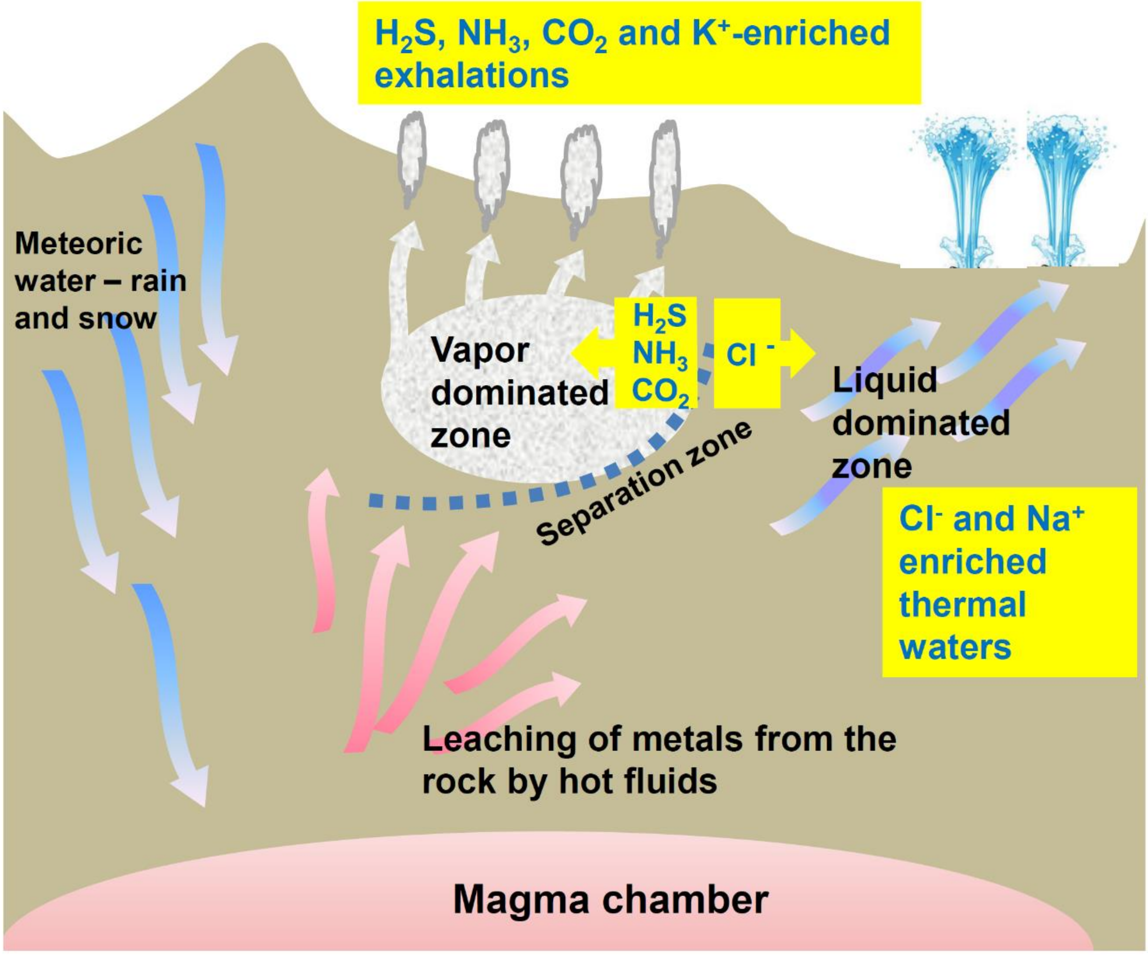

Figure S2.8. Structure of a geothermal field. The image is based on the structure of the Lassen geothermal system, USA. Image credit: USGS. A terrestrial geothermal system is fed mostly by water from rain and snow (meteoric water) that, when it is deep underground, mixes with magmatic fluids and is heated to 300 to 500 °C; such hot fluids can leach various substances from the hot rock. When heated, the water becomes lighter and rises to the surface, enriched in metal cations and such anions as Cl^-, HS^-, and CO_3^{2-}. At shallower depths, the vapor phase usually separates from the liquid phase resulting in typical zoning. This separation is not only physical but also chemical; the gaseous compounds, such as CO_2, NH_3, and H_2S, go into the vapor. Also, the large K^+ anions prefer to go into the vapor. The vapor rises upward and spreads within the rock; the hot subsurface area filled with steam and gas is called the vapor-dominated zone from which the vapor discharges through thermal springs (hot vapor vents).

The chemical properties of the liquid and vapor phases are drastically different [307,308]. The vapor usually contains more K^+ than Na^+ ions, see Table S2.3. and [154], in contrast to the liquid emissions of geysers, which contain mainly Na^+ and Cl^- ions [309]. The predominance of K^+ ions increases with the size of the geothermal field. The $[K^+]/[Na^+]$ ratio reached 32 in the steam condensate of the Larderello geothermal field in Italy [310], and 75 in the steam condensates of the world's largest geothermal field in California, USA [311].

The vapor also accumulates those substances that have affinity for the gas phase. These are compounds that can exist as gases, such as hydrogen sulfide (H_2S), CO_2 and ammonia (NH_3). Being less polar than liquid water, vapor also attracts organic molecules formed in hot rocks (see Section S2.1.).

Table S2.3. Concentration of some essential elements in the water of hot vents and the vapor condensate of the same vents, Mutnovsky volcano, Kamchatka peninsula (data from [154], expanded).

Vent number	V6-14	V6-15	V6-16	V6-17	V6-18	V6-19
water						
T(K)	94	93	89	93	96	96
pH	0.5	-0.28	0.25	-0.58	-0.09	-0.3
Cl (ppm)	6731	9447	8716	6403	5956	7288
Na (ppb)	128609	100599	79224	479027	143699	121597
K	89606	138879	22881	882720	86835	155190
B	95109	54142	35927	72639	83813	133910
P	7399	8615	6434	33689	7568	9163
Ca	279893	121911	455703	213657	334430	168640
Mg	168491	68883	118968	78648	202059	98071
Fe	384075	174308	245163	258688	446416	250982
Zn	657	324	734	471	830	439
Mn	7355	2909	3358	3942	9424	4325
Cu	4.723	4.921	<LOD	<LOD	7.189	4.165
Ni	140	89	82	96	593	67
Mo	<LOD	<LOD	<LOD	<LOD	10.3	<LOD
W	0.357	0.176	0.197	0.155	0.172	0.181
condensate						
pH	2.29	2.19	2.54	2.03	1.05	2.03
Cl (ppm)	9.81	10.77	5.23	4.38	10.42	1.15
Na (ppb)	5427	128	798	14.9	50.7	3082
K	15787	45.5	2317	22.6	37.6	8399
B	2635	84.4	1092	185	215	4296
P	18.0	5.2	11.8	2.0	6.6	4.3
Ca	567	219	424	30.0	90.0	289
Mg	141.0	48.7	139	2.483	15.5	24.5
Fe	760	216	799	10.7	155	99.4
Zn	19.0	3.4	12.8	6.0	6.9	10.8
Mn	9.0	2.3	7.0	0.1	1.9	2.3
Cu	3.08	0.59	1.97	0.15	0.82	0.39
Ni	16.2	0.4	9.2	0.2	1.3	0.7
Mo	0.046	0.014	0.044	0.002	0.013	0.028
W	0.006	0.009	0.003	0.006	0.067	0.002

The main reason why the vapor-dominated geothermal fields have not usually been considered as suitable hatcheries for the early life is that the pools and puddles in such fields are highly acidic (with pH values reaching −0.5, see Table S2.3.) and thus inhospitable to life. The reason for the high acidity is the discharge of large amounts of H_2S, which is promptly oxidized by atmospheric oxygen to strong sulfuric acid. However, in the absence of oxygen on the primordial Earth, the geochemistry of the geothermal fields must have been quite different. The pH of the vapor condensate must have been neutral or slightly alkaline; H_2S and CO_2 ascending with the vapor are weak acids, and their acidity must have been balanced by the interaction with basic rocks and concurrent ascending NH_3.

Furthermore, at neutral pH, silica must have precipitated around the thermal springs not as mud as today [153,154], but as porous silicate minerals such as sinters and clays, in analogy to today's near-neutral hot springs [312].

Concurrent studies at several sites around the world have revealed the similarity of finger-like (digitate), porous sinter deposits around thermal springs [313]. Their pores are usually inhabited by microbes, see [314-316]. It is tempting to think that similar porous sinter deposits may have formed in the Hadean. Lynne and her colleagues have shown that the sinter deposits evolve with time [317]. Particularly remarkable is the conversion of sinter nanospheres into sharp, 1D blades.

From our data, we have previously proposed that the first cells may have emerged in pools of cold geothermal condensate. In the absence of atmospheric oxygen, the inorganic chemistry of these pools must have been similar to that of cellular cytoplasm, see [79,154] for details.

In the same papers, we have explicitly noted that the compounds with specific affinity for geothermal vapor are otherwise considered to be either the building blocks for abiogenic syntheses of the first biomolecules (H_2S, NH_3, simple organics) or the catalysts of these syntheses such as borate (see Table S2.3. and [79,154]). Based on this correlation, we speculated that anoxic geothermal fields may have served as the cradles of life itself, with

geothermal pools sheltering and nourishing the first, pre-cellular life forms until they evolved into the first cells [79,154].

The evolution of life from the very first self-copying molecules to the stage of protocells in the same habitats is the most parsimonious scenario: otherwise one would have to imagine separate mechanisms for the transfer of the first, still vulnerable, pre-cellular organisms from elsewhere to the geothermal fields and for their accommodation in new habitats.

Our work [79,154] had prompted geologists to look for vestiges of anoxic geothermal fields. Van Kranendonk and his colleagues have discovered them in the 3.48 Ga old Dresser Formation of the Pilbara Craton, Western Australia [318-321], i.e. in the same location where the oldest evidence for life on Earth had previously been found [322-326]. Analysis of the Dresser Formation deposits revealed the remnants of hot springs surrounded by sinter terracettes. The mineral assemblage included geyserite, kaolinite/illite, and borate-bearing tourmaline, see Figure S2.9. [318,320,321]. The stromatolites, formed by microbial communities dwelling in basins of these geothermal fields 3.48 Ga ago, are characterized by alternating layers of zinc and nickel [319,321]. These groundbreaking findings indicate that the anoxic geothermal fields existed and were most likely inhabited as early as 3.48 Ga ago.

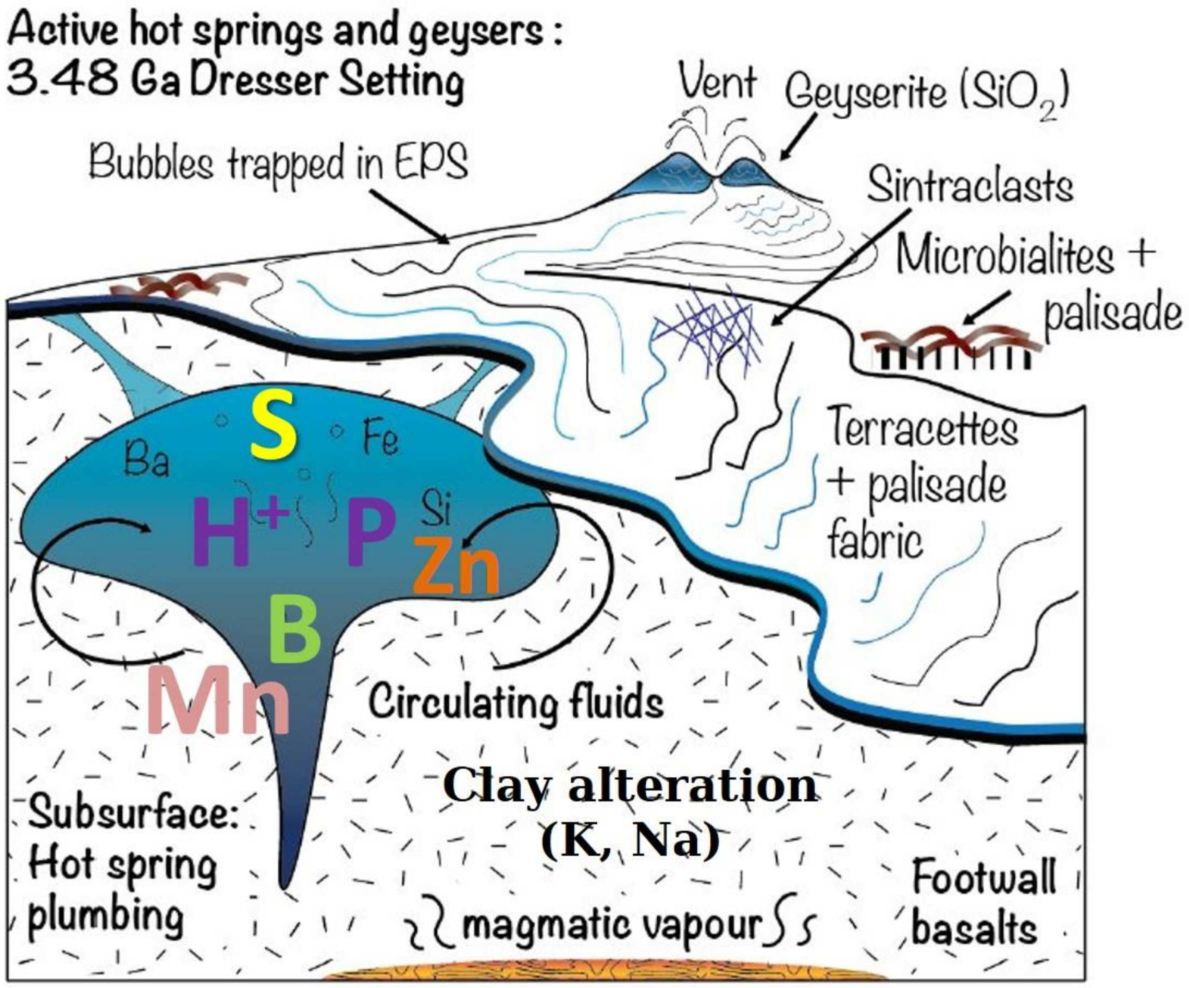

Fig. S2.9. Schematic representation of the Dresser hot spring system [321]; the image has been taken from the webpage of the Pheasant Memorial Laboratory in Misasa, Japan, https://pml.misasa.okayama-u.ac.jp/pages/projects/origin-of-life-2021.php.

2.2.9. Paradox of the high Mg^{2+} to Ca^{2+} ratio in the cell

Paradoxically, the intracellular concentration of free Ca^{2+} ions is typically 10^5 times lower than that of Mg^{2+} (~ 10^{-7} M vs. ~ 10^{-2} M), although their concentrations outside the cells are usually comparable (Table S2.1.). Magnesium makes up about 20% of the Earth's core; not surprisingly, many enzymes attributed to the LUCA use Mg^{2+} ions as cofactors (see Table S2.2.). Intracellular Mg levels do not differ dramatically from those in the cell environment (Table S2.1.), so maintaining physiological levels of Mg^{2+} is not very costly. In contrast, pumping the in-leaking Ca^{2+} ions out of the cell costs energy.

The principle of chemistry conservation implies that low intracellular Ca^{2+} levels may reflect the environmental conditions under which the very first cells may have formed. Why, then, were Ca^{2+} levels in the habitats of the first cells ~10^4 times lower than in today's environments?

A possible solution to this $[Mg^{2+}]/[Ca^{2+}]$ paradox can be seen in the recent data of Mustaev and his colleagues [327]. They investigated whether the involvement of the Mg^{2+} ion as a cofactor in the thirteen evolutionarily ancient RNA and DNA processing enzymes is related to some specific property of Mg^{2+} ions – or simply to their availability in the environment in which these enzymes originated. To answer this question, the authors measured the activities of these 13 enzymes in the presence of Mg^{2+}, Mn^{2+}, Co^{2+}, Zn^{2+}, Cu^{2+}, Ni^{2+}, Cd^{2+}, Ca^{2+}, and Fe^{2+}, respectively. Each of the enzymes studied could be activated by one or more cations other than Mg^{2+}. The authors then evaluated the solubility of all these divalent cations (at 10 mM) in the presence of common anions such as phosphate (PO_4^{3-}) and carbonate (CO_3^{2-}), taken at concentrations of 20 mM and 10 mM, respectively, as the authors anticipated for primordial environments. In these experiments, only the Mg^{2+} salts remained soluble. The authors concluded that all of these enzymes use Mg^{2+} as a cofactor because it was the only divalent cation that remained soluble at high primordial carbonate and/or phosphate levels.

It is noteworthy that Ca^{2+} ions precipitated both phosphate and carbonate ions in the experiments of Mustaev and his colleagues. Based on their data, it is tempting to suggest that the current intracellular concentration of Ca^{2+} corresponds to the concentration of free Ca^{2+} ions in the habitats of the first cells. These concentrations must have been much lower than today because of higher concentrations of natural Ca^{2+}-precipitating anions, especially phosphate and water-dissolved (bi)carbonate in equilibrium with the CO_2-rich atmosphere. Remarkably, the difference in solubility of Mg and Ca carbonates is exploited in industry when mixed mine wastewater is treated with pressurized CO_2 to separate soluble Mg-containing brine from insoluble $CaCO_3$ [328].

From the data of Mustaev and his colleagues, the concentration of bicarbonate in the habitats of the first cells can be estimated in the order of tens of millimoles, i.e. at least 10-100 times higher than in tpresent-day water basins. This estimate is consistent with other estimates in the literature [329,330].

S2.10. Abundance of Zn^{2+} ions and absence of Fe^{2+} ions in the evolutionarily oldest enzymes (the zinc/iron paradox).

Many proteins use zinc ions as cofactors. Zn^{2+} ions can serve both as catalytic cofactors and as structural elements that stabilize the protein folds by linking several amino acid residues [331]. Zn appears to be the only metal whose ions are routinely used by proteins in just such a structural role [332,333]. Furthermore, the ubiquitous proteins common to all free-living cellular organisms and thought to be present in the LUCA mostly use Zn as a transition metal cofactor, see Table S2.2. and [154]. In addition, Zn correlates with the oldest protein folds and is the most abundant transition metal found in the RNA structures, see [154,278,333,334] and references therein.

The total concentration of Zn^{2+} ions in modern cells is about $2\text{-}4 \times 10^{-4}$ M, many orders of magnitude higher than in the environment (Table S2.1.). The accumulation of Zn^{2+} ions inside the cells demands sophisticated ion pumps, ion-tight membranes, and zinc-storing proteins (metallothioneins) [335-337]. Since the first cells were unlikely to have all these "gears", they could only recruit Zn^{2+} ions if they lived in a Zn-rich environment. The recruitment of Zn^{2+} ions as mere structural elements(!) indicates the high abundance of Zn^{2+} ions around the first organisms.

However, this inferred abundance of Zn^{2+} ions in primordial environments conflicts with the poor solubility of common zinc salts (see also the previous Section S2.9. and [327]). Accordingly, the concentration of free Zn^2 in primordial anoxic waters has been estimated to be as low as $\leq 10^{-12}$ M [261,262]. How, then, could the first cells have gained access to the Zn^{2+} ions?

A natural process involving the continuous release of Zn^{2+} ions is one possibility. In this case, biopolymers may have had a chance to capture Zn^{2+} ions before they precipitated as inorganic salts, such as ZnS (sphalerite) or $ZnCO_3$ (smithsonite). Consequently, we have attributed the steady release of Zn^{2+} ions to the photochemical properties of primordial geothermal zinc sulfide (ZnS) precipitates [78,278,334,338]. Crystals of ZnS are semiconductors, in which UV light causes a separation of electric charges. The resulting charge separated states can store the energy of the absorbed light for hours. This unique property manifests itself in phosphorescence (afterglow), so that ZnS – widely known as "phosphor" – is used in numerous devices, from various types of displays to 'glow-in-the-dark' toys [339-341]. By accumulating two or more charge separated states, natural ZnS crystals can serve as potent multielectron reducing agents with redox potentials below –1.0 V [75,76]. In particular, illuminated nanocrystals of ZnS, which exhibit the properties of quantum dots (QD) [341,342], can reduce CO_2 to formate with quantum efficiency of up to 80% [343-348], see Figure S2.10. This efficiency is higher than that of the chlorophyll-based photosynthesis of green plants.

In general, ZnS is considered to be the most potent photocatalyst of natural origin. Similar but weaker photochemical activity is inherent in MnS and CdS crystals, as well as in zinc oxide, ZnO, and titanium oxide, TiO_2 [75,76,349]. Of these compounds, CdS crystals are the least biologically relevant because Cd occurs in nature only as a minor admixture to Zn.

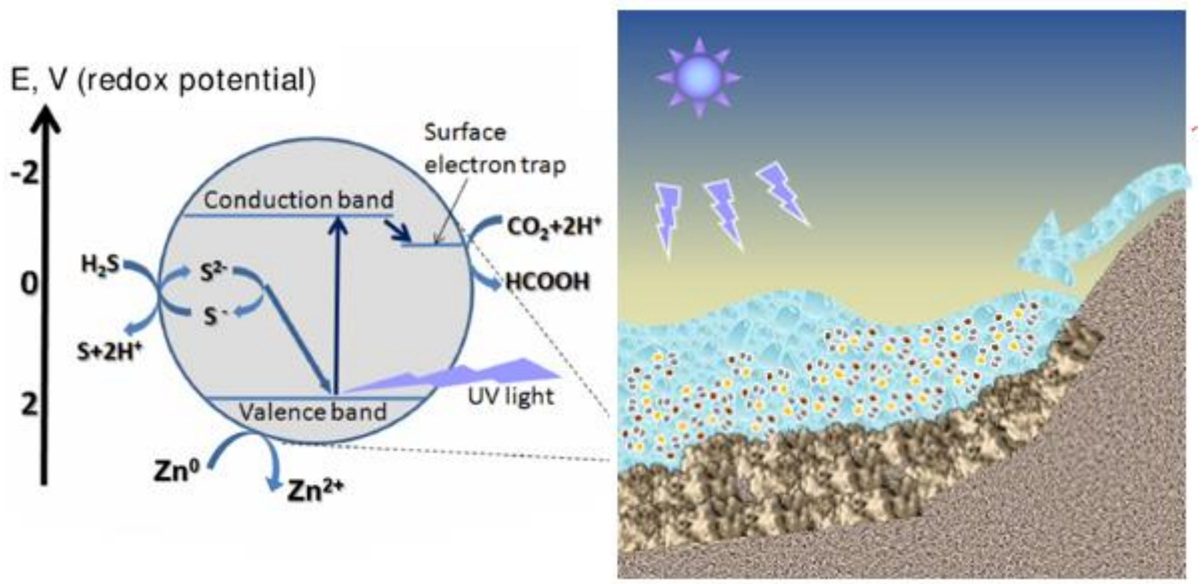

Figure S2.10. Abiotic ZnS-mediated photosynthesis in sub-aerial, illuminated settings (from [78], modified). Right: Diverse nanoparticles, including particles of ZnS, in a Hadean geothermal pool. Left: A schematic representation of the reactions within a photosynthesizing ZnS nanoparticle, as combined with an energy diagram. Initially the absorption of an UV results in the promotion of electrons from the valence band to the conduction band (large blue arrow). The electrons then migrate within the crystal until they are trapped at the surface; the trapped electrons can reduce a CO_2 molecule either via two one-electron transfers [344] or, possibly, in a concerted two-electron reaction. The remaining electron vacancy (hole) is initially reduced by the S_2^- ion of the crystal; however, the ultimate electron equilibration requires external electron donors, e.g. H_2S.

Notably, ZnS-, MnS- and CdS-mediated photochemical reduction is accompanied by the disruption of photosynthesizing crystals and the release of Zn^{2+}, Mn^{2+}, and Cd^{2+} ions, respectively, see Figure S2.12 and [344,350]. With this property, we have explained the exclusive recruitment of Zn^{2+} and Mn^{2+} ions by the first proteins – assuming that the habitats of their hosts contained Mn and Zn sulfides and were accessible to the UV-rich radiation of the young Sun (see Section S2.6.). We have called this early Zn-dependent step in the evolution of life the 'Zinc World', whose past existence is evidenced by the almost exclusive dependence of the evolutionarily oldest proteins on zinc as a transition metal cofactor, as documented by Table S2.2. [78,154,278,334,338].

However, the geochemistry of these primordial ZnS-rich habitats has remained obscure. In nature, large amounts of ZnS, along with other metal sulfides, are found only at the sites of current or ancient geothermal activity, where the leached metals are/were brought to the

surface by very hot geothermal fluids. On the one hand, the involvement of geothermal fluids corresponds to the tentative origin of the first cells in anoxic geothermal fields considered above in Section S2.8. On the other hand, it has remained unclear whether such ZnS-rich systems existed en masse during the Hadean.

We have also been unable to explain convincingly why the evolutionarily oldest proteins avoid using Fe^{2+} ions as cofactors (Table S2.2). The absence of Fe^{2+} ions in such proteins is all the more paradoxical because the Earth's crust and mantle contain a thousand time more iron than zinc. Geothermal settings, although they accumulate more ZnS and MnS than unaltered rocks, are always dominated by Fe^{2+} ions which are also prevalent in geothermal vapor, see Table S2.3.

Furthermore, the sulfides, phosphates and carbonates of divalent iron are moderately soluble. Consequently, the equilibrium concentration of Fe^{2+} in the primordial anoxic waters has been estimated to be as high as 10^{-5} M, compared to estimates of ≤ 10^{-12} M for Zn^{2+} [261,262].

Nevertheless, the found absence of Fe^{2+} ions in the evolutionarily oldest proteins [154,278,334] was confirmed by the data from David and Alm on the delayed recruitment of iron as an enzyme cofactor. These authors "mapped the evolutionary history of 3,983 gene families across the three domains of life onto a geological timeline" [351]. One of their findings was that the mass appearance of Fe^{2+}-containing enzymes occurred about 100-200 million years after the appearance of the very first enzymes. To these first enzymes, David and Alm assigned the ubiquitous nucleotide- and phosphate-processing enzymes, many of which are zinc-dependent, see Table S2.2.

A tentative solution to the Zn/Fe paradox is provided by the scenario proposed in Section 3 of the main text. The scenario clarifies (i) how the primordial Earth may have become covered by Zn-enriched protocrust, (ii) why and how the evolutionarily oldest proteins predominantly recruited Zn^{2+} ions as transition metal cofactors, and (iii) why the recruitment of Fe^{2+} ions by enzymes may have occurred with a delay of about 100 million years as evidenced in [351].

2.2.11. The Phosphate Paradox

The total concentration of phosphate in cells is on the order of 10 mM (Table 1). Since phosphate groups serve as linkers in RNA and DNA, the formation of the first RNA-like polymers, which are thought to have preceded the origin of cells (see Sections S1.3 and S2.6.), must have occurred in phosphate-rich habitats. However, the concentration of phosphate ions in natural waters such as lakes or oceans rarely exceeds 1 μM because phosphate is precipitated by most divalent metals (see Table S2.1., section S2.9. and [327]). Accordingly, modern organisms invest energy and use specific membrane transporters to accumulate phosphate in their cells.

Thus, any origin of life scenario must provide a plausible and abundant source of soluble phosphorus compounds for the first, not yet sophisticated organisms.

As early as 1955, Gulick argued that reduced phosphorus species such as hypophosphite (PO_2^{3-}) and/or phosphite (PO_3^{3-}), which are ~1000 times more soluble than phosphate (PO_4^{3-}), could have been abundant under primordial reduced conditions [352]; this line of thought was further developed in the following years [353-358]. The shortcoming of the original hypothesis was the lack of a clear source for these reduced phosphorus compounds. Both hypophosphite and phosphite have very low redox potentials, well below the low-potential stability limit of water (cf Figure S2.1 and S2.11). Therefore, if dissolved in water, these compounds must have been oxidized by water protons, even in the absence of atmospheric oxygen. Consequently, some origin of life scenarios assumed that reduced phosphorus could have resided in the solid state as a constituent of the relevant minerals and could have been mobilized - as a substrate for primordial reactions - when these minerals were dissolved, e.g. by rainwater, see e.g. [192].

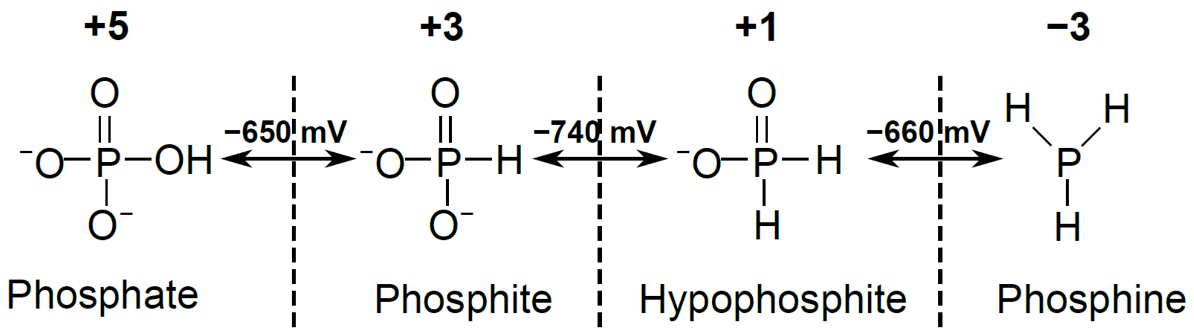

Figure S2.11. Chemical structures of phosphorus compounds and their redox transformations (redrawn with modifications from [359]). Numbers above each compound indicate the oxidation state of phosphorus. The numbers over the arrows indicate the E_0^7 values or respective redox reactions.

One conceivable possibility is a continuous supply of reduced forms of phosphorus from geothermal systems. The phosphorus content of geothermal vapor is, generally, relatively high, see Table S2.3 and [118,309]. The redox state of such geothermally delivered phosphorus was first determined by Foster and her colleagues for the pristine geothermal pool at Hot Creek Gorge near Mammoth Lakes, CA, which the authors considered "representative of early Earth" [360]. The measurement revealed almost equal amounts of phosphite and phosphate, albeit at low levels of ~ 0,05 µM. The pool at Hot Creek Gorge was described as bicarbonate-rich [360], implying a contribution from geothermal vapor. The parity of phosphite and phosphate amounts [360,361] may be due to the state of phosphorus in a hot geothermal vapor being $(PO_2)_n$ [362-364]. Hydrolysis of such phosphorus species by water yields a mixture of hypophosphite, phosphite and phosphate. Apparently, the redox conditions in the vapor zone beneath Hot Creek Gorge are still sufficiently reducing to maintain phosphorus in a partially reduced state. Later, reduced species of phosphorus of unclarified origin have been also discovered in other environmental samples [357,365].

The discovery of highly soluble phosphite justifies the existence of enzymes capable of hypophosphite and phosphite oxidation in many prokaryotes [366].

Since highly soluble reduced phosphorus species are still being supplied at sites of present-day geothermal activity, their supply by geothermal/volcanic systems of the anoxic primordial Earth seems quite plausible. Further support for this view is provided in Section 3 of the main text.

2.2.12. The faint young Sun paradox

As last, we consider the faint young Sun paradox. While some of the paradoxes considered above have not been formulated as such before, the faint young Sun paradox is well recognized, see e.g. [367,368] for recent reviews. As early as 1958 Hoyle showed by applying the physical principles governing the structure and evolution of other stars to our Sun, Hoyle showed that the luminosity of the Sun must have changed over time, with the young Sun being considerably less luminous than today [367,369]. The consequences for life on Earth were recognized by Sagan and Mullen in 1972, who noted that at a solar luminosity of 60-70% of today's the average temperature at the Earth's surface must have been about -25°C, well below the freezing point of water (at today's atmospheric pressure, see [370]). Assuming that "liquid water is almost certainly necessary for the origin of life" [370], these authors considered possible means of maintaining the surface temperatures high enough to keep water in a liquid state. As a solution, they proposed a greenhouse atmosphere with ammonia as the dominant greenhouse gas.

While the analysis by Sagan and Mullen was limited to modeling the Earth's surface temperature as a function of atmospheric composition and solar luminosity [370], a more sophisticated but less well-known model by Moroz and Mukhin, while addressing the same problem, considered the atmospheres of Earth, Venus and Mars in their interaction with geological processes [371,372]. In the following, we will take a closer look at this modeling, as its results are used in the evolutionary reconstruction presented in Section 3 of the main text.

Moroz and Mukhin assumed an initially dry Earth with an atmospheric pressure of 10^{-3} atm. With such a thin atmosphere, the Earth's surface temperature was determined by radiation from the faint young Sun. For these conditions, average surface temperatures around -50°C were calculated, in agreement with earlier estimates by Sagan and Mullen [370]. The temperature must have risen gradually due to the warming of the Sun and the greenhouse effect of the atmosphere built up through volcanic outgassing. The authors argued that ammonia, originally proposed by Sagan and Mullen as a primordial greenhouse gas, could not perform this function because of its photolability in the UV range. Instead, they proposed water vapor and CO_2, the main photostable products of volcanic outgassing, as greenhouse factors.

Moroz and Mukhin proposed that CO_2 must have accumulated in the atmosphere, increasing the greenhouse effect (Figure S2.12), while the water vapor would have initially frozen as ice and snow and, by increasing the Earth's albedo, must have retarded the warming of the Earth; the frozen water could not have contributed to the buildup of atmospheric pressure.

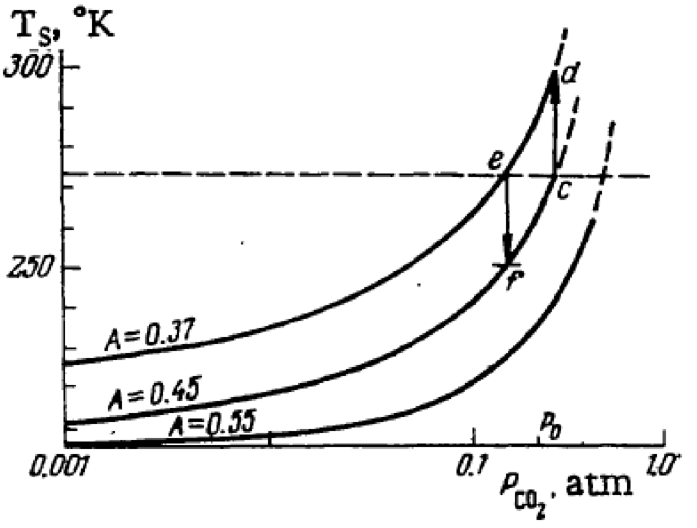

Fig. S2.12. Mean temperature T_S of the Earth's surface as a function of the CO_2 abundance in the atmosphere at three realistic albedo values A, as calculated in [372]. The dashed line indicates 273K.

Moroz and Mukhin suggested that the greenhouse effect from the accumulation of CO_2 in the atmosphere must have eventually enabled the frozen water to melt. The liquid water may have

captured the dissolved CO_2 through the process of chemical weathering. When dissolved in water, CO_2 gets converted to carbonic acid (HCO_3^- or CO_3^{2-}, depending on the pH), which leaches out of the rocks lining the water basins. The released metal cations, mainly Ca^{2+} ions, interact with (bi)carbonate ions to form insoluble precipitating carbonates and thus sequester CO_2. However, chemical weathering must have continued only until the CO_2 concentration dropped to the point where the water froze. As a result, after its initial rise, atmospheric CO_2 must have been maintained at a constant level that provided average global temperatures around the melting point of water. Moroz and Mukhin suggested that such a negative feedback loop must have slowed down both the rise in atmospheric pressure and the development of the greenhouse, which implied a rather mild, if not frosty, climate throughout the Hadean [371,372].

Moroz and Mukhin calculated that the average temperature must not have exceeded the melting point of water until solar luminosity reached about 0.8 of its present value, about 4 Gy ago. The authors predicted that even after this point, the emergent life, by consuming CO_2, could interfere with the constantly operating CO_2 feedback loop and cause further glaciation episodes. Evidence for such glaciation periods was later found, see [373] for a recent review.

Notably, Moroz and Mukhin were the first to identify the CO_2 feedback loop as an important climate factor; similar ideas were later popularized by Walker and his colleagues [374]. This feedback loop still maintains the Earth's mild climate and is considered the key element in current climate models.

It is regrettable that the papers of Moroz and Mukhin did not receive the attention they deserved when published. Moroz and Mukhin did not specifically promote their modeling results as they were busy organizing the exploration of Venus by the Soviet "Venera" landers in the 70s and 80s [375-377]. Still, the unsurpassed success of that program, in which eight "Veneras" landed safely and sent back data and images despite temperatures of ~500°C and pressures of ~100 bar, proves the expertise of Moroz and Mukhin in planetary atmospheres.

Moroz and Mukhin did not consider the presence of a liquid ocean as a prerequisite for the origin of life because they favored the origin of life around terrestrial volcanic systems [52,53,371,372]. Owing to geothermal activity, liquid water must have been present around volcanoes independently of climate. Accordingly, it was acceptable to Moroz and Mukhin that the average temperature on young Earth could initially have been well below the freezing point of water.

As argued above in Section S2.4, liquid water in large quantities was the last thing needed for the emergence of life. Therefore, from the viewpoint of the origin of life in terrestrial volcanic/geothermal systems, which we share, the faintness of the young Sun must have even promoted the emergence of the first organisms by eliminating liquid water through its freezing. Thus, we see no reason to regard the well-publicized "faint young sun paradox" as a paradox.

References

1. Crick, F. Central dogma of molecular biology. *Nature* **1970**, *227*, 561-563.
2. Cramer, W.A.; Knaff, D.B. *Energy Transduction in Biological Membranes: A Textbook of Bioenergetics*; Springer-Verlag: 1990.
3. Kozlova, M.I.; Shalaeva, D.N.; Dibrova, D.V.; Mulkidjanian, A.Y. Common Mechanism of Activated Catalysis in P-loop Fold Nucleoside Triphosphatases-United in Diversity. *Biomolecules* **2022**, *12*, doi:10.3390/biom12101346.
4. Lindahl, T. The Croonian Lecture, 1996: endogenous damage to DNA. *Philos Trans R Soc Lond B Biol Sci* **1996**, *351*, 1529-1538, doi:10.1098/rstb.1996.0139.
5. Lindahl, T. Irreversible heat inactivation of transfer ribonucleic acids. *J Biol Chem* **1967**, *242*, 1970-1973.
6. Watson, J.D.; Crick, F.H. Genetical implications of the structure of deoxyribonucleic acid. *Nature* **1953**, *171*, 964-967.
7. Yusupov, M.M.; Yusupova, G.Z.; Baucom, A.; Lieberman, K.; Earnest, T.N.; Cate, J.H.D.; Noller, H.F. Crystal structure of the ribosome at 5.5 angstrom resolution. *Science* **2001**, *292*, 883-896.
8. Steitz, T.A.; Moore, P.B. RNA, the first macromolecular catalyst: the ribosome is a ribozyme. *Trends Biochem. Sci.* **2003**, *28*, 411-418.
9. Wadsworth, G.M.; Zahurancik, W.J.; Zeng, X.; Pullara, P.; Lai, L.B.; Sidharthan, V.; Pappu, R.V.; Gopalan, V.; Banerjee, P.R. RNAs undergo phase transitions with lower critical solution temperatures. *Nat Chem* **2023**, *15*, 1693-1704, doi:10.1038/s41557-023-01353-4.
10. Leamy, K.A.; Assmann, S.M.; Mathews, D.H.; Bevilacqua, P.C. Bridging the gap between in vitro and in vivo RNA folding. *Q Rev Biophys* **2016**, *49*, e10, doi:10.1017/S003358351600007X.
11. Yu, Y.; Gim, S.; Kim, D.; Arnon, Z.A.; Gazit, E.; Seeberger, P.H.; Delbianco, M. Oligosaccharides Self-Assemble and Show Intrinsic Optical Properties. *J Am Chem Soc* **2019**, *141*, 4833-4838, doi:10.1021/jacs.8b11882.
12. Israelachvili, J.N.; Mitchell, D.J.; Ninham, B.W. Theory of self-assembly of lipid bilayers and vesicles. *Biochim Biophys Acta* **1977**, *470*, 185-201, doi:0005-2736(77)90099-2 [pii].
13. Fox, G.E.; Magrum, L.J.; Balch, W.E.; Wolfe, R.S.; Woese, C.R. Classification of methanogenic bacteria by 16S ribosomal RNA characterization. *Proc Natl Acad Sci U S A* **1977**, *74*, 4537-4541.

14. Woese, C.R.; Fox, G.E. Phylogenetic structure of the prokaryotic domain: the primary kingdoms. *Proc Natl Acad Sci U S A* **1977**, *74*, 5088-5090.
15. Woese, C. The universal ancestor. *Proc Natl Acad Sci U S A* **1998**, *95*, 6854-6859.
16. Eme, L.; Tamarit, D.; Caceres, E.F.; Stairs, C.W.; De Anda, V.; Schon, M.E.; Seitz, K.W.; Dombrowski, N.; Lewis, W.H.; Homa, F.; et al. Inference and reconstruction of the heimdallarchaeial ancestry of eukaryotes. *Nature* **2023**, *618*, 992-999, doi:10.1038/s41586-023-06186-2.
17. Krupovic, M.; Dolja, V.V.; Koonin, E.V. The virome of the last eukaryotic common ancestor and eukaryogenesis. *Nat Microbiol* **2023**, *8*, 1008-1017, doi:10.1038/s41564-023-01378-y.
18. Woese, C.R.; Kandler, O.; Wheelis, M.L. Towards a natural system of organisms: proposal for the domains Archaea, Bacteria, and Eucarya. *Proc Natl Acad Sci U S A* **1990**, *87*, 4576-4579.
19. Cox, C.J.; Foster, P.G.; Hirt, R.P.; Harris, S.R.; Embley, T.M. The archaebacterial origin of eukaryotes. *Proc Natl Acad Sci U S A* **2008**, *105*, 20356-20361, doi:10.1073/pnas.0810647105.
20. Koonin, E.V. How many genes can make a cell: the minimal-gene-set concept. *Annu Rev Genomics Hum Genet* **2000**, *1*, 99-116.
21. Koonin, E.V. Comparative genomics, minimal gene-sets and the last universal common ancestor. *Nat Rev Microbiol* **2003**, *1*, 127-136.
22. Charlebois, R.L.; Doolittle, W.F. Computing prokaryotic gene ubiquity: rescuing the core from extinction. *Genome Res* **2004**, *14*, 2469-2477.
23. Forterre, P. The Last Universal Common Ancestor of Ribosome-Encoding Organisms: Portrait of LUCA. *J Mol Evol* **2024**, *92*, 550-583, doi:10.1007/s00239-024-10186-9.
24. Pereto, J.; Lopez-Garcia, P.; Moreira, D. Ancestral lipid biosynthesis and early membrane evolution. *Trends Biochem Sci* **2004**, *29*, 469-477.
25. Kozlova, M.I.; Bushmakin, I.M.; Belyaeva, J.D.; Shalaeva, D.N.; Dibrova, D.V.; Cherepanov, D.A.; Mulkidjanian, A.Y. Expansion of the "Sodium World" through Evolutionary Time and Taxonomic Space. *Biochemistry (Mosc)* **2020**, *85*, 1518-1542, doi:10.1134/S0006297920120056.
26. Forterre, P.; Philippe, H. The last universal common ancestor (LUCA), simple or complex? *Biological Bulletin* **1999**, *196*, 373-375.
27. Forterre, P.; Philippe, H. Where is the root of the universal tree of life? *Bioessays* **1999**, *21*, 871-879.
28. Koonin, E.V.; Krupovic, M.; Ishino, S.; Ishino, Y. The replication machinery of LUCA: common origin of DNA replication and transcription. *BMC Biol* **2020**, *18*, 61, doi:10.1186/s12915-020-00800-9.
29. Deamer, D.W. Role of Amphiphilic Compounds in the Evolution of Membrane-Structure on the Early Earth. *Orig. Life Evol. Biosph.* **1986**, *17*, 3-25.
30. Deamer, D.W. The first living systems: a bioenergetic perspective. *Microbiol. Mol. Biol. Rev.* **1997**, *61*, 239-261.
31. Dibrova, D.V.; Chudetsky, M.Y.; Galperin, M.Y.; Koonin, E.V.; Mulkidjanian, A.Y. The role of energy in the emergence of biology from chemistry. *Orig Life Evol Biosph* **2012**, *42*, 459-468, doi:10.1007/s11084-012-9308-z.
32. Koonin, E.V. The cosmological model of eternal inflation and the transition from chance to biological evolution in the history of life. *Biol. Direct* **2007**, *2*, 15.
33. Benner, S.A. Paradoxes in the origin of life. *Orig Life Evol Biosph* **2014**, *44*, 339-343, doi:10.1007/s11084-014-9379-0.
34. Marshall, M. The water paradox and the origins of life. *Nature* **2020**, *588*, 210-213.
35. Benner, S.A. Rethinking nucleic acids from their origins to their applications. *Philos Trans R Soc Lond B Biol Sci* **2023**, *378*, 20220027, doi:10.1098/rstb.2022.0027.
36. Bratsch, S.G. Standard electrode potentials and temperature coefficients in water at 298.15 K. *Journal of Physical and Chemical Reference Data* **1989**, *18*, 1-21.
37. Nelson, D.L.; Lehninger, A.L.; Cox, M.M. *Lehninger principles of biochemistry*; Macmillan: 2008.
38. Saji, V.S.; Lee, C.W. Molybdenum, molybdenum oxides, and their electrochemistry. *ChemSusChem* **2012**, *5*, 1146-1161.
39. Habisreutinger, S.N.; Schmidt-Mende, L.; Stolarczyk, J.K. Photocatalytic reduction of CO2 on TiO2 and other semiconductors. *Angewandte Chemie International Edition* **2013**, *52*, 7372-7408.
40. Yates, N.D.J.; Fascione, M.A.; Parkin, A. Methodologies for “wiring” redox proteins/enzymes to electrode surfaces. *Chemistry–A European Journal* **2018**, *24*, 12164-12182.

41. Milton, R.D.; Minteer, S.D. Nitrogenase bioelectrochemistry for synthesis applications. *Accounts of chemical research* **2019**, *52*, 3351-3360.
42. Frost, B.R. Introduction to oxygen fugacity and its petrologic importance. In *Oxide minerals*; De Gruyter: 2018; pp. 1-10.
43. Liu, J.; Chakraborty, S.; Hosseinzadeh, P.; Yu, Y.; Tian, S.; Petrik, I.; Bhagi, A.; Lu, Y. Metalloproteins containing cytochrome, iron–sulfur, or copper redox centers. *Chemical reviews* **2014**, *114*, 4366-4469.
44. Blankenship, R.E. *Molecular Mechanisms of Photosynthesis*; Wiley: 2021.
45. Berg, I.A. Ecological aspects of the distribution of different autotrophic CO2 fixation pathways. *Appl Environ Microbiol* **2011**, *77*, 1925-1936, doi:10.1128/AEM.02473-10.
46. Keller, M.A.; Turchyn, A.V.; Ralser, M. Non-enzymatic glycolysis and pentose phosphate pathway-like reactions in a plausible Archean ocean. *Mol Syst Biol* **2014**, *10*, 725, doi:10.1002/msb.20145228.
47. Keller, M.A.; Zylstra, A.; Castro, C.; Turchyn, A.V.; Griffin, J.L.; Ralser, M. Conditional iron and pH-dependent activity of a non-enzymatic glycolysis and pentose phosphate pathway. *Sci Adv* **2016**, *2*, e1501235, doi:10.1126/sciadv.1501235.
48. Ralser, M. An appeal to magic? The discovery of a non-enzymatic metabolism and its role in the origins of life. *Biochem J* **2018**, *475*, 2577-2592, doi:10.1042/BCJ20160866.
49. Muchowska, K.B.; Varma, S.J.; Chevallot-Beroux, E.; Lethuillier-Karl, L.; Li, G.; Moran, J. Metals promote sequences of the reverse Krebs cycle. *Nat Ecol Evol* **2017**, *1*, 1716-1721, doi:10.1038/s41559-017-0311-7.
50. Muchowska, K.B.; Varma, S.J.; Moran, J. Nonenzymatic metabolic reactions and life's origins. *Chemical Reviews* **2020**, *120*, 7708-7744.
51. Florovskaya, V.N.; Zezin, R.D.; Ovchinnikova, L.I.; Pikovsky, Y.I.; Teplitskaya, T.A. *Diagnostics of organic matter in rocks and minerals of magmatic and geothermal origin* Nauka: Moscow, 1968.
52. Mukhin, L.M. Evolution of organic compounds in volcanic regions. *Nature* **1974**, *251*, 50-51.
53. Mukhin, L.M. Volcanic processes and synthesis of simple organic compounds on primitive earth. *Orig Life* **1976**, *7*, 355-368.
54. Markhinin, E.K.; Podkletnov, N.E. The phenomenon of formation of prebiological compounds in volcanic processes. *Orig Life* **1977**, *8*, 225-235, doi:10.1007/BF00930684.
55. Podkletnov, N.E.; Markhinin, E.K. New data on abiogenic synthesis of prebiological compounds in volcanic processes. *Orig Life* **1981**, *11*, 303-315, doi:10.1007/BF00931484.
56. Simoneit, B.R. Evidence for organic synthesis in high temperature aqueous media--facts and prognosis. *Orig Life Evol Biosph* **1995**, *25*, 119-140, doi:10.1007/BF01581578.
57. Sherwood Lollar, B.; Westgate, T.D.; Ward, J.A.; Slater, G.F.; Lacrampe-Couloume, G. Abiogenic formation of alkanes in the Earth's crust as a minor source for global hydrocarbon reservoirs. *Nature* **2002**, *416*, 522-524.
58. Sleep, N.H.; Meibom, A.; Fridriksson, T.; Coleman, R.G.; Bird, D.K. H_2-rich fluids from serpentinization: geochemical and biotic implications. *Proc Natl Acad Sci U S A* **2004**, *101*, 12818-12823, doi:10.1073/pnas.0405289101.
59. McCollom, T.M.; Seewald, J.S. Carbon isotope composition of organic compounds produced by abiotic synthesis under hydrothermal conditions. *Earth and Planetary Science Letters* **2006**, *243*, 74-84.
60. Simoneit, B.R.T.; Deamer, D.W.; Kompanichenko, V. Characterization of hydrothermally generated oil from the Uzon caldera, Kamchatka. *Applied Geochemistry* **2009**, *24*, 303-309.
61. Taran, Y.A.; Varley, N.R.; Inguaggiato, S.; Cienfuegos, E. Geochemistry of H_2 and CH_4-enriched hydrothermal fluids of Socorro Island, Revillagigedo Archipelago, Mexico. Evidence for serpentinization and abiogenic methane. *Geofluids* **2010**, *10*, 542-555.
62. Etiope, G.; Sherwood Lollar, B. Abiotic methane on Earth. *Reviews of Geophysics* **2013**, *51*, 276-299.
63. Klein, F.; Tarnas, J.D.; Bach, W. Abiotic sources of molecular hydrogen on Earth. *Elements: An International Magazine of Mineralogy, Geochemistry, and Petrology* **2020**, *16*, 19-24.
64. Truche, L.; Bourdelle, F.; Salvi, S.; Lefeuvre, N.; Zug, A.; Lloret, E. Hydrogen generation during hydrothermal alteration of peralkaline granite. *Geochimica et Cosmochimica Acta* **2021**, *308*, 42-59.
65. Garrels, R.M.; Christ, C.L. *Solutions, Minerals and Equilibria*; W. H. Freeman: San Francisco, 1965.

66. Okrusch, M.; Frimmel, H.M. *Mineralogy: An Introduction to Minerals, Rocks, and Mineral Deposits*; Springer: Heidelberg, 2020.
67. Moretti, R.; Neuville, D.R. Redox equilibria: from basic concepts to the magmatic realm. *Magma Redox Geochemistry* **2021**, 1-17.
68. Schoonen, M.A.A.; Xu, Y.; Bebie, J. Energetics and kinetics of the prebiotic synthesis of simple organic acids and amino acids with the FeS-H_2S/FeS_2 redox couple as reductant. *Orig. Life Evol. Biosph.* **1999**, *29*, 5-32.
69. Søndergaard, M. Redox Potetial. In *Encyclopedia of Inland Waters*, Likens, G.E., Ed.; 2009; pp. 852-859.
70. Cicconi, M.R.; Moretti, R.; Neuville, D.R. Earth's electrodes. *Elements: An International Magazine of Mineralogy, Geochemistry, and Petrology* **2020**, *16*, 157-160.
71. Dutton, P.L. Redox potentiometry: Determination of midpoint potentials of oxidation-reduction components of biological electron-transfer systems. In *Methods in enzymology*; Elsevier: 1978; Volume 54, pp. 411-435.
72. Migdisov, A.A.; Bychkov, A.Y. The behaviour of metals and sulphur during the formation of hydrothermal mercury-antimony-arsenic mineralization, Uzon caldera, Kamchatka, Russia. *Journal of Volcanology and Geothermal Research* **1998**, *84*, 153-171.
73. Lopez-Mirabal, H.R.; Winther, J.R. Redox characteristics of the eukaryotic cytosol. *Biochim Biophys Acta* **2008**, *1783*, 629-640, doi:10.1016/j.bbamcr.2007.10.013.
74. Wald, G. The Origins of Life. *Proc Natl Acad Sci U S A* **1964**, *52*, 595-611.
75. Xu, Y.; Schoonen, M.A.A. The absolute energy positions of conduction and valence bands of selected semiconducting minerals. *American Mineralogist* **2000**, *85*, 543-556.
76. Schoonen, M.; Smirnov, A.; Cohn, C. A perspective on the role of minerals in prebiotic synthesis. *Ambio* **2004**, *33*, 539-551.
77. Senanayake, S.D.; Idriss, H. Photocatalysis and the origin of life: synthesis of nucleoside bases from formamide on TiO2(001) single surfaces. *Proc Natl Acad Sci U S A* **2006**, *103*, 1194-1198, doi:10.1073/pnas.0505768103.
78. Mulkidjanian, A.Y. On the origin of life in the zinc world: 1. Photosynthesizing, porous edifices built of hydrothermally precipitated zinc sulfide as cradles of life on Earth. *Biol Direct* **2009**, *4*, 26, doi:10.1186/1745-6150-4-26.
79. Mulkidjanian, A.Y.; Bychkov, A.Y.; Dibrova, D.V.; Galperin, M.Y.; Koonin, E.V. Open questions on the origin of life at anoxic geothermal fields. *Orig Life Evol Biosph* **2012**, *42*, 507-516, doi:10.1007/s11084-012-9315-0.
80. Zhou, R.; Guzman, M.I. Photocatalytic reduction of fumarate to succinate on ZnS mineral surfaces. *The Journal of Physical Chemistry C* **2016**, *120*, 7349-7357.
81. Garrison, W.M.; Morrison, D.C.; Hamilton, J.G.; Benson, A.A.; Calvin, M. Reduction of carbon dioxide in aqueous solutions by ionizing radiation. *Science* **1951**, *114*, 416-418.
82. Getoff, N.; Scholes, G.; Weiss, J. Reduction of Carbon Dioxide in Aqueous Solutions under the Influence of Radiation. *Tetrahedron Letters* **1960**, 17-23.
83. Wu, S.; Shen, L.; Lin, Y.; Yin, K.; Yang, C. Sulfite-based advanced oxidation and reduction processes for water treatment. *Chemical Engineering Journal* **2021**, *414*, 128872.
84. Neupane, P.; Bartels, D.M.; Thompson, W.H. Exploring the Unusual Reactivity of the Hydrated Electron with CO(2). *J Phys Chem B* **2024**, *128*, 567-575, doi:10.1021/acs.jpcb.3c06935.
85. Getoff, N. Significance of solvated electrons (eaq−) as promoters of life on Earth. *in vivo* **2014**, *28*, 61-66.
86. Saladino, R.; Carota, E.; Botta, G.; Kapralov, M.; Timoshenko, G.N.; Rozanov, A.Y.; Krasavin, E.; Di Mauro, E. Meteorite-catalyzed syntheses of nucleosides and of other prebiotic compounds from formamide under proton irradiation. *Proc Natl Acad Sci U S A* **2015**, *112*, E2746-2755, doi:10.1073/pnas.1422225112.
87. Bizzarri, B.M.; Fanelli, A.; Kapralov, M.; Krasavin, E.; Saladino, R. Meteorite-catalyzed intermolecular trans-glycosylation produces nucleosides under proton beam irradiation. *RSC Adv* **2021**, *11*, 19258-19264, doi:10.1039/d1ra02379a.
88. Adam, Z. Actinides and life's origins. *Astrobiology* **2007**, *7*, 852-872.
89. Maruyama, S.; Kurokawa, K.; Ebisuzaki, T.; Sawaki, Y.; Suda, K.; Santosh, M. Nine requirements for the origin of Earth's life: Not at the hydrothermal vent, but in a nuclear geyser system. *Geoscience Frontiers* **2019**, *10*, 1337-1357.
90. Ershov, B. Natural Radioactivity and Chemical Evolution on the Early Earth: Prebiotic Chemistry and Oxygenation. *Molecules* **2022**, *27*, doi:10.3390/molecules27238584.

91. Vladilo, G. On the Role of (40)K in the Origin of Terrestrial Life. *Life (Basel)* **2022**, *12*, doi:10.3390/life12101620.
92. Moore, B.; Webster, T.A. Synthesis by sunlight in relationship to the origin of life. Synthesis of formaldehyde from carbon dioxide and water by inorganic colloids acting as transformers of light energy *Proc. R. Soc. Lond. B Biol. Sci.* **1913**, *87*, 163-176.
93. Miller, S.L. A production of amino acids under possible primitive Earth conditions. *Science* **1953**, *117*, 528-529.
94. Sagan, C.; Miller, S.L. Molecular Synthesis in Simulated Reducing Planetary Atmospheres. *Astronomical Journal, Vol. 65, p. 499* **1960**, *65*, 499.
95. Ponnamperuma, C.; Lemmon, R.M.; Mariner, R.; Calvin, M. Formation of Adenine by Electron Irradiation of Methane, Ammonia, and Water. *Proc Natl Acad Sci U S A* **1963**, *49*, 737-740.
96. Ponnamperuma, C.; Mariner, R.; Sagan, C. Formation of adenosine by ultra-violet irradiation of a solution of adenine and ribose. *Nature* **1963**, *198*, 1199-1200.
97. Ponnamperuma, C.; Sagan, C.; Mariner, R. Synthesis of adenosine triphosphate under possible primitive Earth conditions. *Nature* **1963**, *199*, 222-226.
98. Calvin, M. *Chemical Evolution: Molecular Evolution Towards the Origin of Living Systems on the Earth and Elsewhere*; Oxford University Press: Oxford, 1969.
99. Mason, S.V. *Chemical Evolution*; Clarendon Press: Oxford, 1992.
100. Cleaves, H.J.; Chalmers, J.H.; Lazcano, A.; Miller, S.L.; Bada, J.L. A reassessment of prebiotic organic synthesis in neutral planetary atmospheres. *Orig Life Evol Biosph* **2008**, *38*, 105-115, doi:10.1007/s11084-007-9120-3.
101. Parker, E.T.; Cleaves, H.J.; Dworkin, J.P.; Glavin, D.P.; Callahan, M.; Aubrey, A.; Lazcano, A.; Bada, J.L. Primordial synthesis of amines and amino acids in a 1958 Miller H2S-rich spark discharge experiment. *Proc Natl Acad Sci U S A* **2011**, doi:10.1073/pnas.1019191108.
102. Saladino, R.; Crestini, C.; Pino, S.; Costanzo, G.; Di Mauro, E. Formamide and the origin of life. *Phys Life Rev* **2012**, *9*, 84-104, doi:10.1016/j.plrev.2011.12.002.
103. Benner, S.A.; Kim, H.J.; Carrigan, M.A. Asphalt, water, and the prebiotic synthesis of ribose, ribonucleosides, and RNA. *Acc Chem Res* **2012**, *45*, 2025-2034, doi:10.1021/ar200332w.
104. Oparin, A.I. *The Origin of Life*; Moskowskiy rabochiy: Moscow, 1924.
105. Oparin, A.I. *The Origin of Life*; Macmillan: New York, 1938.
106. Butlerov, A.M. Formation synthétique d'une substance sucreé. *C.R. Acad. Sci.* **1861**, *53*, 145-147.
107. Delidovich, I.V.; Simonov, A.N.; Taran, O.P.; Parmon, V.N. Catalytic formation of monosaccharides: From the formose reaction towards selective synthesis. *ChemSusChem* **2014**, *7*, 1833-1846.
108. Omran, A.; Menor-Salvan, C.; Springsteen, G.; Pasek, M. The messy alkaline formose reaction and its link to metabolism. *Life* **2020**, *10*, 125.
109. Pestunova, O.; Simonov, A.; Snytnikov, V.; Stoyanovsky, V.; Parmon, V. Putative mechanism of the sugar formation on prebiotic Earth initiated by UV-radiation. *Space Life Sciences: Astrobiology: Steps toward Origin of Life and Titan before Cassini* **2005**, *36*, 214-219.
110. Ricardo, A.; Carrigan, M.A.; Olcott, A.N.; Benner, S.A. Borate minerals stabilize ribose. *Science* **2004**, *303*, 196, doi:10.1126/science.1092464.
111. Benner, S.A.; Kim, H.J.; Kim, M.J.; Ricardo, A. Planetary organic chemistry and the origins of biomolecules. *Cold Spring Harb Perspect Biol* **2010**, *2*, a003467, doi:10.1101/cshperspect.a003467.
112. Ziegler, E.W.; Kim, H.J.; Benner, S.A. Molybdenum(VI)-Catalyzed Rearrangement of Prebiotic Carbohydrates in Formamide, a Candidate Prebiotic Solvent. *Astrobiology* **2018**, *18*, 1159-1170, doi:10.1089/ast.2017.1742.
113. Costanzo, G.; Pino, S.; Timperio, A.M.; Sponer, J.E.; Sponer, J.; Novakova, O.; Sedo, O.; Zdrahal, Z.; Di Mauro, E. Non-Enzymatic Oligomerization of 3', 5' Cyclic AMP. *PLoS One* **2016**, *11*, e0165723, doi:10.1371/journal.pone.0165723.
114. White, H.B., 3rd. Coenzymes as fossils of an earlier metabolic state. *J. Mol. Evol.* **1976**, *7*, 101-104.
115. Goldman, A.D.; Kacar, B. Cofactors are Remnants of Life's Origin and Early Evolution. *J Mol Evol* **2021**, *89*, 127-133, doi:10.1007/s00239-020-09988-4.
116. Calvin, M. *Chemical Evolution*; Clarendon Press: Oxford, 1969.
117. Burcar, B.; Pasek, M.; Gull, M.; Cafferty, B.J.; Velasco, F.; Hud, N.V.; Menor-Salvan, C. Darwin's Warm Little Pond: A One-Pot Reaction for Prebiotic Phosphorylation and the Mobilization of

Phosphate from Minerals in a Urea-Based Solvent. *Angew Chem Int Ed Engl* **2016**, *55*, 13249-13253, doi:10.1002/anie.201606239.
118. Bortnikova, S.B.; S.P., B.; Manstein, Y.A.; Kiryuhin, A.V.; Vernikovskaya, I.V.; Palchik, N.A. Thermal springs hydrogeochemistry and structure at Northmutnovskoe fumarole field (South Kamchatka, Russia). In *Proceedings, Thirty-Fourth Workshop on Geothermal Reservoir Engineering, Stanford University*, Gordeev, E.I., Ed.; Stanford, CA, 2009; pp. SGP-TR-187.
119. Pino, S.; Sponer, J.E.; Costanzo, G.; Saladino, R.; Mauro, E.D. From formamide to RNA, the path is tenuous but continuous. *Life (Basel)* **2015**, *5*, 372-384, doi:10.3390/life5010372.
120. Harada, K. Formation of Amino-Acids by Thermal Decomposition of Formamide - Oligomerization of Hydrogen Cyanide. *Nature* **1967**, *214*, 479-&.
121. Schoffstall, A.M.; Laing, E.M. Equilibration of nucleotide derivatives in formamide. *Origins of Life and Evolution of the Biosphere* **1984**, *14*, 221-228.
122. Schoffstall, A.M. Prebiotic phosphorylation of nucleosides in formamide. *Orig Life* **1976**, *7*, 399-412.
123. Schoffstall, A.M.; Barto, R.J.; Ramos, D.L. Nucleoside and deoxynucleoside phosphorylation in formamide solutions. *Orig Life* **1982**, *12*, 143-151.
124. Schoffstall, A.M.; Mahone, S.M. Formate ester formation in amide solutions. *Orig Life Evol Biosph* **1988**, *18*, 389-396.
125. Saladino, R.; Crestini, C.; Costanzo, G.; Negri, R.; Di Mauro, E. A possible prebiotic synthesis of purine, adenine, cytosine, and 4(3H)-pyrimidinone from formamide: implications for the origin of life. *Bioorg Med Chem* **2001**, *9*, 1249-1253, doi:S0968089600003400 [pii].
126. Saladino, R.; Crestini, C.; Ciciriello, F.; Costanzo, G.; Di Mauro, E. About a formamide-based origin of informational polymers: syntheses of nucleobases and favourable thermodynamic niches for early polymers. *Orig Life Evol Biosph* **2006**, *36*, 523-531, doi:10.1007/s11084-006-9053-2.
127. Costanzo, G.; Saladino, R.; Crestini, C.; Ciciriello, F.; Di Mauro, E. Formamide as the main building block in the origin of nucleic acids. *BMC Evol Biol* **2007**, *7 Suppl 2*, S1, doi:10.1186/1471-2148-7-S2-S1.
128. Saladino, R.; Crestini, C.; Ciciriello, F.; Costanzo, G.; Di Mauro, E. Formamide chemistry and the origin of informational polymers. *Chem Biodivers* **2007**, *4*, 694-720, doi:10.1002/cbdv.200790059.
129. Saladino, R.; Crestini, C.; Ciciriello, F.; Pino, S.; Costanzo, G.; Di Mauro, E. From formamide to RNA: the roles of formamide and water in the evolution of chemical information. *Res Microbiol* **2009**, *160*, 441-448, doi:10.1016/j.resmic.2009.06.001.
130. Barks, H.L.; Buckley, R.; Grieves, G.A.; Di Mauro, E.; Hud, N.V.; Orlando, T.M. Guanine, adenine, and hypoxanthine production in UV-irradiated formamide solutions: relaxation of the requirements for prebiotic purine nucleobase formation. *Chembiochem* **2010**, *11*, 1240-1243, doi:10.1002/cbic.201000074.
131. Saladino, R.; Botta, G.; Pino, S.; Costanzo, G.; Di Mauro, E. Genetics first or metabolism first? The formamide clue. *Chem Soc Rev* **2012**, doi:10.1039/c2cs35066a.
132. Sponer, J.E.; Sponer, J.; Novakova, O.; Brabec, V.; Sedo, O.; Zdrahal, Z.; Costanzo, G.; Pino, S.; Saladino, R.; Di Mauro, E. Emergence of the First Catalytic Oligonucleotides in a Formamide-Based Origin Scenario. *Chemistry* **2016**, *22*, 3572-3586, doi:10.1002/chem.201503906.
133. Saladino, R.; Di Mauro, E.; García-Ruiz, J.M. A universal geochemical scenario for formamide condensation and prebiotic chemistry. *Chemistry–A European Journal* **2019**, *25*, 3181-3189.
134. Šponer, J.E.; Šponer, J.; Výravský, J.; Šedo, O.; Zdráhal, Z.; Costanzo, G.; Di Mauro, E.; Wunnava, S.; Braun, D.; Matyášek, R. Nonenzymatic, Template-Free Polymerization of 3', 5'Cyclic Guanosine Monophosphate on Mineral Surfaces. *ChemSystemsChem* **2021**, *3*, e2100017.
135. Sponer, J.E.; Sponer, J.; Kovarik, A.; Sedo, O.; Zdrahal, Z.; Costanzo, G.; Di Mauro, E. Questions and Answers Related to the Prebiotic Production of Oligonucleotide Sequences from 3',5' Cyclic Nucleotide Precursors. *Life (Basel)* **2021**, *11*, doi:10.3390/life11080800.
136. Powner, M.W.; Gerland, B.; Sutherland, J.D. Synthesis of activated pyrimidine ribonucleotides in prebiotically plausible conditions. *Nature* **2009**, *459*, 239-242, doi:10.1038/nature08013.
137. Powner, M.W.; Sutherland, J.D. Prebiotic chemistry: a new modus operandi. *Philos Trans R Soc Lond B Biol Sci* **2011**, *366*, 2870-2877, doi:10.1098/rstb.2011.0134.

138. Patel, B.H.; Percivalle, C.; Ritson, D.J.; Duffy, C.D.; Sutherland, J.D. Common origins of RNA, protein and lipid precursors in a cyanosulfidic protometabolism. *Nat Chem* **2015**, *7*, 301-307, doi:10.1038/nchem.2202.
139. Sutherland, J.D. The Origin of Life--Out of the Blue. *Angew Chem Int Ed Engl* **2016**, *55*, 104-121, doi:10.1002/anie.201506585.
140. Liu, Z.; Wu, L.F.; Kufner, C.L.; Sasselov, D.D.; Fischer, W.W.; Sutherland, J.D. Prebiotic photoredox synthesis from carbon dioxide and sulfite. *Nat Chem* **2021**, *13*, 1126-1132, doi:10.1038/s41557-021-00789-w.
141. Green, N.J.; Xu, J.; Sutherland, J.D. Illuminating Life's Origins: UV Photochemistry in Abiotic Synthesis of Biomolecules. *J Am Chem Soc* **2021**, *143*, 7219-7236, doi:10.1021/jacs.1c01839.
142. Chyba, C.; Sagan, C. Endogenous production, exogenous delivery and impact-shock synthesis of organic molecules: an inventory for the origins of life. *Nature* **1992**, *355*, 125-132, doi:10.1038/355125a0.
143. Meierhenrich, U.J.; Muñoz Caro, G.M.; Schutte, W.A.; Thiemann, W.H.P.; Barbier, B.; Brack, A. Precursors of biological cofactors from ultraviolet irradiation of circumstellar/interstellar ice analogues. *Chemistry–A European Journal* **2005**, *11*, 4895-4900.
144. Airapetian, V.S.; Glocer, A.; Gronoff, G.; Hébrard, E.; Danchi, W. Prebiotic chemistry and atmospheric warming of early Earth by an active young Sun. *Nature Geoscience* **2016**, *9*, 452-455, doi:10.1038/ngeo2719.
145. Gomes, R.J.; Birch, C.; Cencer, M.M.; Li, C.; Son, S.-B.; Bloom, I.D.; Assary, R.S.; Amanchukwu, C.V. Probing electrolyte influence on CO2 reduction in aprotic solvents. *The Journal of Physical Chemistry C* **2022**, *126*, 13595-13606.
146. Iffland, L.; Siegmund, D.; Apfel, U.P. Electrochemical CO2 and Proton Reduction by a Co (dithiacyclam) Complex. *Zeitschrift für anorganische und allgemeine Chemie* **2020**, *646*, 746-753.
147. Hulshof, J.; Ponnamperuma, C. Prebiotic condensation reactions in an aqueous medium: a review of condensing agents. *Orig Life* **1976**, *7*, 197-124, doi:10.1007/BF00926938.
148. Cafferty, B.J.; Hud, N.V. Abiotic synthesis of RNA in water: a common goal of prebiotic chemistry and bottom-up synthetic biology. *Curr Opin Chem Biol* **2014**, *22*, 146-157, doi:10.1016/j.cbpa.2014.09.015.
149. Bernal, J.D. *The Origin of Life*; Weidenfeld and Nicolson: London, 1967.
150. Joyce, G.F. RNA evolution and the origins of life. *Nature* **1989**, *338*, 217-224, doi:10.1038/338217a0.
151. Zubay, G.; Mui, T. Prebiotic synthesis of nucleotides. *Orig Life Evol Biosph* **2001**, *31*, 87-102, doi:10.1023/a:1006722423070.
152. Benner, S.A.; Ricardo, A.; Carrigan, M.A. Is there a common chemical model for life in the universe? *Curr Opin Chem Biol* **2004**, *8*, 672-689, doi:10.1016/j.cbpa.2004.10.003.
153. Deamer, D.; Singaram, S.; Rajamani, S.; Kompanichenko, V.; Guggenheim, S. Self-assembly processes in the prebiotic environment. *Philos Trans R Soc Lond B Biol Sci* **2006**, *361*, 1809-1818, doi:10.1098/rstb.2006.1905.
154. Mulkidjanian, A.Y.; Bychkov, A.Y.; Dibrova, D.V.; Galperin, M.Y.; Koonin, E.V. Origin of first cells at terrestrial, anoxic geothermal fields. *Proc Natl Acad Sci U S A* **2012**, *109*, E821-830, doi:10.1073/pnas.1117774109.
155. Deamer, D.; Damer, B.; Kompanichenko, V. Hydrothermal Chemistry and the Origin of Cellular Life. *Astrobiology* **2019**, *19*, 1523-1537, doi:10.1089/ast.2018.1979.
156. Bada, J.L.; Bigham, C.; Miller, S.L. Impact melting of frozen oceans on the early Earth: implications for the origin of life. *Proc Natl Acad Sci U S A* **1994**, *91*, 1248-1250, doi:10.1073/pnas.91.4.1248.
157. Levy, M.; Miller, S.L. The stability of the RNA bases: implications for the origin of life. *Proc Natl Acad Sci U S A* **1998**, *95*, 7933-7938, doi:10.1073/pnas.95.14.7933.
158. Miyakawa, S.; Cleaves, H.J.; Miller, S.L. The cold origin of life: A. Implications based on the hydrolytic stabilities of hydrogen cyanide and formamide. *Orig Life Evol Biosph* **2002**, *32*, 195-208.
159. Monnard, P.A.; Kanavarioti, A.; Deamer, D.W. Eutectic phase polymerization of activated ribonucleotide mixtures yields quasi-equimolar incorporation of purine and pyrimidine nucleobases. *J Am Chem Soc* **2003**, *125*, 13734-13740, doi:10.1021/ja036465h.
160. Monnard, P.A.; Szostak, J.W. Metal-ion catalyzed polymerization in the eutectic phase in water-ice: a possible approach to template-directed RNA polymerization. *J Inorg Biochem* **2008**, *102*, 1104-1111, doi:10.1016/j.jinorgbio.2008.01.026.

161. Attwater, J.; Wochner, A.; Pinheiro, V.B.; Coulson, A.; Holliger, P. Ice as a protocellular medium for RNA replication. *Nat Commun* **2010**, *1*, 76, doi:10.1038/ncomms1076.
162. Attwater, J.; Wochner, A.; Holliger, P. In-ice evolution of RNA polymerase ribozyme activity. *Nat Chem* **2013**, *5*, 1011-1018, doi:10.1038/nchem.1781.
163. Mutschler, H.; Wochner, A.; Holliger, P. Freeze-thaw cycles as drivers of complex ribozyme assembly. *Nat Chem* **2015**, *7*, 502-508, doi:10.1038/nchem.2251.
164. Sasselov, D.D.; Grotzinger, J.P.; Sutherland, J.D. The origin of life as a planetary phenomenon. *Sci Adv* **2020**, *6*, eaax3419, doi:10.1126/sciadv.aax3419.
165. Belozersky, A.N. On the species specificity of the nucleic acids of bacteria. In *The Origin of Life on the Earth*, Oparin, A.I., Pasynskii, A.G., Braunshtein, A.E., Pavlovskaya, T.E., Clark, F., Synge, R.L.M., Eds.; Pergamon Publishers: London, 1959; pp. 322-331.
166. Rich, A. On the problems of evolution and biochemical information transfer. In *Horizons in Biochemistry*, Kasha, M., Pullman, B., Eds.; Academic Press: New York, 1962; pp. 103-126.
167. Woese, C.R. *The Genetic Code*; Harper and Row: New York, 1967.
168. Crick, F.H. The origin of the genetic code. *J. Mol. Biol.* **1968**, *38*, 367-379.
169. Orgel, L.E. Evolution of the genetic apparatus. *J. Mol. Biol.* **1968**, *38*, 381-393.
170. Barbieri, M. The ribotype theory on the origin of life. *J Theor Biol* **1981**, *91*, 545-601, doi:10.1016/0022-5193(81)90211-3.
171. Cech, T.R. RNA Splicing: Three Themes with Variations. *Cell* **1983**, *34*, 713-716.
172. Guerrier-Takada, C.; Gardiner, K.; Marsh, T.; Pace, N.; Altman, S. The RNA moiety of ribonuclease P is the catalytic subunit of the enzyme. *Cell* **1983**, *35*, 849-857.
173. Gilbert, W. The RNA world. *Nature* **1986**, *319*, 618.
174. Jeffares, D.C.; Poole, A.M.; Penny, D. Relics from the RNA world. *J Mol Evol* **1998**, *46*, 18-36, doi:10.1007/pl00006280.
175. Lincoln, T.A.; Joyce, G.F. Self-sustained replication of an RNA enzyme. *Science* **2009**, *323*, 1229-1232, doi:10.1126/science.1167856.
176. Vaidya, N.; Manapat, M.L.; Chen, I.A.; Xulvi-Brunet, R.; Hayden, E.J.; Lehman, N. Spontaneous network formation among cooperative RNA replicators. *Nature* **2012**, *491*, 72-77, doi:10.1038/nature11549.
177. Cech, T.R. The RNA worlds in context. *Cold Spring Harb Perspect Biol* **2012**, *4*, a006742, doi:10.1101/cshperspect.a006742.
178. Bernhardt, H.S. The RNA world hypothesis: the worst theory of the early evolution of life (except for all the others)(a). *Biol Direct* **2012**, *7*, 23, doi:10.1186/1745-6150-7-23.
179. Eigen, M. *From Strange Simplicity to Complex Familiarity: A Treatise on Matter, Information, Life and Thought* Oxford University Press: Oxford, 2013.
180. Neveu, M.; Kim, H.J.; Benner, S.A. The "strong" RNA world hypothesis: fifty years old. *Astrobiology* **2013**, *13*, 391-403, doi:10.1089/ast.2012.0868.
181. Bowman, J.C.; Hud, N.V.; Williams, L.D. The ribosome challenge to the RNA world. *J Mol Evol* **2015**, *80*, 143-161, doi:10.1007/s00239-015-9669-9.
182. Higgs, P.G.; Lehman, N. The RNA World: molecular cooperation at the origins of life. *Nat Rev Genet* **2015**, *16*, 7-17, doi:10.1038/nrg3841.
183. Lazcano, A. The RNA World: Piecing together the historical development of a hypothesis. *Metode Science Studies Journal* **2016**, 167-173.
184. Sutherland, J.D. Opinion: Studies on the origin of life—the end of the beginning. *Nature Reviews Chemistry* **2017**, *1*, 0012.
185. Wachowius, F.; Attwater, J.; Holliger, P. Nucleic acids: function and potential for abiogenesis. *Q Rev Biophys* **2017**, *50*, e4, doi:10.1017/S0033583517000038.
186. Benner, S.A.; Kim, H.-J.; Biondi, E. Mineral-organic interactions in prebiotic synthesis: The discontinuous synthesis model for the formation of RNA in naturally complex geological environments. In *Prebiotic Chemistry and Chemical Evolution of Nucleic Acids*, Menor-Salván, C., Ed.; Springer: 2018; pp. 31-83.
187. Joyce, G.F.; Szostak, J.W. Protocells and RNA self-replication. *Cold Spring Harbor Perspectives in Biology* **2018**, *10*, a034801.
188. Hud, N.V. Searching for lost nucleotides of the pre-RNA World with a self-refining model of early Earth. *Nat Commun* **2018**, *9*, 5171, doi:10.1038/s41467-018-07389-2.
189. Crisp, A.; Carell, T. Rethinking the tools of the RNA world. *Elife* **2018**, *7*, doi:10.7554/eLife.38297.

190. Mutschler, H.; Taylor, A.I.; Porebski, B.T.; Lightowlers, A.; Houlihan, G.; Abramov, M.; Herdewijn, P.; Holliger, P. Random-sequence genetic oligomer pools display an innate potential for ligation and recombination. *Elife* **2018**, *7*, doi:10.7554/eLife.43022.
191. Adamski, P.; Eleveld, M.; Sood, A.; Kun, A.; Szilagyi, A.; Czaran, T.; Szathmary, E.; Otto, S. From self-replication to replicator systems en route to de novo life. *Nat Rev Chem* **2020**, *4*, 386-403, doi:10.1038/s41570-020-0196-x.
192. Benner, S.A.; Bell, E.A.; Biondi, E.; Brasser, R.; Carell, T.; Kim, H.J.; Mojzsis, S.J.; Omran, A.; Pasek, M.A.; Trail, D. When did life likely emerge on Earth in an RNA-first process? *ChemSystemsChem* **2020**, *2*, e1900035, doi:10.1002/syst.201900035.
193. Zhou, L.; O'Flaherty, D.K.; Szostak, J.W. Assembly of a Ribozyme Ligase from Short Oligomers by Nonenzymatic Ligation. *J Am Chem Soc* **2020**, *142*, 15961-15965, doi:10.1021/jacs.0c06722.
194. Mizuuchi, R.; Ichihashi, N. Minimal RNA self-reproduction discovered from a random pool of oligomers. *Chem Sci* **2023**, *14*, 7656-7664, doi:10.1039/d3sc01940c.
195. Fine, J.L.; Pearlman, R.E. On the origin of life: an RNA-focused synthesis and narrative. *RNA* **2023**, *29*, 1085-1098, doi:10.1261/rna.079598.123.
196. Papastavrou, N.; Horning, D.P.; Joyce, G.F. RNA-catalyzed evolution of catalytic RNA. *Proc Natl Acad Sci U S A* **2024**, *121*, e2321592121, doi:10.1073/pnas.2321592121.
197. Vlassov, A.V.; Kazakov, S.A.; Johnston, B.H.; Landweber, L.F. The RNA world on ice: a new scenario for the emergence of RNA information. *J Mol Evol* **2005**, *61*, 264-273, doi:10.1007/s00239-004-0362-7.
198. Akoopie, A.; Muller, U.F. Lower temperature optimum of a smaller, fragmented triphosphorylation ribozyme. *Phys Chem Chem Phys* **2016**, *18*, 20118-20125, doi:10.1039/c6cp00672h.
199. Smail, B.A.; Clifton, B.E.; Mizuuchi, R.; Lehman, N. Spontaneous advent of genetic diversity in RNA populations through multiple recombination mechanisms. *RNA* **2019**, *25*, 453-464, doi:10.1261/rna.068908.118.
200. Belozersky, A.N.; Spirin, A.S. A correlation between the compositions of deoxyribonucleic and ribonucleic acids. *Nature* **1958**, *182*, 111-112.
201. Chetverina, H.V.; Demidenko, A.A.; Ugarov, V.I.; Chetverin, A.B. Spontaneous rearrangements in RNA sequences. *FEBS Lett.* **1999**, *450*, 89-94.
202. Harris, A.J.; Goldman, A.D. The very early evolution of protein translocation across membranes. *PLoS Comput Biol* **2021**, *17*, e1008623, doi:10.1371/journal.pcbi.1008623.
203. Hury, J.; Nagaswamy, U.; Larios-Sanz, M.; Fox, G.E. Ribosome origins: the relative age of 23S rRNA Domains. *Orig Life Evol Biosph* **2006**, *36*, 421-429, doi:10.1007/s11084-006-9011-z.
204. Bokov, K.; Steinberg, S.V. A hierarchical model for evolution of 23S ribosomal RNA. *Nature* **2009**, *457*, 977-980.
205. Davidovich, C.; Belousoff, M.; Bashan, A.; Yonath, A. The evolving ribosome: from non-coded peptide bond formation to sophisticated translation machinery. *Res Microbiol* **2009**, *160*, 487-492.
206. Fox, G.E. Origin and evolution of the ribosome. *Cold Spring Harb Perspect Biol* **2010**, *2*, a003483, doi:10.1101/cshperspect.a003483.
207. Krupkin, M.; Matzov, D.; Tang, H.; Metz, M.; Kalaora, R.; Belousoff, M.J.; Zimmerman, E.; Bashan, A.; Yonath, A. A vestige of a prebiotic bonding machine is functioning within the contemporary ribosome. *Philos Trans R Soc Lond B Biol Sci* **2011**, *366*, 2972-2978.
208. Fox, G.E.; Tran, Q.; Yonath, A. An exit cavity was crucial to the polymerase activity of the early ribosome. *Astrobiology* **2012**, *12*, 57-60, doi:10.1089/ast.2011.0692.
209. Noller, H.F. Evolution of protein synthesis from an RNA world. *Cold Spring Harb Perspect Biol* **2012**, *4*, a003681, doi:10.1101/cshperspect.a003681.
210. Petrov, A.S.; Bernier, C.R.; Hsiao, C.; Norris, A.M.; Kovacs, N.A.; Waterbury, C.C.; Stepanov, V.G.; Harvey, S.C.; Fox, G.E.; Wartell, R.M.; et al. Evolution of the ribosome at atomic resolution. *Proc Natl Acad Sci U S A* **2014**, *111*, 10251-10256, doi:10.1073/pnas.1407205111.
211. Petrov, A.S.; Gulen, B.; Norris, A.M.; Kovacs, N.A.; Bernier, C.R.; Lanier, K.A.; Fox, G.E.; Harvey, S.C.; Wartell, R.M.; Hud, N.V.; et al. History of the ribosome and the origin of translation. *Proc Natl Acad Sci U S A* **2015**, *112*, 15396-15401, doi:10.1073/pnas.1509761112.
212. Bose, T.; Fridkin, G.; Davidovich, C.; Krupkin, M.; Dinger, N.; Falkovich, A.H.; Peleg, Y.; Agmon, I.; Bashan, A.; Yonath, A. Origin of life: protoribosome forms peptide bonds and links RNA and protein dominated worlds. *Nucleic Acids Res* **2022**, *50*, 1815-1828, doi:10.1093/nar/gkac052.

213. Rivas, M.; Fox, G.E. How to build a protoribosome: structural insights from the first protoribosome constructs that have proven to be catalytically active. *RNA* **2023**, *29*, 263-272.
214. Eigen, M. Selforganization of matter and the evolution of biological macromolecules. *Naturwissenschaften.* **1971**, *58*, 465-523.
215. Eigen, M. Error catastrophe and antiviral strategy. *Proc Natl Acad Sci U S A* **2002**, *99*, 13374-13376, doi:10.1073/pnas.212514799.
216. Eigen, M.; Schuster, P. The hypercycle. A principle of natural self-organization. Part A: Emergence of the hypercycle. *Naturwissenschaften* **1977**, *64*, 541-565.
217. Eigen, M.; Schuster, P. The hypercycle. A principle of natural self-organization. Part C: the realistic hypercycle. . *Z. Naturwissenschaften* **1978**, *65*, 341-369.
218. Eigen, M.; Gardiner, W.C., Jr.; Schuster, P. Hypercycles and compartments. Compartments assists--but do not replace--hypercyclic organization of early genetic information. *J Theor Biol* **1980**, *85*, 407-411, doi:0022-5193(80)90315-X [pii].
219. Takeuchi, N.; Hogeweg, P. Evolutionary dynamics of RNA-like replicator systems: A bioinformatic approach to the origin of life. *Phys Life Rev* **2012**, doi:S1571-0645(12)00048-6.
220. Monnard, P.A.; Deamer, D.W. Membrane self-assembly processes: steps toward the first cellular life. *Anat Rec* **2002**, *268*, 196-207, doi:10.1002/ar.10154.
221. Hanczyc, M.M.; Fujikawa, S.M.; Szostak, J.W. Experimental models of primitive cellular compartments: Encapsulation, growth, and division. *Science* **2003**, *302*, 618-622.
222. Chen, I.A.; Salehi-Ashtiani, K.; Szostak, J.W. RNA catalysis in model protocell vesicles. *J Am Chem Soc* **2005**, *127*, 13213-13219, doi:10.1021/ja051784p.
223. Ricardo, A.; Szostak, J.W. Origin of life on earth. *Sci Am* **2009**, *301*, 54-61.
224. Mansy, S.S. Membrane transport in primitive cells. *Cold Spring Harb Perspect Biol* **2010**, *2*, a002188, doi:10.1101/cshperspect.a002188.
225. Mansy, S.S.; Schrum, J.P.; Krishnamurthy, M.; Tobe, S.; Treco, D.A.; Szostak, J.W. Template-directed synthesis of a genetic polymer in a model protocell. *Nature* **2008**, *454*, 122-125, doi:10.1038/nature07018.
226. Mansy, S.S.; Szostak, J.W. Reconstructing the emergence of cellular life through the synthesis of model protocells. *Cold Spring Harb Symp Quant Biol* **2009**, *74*, 47-54, doi:10.1101/sqb.2009.74.014.
227. Blain, J.C.; Szostak, J.W. Progress toward synthetic cells. *Annual review of biochemistry* **2014**, *83*, 615-640.
228. Engelhart, A.E.; Adamala, K.P.; Szostak, J.W. A simple physical mechanism enables homeostasis in primitive cells. *Nature chemistry* **2016**, *8*, 448-453.
229. Monnard, P.-A.; Deamer, D. RNA Protocells. In *Frontiers in Developmental Biology: Advances in Molecular Biology and Medicine*, Meyers, R.A., Ed.; Wiley-VCH: Weinheim, Germany, 2019; pp. 213-225.
230. Chetverina, H.V.; Chetverin, A.B. Cloning of RNA molecules *in vitro*. *Nucleic Acids Res.* **1993**, *21*, 2349-2353.
231. Chetverin, A.B.; Chetverina, E.V. Scientific and practical applications of molecular colonies. *Mol Biol (Mosk)* **2007**, *41*, 284-296.
232. Ashe, K. Studies towards the prebiotic synthesis of nucleotides and amino acids. UCL, London, 2018.
233. Fiore, M.; Strazewski, P. Bringing Prebiotic Nucleosides and Nucleotides Down to Earth. *Angew Chem Int Ed Engl* **2016**, *55*, 13930-13933, doi:10.1002/anie.201606232.
234. Whitaker, D.; Powner, M.W. Prebiotic nucleic acids need space to grow. *Nat Commun* **2018**, *9*, 5172, doi:10.1038/s41467-018-07221-x.
235. Becker, S.; Feldmann, J.; Wiedemann, S.; Okamura, H.; Schneider, C.; Iwan, K.; Crisp, A.; Rossa, M.; Amatov, T.; Carell, T. Unified prebiotically plausible synthesis of pyrimidine and purine RNA ribonucleotides. *Science* **2019**, *366*, 76-82, doi:10.1126/science.aax2747.
236. Sanchez, R.A.; Orgel, L.E. Studies in prebiotic synthesis. V. Synthesis and photoanomerization of pyrimidine nucleosides. *J Mol Biol* **1970**, *47*, 531-543, doi:10.1016/0022-2836(70)90320-7.
237. Rudolph, S.A.; Johnson, E.M.; Greengard, P. The enthalpy of hydrolysis of various 3',5'-and 2',3'-cyclic nucleotides. *J Biol Chem* **1971**, *246*, 1271-1273.
238. Markham, R.; Smith, J.D. The structure of ribonucleic acid. 3. The end groups, the general structure and the nature of thecore'. *Biochemical Journal* **1952**, *52*, 565.
239. Nichols, N.M.; Yue, D. Ribonucleases. *Curr Protoc Mol Biol* **2008**, *Chapter 3*, Unit3 13, doi:10.1002/0471142727.mb0313s84.

240. Zhenodarova, S.M. Stepwise Enzymatic Synthesis of the Specific C(3') - C(5') Internucleotide Bond. *Russian Chemical Journal* **1970**, *39*, 695-703.
241. Dirscherl, C.F.; Ianeselli, A.; Tetiker, D.; Matreux, T.; Queener, R.M.; Mast, C.B.; Braun, D. A heated rock crack captures and polymerizes primordial DNA and RNA. *Phys Chem Chem Phys* **2023**, *25*, 3375-3386, doi:10.1039/d2cp04538a.
242. Dass, A.V.; Wunnava, S.; Langlais, J.; von der Esch, B.; Krusche, M.; Ufer, L.; Chrisam, N.; Dubini, R.C.A.; Gartner, F.; Angerpointner, S.; et al. RNA Oligomerisation without Added Catalyst from 2′, 3′-Cyclic Nucleotides by Drying at Air-Water Interfaces. *ChemSystemsChem* **2023**, *5*, e202200026.
243. Cadet, J.; Vigny, P. The photochemistry of nucleic acids In *Bioorganic Photochemistry: Photochemistry and the Nucleic Acids* Morrison, H., Ed.; John Wiley & Sons: New York, 1990; pp. 1-273.
244. Sobolewski, A.L.; Domcke, W. The chemical physics of the photostability of life. *Europhysics News* **2006**, *37*, 20-23.
245. Beckstead, A.A.; Zhang, Y.; de Vries, M.S.; Kohler, B. Life in the light: nucleic acid photoproperties as a legacy of chemical evolution. *Physical Chemistry Chemical Physics* **2016**, *18*, 24228-24238.
246. Serrano-Andres, L.; Merchan, M. Are the five natural DNA/RNA base monomers a good choice from natural selection? A photochemical perspective. *Journal of Photochemistry and Photobiology C-Photochemistry Reviews* **2009**, *10*, 21-32.
247. Kim, S.C.; O'Flaherty, D.K.; Zhou, L.; Lelyveld, V.S.; Szostak, J.W. Inosine, but none of the 8-oxo-purines, is a plausible component of a primordial version of RNA. *Proc Natl Acad Sci U S A* **2018**, *115*, 13318-13323, doi:10.1073/pnas.1814367115.
248. Jericevic, Z.; Kucan, I.; Chambers, R.W. Photochemical cleavage of phosphodiester bonds in oligoribonucleotides. *Biochemistry* **1982**, *21*, 6563-6567, doi:10.1021/bi00268a037.
249. Halmann, M.; Platzner, I. The photochemistry of phosphorus compounds. Part II. Far-ultraviolet absorption spectra of some phosphorus oxyanions in aqueous solution. *Journal of the Chemical Society (Resumed)* **1965**, 1440-1449.
250. Fetzer, S.M.; Lebreton, P.R.; Rohmer, M.M.; Veillard, A. Valence ionization potentials of anionic phosphate esters: An ab initio quantum mechanical study. *International journal of quantum chemistry* **1997**, *65*, 1095-1106.
251. Ma, J.; Denisov, S.A.; Marignier, J.L.; Pernot, P.; Adhikary, A.; Seki, S.; Mostafavi, M. Ultrafast Electron Attachment and Hole Transfer Following Ionizing Radiation of Aqueous Uridine Monophosphate. *J Phys Chem Lett* **2018**, *9*, 5105-5109, doi:10.1021/acs.jpclett.8b02170.
252. Goossen, J.T.H.; Kloosterboer, J.G. Photolysis and hydrolysis of adenosine 5'-phosphates. *Photochem. Photobiol.* **1978**, *27*, 703-708.
253. Lindahl, T. The Intrinsic Fragility of DNA (Nobel Lecture). *Angew Chem Int Ed Engl* **2016**, *55*, 8528-8534, doi:10.1002/anie.201602159.
254. Sagan, C. Ultraviolet selection pressure on the earliest organisms. *J Theor Biol* **1973**, *39*, 195-200, doi:10.1016/0022-5193(73)90216-6.
255. Sagan, C. Radiation and the origin of the gene. *Evolution* **1957**, 40-55.
256. Mulkidjanian, A.Y.; Cherepanov, D.A.; Galperin, M.Y. Survival of the fittest before the beginning of life: selection of the first oligonucleotide-like polymers by UV light. *BMC Evol Biol* **2003**, *3*, 12, doi:10.1186/1471-2148-3-12.
257. Koonin, E.V.; Chumakov, K.M.; Agol, V.I. A comparative study on the UV resistance of double-stranded and single-stranded encephalomyocarditis virus RNAs - evaluation of the possible contribution of host-mediated repair. *J. Gen. Virol.* **1980**, *49*, 437-441.
258. Landauer, R. Irreversibility and heat generation in the computing process. *IBM Journal of Research and Development* **1961**, *5*, 183-191.
259. Williams, R.J.P.; Frausto da Silva, J.J.R. *The Biological Chemistry of the Elements*; Clarendon Press: Oxford, 1991.
260. Williams, R.J.; Frausto da Silva, J.J. The involvement of molybdenum in life. *Biochem Biophys Res Commun* **2002**, *292*, 293-299, doi:10.1006/bbrc.2002.6518.
261. Williams, R.J.P.; Frausto da Silva, J.J.R. *The Chemistry of Evolution: The Development of our Ecosystem* Elsevier: Amsterdam, 2006.
262. Anbar, A.D. Oceans. Elements and evolution. *Science* **2008**, *322*, 1481-1483.
263. Williams, R.J. Zinc in evolution. *J Inorg Biochem* **2012**, *111*, 104-109, doi:10.1016/j.jinorgbio.2012.01.004.

264. Smith, D.G.; Pal, R.; Parker, D. Measuring equilibrium bicarbonate concentrations directly in cellular mitochondria and in human serum using europium/terbium emission intensity ratios. *Chemistry–A European Journal* **2012**, *18*, 11604-11613.
265. Maret, W. Analyzing free zinc(II) ion concentrations in cell biology with fluorescent chelating molecules. *Metallomics* **2015**, *7*, 202-211, doi:10.1039/c4mt00230j.
266. Komarova, T.; McKeating, D.; Perkins, A.V.; Tinggi, U. Trace Element Analysis in Whole Blood and Plasma for Reference Levels in a Selected Queensland Population, Australia. *Int J Environ Res Public Health* **2021**, *18*, doi:10.3390/ijerph18052652.
267. Aulakh, S.K.; Varma, S.J.; Ralser, M. Metal ion availability and homeostasis as drivers of metabolic evolution and enzyme function. *Curr Opin Genet Dev* **2022**, *77*, 101987, doi:10.1016/j.gde.2022.101987.
268. Nies, D.H.; Silver, S., (Eds.) *Molecular Microbiology of Heavy Metals*. Springer-Verlag: Berlin, 2007.
269. Szostak, J.W.; Bartel, D.P.; Luisi, P.L. Synthesizing life. *Nature* **2001**, *409*, 387-390, doi:10.1038/35053176.
270. Szathmáry, E. Coevolution of metabolic networks and membranes: the scenario of progressive sequestration. *Philos. Trans. R. Soc. Lond. B Biol. Sci.* **2007**, *362*, 1781-1787.
271. Deamer, D.W. Origins of life: How leaky were primitive cells? *Nature* **2008**, *454*, 37-38.
272. Mulkidjanian, A.Y.; Galperin, M.Y.; Koonin, E.V. Co-evolution of primordial membranes and membrane proteins. *Trends Biochem Sci* **2009**, *34*, 206-215, doi:10.1016/j.tibs.2009.01.005.
273. Mulkidjanian, A.Y.; Galperin, M.Y. Evolutionary origins of membrane proteins In *Structural Bioinformatics of Membrane Proteins*, Frishman, D., Ed.; Spriger: Viena, 2010; pp. 1-28.
274. Koonin, E.V.; Mulkidjanian, A.Y. Evolution of cell division: from shear mechanics to complex molecular machineries. *Cell* **2013**, *152*, 942-944, doi:10.1016/j.cell.2013.02.008.
275. Macallum, A.B. The paleochemistry of the body fluids and tissues. *Physiol. Rev.* **1926**, *6*, 316-357.
276. Mulkidjanian, A.Y.; Galperin, M.Y. Physico-chemical and evolutionary constraints for the formation and selection of first biopolymers: towards the consensus paradigm of the abiogenic origin of life. *Chem Biodivers* **2007**, *4*, 2003-2015, doi:10.1002/cbdv.200790167.
277. Natochin, Y.V. The physiological evolution of animals: Sodium is the clue to resolving contradictions. *Herald of the Russian Academy of Sciences* **2007**, *77*, 581-591.
278. Mulkidjanian, A.Y.; Galperin, M.Y. On the abundance of zinc in the evolutionarily old protein domains. *Proc Natl Acad Sci U S A* **2010**, *107*, E137, doi:10.1073/pnas.1008745107.
279. Macallum, A.B. *The palaeochemistry of the ocean in relation to animal and vegetable protoplasm*; University Library; published by the Librarian: 1904.
280. Forterre, P.; Philippe, H. The last universal common ancestor (LUCA), simple or complex? *Biol Bull* **1999**, *196*, 373-375; discussion 375-377, doi:10.2307/1542973.
281. Mushegian, A. Gene content of LUCA, the last universal common ancestor. *Front Biosci* **2008**, *13*, 4657-4666, doi:10.2741/3031.
282. Goldman, A.D.; Becerra, A. A New View of the Last Universal Common Ancestor. *J Mol Evol* **2024**, *92*, 659-661, doi:10.1007/s00239-024-10193-w.
283. Delaye, L. The Unfinished Reconstructed Nature of the Last Universal Common Ancestor. *J Mol Evol* **2024**, *92*, 584-592, doi:10.1007/s00239-024-10187-8.
284. Goldman, A.D.; Bernhard, T.M.; Dolzhenko, E.; Landweber, L.F. LUCApedia: a database for the study of ancient life. *Nucleic Acids Res* **2013**, *41*, D1079-1082, doi:10.1093/nar/gks1217.
285. Crapitto, A.J.; Campbell, A.; Harris, A.J.; Goldman, A.D. A consensus view of the proteome of the last universal common ancestor. *Ecol Evol* **2022**, *12*, e8930, doi:10.1002/ece3.8930.
286. Hutchison, C.A., 3rd; Chuang, R.Y.; Noskov, V.N.; Assad-Garcia, N.; Deerinck, T.J.; Ellisman, M.H.; Gill, J.; Kannan, K.; Karas, B.J.; Ma, L.; et al. Design and synthesis of a minimal bacterial genome. *Science* **2016**, *351*, aad6253, doi:10.1126/science.aad6253.
287. Pelletier, J.F.; Sun, L.; Wise, K.S.; Assad-Garcia, N.; Karas, B.J.; Deerinck, T.J.; Ellisman, M.H.; Mershin, A.; Gershenfeld, N.; Chuang, R.Y.; et al. Genetic requirements for cell division in a genomically minimal cell. *Cell* **2021**, *184*, 2430-2440 e2416, doi:10.1016/j.cell.2021.03.008.
288. Galperin, M.Y.; Vera Alvarez, R.; Karamycheva, S.; Makarova, K.S.; Wolf, Y.I.; Landsman, D.; Koonin, E.V. COG database update 2024. *Nucleic Acids Res* **2024**, doi:10.1093/nar/gkae983.
289. Chang, A.; Jeske, L.; Ulbrich, S.; Hofmann, J.; Koblitz, J.; Schomburg, I.; Neumann-Schaal, M.; Jahn, D.; Schomburg, D. BRENDA, the ELIXIR core data resource in 2021: new developments and updates. *Nucleic Acids Res* **2021**, *49*, D498-D508, doi:10.1093/nar/gkaa1025.

290. Dibrova, D.V.; Konovalov, K.A.; Perekhvatov, V.V.; Skulachev, K.V.; Mulkidjanian, A.Y. COGcollator: a web server for analysis of distant relationships between homologous protein families. *Biol Direct* **2017**, *12*, 29, doi:10.1186/s13062-017-0198-x.
291. Prask, J.A.; Plocke, D.J. A Role for Zinc in the Structural Integrity of the Cytoplasmic Ribosomes of Euglena gacilis. *Plant Physiol* **1971**, *48*, 150-155, doi:10.1104/pp.48.2.150.
292. Akanuma, G. Diverse relationships between metal ions and the ribosome. *Biosci Biotechnol Biochem* **2021**, *85*, 1582-1593, doi:10.1093/bbb/zbab070.
293. Bayley, S.T.; Kushner, D.J. The ribosomes of the extremely halophilic bacterium, *Halobacterium cutirubrum*. *J Mol Biol* **1964**, *9*, 654-669.
294. Spirin, A.S.; Baranov, V.I.; Ryabova, L.A.; Ovodov, S.Y.; Alakhov, Y.B. A continuous cell-free translation system capable of producing polypeptides in high yield. *Science* **1988**, *242*, 1162-1164.
295. Conway, T.W. On the role of ammonium or potassium ion in amino acid polymerization. *Proc Natl Acad Sci U S A* **1964**, *51*, 1216-1220.
296. Yonath, A. The search and its outcome: high-resolution structures of ribosomal particles from mesophilic, thermophilic, and halophilic bacteria at various functional states. *Annu Rev Biophys Biomol Struct* **2002**, *31*, 257-273.
297. Klein, D.J.; Moore, P.B.; Steitz, T.A. The contribution of metal ions to the structural stability of the large ribosomal subunit. *RNA* **2004**, *10*, 1366-1379, doi:10.1261/rna.7390804.
298. Rozov, A.; Khusainov, I.; El Omari, K.; Duman, R.; Mykhaylyk, V.; Yusupov, M.; Westhof, E.; Wagner, A.; Yusupova, G. Importance of potassium ions for ribosome structure and function revealed by long-wavelength X-ray diffraction. *Nat Commun* **2019**, *10*, 2519, doi:10.1038/s41467-019-10409-4.
299. Shalaeva, D.N.; Cherepanov, D.A.; Galperin, M.Y.; Golovin, A.V.; Mulkidjanian, A.Y. Evolution of cation binding in the active sites of P-loop nucleoside triphosphatases in relation to the basic catalytic mechanism. *Elife* **2018**, *7*, doi:10.7554/eLife.37373.
300. Näslund, P.H.; Hultin, T. Effects of potassium deficiency on mammalian ribosomes. *Biochimica et Biophysica Acta (BBA)-Nucleic Acids and Protein Synthesis* **1970**, *204*, 237-247.
301. Oren, A. Life at high salt concentrations, intracellular KCl concentrations, and acidic proteomes. *Front Microbiol* **2013**, *4*, 315, doi:10.3389/fmicb.2013.00315.
302. Skulachev, V.P. *Membrane Bioenergetics*; Springer-Verlag: Berlin, 1988.
303. Drever, J.I. *The Geochemistry of Natural Waters: Surface and Groundwater Environments, 3rd Ed.*; Prentice Hall: NJ, 1997.
304. Maruyama, S.; Ikoma, M.; Genda, H.; Hirose, K.; Yokoyama, T.; Santosh, M. The naked planet Earth: Most essential pre-requisite for the origin and evolution of life. *Geoscience Frontiers* **2013**, *4*, 141-165.
305. Edwards, G. Sodium and potassium in meteorites. *Geochimica et Cosmochimica Acta* **1955**, *8*, 285-294.
306. Che, S.; Zega, T.J. Hydrothermal fluid activity on asteroid Itokawa. *Nature Astronomy* **2023**, 1-7.
307. Averiev, V.V. Conditions for the discharge of the Pauzhetka high-temperature waters in Southern Kamchatka. In *Proceedings of the Volcanology Laboratory of the Russian Academy of Sciences, Issue 19*; Moscow, 1961; pp. 90-98.
308. White, D.E.; Muffler, L.J.P.; Truesdell, A.N. Vapor-dominated hydrothermal systems compared with hot-water systems. *Econ Geol* **1971**, *66*, 75-97.
309. Fournier, R.O. *Geochemistry and Dynamics of the Yellowstone National Park Hydrothermal System*; US Geological Survey: Menlo Park, California, 2004.
310. Duchi, V.; Minissale, A.; Manganelli, M. Chemical composition of natural deep and shallow hydrothermal fluids in the Larderello geothermal field. *J Volcan Geotherm Res* **1992**, *49*, 313-328.
311. Bouwer, H. Geothermal power production with irrigation waste water. *Ground Water* **1979**, *17*, 375-384.
312. Jones, B.; Renaut, R.W.; Rosen, M.R. Silicified microbes in a geyser mound: the enigma of low-temperature cyanobacteria in a high-temperature setting. *Palaios* **2003**, *18*, 87-109.
313. Nersezova, E.E.; Rowe, M.C.; Campbell, K.A.; Ang, A.; Matthews, S.; Ruff, S.W.; Meghwal, A.; Adam, L.; Galligan, N.; Loho, T. Exploring the internal textures and physical properties of digitate sinter in hot springs: Implications for remote sampling on Mars. *Planetary and Space Science* **2023**, *238*, 105786.

314. Urusov, V.; Shvanskaya, L.; Bychkov, A.Y.; Mokhov, A.; Labutova, E. Microstructures of siliceous deposits of the Kamchatka hot springs. *Proc. Rus. Acad. Sci. Earth Sciences* **2008**, *418*, 123-127.
315. Urusov, V.S.; Shvanskaya, L.V.; Bychkov, A.Y.; Mokhov, A.V.; Labutova, E.A. Microstructure investigations of Kamchatka geyserites. *Moscow University Geology Bulletin* **2008**, *63*, 311-319.
316. Campbell, K.A.; Guido, D.M.; Gautret, P.; Foucher, F.; Ramboz, C.; Westall, F. Geyserite in hot-spring siliceous sinter: Window on Earth's hottest terrestrial (paleo) environment and its extreme life. *Earth-Science Reviews* **2015**, *148*, 44-64.
317. Lynne, B.Y.; Campbell, K.A.; Moore, J.N.; Browne, P.R.L. Diagenesis of 1900-year-old siliceous sinter (opal-A to quartz) at Opal Mound, Roosevelt Hot Springs, Utah, USA. *Sedimentary Geology* **2005**, *179*, 249-278.
318. Djokic, T.; Van Kranendonk, M.J.; Campbell, K.A.; Walter, M.R.; Ward, C.R. Earliest signs of life on land preserved in ca. 3.5 Ga hot spring deposits. *Nat Commun* **2017**, *8*, 15263, doi:10.1038/ncomms15263.
319. Baumgartner, R.J.; Van Kranendonk, M.J.; Pagès, A.; Fiorentini, M.L.; Wacey, D.; Ryan, C. Accumulation of transition metals and metalloids in sulfidized stromatolites of the 3.48 billion–year–old Dresser Formation, Pilbara Craton. *Precambrian Research* **2020**, *337*, 105534.
320. Djokic, T.; Van Kranendonk, M.J.; Campbell, K.A.; Havig, J.R.; Walter, M.R.; Guido, D.M. A Reconstructed Subaerial Hot Spring Field in the approximately 3.5 Billion-Year-Old Dresser Formation, North Pole Dome, Pilbara Craton, Western Australia. *Astrobiology* **2021**, *21*, 1-38, doi:10.1089/ast.2019.2072.
321. Van Kranendonk, M.J.; Baumgartner, R.; Djokic, T.; Ota, T.; Steller, L.; Garbe, U.; Nakamura, E. Elements for the Origin of Life on Land: A Deep-Time Perspective from the Pilbara Craton of Western Australia. *Astrobiology* **2021**, *21*, 39-59, doi:10.1089/ast.2019.2107.
322. Walter, M.R.; Buick, R.; Dunlop, J.S.R. Stromatolites 3,400–3,500 Myr old from the North pole area, Western Australia. *Nature* **1980**, *284*, 443-445.
323. Ueno, Y.; Isozaki, Y.; Yurimoto, H.; Maruyama, S. Carbon isotopic signatures of individual Archean microfossils (?) from Western Australia. *International Geology Review* **2001**, *43*, 196-212.
324. Van Kranendonk, M.J. Volcanic degassing, hydrothermal circulation and the flourishing of early life on Earth: A review of the evidence from c. 3490-3240 Ma rocks of the Pilbara Supergroup, Pilbara Craton, Western Australia. *Earth-Science Reviews* **2006**, *74*, 197-240.
325. Glikson, M.; Duck, L.J.; Golding, S.D.; Hofmann, A.; Bolhar, R.; Webb, R.; Baiano, J.C.F.; Sly, L.I. Microbial remains in some earliest Earth rocks: comparison with a potential modern analogue. *Precambrian Research* **2008**, *164*, 187-200.
326. Wacey, D.; Kilburn, M.R.; Saunders, M.; Cliff, J.; Brasier, M.D. Microfossils of sulphur-metabolizing cells in 3.4-billion-year-old rocks of Western Australia. *Nature Geoscience* **2011**, *4*, 698-702.
327. Gottesman, M.E.; Chudaev, M.; Mustaev, A. Key features of magnesium that underpin its role as the major ion for electrophilic biocatalysis. *FEBS J* **2020**, *287*, 5439-5463, doi:10.1111/febs.15318.
328. Rukuni, T.T.; Maree, J.P.; Carlsson, F.H.H. Investigation of carbonate dissolution for the separation of magnesium hydroxide and calcium sulphate in a magnesium hydroxide-calcium sulphate mixed sludge. *Water SA* **2015**, *41*, 253-262.
329. Rye, R.; Kuo, P.H.; Holland, H.D. Atmospheric carbon dioxide concentrations before 2.2 billion years ago. *Nature* **1995**, *378*, 603-605.
330. Lichtenegger, H.I.M.; Lammer, H.; Grießmeier, J.-M.; Kulikov, Y.N.; von Paris, P.; Hausleitner, W.; Krauss, S.; Rauer, H. Aeronomical evidence for higher CO2 levels during Earth's Hadean epoch. *Icarus* **2010**, *210*, 1-7.
331. Qian, X.; Gozani, S.N.; Yoon, H.; Jeon, C.J.; Agarwal, K.; Weiss, M.A. Novel zinc finger motif in the basal transcriptional machinery: three-dimensional NMR studies of the nucleic acid binding domain of transcriptional elongation factor TFIIS. *Biochemistry* **1993**, *32*, 9944-9959, doi:10.1021/bi00089a010.
332. Harding, M.M.; Nowicki, M.W.; Walkinshaw, M.D. Metals in protein structures: a review of their principal features. *Crystallography Reviews* **2010**, *16*, 247-302.
333. Andreini, C.; Bertini, I. A bioinformatics view of zinc enzymes. *J Inorg Biochem* **2012**, *111*, 150-156, doi:10.1016/j.jinorgbio.2011.11.020.

334. Mulkidjanian, A.Y.; Galperin, M.Y. On the origin of life in the zinc world. 2. Validation of the hypothesis on the photosynthesizing zinc sulfide edifices as cradles of life on Earth. *Biol Direct* **2009**, *4*, 27, doi:10.1186/1745-6150-4-27.
335. Maret, W. Zinc biochemistry: from a single zinc enzyme to a key element of life. *Adv Nutr* **2013**, *4*, 82-91, doi:10.3945/an.112.003038.
336. Krezel, A.; Maret, W. The Functions of Metamorphic Metallothioneins in Zinc and Copper Metabolism. *Int J Mol Sci* **2017**, *18*, doi:10.3390/ijms18061237.
337. Cuajungco, M.P.; Ramirez, M.S.; Tolmasky, M.E. Zinc: Multidimensional Effects on Living Organisms. *Biomedicines* **2021**, *9*, doi:10.3390/biomedicines9020208.
338. Mulkidjanian, A.Y. Energetics of the first life. In *Origind of Life: The Primal Self-organization*, Egel, E., Lankenau, D.-H., Mulkidjanian, A.Y., Eds.; Springer Verlag: Heidelberg, 2011; pp. 3-33.
339. Kallmann, H.; Sucov, E. Energy storage in ZnS and ZnCdS phosphors. *Physical Reviews* **1958**, *109*, 1473-1478.
340. Gratzel, M., (Ed.) *Energy Resources through Photochemistry and Catalysis* Academic Press: New York, 1983.
341. Henglein, A. Fluorescence, Photochemistry and Size Quantization Effects of Colloidal Semiconductor Particles. *Journal De Chimie Physique Et De Physico-Chimie Biologique* **1987**, *84*, 1043-1047.
342. Brus, L.E. Electron–electron and electron-hole interactions in small semiconductor crystallites: The size dependence of the lowest excited electronic state. *The Journal of chemical physics* **1984**, *80*, 4403-4409.
343. Henglein, A.; Gutierrez, M. Photochemistry of colloidal metal sulfides .5. Fluorescence and chemical reactions of ZnS and ZnS/CdS co-colloids. *Berichte Der Bunsen-Gesellschaft-Physical Chemistry Chemical Physics* **1983**, *87*, 852-858.
344. Henglein, A. Catalysis of photochemical reactions by colloidal semiconductors. *Pure Appl. Chem.* **1984**, *56*, 1215-1224.
345. Henglein, A.; Gutierrez, M.; Fischer, C.H. Photochemistry of colloidal metal sulfides. 6. Kinetics of interfacial reactions at ZnS particles. *Berichte Der Bunsen-Gesellschaft-Physical Chemistry Chemical Physics* **1984**, *88*, 170-175.
346. Kanemoto, M.; Shiragami, T.; Pac, C.J.; Yanagida, S. Semiconductor photocatalysis - effective photoreduction of carbon-dioxide catalyzed by ZnS quantum crystallites with low-density of surface-defects. *J. Phys. Chem.* **1992**, *96*, 3521-3526.
347. Eggins, B.R.; Robertson, P.K.J.; Stewart, J.H.; Woods, E. Photoreduction of carbon dioxide on zinc sulfide to give four-carbon and two-carbon acids. *J. Chem. Soc. Chem. Commun.* **1993**, 349-350.
348. Pan, H.; Heagy, M.D. Photons to formate: a review on photocatalytic reduction of CO2 to formic acid. *Nanomaterials* **2020**, *10*, 2422.
349. Das, S.; Daud, W.M.A.W. A review on advances in photocatalysts towards CO2 conversion. *Rsc Advances* **2014**, *4*, 20856-20893.
350. Kisch, H.; Künneth, R. Photocatalysis by semiconductor powders: Preparative and mechanistic aspects. In *Photochemistry and Photophysics*, Rabek, J., Ed.; CRC Press Inc.: 1991; pp. 131-175.
351. David, L.A.; Alm, E.J. Rapid evolutionary innovation during an Archaean genetic expansion. *Nature* **2011**, *469*, 93-96, doi:10.1038/nature09649.
352. Gulick, A. Phosphorus as a factor in the origin of life. *American Scientist* **1955**, *43*, 479-489.
353. Hanrahan, G.; Salmassi, T.M.; Khachikian, C.S.; Foster, K.L. Reduced inorganic phosphorus in the natural environment: significance, speciation and determination. *Talanta* **2005**, *66*, 435-444, doi:10.1016/j.talanta.2004.10.004.
354. Schwartz, A.W. Phosphorus in prebiotic chemistry. *Philos. Trans. R. Soc. Lond. B Biol. Sci.* **2006**, *361*, 1743-1749.
355. Pasek, M.A.; Kee, T.P.; Bryant, D.E.; Pavlov, A.A.; Lunine, J.I. Production of potentially prebiotic condensed phosphates by phosphorus redox chemistry. *Angew Chem Int Ed Engl* **2008**, *47*, 7918-7920, doi:10.1002/anie.200802145.
356. Pasek, M.; Herschy, B.; Kee, T.P. Phosphorus: a case for mineral-organic reactions in prebiotic chemistry. *Orig Life Evol Biosph* **2015**, *45*, 207-218, doi:10.1007/s11084-015-9420-y.
357. Herschy, B.; Chang, S.J.; Blake, R.; Lepland, A.; Abbott-Lyon, H.; Sampson, J.; Atlas, Z.; Kee, T.P.; Pasek, M.A. Archean phosphorus liberation induced by iron redox geochemistry. *Nat Commun* **2018**, *9*, 1346, doi:10.1038/s41467-018-03835-3.

358. Pasek, M.A. Thermodynamics of Prebiotic Phosphorylation. *Chem Rev* **2020**, *120*, 4690-4706, doi:10.1021/acs.chemrev.9b00492.
359. Figueroa, I.A.; Coates, J.D. Microbial phosphite oxidation and its potential role in the global phosphorus and carbon cycles. *Advances in Applied Microbiology* **2017**, *98*, 93-117.
360. Pech, H.; Henry, A.; Khachikian, C.S.; Salmassi, T.M.; Hanrahan, G.; Foster, K.L. Detection of geothermal phosphite using high-performance liquid chromatography. *Environmental Science & Technology* **2009**, *43*, 7671-7675.
361. Pech, H.; Vazquez, M.G.; Van Buren, J.; Foster, K.L.; Shi, L.; Salmassi, T.M.; Ivey, M.M.; Pasek, M.A. Elucidating the redox cycle of environmental phosphorus using ion chromatography. *Journal of chromatographic science* **2011**, *49*, 573-581.
362. Muenow, D.W.; Uy, O.M.; Margrave, J.L. Mass spectrometric studies of the vaporization of phosphorus oxides. *J. inorg, nucl. Chem* **1970**, *32*, 34359-33467.
363. Mambo, V.S.; Yoshida, M.; Matsuo, S. Partition of arsenic and phosphorus between volcanic gases and rock. Part I: Analytical data and magmatic conditions of Mt. Usu, Japan. *Journal of volcanology and geothermal research* **1991**, *46*, 37-47.
364. Nikolaeva, I.Y.; Tarnopolskaia, M.E.; Bychkov, A.Y. Experimental study of the phosphorus volatility in hydrothermal solutions. In Proceedings of the The 4th International Scientific and Practical Conference «Innovations in Geology, Geophysics and Geography–2019», 2019; pp. 64-65.
365. Pasek, M.A.; Sampson, J.M.; Atlas, Z. Redox chemistry in the phosphorus biogeochemical cycle. *Proc Natl Acad Sci U S A* **2014**, *111*, 15468-15473, doi:10.1073/pnas.1408134111.
366. White, A.K.; Metcalf, W.W. Microbial metabolism of reduced phosphorus compounds. *Annu. Rev. Microbiol.* **2007**, *61*, 379-400.
367. Feulner, G. The faint young Sun problem. *Reviews of Geophysics* **2012**, *50*.
368. Charnay, B.; Wolf, E.T.; Marty, B.; Forget, F. Is the faint young Sun problem for Earth solved? *Space Science Reviews* **2020**, *216*, 1-29.
369. Hoyle, F. Remarks on the computation of evolutionary tracks. In Proceedings of the Ricerche Astronomiche, Vol. 5, Specola Vaticana, Proceedings of a Conference at Vatican Observatory, Castel Gandolfo, May 20-28, 1957, Amsterdam: North-Holland, and New York: Interscience, 1958, edited by DJK O'Connell., p. 223, 1958; p. 223.
370. Sagan, C.; Mullen, G. Earth and Mars: evolution of atmospheres and surface temperatures. *Science* **1972**, *177*, 52-56, doi:10.1126/science.177.4043.52.
371. Moroz, V.; Mukhin, L. On early stages of evolution of the atmosphere and climate of the terrestrial planets. *Kosmicheskie Issledovaniia* **1977**, *15*, 901-922, doi:AN English translation is available at https://ui.adsabs.harvard.edu/abs/1977KosIs..15..901M/abstract.
372. Mukhin, L.M.; Moroz, V.I. Early evolution of the terrestrial atmosphere and hydrosphere. *Soviet Astronomy Letters* **1977**, *3*, 39-41.
373. Walzer, U.; Hendel, R. Natural climate change and glaciations. *Earth-Science Reviews* **2023**, 104435.
374. Walker, J.C.G.; Hays, P.B.; Kasting, J.F. A negative feedback mechanism for the long-term stabilization of Earth's surface temperature. *Journal of Geophysical Research: Oceans* **1981**, *86*, 9776-9782.
375. Moroz, V.I. Height of the Venusian clouds at equatorial and polar latitudes. *Nature Physical Science* **1971**, *231*, 36-37.
376. Moroz, V.; Golovin, Y.M.; Ekonomov, A.; Moshkin, B.; Parfent'Ev, N.; San'ko, N. Spectrum of the Venus day sky. *Nature* **1980**, *284*, 243-244.
377. Ekonomov, A.; Moroz, V.; Moshkin, B.; Gnedykh, V.; Golovin, Y.M.; Crigoryev, A. Scattered UV solar radiation within the clouds of Venus. *Nature* **1984**, *307*, 345-347.
